# A Survey of the Angular Distortion Landscape in the Coordination Geometries of High-Spin Iron(II) 2,6-Bis(pyrazolyl)pyridine Complexes

**DOI:** 10.1021/acs.inorgchem.3c04138

**Published:** 2024-01-23

**Authors:** Izar Capel Berdiell, Evridiki Michaels, Orde Q. Munro, Malcolm A. Halcrow

**Affiliations:** School of Chemistry, University of Leeds, Woodhouse Lane, Leeds LS2 9JT, U.K.

## Abstract

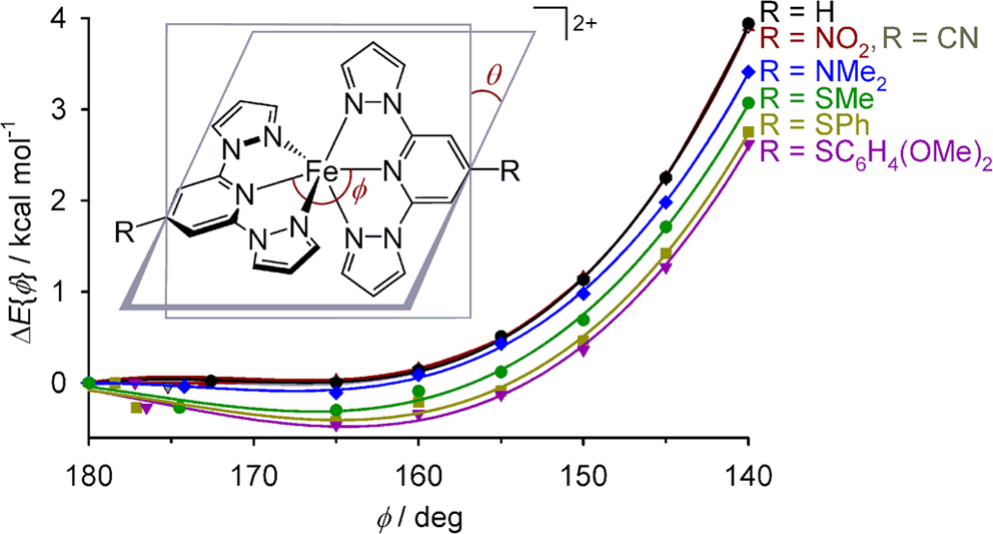

Reaction of 2,4,6-trifluoropyridine with sodium 3,4-dimethoxybenzenethiolate and 2 equiv of sodium pyrazolate in tetrahydrofuran at room temperature affords 4-(3,4-dimethoxyphenylsulfanyl)-2,6-di(pyrazol-1-yl)pyridine (*L*), in 30% yield. The iron(II) complexes [Fe*L*_2_][BF_4_]_2_ (**1a**) and [Fe*L*_2_][ClO_4_]_2_ (**1b**) are high-spin with a highly distorted six-coordinate geometry. This structural deviation from ideal *D*_2d_ symmetry is common in high-spin [Fe(bpp)_2_]^2+^ (bpp = di{pyrazol-1-yl}pyridine) derivatives, which are important in spin-crossover materials research. The magnitude of the distortion in **1a** and **1b** is the largest yet discovered for a mononuclear complex. Gas-phase DFT calculations at the ω-B97X-D/6-311G** level of theory identified four minimum or local minimum structural pathways across the distortion landscape, all of which are observed experimentally in different complexes. Small distortions from *D*_2d_ symmetry are energetically favorable in complexes with electron-donating ligand substituents, including sulfanyl groups, which also have smaller energy penalties associated with the lowest energy distortion pathway. Natural population analysis showed that these differences reflect greater changes to the Fe–N{pyridyl} σ-bonding as the distortion proceeds, in the presence of more electron-rich pyridyl donors. The results imply that [Fe(bpp)_2_]^2+^ derivatives with electron-donating pyridyl substituents are more likely to undergo cooperative spin transitions in the solid state. The high-spin salt [Fe(bpp)_2_][CF_3_SO_3_]_2_, which also has a strong angular distortion, is also briefly described and included in the analysis.

## Abstract 2

The computed energy profile of the angular Jahn−Teller distortion that is often shown by high-spin [Fe(bpp^R^)_2_]^2+^ (bpp^R^ = a 4-substituted-2,6-di(pyrazolyl)pyridine) derivatives depends on the nature of the ligand substituents. Crystalline salts of [Fe*L*_2_]^2+^ (*L* = 4-{3,4-dimethoxyphenylsulfanyl}-2,6-di{pyrazol-1-yl}pyridine) show the largest such distortion yet observed for this family of compounds, which are particularly important for spin-crossover materials research.

## Introduction

**Chart 1:**
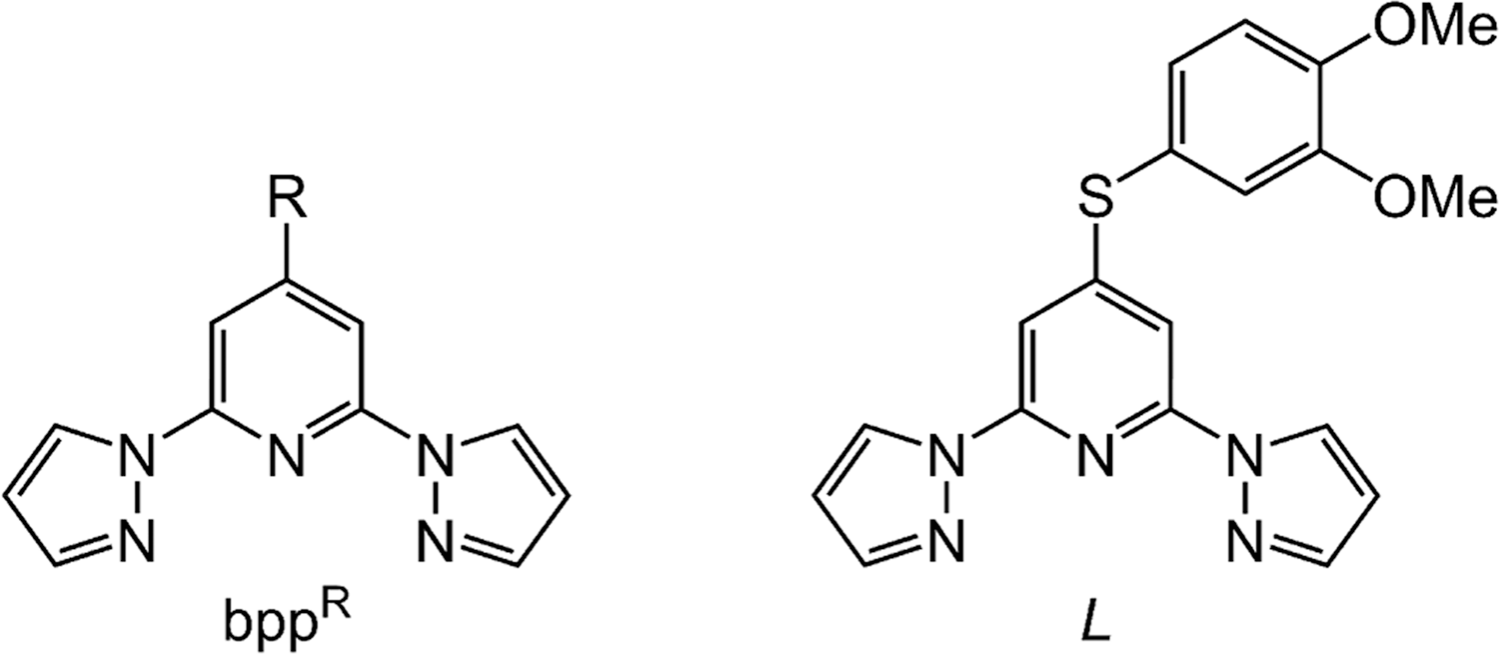
The 4-Substituted-2,6-Di(pyrazol-1-yl)pyridine (bpp^R^) Ligand Family, and the New Ligand *L*. The Prototype Ligand bpp Has R = H

Spin-crossover (SCO) materials^1−6^ continue to be of great interest in fundamental studies of crystal engineering;^7,8^ as components in switchable multifunctional materials;^9−14^ and for their applications in macroscopic and nanoscale devices.^15−19^ Iron(II) complexes of 2,6-di(pyrazol-1-yl)pyridine (bpp; Chart 1) and its derivatives are some of the most widely studied compounds for SCO research.^20−23^ Their popularity reflects that substituted bpp derivatives are readily accessible using appropriately substituted synthetic reagents, or by functional group transformations at preformed bpp precursors.^24^ The electronic and steric character of those substituents predictably influences the spin states of substituted [Fe(bpp)_2_]^2+^ derivatives in solution^25−27^ and, to a degree, in the solid state.^25,28−30^

**Figure 1:**
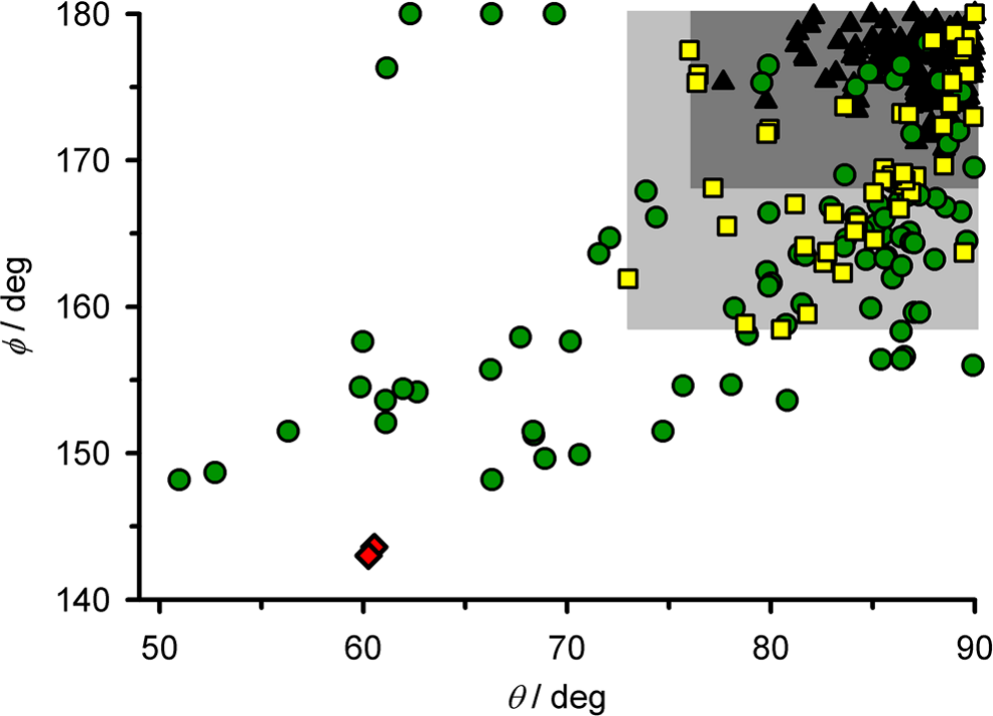
Plotted distortion parameters (Chart 2) from **1a**, **1b** (red diamonds), and other [Fe(bpp)_2_]^2+^ derivatives, updated from ref (20). Compounds that are low-spin (black triangles); high-spin and SCO-active (yellow squares); and high-spin and SCO-inactive (green circles) are plotted separately. High-spin complexes in the shaded parts of the graph commonly (dark gray) or more rarely (pale gray) exhibit SCO on cooling. Compounds in the unshaded part of the graph never exhibit SCO in the solid state.

**Chart 2:**
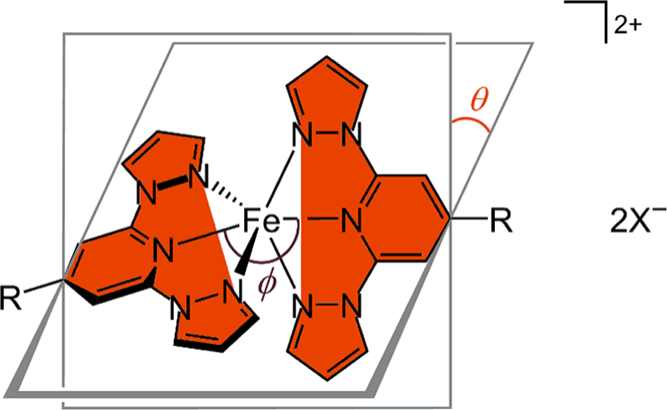
Distortion Parameters ϕ and θ in a [Fe(bpp^R^)_2_]X_2_ Derivative (Chart 1; X^–^ = a Monovalent Anion)a

However, a complication in this chemistry is that high-spin [Fe(bpp)_2_]^2+^ complexes are prone to structural distortions from their ideal *D*_2d_ molecular symmetry. This is characterized by a reduction in the *trans*-N{pyridyl}–Fe–N{pyridyl} angle (ϕ) from its ideal value of 180°, and a canting of the two bpp ligands away from the perpendicular, so the dihedral angle between them (θ) < 90° (Chart 2).^31^ This represents a geometric distortion along the octahedron (*O*_h_)–trigonal prism (*D*_3h_) coordination geometry pathway, within the conformational constraints of the bpp ligand.^22^ ϕ and θ can vary independently of each other and high-spin [Fe(bpp)_2_]^2+^ derivatives with 148 ≤ ϕ ≤ 180° and 50 ≤ θ ≤ 90° have been reported to date (Figure 1).^22,32^ Clustering of the data in Figure 1 implies there may be preferred structural ϕ vs θ pathways for the most strongly distorted molecules. That observation is addressed in this study.

a The least-squares planes of the ligands used to measure the dihedral angle θ are shaded in red.

While the distortion is most prevalent in [Fe(bpp)_2_]^2+^ chemistry, it also occurs in high-spin iron(II) complexes of other *tris*-heterocyclic ligands,^33−43^ and in related complexes of high-spin iron(III),^44−47^ manganese(II),^48−53^ cobalt(II),^54−58^ and zinc(II).^58−62^ In high-spin iron(II) complexes, the distortion electronically resembles a Jahn–Teller distortion of a ^5^*E* ground state (in *D*_2d_ symmetry), which is favored in complexes of tridentate ligands with a narrow bite angle.^31,63^ However, distortion also occurs in complexes with an orbital singlet ground state. In that case, the absence of a ligand field stabilization energy makes their coordination geometry more susceptible to deformations induced by crystal packing.

Since the distortion is a property of high-spin iron(II) complexes, their low-spin forms prefer regular geometries close to the *D*_2d_ ideal and are clustered in the top-right corner of Figure 1. Thus, some materials show a significant difference in ϕ and θ between the spin states, leading to a large structural rearrangement during SCO. That can be beneficial in some cases, by inducing cooperative spin transitions showing significant thermal hysteresis.^64−67^ Many SCO-active compounds whose high-spin structures lie inside the pale gray region of Figure 1 fall into that category. However, as ϕ and θ deviate more strongly from the regular geometries preferred by the low-spin complexes, a material is more likely to be kinetically trapped in its high-spin state upon cooling.^30,31,63^ Over half of the high-spin [Fe(bpp)_2_]^2+^ derivatives that have been crystallographically characterized to date fall into this category.

We now report the new ligand 4-(3,4-dimethoxyphenylsulfanyl)-2,6-di(pyrazol-1-yl)pyridine (*L*) and two salts of [Fe*L*_2_]^2+^. These compounds exhibit the largest angular distortion yet known for a mononuclear [Fe(bpp)_2_]^2+^ derivative, or any comparable molecule.^32^ Inspired by that discovery, we performed a comprehensive survey of the distortion map in Figure 1 using gas-phase DFT calculations. The results confirm the existence of more than one *ϕ* vs. θ distortion pathway. They also give insight into the relationship between the chemical structure and geometric preferences in these compounds, which is relevant to the design of new [Fe(bpp)_2_]^2+^ derivatives showing cooperative spin transitions.

## Experimental Section

Unless otherwise stated, reactions were carried out in air by using as-supplied reagents and AR-grade solvents. Synthetic details and characterization data for the new ligand *L* are given in the Supporting Information.

### Synthesis of [Fe*L*_2_][BF_4_]_2_ (**1a**)

Solutions of *L* (20 mg, 0.053 mmol) in acetonitrile (3 cm^3^) and Fe[BF_4_]_2_·6H_2_O (9 mg, 0.027 mmol) in acetonitrile (1 cm^3^) were mixed, affording an immediate bright yellow solution. Crystallization of the filtered solution by slow diffusion of diethyl ether antisolvent yielded a yellow polycrystalline material. Single crystals of this salt were grown by vapor diffusion methods from nitromethane/diethyl ether. Yield 20 mg, 77%. Found C, 46.3; H, 3.49; N, 14.0%. Calcd for C_38_H_34_B_2_F_8_FeN_10_O_4_S_2_ C, 46.2; H, 3.47; N, 14.2%. ESMS *m*/*z* 407.0764 (100%; calcd for [Fe*L*_2_]^2+^ 407.0778), 454.0422 (17%; calcd for [Fe*L*F]^+^ 454.0436), 833.1463 (23%; calcd for [Fe*L*_2_F]^+^ 833.1359), 901.1515 (13%; calcd for [Fe*L*_2_(BF_4_)]^+^ 901.1584). ^1^H NMR (CD_3_CN) δ 3.7, 3.9 (both 6H, OC*H*_3_), 6.5, 6.6, 7.1 (all 2H, Ph *H*^2^, *H*^5^ and *H*^6^), 41.6 (8H, Pz *H*^3^ and Py *H*^*3/*5^), 60.1 (4H, Pz *H*^4^), 71.1 (4H, Pz *H*^5^) ppm.

### Synthesis of [Fe*L*_2_][ClO_4_]_2_ (**1b**)

Method as for [Fe*L*_2_][BF_4_]_2_, using Fe[ClO_4_]_2_·6H_2_O (10 mg, 0.027 mmol). The product formed yellow single crystals from acetonitrile/diethyl ether. Yield 19 mg, 69%. Found C, 44.9; H, 3.19; N, 13.6%. Calcd for C_38_H_34_Cl_2_FeN_10_O_12_S_2_ C, 45.0; H, 3.38; N, 13.8%.

### Synthesis of [Fe(bpp)_2_][CF_3_SO_3_]_2_

Method as for [Fe*L*_2_][BF_4_]_2_, using bpp (50 mg, 0.24 mmol), Fe[CF_3_SO_3_]_2_ (42 mg, 0.12 mmol), and acetonitrile (5 cm^3^). The crude material is hygroscopic and must be recrystallized from dried solvent, but its single crystals are more stable under ambient conditions. Yellow prisms were obtained by diffusion of diethyl ether vapor into a solution of the complex in nitromethane, containing the drying agent triethyl orthoformate. Yield 76 mg, 82%. Found C, 37.2; H, 2.34; N, 17.6%. Calcd for C_24_H_18_F_6_FeN_10_O_6_S_2_ C, 37.1; H, 2.34; N, 18.0%. Full characterization of other salts of [Fe(bpp)_2_]^2+^ can be found in ref (31).

#### Single-Crystal Structure Analyses

Crystals of *L* were obtained from CDCl_3_ solution by slow evaporation; the crystallization procedure for the complex salts is described above. A crystal structure of the previously unpublished, highly distorted salt [Fe(bpp)_2_][CF_3_SO_3_]_2_ is also included in the Supporting Information. Diffraction data were collected with an Agilent Supernova diffractometer using monochromated Cu-K_α_ radiation (λ = 1.54184 Å). Experimental details of the structure determinations and full details of the crystallographic refinements are given in Table S1. The structures were solved by direct methods *(SHELX-TL*(68)) and developed by full least-squares refinement on *F*^2^ (*SHELXL2018*^69^). Crystallographic figures were produced using *XSEED*,^70^ and other publication materials were prepared with *OLEX*2.^71^

#### Other Measurements

CHN microanalyses were performed by the analytical service at the London Metropolitan University School of Human Sciences. Electrospray mass spectra were recorded on a Bruker MicroTOF-q instrument from a CHCl_3_ solution. Diamagnetic NMR spectra employed a Bruker AV3HD spectrometer operating at 400.1 MHz (^1^H) or 100.6 MHz (^13^C), while paramagnetic ^1^H NMR spectra were obtained with a Bruker AV3 spectrometer operating at 300.1 MHz. X-ray diffraction data were obtained with a Bruker D8 Advance A25 diffractometer using Cu-K_α_ radiation (λ = 1.5418 Å).

Solid-state magnetic measurements were performed on a Quantum Design MPMS-3 VSM magnetometer with an applied field of 5000 G and a scan rate of 5 K min^–1^. Diamagnetic corrections for the sample^72^ and for the sample holder were applied to these data. Evans method susceptibility measurements in solution were obtained using a Bruker AV-NEO spectrometer operating at 500.2 MHz.^73^ A diamagnetic correction for the sample^72^ and a correction for the temperature-dependence of the solution density^74^ were applied to these data.

#### Calculations

DFT minimizations were performed using *Spartan’20*(75) with the ω-B97X-D functional and 6-311G** basis set.^76^ The molecules are high-spin (*S* = 2) and were treated as spin-unrestricted. The calculations were performed in the gas phase because a solvent gradient for iron is not implemented in *Spartan’20*. Initial models were constructed *de novo* with the appropriate geometry restraints and then subjected to a preliminary molecular mechanics minimization before the full DFT geometry minimization calculation. Minimizations with fixed values of ϕ (Chart 2) were performed by fixing the relevant bond angle in the molecule. The θ and θ′ parameters could not be fixed exactly but were constrained by fixing the N{pyrazolyl}–Fe–N{pyridyl}–C{pyridyl} torsions to appropriate values.

Thermochemical data were obtained from frequency calculations on the freely minimized geometries of *S* = 0 and 2 [Fe*L*_2_]^2+^, according to the method of Cirera et al.^77^ Natural population analyses (NBO 3.0)^78^ were performed at the same level of theory via a single point calculation on the restrained geometry-optimized structures for [Fe(bpp)]^2+^ and [Fe(bpp^SMe^)_2_]^2+^ using *Gaussian* 16 Rev. C.01.^79^ Molecular orbitals were analyzed with *GaussView* 6.1.1^80^ using the *D*_2d_-symmetric structure of [Fe(bpp)_2_]^2+^ as the reference point. The MO with substantial iron 3d_*z*_2 atomic orbital character was considered to be the true direction of the molecular *z*-axis. The remaining d-orbitals (admixed with ligand MOs) were then labeled by inspection. The molecular axes used by Gaussian differ from the exact molecular point symmetry applied in the natural population analyses, which is discussed below.

## Results and Discussion

We originally synthesized 4-alkylsulfanyl-bpp (bpp^SR’^, Chart 1) derivatives by treating preformed bpp^SH^ with iodoalkanes in the presence of base.^81,82^ That reaction is low-yielding^67^ and is limited to electrophilic alkylation reagents. These drawbacks are addressed by the alternative method of treating 2,4,6-trifluoropyridine with a sodium thiolate reagent, then with 2 equiv of sodium pyrazolate, in a one-pot reaction. That approach also presents a purification challenge but is more flexible and involves fewer synthesis steps overall.^83,84^ Such a reaction employing sodium 3,4-dimethoxyphenylthiolate afforded *L* in 30% yield after purification by flash silica chromatography. The identity of *L* was confirmed crystallographically, in addition to the usual analytical characterization (Figure S3).

**Figure 2:**
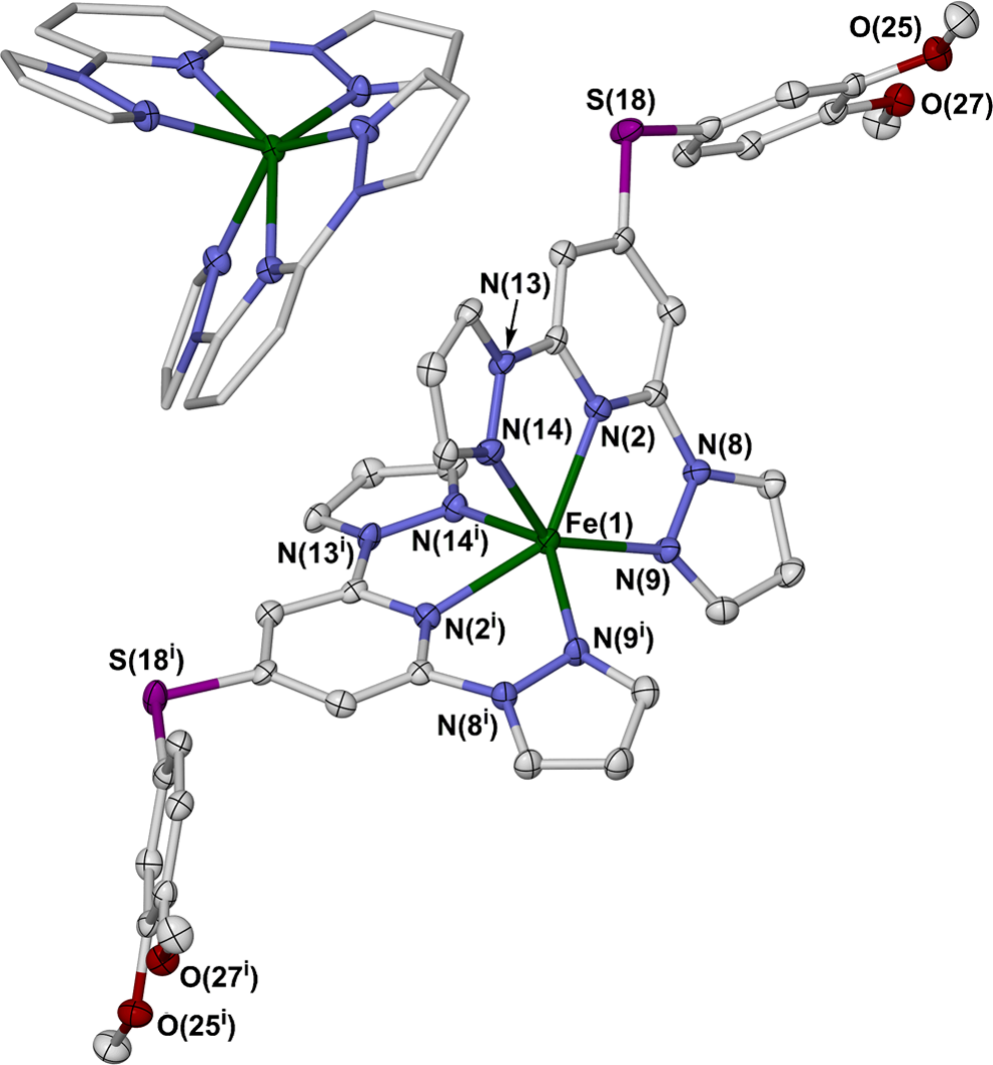
[Fe*L*_2_]^2+^ cation in **1b** with selected atom numbering, and (inset) the [Fe(bpp)_2_]^2+^ core of the molecule, highlighting its coordination geometry. Displacement ellipsoids are at the 50% probability level, and H atoms are omitted for clarity. Symmetry code: (i) 1–*x*, *y*, 3/2–*z*. Color code: C, white; Fe, green; N, blue; O, red; S, purple.

Complexation of Fe[BF_4_]_2_·6H_2_O or Fe[ClO_4_]_2_·6H_2_O with 2 equiv of *L* in acetonitrile solution afforded yellow [Fe*L*_2_][BF_4_]_2_ (**1a**) and [Fe*L*_2_][ClO_4_]_2_ (**1b**) after the usual workup. Crystals of **1a** and **1b** are isomorphous, in the monoclinic space group *C*2/*c* with *Z* = 4. The asymmetric unit contains half a complex cation with Fe(1) on a crystallographic *C*_2_ axis and one unique anion. The metric parameters at Fe(1) are typical of high-spin [Fe(bpp)_2_]^2+^ derivatives, with a very strong angular distortion (Figure 2, Table 1). The ϕ angle is significantly smaller than for any other [Fe(bpp)_2_]^2+^ derivative reported to date, while the θ distortion is also close to the record value for a mononuclear complex (Figures 1 and S1). The iron atom protrudes from the plane of each tridentate ligand by 0.478(6)-0.480(4) Å, and there are weak Fe···F or Fe···O secondary contacts of *ca* 3.2 Å between the anions and the open face of Fe(1) (Table S2). Both features are typical for [Fe(bpp)_2_]^2+^ derivatives with large angular distortions.^22^

**Table 1:** Selected Bond Lengths (Å) and Other Structural Parameters for the Metal Complex Crystal Structures^a^^,^^b^

	**1a**	**1b**
Fe(1)–N(2)	2.163(5)	2.171(4)
Fe(1)–N(9)	2.169(5)	2.180(4)
Fe(1)–N(14)	2.218(6)	2.215(4)
*V*_Oh_/Å^3^	11.02(2)	10.991(16)
Σ/°	203.6(7)	208.5(5)
Θ/°	500	506
ϕ/°	143.6(3)	143.0(2)
θ/°	60.53(6)	60.25(5)
θ′/°	67.79(19)	66.45(15)

a The atom numbering scheme is shown in Figure 2, and ϕ, θ, and θ′ are defined in the text.

b *V*_Oh_ is the volume of the FeN_6_ inner coordination sphere, while Σ and Θ are bond angle parameters characteristic for the spin state of a complex.^86,87^ Full definitions of these parameters are given in the Supporting Information.

The cations associate into layers in the (101) crystal plane through columns of face-to-face π···π interactions between their phenyl substituents (Figure S6). The dimethoxyphenylsulfanyl groups adopt very similar conformations in the complex and free ligand crystals. The C–S–C group lies in the plane of the pyridyl ring, which facilitates conjugation between the S lone pairs and the pyridyl π-system.^85^ The phenyl and pyridyl rings on each ligand are almost perpendicular to each other with a dihedral angle of 73.8(2)–77.10(14)° (Table S2).

Samples of **1a** and **1b** are phase-pure by powder diffraction and are high-spin between 5 and 300 K as expected. A complex that is high-spin in the solid state could still exhibit SCO in solution, where its conformation is more flexible. However, **1b** is also high-spin within experimental error over the liquid range of CD_3_CN (233–343 K). Our correlation for [Fe(bpp^R^)_2_]^2+^ derivatives^26^ predicts a much lower SCO midpoint temperature (*T*_1/2_) of 188 K in solution for [Fe*L*_2_]^2+^, bearing SAr (Ar = aryl) pyridyl substituents with a σ_P_^+^ Hammett parameter of −0.55.^88^

Calculations of high-spin [Fe(bpp)_2_]^2+^ and related complexes have shown distortions of 180 ≤ ϕ ≤ 155° carry an energy penalty of only *ca.* 1 kcal mol^–1^ in the gas phase.^31,63,89−91^ That implies both distorted and undistorted geometries should be accessible at room temperature, and the data in Figure 1 should be influenced by crystal packing effects.^63^ Hence, we have now systematically investigated the larger distortion in **1a** and **1b** with DFT calculations over a wider distortion range. The calculations were performed in the gas phase, using the ω-B97X-D GGA functional and 6-311G** basis set.^76^ That protocol was chosen because it includes a correction for intramolecular dispersion interactions between nonbonded atoms which can be important for accurate descriptions of Jahn–Teller-active distortions.^63,90,92^ It also performed well on energy minimizations of a family of Jahn–Teller-active molecular magnets, in another study from our lab.^93^

**Chart 3:**
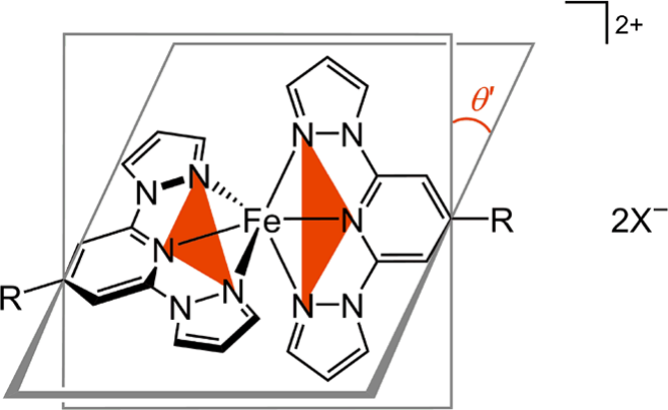
Planes of the N-Donor Atoms of the Two Ligands Used to Measure the Dihedral Angle θ′ in a [Fe(bpp^R^)_2_]X_2_ Derivative (Shaded in Red; *cf.*Chart 2)

During the analysis, we found that θ is influenced by bowl-shaped or S-shaped ligand conformations that are often found in highly distorted [Fe(bpp^R^)_2_]^2+^ complexes (Figures 2 and S11). These ligand conformations are influenced by crystal packing effects, which are not described by gas-phase calculations. That was accounted for using an alternative parameter to define the computed angular distortion, θ′ which ignores the peripheral ligand atoms. θ′ is the dihedral angle between the planes formed by the three N-donor atoms on each ligand (Chart 3). Experimental or computed θ and θ′ values for a given structure are often similar, but can differ by up to 15° in highly distorted molecules (Table S4). Experimental θ′ values give better agreement with the computational results, and the θ′ parameter is used in the following discussion.

**Figure 3:**
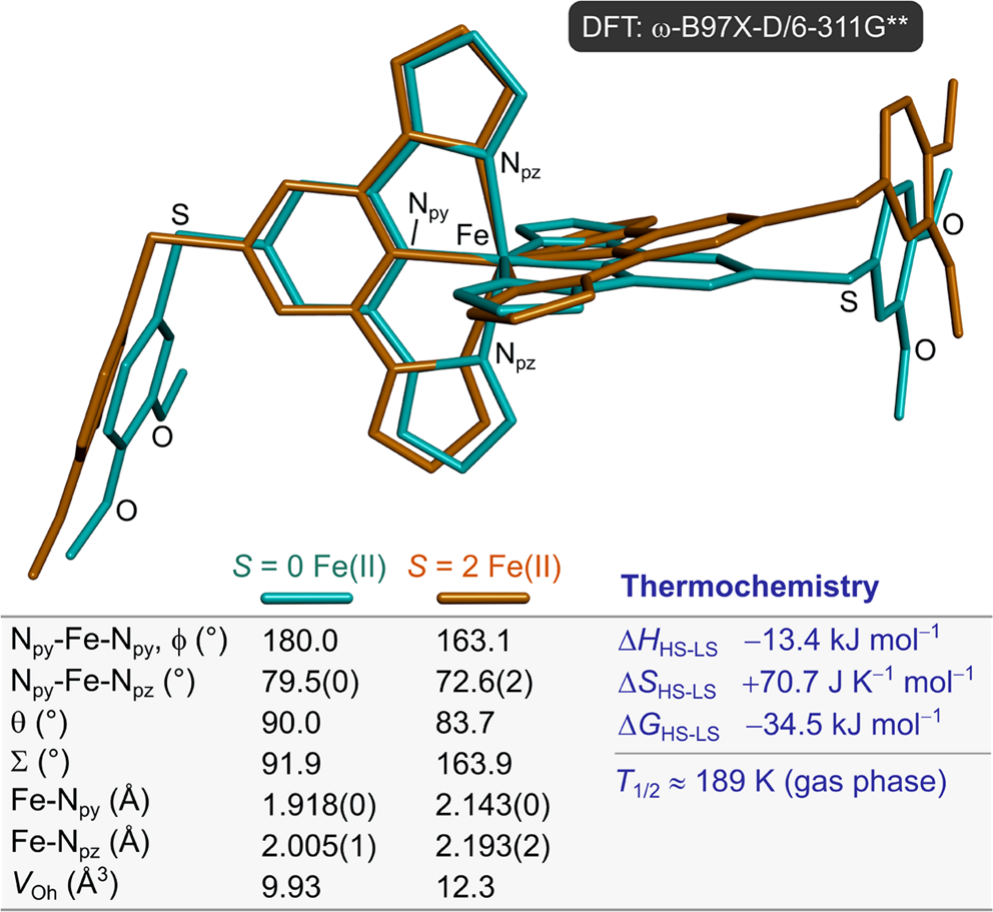
Comparison of the freely minimized structures for *S* = 0 and 2 [Fe*L*_2_]^2+^, illustrating the marked distortion for the high-spin complex in the absence of crystal packing effects. Selected structural parameters and atom types are listed (defined in Table 1). The structures were superimposed by regression-fitting based on their FeN_6_ coordination groups; RMSD = 33.6%. (HS = high-spin; LS = low-spin).

Initial unconstrained minimizations of high- and low-spin [Fe*L*_2_]^2+^ confirm that the high-spin state is thermodynamically favored at 298 K (Δ*G*^298^ = *G*_HS_^298^–*G*_LS_^298^ = −34.5 kJ mol^–1^; Figure 3). The estimated gas-phase *T*_1/2_ for the system is 189 K, in excellent agreement with the empirical prediction above.^26^ The low-spin complex minimizes to an almost perfect *D*_2d_-symmetric coordination geometry, as expected.^63,89−91^ The *S* = 2 cation minimizes with an angular distortion (ϕ_calc_ = 163.9°, θ_calc_ = 83.7°), but this is significantly less pronounced than the crystallographic structures (ϕ = 143°, θ = 60°; Table 1). That supports the importance of crystal packing interactions to the observed molecular geometry in the crystalline solid state.

As a starting point for the angular distortion analysis, [Fe(bpp)_2_]^2+^ and the other molecules investigated (see below) were minimized in an undistorted geometry. Minimizations with fixed ϕ = 180° and θ′ = 90° did not converge, presumably because they did not allow manifestation of Jahn–Teller effects in the ^5^*E* high-spin iron(II) centers (in *D*_2d_ notation). Minimizations starting from a *D*_2d_-symmetric coordination geometry, with *N*{pyrazolyl}–Fe–*N*{pyridyl}–C{pyridyl} torsions fixed at ±90°, were successful if the ligand donor groups had conformational freedom to twist slightly. These “undistorted” minimized energies were used as the reference values in the following discussion.

A survey of the ϕ vs θ′ landscape using the parent complex [Fe(bpp)_2_]^2+^ identified four distinct structural distortion pathways. The molecule was first minimized by fixing ϕ at values between 165 ≥ ϕ ≥ 140° and allowing θ′ to refine freely. An additional data point between 180 > ϕ > 165° was obtained by performing an unconstrained minimization beginning from ϕ = 170°.^94^ The energy of the distortion from *D*_2d_ symmetry at a particular ϕ angle was calculated according to eq 1.
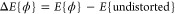
1

**Figure 4:**
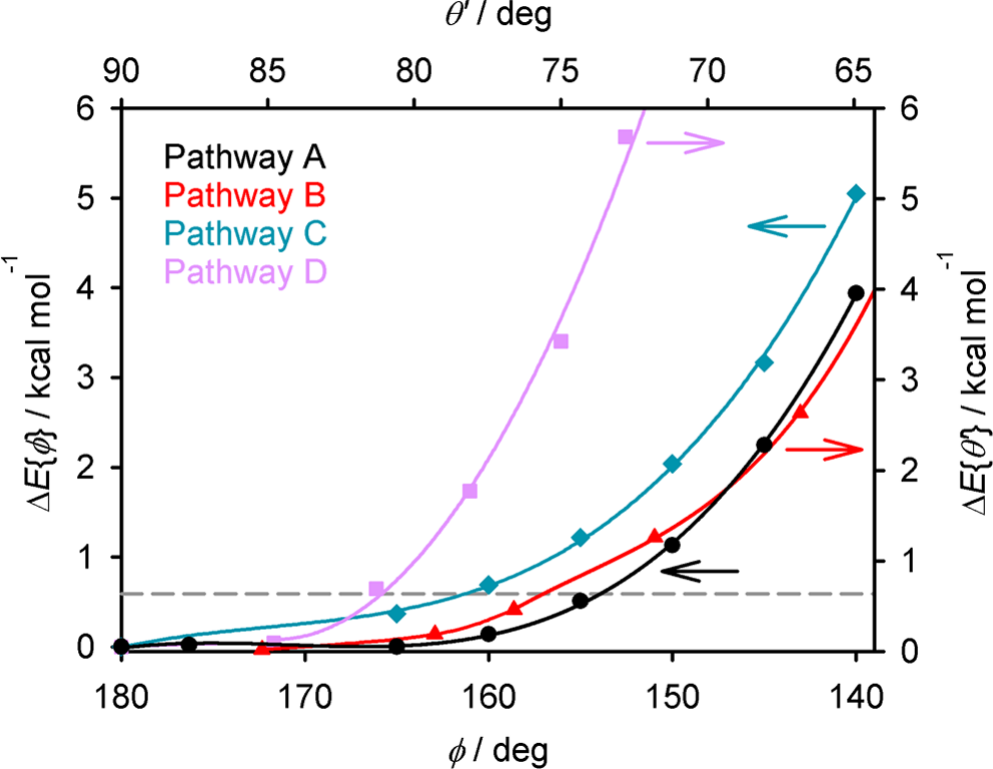
Minimized energies of distortion pathways A-D for [Fe(bpp)_2_]^2+^, plotted against the parameter that was fixed or constrained during the minimization of each pathway (indicated by the arrows). The two horizontal axes are scaled such that plots of pathway A against ϕ and θ′ overlap on the graph. The dotted line indicates *kT* at room temperature (0.6 kcal mol^–1^).

The results are shown in Table 2 and plotted in Figure 4, as pathway A. As expected, Δ*E*{ϕ} increases as ϕ decreases, reaching +3.9 kcal mol^–1^ at ϕ = 140° (Figure 4). The relationship is nonlinear, and distortions of 180 > ϕ ≥ 155° along this pathway carry an energy penalty below *kT* at room temperature (0.6 kcal mol^–1^).

**Table 2:** Energies and Structural Indices for [Fe(bpp)_2_]^2+^ Minimized along Distortion Pathways A–D (Chart 4)^a^

pathway A								
ϕ/°	180.0^b^	172.6	165.0^c^	160.0^c^	155.0^c^	150.0^c^	145.0^c^	140.0^c^
θ/°	89.0^b^	87.0	84.1	82.2	78.9	74.2	71.3	68.8
θ′/°	88.0^b^	85.2	82.0	78.7	74.8	71.0	68.0	65.0
Δ*E*{ϕ}/kcal mol^–1^^d^	0	+0.02	0.00	+0.14	+0.51	+1.13	+2.25	+3.94
pathway B								
ϕ/°	180.0^b^	164.4	163.8	160.8	157.9	157.3	157.0	
θ/°	89.0^b^	79.8	77.3	70.8	65.1	59.8	55.3	
θ′/°	88.0^b^	79.3^d^	76.6^d^	71.8^d^	66.8^d^	63.6^d^	60.6^d^	
Δ*E*{θ′}/kcal mol^–1^^e^	0	+0.19	+0.46	+1.26	+2.63	+4.49	+7.12	
Pathway C								
ϕ/°	180.0^b^		165.0^c^	160.0^c^	155.0^c^	150.0^c^	145.0^c^	140.0^c^
θ/°	89.0^b^		89.4	89.4	89.3	89.3	87.9	88.1
θ′/°	88.0^b^		89.5	89.5	89.2	89.2	87.5	87.4
Δ*E*{ϕ}/kcal mol^–1^^e^	0		+0.37	+0.69	+1.22	+2.04	+3.17	+5.05
pathway D								
ϕ/°	180.0^b^	180.0	179.9	179.8	179.8	180.0	180.0	
θ/°	89.0^b^	85.3	80.3	75.2	69.7	64.1	58.3	
θ′/°	88.0^b^	84.8^d^	81.3^d^	78.1^d^	75.0^d^	72.2^d^	69.6^d^	
Δ*E*{θ′}/kcal mol^–1^^e^	0	+0.05	+0.65	+1.74	+3.40	+5.68	+8.47	

a Pathways A and C were minimized by fixing ϕ at different values, with no other restraints. Pathways B and D were accessed by constraining θ′ with fixed interligand torsions, while allowing ϕ to refine. Computed energies for each minimization are listed in Table S6.

b This is the “undistorted” minimization of this molecule where θ was constrained to be near, but not exactly, 90°—see the text for more details.

c Fixed value.

d Constrained during the minimization—see the text for details.

e Δ*E*{*i*} = *E*{*i*} – *E*{undistorted} (*i* = ϕ or θ′).

**Chart 4:**
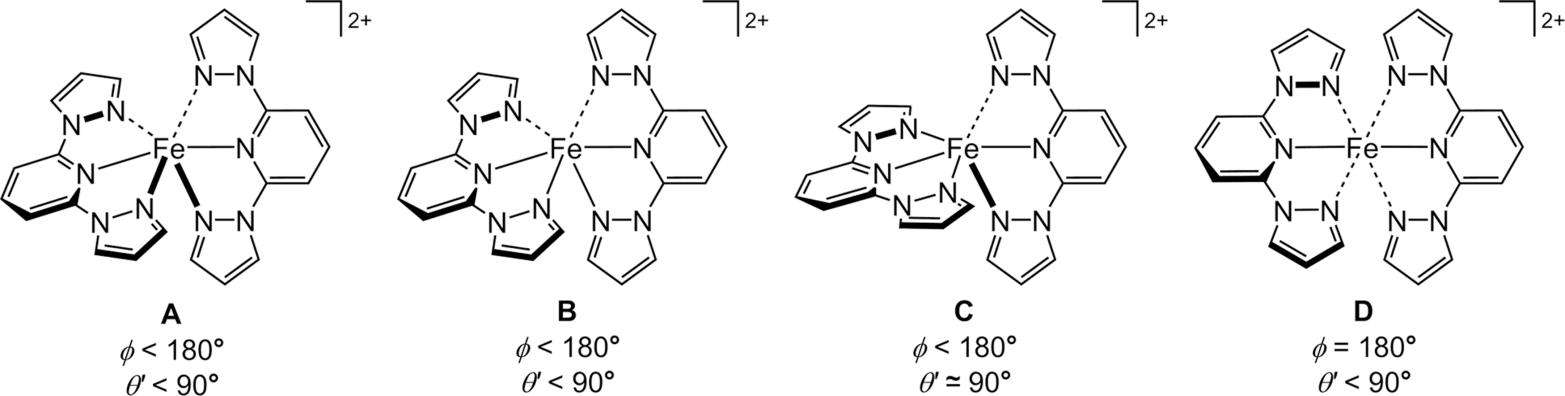
Schematic of the Distortion Pathways Identified during the Computational Study of [Fe(bpp)_2_]^2+^a

The minimized geometries with ϕ ≤ 165° have exact or approximate *C*_2_ symmetry, and θ′ component of the distortion deviates more strongly from 90° as ϕ is lowered. One Fe–N{pyrazolyl} bond to each ligand becomes successively longer and the other shorter as ϕ decreases, giving a [2 + 2+2] Fe–N bond distribution; the short Fe–N{pyrazolyl} bonds are shorter than the Fe–N{pyridyl} bonds at ϕ < 150° (Table S7, Chart 4). These results are consistent with the earlier computational studies.^31,63,89−91^

a Thick, thin, and dashed bonds correspond to short, medium, and long Fe–N distances in the most distorted molecules minimized along each pathway (Tables S7–S10).

Since θ′ could not be fixed during the minimizations, it was constrained by fixing the interligand N{pyrazolyl}–Fe–N{pyridyl}–C{pyridyl} torsions to different values. Letting ϕ refine with these torsional constraints gave access to a second distortion pathway B (Chart 4). θ′ adopts a range of values in this distortion, but ϕ spans a narrower range of 164 ≥ ϕ ≥ 157°. The Fe–N bonds evolve toward a [4 + 2] distribution along this pathway, with the Fe–N{pyridyl} and two Fe–N{pyrazolyl} distances being equal in minimizations with θ′ < 67° (Table S8). Pathway B could only be distinguished from pathway A in the minimizations when θ′ was constrained to be <80°.

An alternative pathway in ϕ was also located, where θ′ remains near 90° (pathway C, Chart 4). This was minimized without additional constraints if the two bpp ligands were strictly perpendicular in the starting model. This angular distortion leads to the evolution of the Fe–N bond lengths toward [5 + 1]-coordination (Table S9). Lastly, a distortion in θ′ only was achieved by constraining θ′ to a range of values while keeping ϕ = 180° in the starting model (pathway D, Chart 4). The bpp ligands in this distortion remain symmetrically coordinated, but the Fe–N{pyrazolyl} distances become successively longer as the distortion proceeds; the Fe–N{pyridyl} bonds are less affected (Table S10).

At moderate or large distortions, the relative energies of the four pathways run as A ≈ B < C ≪ D (Table 2, Figure 4). However, the distortion energies of pathways A and C with ϕ ≥ 160°, and pathway B with θ′ ≥ 75°, are all ≤ *kT* at room temperature by this protocol. Hence, molecules should access those pathways interchangeably under ambient conditions.

The influence of ligand substituents on the distortion was investigated by minimizing six other [Fe(bpp^R^)_2_]^2+^ derivatives (Chart 1) across pathway A (Table 3). Calculations were performed on [Fe(bpp^NO_2_^)_2_]^2+^ and [Fe(bpp^CN^)_2_]^2+^ as [Fe(bpp^R^)_2_]^2+^ molecules bearing electron-withdrawing substituents; [Fe(bpp^NMe_2_^)_2_]^2+^ and [Fe(bpp^SMe^)_2_]^2+^ with electron-donating R-groups; and two other sulfanyl complexes, [Fe(bpp^SPh^)_2_]^2+^ and [Fe*L*_2_]^2+^ itself.

**Table 3:** Energies and Selected Structural Parameters of the Minimized High-Spin [Fe(bpp^R^)_2_]^2+^ Complexes (R ≠ H; Chart 1), along Distortion Pathway A (Chart 4)^a^

[Fe(bpp^NO_2_^)_2_]^2+^								
ϕ/°	180.0	173.0	165.0^b^	160.0^b^	155.0^b^	150.0^b^	145.0^b^	140.0^b^
θ/°	89.3	86.0	84.0	81.9	78.5	74.0	71.3	68.7
θ′/°	88.2	85.0	81.7	78.6	75.0	71.0	68.1	65.4
Δ*E*{ϕ}/kcal mol^–1^	0	0.00	+0.03	+0.16	+0.49	+1.16	+2.26	+3.90
[Fe(bpp^CN^)_2_]^2+^								
ϕ/°	180.0	175.2	165.0^b^	160.0^b^	155.0^b^	150.0^b^	145.0^b^	140.0^b^
θ/°	89.2	87.0	84.0	81.8	78.4	74.0	71.3	68.8
θ′/°	88.2	85.6	81.6	78.5	75.0	71.0	68.2	65.5
Δ*E*{ϕ}/kcal mol^–1^	0	–0.04	+0.01	+0.14	+0.49	+1.13	+2.24	+3.91
[Fe(bpp^NMe_2_^)_2_]^2+^								
ϕ/°	179.9	174.2	165.0^b^	160.0^b^	155.0^b^	150.0^b^	145.0^b^	140.0^b^
θ/°	89.3	89.1	86.8	84.4	80.9	76.5	74.1	72.3
θ′/°	89.7	88.5	85.5	82.1	78.4	74.8	72.5	70.4
Δ*E*{ϕ}/kcal mol^–1^	0	–0.04	–0.11	–0.09	+0.43	+0.98	+1.98	+3.41
[Fe(bpp^SMe^)_2_]^2+^								
ϕ/°	180.0	174.5	165.0^b^	160.0^b^	155.0^b^	150.0^b^	145.0^b^	140.0^b^
θ/°	89.0	88.9	85.1	82.5	76.5	74.4	73.2	68.3
θ′/°	88.1	88.6	83.6	80.2	76.3	73.6	71.9	68.6
Δ*E*{ϕ}/kcal mol^–1^	0	–0.27	–0.30	–0.09	+0.12	+0.69	+1.71	+3.07
[Fe(bpp^SPh^)_2_]^2+^								
ϕ/°	178.4^c^	177.1	165.0^b^	160.0^b^	155.0^b^	150.0^b^	145.0^b^	140.0^b^
θ/°	88.9	89.7	85.5	82.9	76.7	75.0	73.9	68.3
θ′/°	88.2	90.0	84.2	80.3	76.6	74.2	72.5	68.9
Δ*E*{ϕ}/kcal mol^–1^	0	–0.27	–0.42	–0.21	–0.09	+0.46	+1.42	+2.75
[Fe*L*_2_]^2+^								
ϕ/°	177.2^c^	176.5	165.0^b^	160.0^b^	155.0^b^	150.0^b^	145.0^b^	140.0^b^
θ/°	89.0	89.6	85.1	82.0	77.3	74.6	71.6	68.9
θ′/°	88.0	89.5	84.1	80.7	77.1	74.3	71.8	69.7
Δ*E*{ϕ}/kcal mol^–1^	0	–0.27	–0.46	–0.34	–0.13	+0.36	+1.27	+2.62

a The computed energies for each minimization are listed in Table S11. Other details are given in Table 2.

b Fixed value.

c The idealized point symmetry of the undistorted complexes is *D*_2d_ for [Fe(bpp)_2_]^2+^, [Fe(bpp^NO_2_^)_2_]^2+^, and [Fe(bpp^NMe_2_^)_2_]^2+^, but *C*_2_ for [Fe(bpp^SMe^)_2_]^2+^, [Fe(bpp^SPh^)_2_]^2+^, and [Fe*L*_2_]^2+^. Hence, ϕ is not constrained by symmetry to be 180° for the undistorted sulfanyl-substituted molecules.

**Figure 5:**
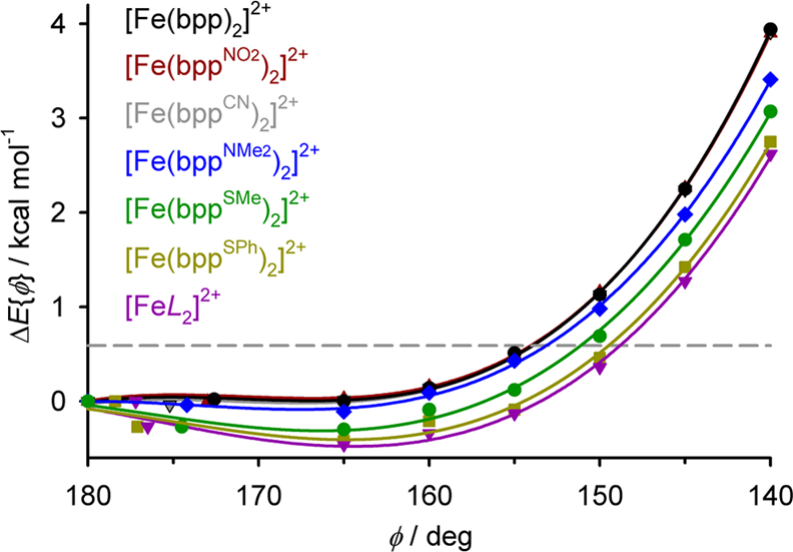
Minimized energies of seven [Fe(bpp^R^)_2_]^2+^ molecules along distortion pathway A (Table 3). Data for each molecule are connected by a regression curve, and the data for [Fe(bpp)_2_]^2+^, [Fe(bpp^NO_2_^)_2_]^2+^ and [Fe(bpp^CN^)_2_]^2+^ are superimposed on each other in the graph. Other details are given in Figure 4.

The Δ*E*{ϕ} energies of [Fe(bpp^NO_2_^)_2_]^2+^ and [Fe(bpp^CN^)_2_]^2+^ are identical to [Fe(bpp)_2_]^2+^ at each value of ϕ (Tables 2 and 3; Figure 5), showing electron-withdrawing ligand substituents have no impact on the energetics of the distortion. However, the energy penalty associated with the distortion is smaller for molecules with electron-donating ‘R’ substituents. The lowest Δ*E*{ϕ} values for each geometry were obtained for [Fe(bpp^SPh^)_2_]^2+^ and [Fe*L*_2_]^2+^, which were equal within experimental error for each value of ϕ. Hence, the methoxy substituents in [Fe*L*_2_]^2+^ have little electronic impact on its coordination geometry. The energy penalty for the strongest computed distortion, with ϕ = 140°, is only about two-thirds as large for [Fe*L*_2_]^2+^ (+2.6 kcal mol^–1^) as for [Fe(bpp)_2_]^2+^ (+3.9 kcal mol^–1^).

While Δ*E*{ϕ} is ≥0 for [Fe(bpp)_2_]^2+^, [Fe(bpp^NO_2_^)_2_]^2+^ and [Fe(bpp^CN^)_2_]^2+^, the lowest energy geometry for the complexes with π-donor substituents is near ϕ ≈ 165°, not 180°. This reduction in ϕ leads to a computed energy gain of no more than −0.4 kcal mol^–1^. However, it is noteworthy that most published crystal structures of high-spin [Fe(bpp^NR’2^)_2_]^2+^, [Fe(bpp^NHC{O}Me^)_2_]^2+^, and [Fe(bpp^SR’^)_2_]^2+^ (R’ = H or alkyl) derivatives exhibit ϕ = 165 ± 4° (see below). While the amino-substituted complexes are high-spin,^26,95,96^ many [Fe(bpp^NHC{O}Me^)_2_]^2+^ and [Fe(bpp^SR’^)_2_]^2+^ salts exhibit cooperative spin transitions.^65,67,81,82,97−99^ That reflects the structural consequences of increasing ϕ from *ca*. 165° toward 180° in the low-spin state, on the lattice energy of the crystal.^63^

**Figure 6:**
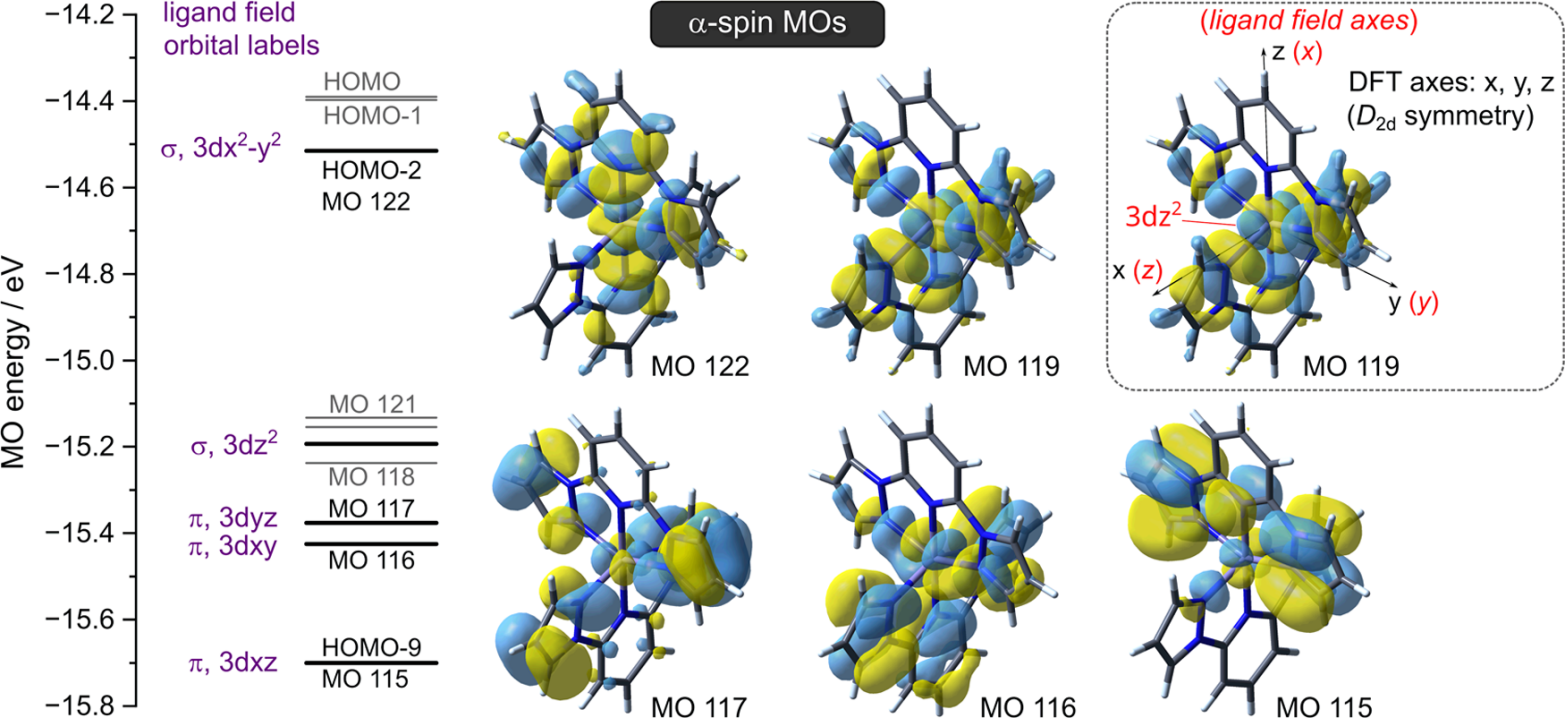
Energy-level scheme for the top ten α-spin singly occupied molecular orbitals for the undistorted structure of high-spin [Fe(bpp)_2_]^2+^ with ϕ = 180°. The MOs with substantial *d*-orbital character are indicated. The inset shows the Cartesian axis convention used to assign the *d*-orbital labels by the natural population analysis (the ligand field axes shown in red) and, for comparison, the more familiar axis convention corresponding to idealized *D*_2d_ molecular symmetry (black).^99^ MO plots from a distorted minimization of [Fe(bpp)_2_]^2+^ are in Figure S25.

To probe the different energy profile of pathway A in the presence and absence of π-donor substituents, the *d*-orbital energies for each minimized structure of [Fe(bpp)_2_]^2+^ and [Fe(bpp^SMe^)_2_]^2+^ were calculated by natural population analysis, also at the ω-B97X-D/6-311G** level of theory.^100^ A complication is that the Cartesian axis convention produced by the analysis is different from expectation, based on the idealized *D*_2d_ symmetry of undistorted [Fe(bpp)_2_]^2+^ (Figures 6 and S24). That reflects the small deviations from *D*_2d_ symmetry in the undistorted structures that were required for successful minimization.^101^

**Figure 7:**
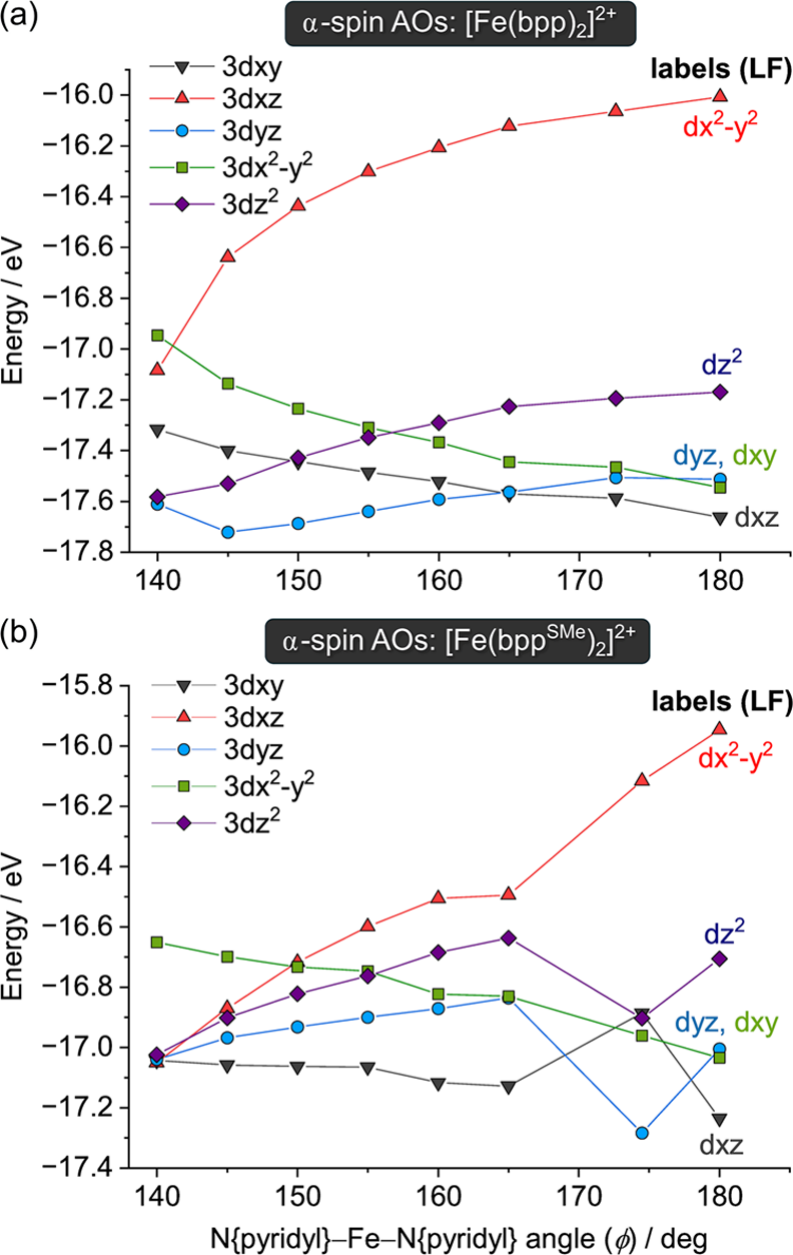
Variation of the natural atomic d-orbital energies with ϕ along pathway A for: (a) [Fe(bpp)_2_]^2+^; (b) [Fe(bpp^SMe^)_2_]^2+^. The orbital labels use the ligand field axis convention in Figure 6.

The variation of the *d*-orbital energies for both molecules at different stages of distortion is shown in Figure 7. The clearest difference between them is that the energy of *d*_*x*_2_–*y*_2 in [Fe(bpp^SMe^)_2_]^2+^ decreases much more rapidly as the distortion proceeds than for [Fe(bpp)_2_]^2+^. This indicates that the Fe–N{pyridyl} σ-bonding along the molecular *x* axis (Figure 6) is weakened more strongly by the distortion in [Fe(bpp^SMe^)_2_]^2+^. The electron-donating sulfanyl substituents in [Fe(bpp^SMe^)_2_]^2+^ make its pyridyl donors more electron-rich.^85^ Hence, the distortion has a proportionately larger effect on the stronger Fe–N{pyridyl} σ-bonding in that complex, as the Fe–N{pyridyl} bonds move off-axis. That is the likely origin of the different energy profiles for [Fe(bpp^R^)_2_]^2+^ along the distortion pathway when R is an electron-donating substituent.

The *d*-orbital splitting is reduced by the distortion. While this is difficult to quantify, it occurs more rapidly for [Fe(bpp^SMe^)_2_]^2+^ as ϕ decreases (Figure 7). Hence, the angular distortion electronically disfavors SCO, as well as increasing the lattice activation energy for the transition.^63^ A previous study of two crystallographically characterized [Fe(bpp)_2_]^2+^ salts computed that a severe molecular distortion along pathway B lowers its thermodynamic SCO *T*_1/2_ by 13 K.^63^ While our study has focused on pathway A, our data are consistent with that report.

Where the comparison can be made, there is good agreement between computed and crystallographic Fe–N bond lengths in all of the complexes at large distortions, when ϕ ≤ 155° (Tables S7–S17). The less distorted minimized geometries deviate more strongly from experiment, with some computed Fe–N bonds being up to 0.4 Å longer than the crystallographic values. The Fe–N{pyridyl} bonds in those minimizations most often show the largest discrepancies with experiment; the Fe–N{pyrazolyl} distances are reproduced more accurately.

**Figure 8:**
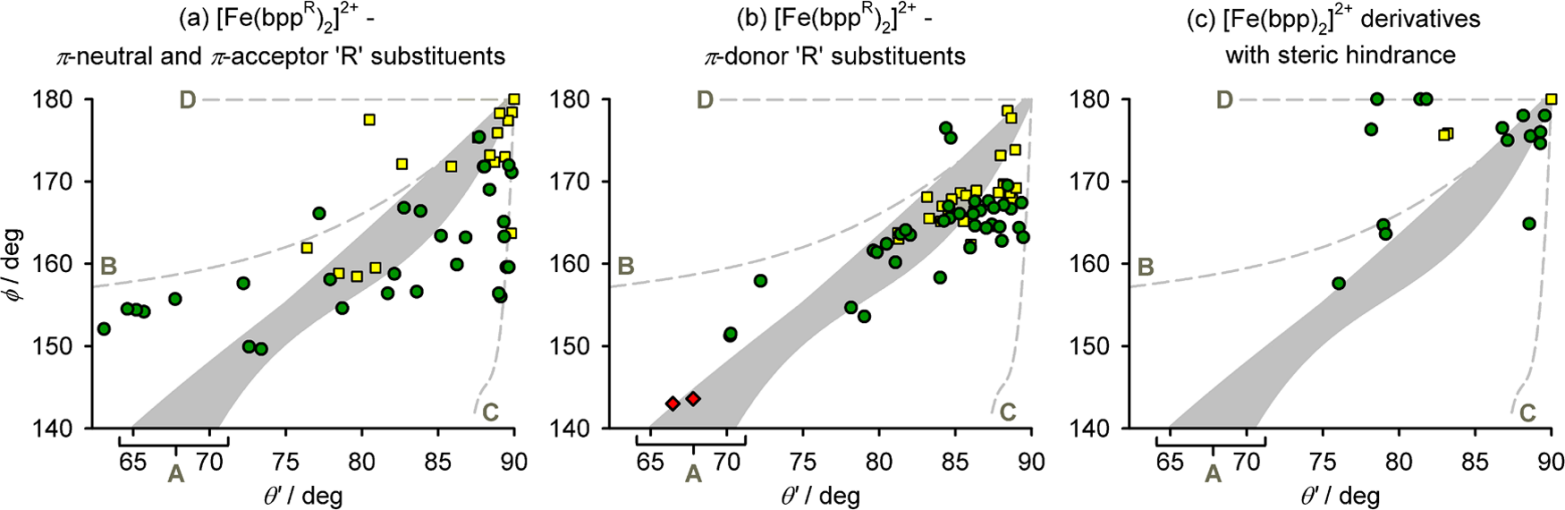
Comparison of the computed distortion pathways A–D (dashed lines) with structural data from mononuclear high-spin complexes, replotted on the ϕ vs θ′ scale (Table S4). Complexes with different patterns of ligand substituents are plotted separately, as shown (Chart 1). The shaded area shows the range of geometries for pathway A adopted by the molecules in Tables 2 and 3. Other details are as in Figure 1.

Figure 8 plots the computed distortion pathways against the experimental crystal structures of high-spin [Fe(bpp^R^)_2_]^2+^ salts, separated according to the ligand substituents present. Figure 1 includes a range of distortions for pathway A, computed for [Fe(bpp^R^)_2_]^2+^ derivatives with different R substituents (Table 3). [Fe(bpp^R^)_2_]^2+^ salts with π-neutral (R = H, alkyl, halogen) and π-acceptor R substituents show similar experimental trends and are plotted together in Figure 8a. The structure distribution for these compounds is scattered, but there are clear groups of structures close to pathways A–C (and one example near pathway D). Four of the six most distorted structures near pathway B are the isomorphous salts [Fe(bpp)_2_]X_2_ [X^–^ = PF_6_^–^,^31^ ClO_4_^–^,^102^ SbF_6_^–^,^102^ and CF_3_SO_3_^–^ (Table S3)]

Crystal structures of high-spin [Fe(bpp^R^)_2_]^2+^ derivatives with π-donor substituents are more numerous because such compounds are usually high-spin in solution under typical conditions for crystal growth [Figure 8b].^26^ Many of these structures lie within the ranges 170 ≥ ϕ ≥ 163° and 89 ≥ θ′ ≥ 83°, with a second smaller grouping at 164 ≥ ϕ ≥ 161° and 82 ≥ θ′ ≥ 80°. There is no such clustering of the structures in graph (a). That is consistent with the computed prediction that the minimum energy conformation lies at ϕ ≈ 165° for π-donor-substituted [Fe(bpp^R^)_2_]^2+^, but not for other substituent types (Figure 5). The crystal structures of **1a** and **1b** lie close to distortion pathway A. However, proportionately fewer structures in graph (b) have large angular distortions compared to graph (a), although these were computed to be more facile energetically for the π-donor-substituted complexes.

The sterically hindered molecules in Figure 8c have distal substituents at their pyrazolyl *C*3 positions, leading to steric crowding around the metal ion which should inhibit SCO. The only SCO-active complexes of this type have smaller methyl groups at this position. While the sample is small, most examples of distortion pathway D come from this group. Only D-type distortions with θ′ > 78° have been observed experimentally, which is consistent with the larger energy penalty associated with that distortion pathway (Figure 4).

## Conclusions

The isomorphous crystalline salts [Fe*L*_2_][BF_4_]_2_ (**1a**) and [Fe*L*_2_][ClO_4_]_2_ (**1b**) exhibit one of the strongest examples of the angular distortion from *D*_2d_ symmetry that is commonly found in high-spin [Fe(bpp)_2_]^2+^ derivatives.^22^ They are the most distorted mononuclear complexes observed to date by the ϕ and θ parameters used to quantify the distortion (Figure 1).^32^

In view of that result, the ϕ vs θ distortion landscape was surveyed with gas-phase DFT calculations on [Fe(bpp)_2_]^2+^, [Fe*L*_2_]^2+^, and other selected [Fe(bpp^R^)_2_]^2+^ derivatives (Chart 1). A modified θ parameter was employed in this analysis, θ′ (Chart 3), which gives better agreement between theory and experiment since it is less influenced by crystal packing effects.

Two minimum distortion pathways in ϕ and θ′ with comparable energies were identified for [Fe(bpp)_2_]^2+^ (Figure 4). Pathway A was preferred when ϕ was fixed during the minimizations, while pathway B was accessed in minimizations where θ′ was constrained by fixing interligand torsions. These differ in their ϕ parameters, which span a narrower range in pathway B than in pathway A (Table 2). Distortions in ϕ only (pathway C), and in θ′ only (pathway D), also minimized successfully. Compounds adopting each distortion type and geometries between those limiting pathways are known experimentally. Pathway D is rarer, however, and is mostly seen in sterically crowded complexes (Figure 8).

Discrepancies between the experimental and computed structure data in Figure 8 should reflect that the gas-phase calculations do not account for the influence of crystal packing on the molecular geometry. Nonetheless, the existence of pathways A and B is consistent with the distribution of highly distorted compounds in Figure 1. At smaller distortions, Δ*E*{*i*} approaches *kT* (*i* = ϕ or θ′; Figure 4) and becomes negative for pathway A when the molecules have electron-donating substituents (Table 3, Figure 5). Hence the distribution of experimental structures appears more random when ϕ > 160° or θ > 75°.

The energy penalty associated with pathways A–C is ≤ *kT* within the range of each distortion type that is commonly found experimentally (Figure 4). Hence, those pathways should be equally accessible to [Fe(bpp)_2_]^2+^ at room temperature, and the range of distortions observed for such compounds will reflect their different crystal packing requirements (Figure 1). Distortions along pathway D are higher in energy, and experimentally observed distortions of that type span a narrower range of θ′ values (Figure 8).

The energetics of the distortion significantly depend on the electronic character of the ligand pyridyl substituents in [Fe(bpp^R^)_2_]^2+^ (Chart 1). While electron-withdrawing nitro or cyano substituents have no computational effect, electron-donating dimethylamino or sulfanyl R groups lower the energy of the distortion. They also introduce a new minimum energy conformation at ϕ ≈ 165°, which is not shown by [Fe(bpp)_2_]^2+^ itself (Figure 5). The different behavior is attributed to the stronger Fe–N{pyridyl} σ-bonding when R is electron-donating, which is proportionately weakened more strongly as the distortion progresses (Figure 7).

The minimum at ϕ ≈ 165° for electron-donating substituents is no more than −0.4 kcal mol^–1^ below the undistorted geometry by our protocol. However, it is striking that, crystallographically, the ϕ ≈ 165° structure is adopted by most [Fe(bpp^R^)_2_]^2+^ derivatives with π-donor NR’_2_, OR’ or SR’ (R’ = H, alkyl or aryl) groups but not by their analogues with other substituent types (Figure 8). Among the molecules examined, [Fe*L*_2_]^2+^ and the other sulfanyl-substituted complexes give the lowest distortion energies across all values of ϕ.

Most salts of [Fe(bpp^R^)_2_]^2+^ derivatives with simple amino or alkoxy R substituents are high-spin, on electronic grounds.^26^ However, examples with R = *N*-acetamido^65,97^ or an alkylsulfanyl group^81−83,98,99^ consistently show cooperative thermal spin transitions at lower temperatures, which often involve unusual structural chemistry. That reflects their intrinsic preference for the ϕ ≈ 165° geometry in the high-spin state, which is observed crystallographically and *in silico* (Figures 4 and 7). That leads to a large structural rearrangement during SCO, as the switching molecules transform between their high-spin (ϕ ≈ 165°) and low-spin (ϕ ≈ 175°) states. The lattice energy profile associated with the molecular structure rearrangement in the solid state is the source of their cooperative behavior.

These results imply that if they are SCO-active, [Fe(bpp^R^)_2_]^2+^ derivatives bearing electron-donating R groups are the most likely to show cooperative spin transitions. That insight will guide our future research targets.

## Data Availability

Experimental data sets associated with this paper are available from the University of Leeds library (10.5518/1452).

## Supporting Information Available

Synthesis procedure and characterization data for the new ligand *L*; crystallographic data and refinement procedures; crystallographic figures and tables; X-ray powder diffraction data; solid-state and solution phase magnetic susceptibility data; and details of the minimized structures from the DFT calculations (PDF)
