# Supplementary material for: A Survey of the Angular Distortion Landscape in the Coordination Geometries of High-Spin Iron(II) 2,6-Bis(pyrazolyl)pyridine Complexes
Source: Inorg Chem. 2024 Jan 23;63(5):2732–44. doi: 10.1021/acs.inorgchem.3c04138 (PMC10848207; doi:10.1021/acs.inorgchem.3c04138)
Supplement: Supplementary file 1 — ic3c04138_si_001.pdf [file ic3c04138_si_001.pdf]

Supporting Information

A Survey of the Angular Distortion Landscape in the Coordination Geometries of High-Spin Iron(II) 2,6-Bis(pyrazolyl)pyridine Complexes

Izar Capel Berdiell,[a,b] Evridiki Michaels,[a] Orde Q. Munro*[a] and Malcolm A. Halcrow*[a]

[a]School of Chemistry, University of Leeds, Woodhouse Lane, Leeds, UK LS2 9JT.
E-mail: o.munro@leeds.ac.uk
m.a.halcrow@leeds.ac.uk

[b]Current address: Center for Material Science and Nanomaterials (SMN), University of Oslo, Sem Sælands 26, 0371 Oslo, Norway.

		Page
**Figure S1**	Plotted distortion parameters from $[Fe(bpp)_2]^{2+}$ derivatives, highlighting the conformationally rigid supramolecular assemblies.	S3
**Experimental – ligand synthesis.**		S4
**Scheme S1**	Synthesis of *L*.	S4
**Crystallographic refinement details**		S4
**Table S1**	Experimental data for the crystal structures in this work.	S5
**Figure S2**	NMR spectra of *L*.	S6
**Figure S3**	The asymmetric unit and packing diagram of *L*.	S7
**Definitions of the Structural Parameters in Tables S1 and S6-S14**		S8
**Chart S1**	Angles used in the definitions of the distortion parameters Σ and Θ.	S8
**Chart S2**	Definition of the Jahn-Teller distortion parameters θ and ϕ.	S8
**Chart S3**	The molecular planes used to calculate the modified θ parameter θ'.	S9
**Figure S4**	The formula units in the crystal structures of isostructural **1a** and **1b**.	S10
**Table S2**	Bond lengths, angles and other structural parameters for **1a** and **1b**.	S11
**Figure S5**	Packing diagram of **1b** viewed along the [010] vector.	S12
**Figure S6**	A cation layer in **1b**, showing the columns of $\pi\cdots\pi$ stacking interactions.	S13
**Figure S7**	Room temperature X-ray powder diffraction data **1a** and **1b**.	S14
**Figure S8**	Solid state magnetic susceptibility data for **1a** and **1b**.	S15
**Figure S9**	Variable temperature magnetic susceptibility data for **1b** in CD_3CN solution.	S15
**Figure S10**	The formula unit in the crystal structure of $[Fe(bpp)_2][CF_3SO_3]_2$.	S16
**Table S3**	Bond lengths, angles and other structural parameters for $[Fe(bpp)_2][CF_3SO_3]_2$.	S16
**Figure S11**	Comparison of the crystallographic and computed geometries of two $[Fe(bpp)_2]^{2+}$ derivatives with large angular distortions.	S17
**Table S4**	Distortion parameters for literature salts of high-spin $[Fe(bpp)_2]^{2+}$ derivatives.	S18
**Chart S4**	Ligand abbreviations used in Table S15.	S21
**Figure S12**	The minimized undistorted structures of the complexes computed in this study.	S22
**Figure S13**	Freely minimized structures of the high- and low-spin states of $[FeL_2]^{2+}$.	S24
**Table S5**	Metric parameters and minimized energies for the freely minimized high- and low-spin states of $[FeL_2]^{2+}$.	S24
**Figure S14**	Computed structures of $[Fe(bpp)_2]^{2+}$ along distortion pathway A.	S25
**Figure S15**	Computed structures of $[Fe(bpp)_2]^{2+}$ along distortion pathway B.	S26
**Figure S16**	Computed structures of $[Fe(bpp)_2]^{2+}$ along distortion pathway C.	S27
**Figure S17**	Computed structures of $[Fe(bpp)_2]^{2+}$ along distortion pathway D.	S28
**Figure S18**	Computed structures of $[Fe(bpp^{NO2})_2]^{2+}$ along distortion pathway A.	S29
**Figure S19**	Computed structures of $[Fe(bpp^{CN})_2]^{2+}$ along distortion pathway A.	S30
**Figure S20**	Computed structures of $[Fe(bpp^{NMe2})_2]^{2+}$ along distortion pathway A.	S31
**Figure S21**	Computed structures of $[Fe(bpp^{SMe})_2]^{2+}$ along distortion pathway A.	S32
**Figure S22**	Computed structures of $[Fe(bpp^{SPh})_2]^{2+}$ along distortion pathway A.	S33
**Figure S23**	Computed structures of $[FeL_2]^{2+}$ along distortion pathway A.	S34
**Table S6**	Computed energies of $[Fe(bpp)_2]^{2+}$ along distortion pathways A-D	S36
**Table S7**	Metric parameters for minimized $[Fe(bpp)_2]^{2+}$ along distortion pathway A.	S37
**Table S8**	Metric parameters for minimized $[Fe(bpp)_2]^{2+}$ along distortion pathway B.	S38
**Table S9**	Metric parameters for minimized $[Fe(bpp)_2]^{2+}$ along distortion pathway C.	S39

		Page
**Table S10**	Metric parameters for minimized $[Fe(bpp)_2]^{2+}$ along distortion pathway D.	S40
**Table S11**	Computed energies of the other high-spin complexes along distortion pathway A.	S41
**Table S12**	Metric parameters for minimized $[Fe(bpp^{NO2})_2]^{2+}$ along distortion pathway A.	S42
**Table S13**	Metric parameters for minimized $[Fe(bpp^{CN})_2]^{2+}$ along distortion pathway A.	S43
**Table S14**	Metric parameters for minimized $[Fe(bpp^{NMe2})_2]^{2+}$ along distortion pathway A.	S44
**Table S15**	Metric parameters for minimized $[Fe(bpp^{SMe})_2]^{2+}$ along distortion pathway A.	S45
**Table S16**	Metric parameters for minimized $[Fe(bpp^{SPh})_2]^{2+}$ along distortion pathway A.	S46
**Table S17**	Metric parameters for minimized $[Fe\mathit{L}_2]^{2+}$ along distortion pathway A.	S47
**Figure S24**	Molecular structures of high-spin $[Fe(bpp)_2]^{2+}$ showing the cartesian axis orientations in point group symmetries ranging from C_1 to D_{2d}.	S48
**Figure S25**	Energy levels of the top 9 occupied MOs of high-spin $[Fe(bpp)_2]^{2+}$ with $\phi = 140°$.	S49
**Table S18**	Atomic coordinates for freely minimized $[Fe\mathit{L}_2]^{2+}$ in its high- and low-spin states.	S50
**Table S19**	Atomic coordinates for $[Fe(bpp)_2]^{2+}$ along the different distortion pathways.	S54
**Table S20**	Atomic coordinates for the other $[Fe(bpp^R)_2]^{2+}$ molecules.	S80
**References**		S141

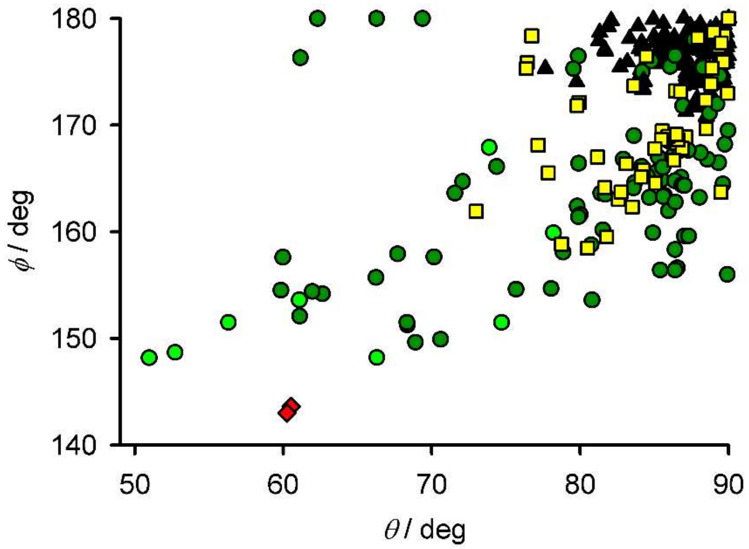

Figure S1 Plotted distortion parameters from **1a** and **1b** (red diamonds) and other $[Fe(bpp)_2]^{2+}$ derivatives, updated from ref. 1. Compounds that are low-spin (black triangles); high-spin and SCO-active (yellow squares) and high-spin and SCO-inactive (green circles) are plotted separately. Dark green circles are from mononuclear high-spin complexes, while pale green circles are helicate or metallacycle assemblies supported by conformationally rigid bpp derivatives.[2]

This is a repeat of Figure 1 in the main article, but which distinguishes the rigid supramolecular assemblies from the mononuclear complexes in the plot. Compounds **1a** and **1b** are the most distorted mononuclear complexes of this type yet reported.

Experimental

Synthesis of 4-(3,4-dimethoxyphenylsulfanyl)-2,6-di(pyrazol-1-yl)pyridine (*L*; Scheme S1). Sodium hydride 60% (60 wt % in mineral oil; 48 mg, 1.2 mmol) was suspended in tetrahydrofuran (thf; 20 cm^3) and 3,4-dimethoxybenzenethiol (200 mg, 1.2 mmol) was then added. The mixture was stirred at room temperature for two mins, then 2,4,6-trifluoropyridine (155 mg, 1.2 mmol) was added. The mixture was further stirred for 3 hrs. Pyrazole (170 mg, 2.5 mmol) was then added, followed by another portion of NaH (60 wt % in mineral oil; 100 mg, 2.5 mmol). The reaction was complete by thin layer chromatography after an additional 2 hrs stirring at room temperature. The solvent was removed *in vacuo*, and the residue was purified by flash silica chromatography (eluent: 4:1 hexane:ethyl acetate). The target product *L* (Rf 0.37) was an off-white solid. Yield 134 mg, 30 %. Mp 96-97 °C. Found C, 60.1; H, 4.49; N, 18.3 %. Calcd for $C_{19}H_{17}N_5O_2S$ C, 60.1; H, 4.52; N, 18.5 %. ESMS *m/z* 380.1743 (87 %; calcd for $[HL]^+$ 380.1776), 402.1002 (100 %; calcd for $[NaL]^+$ 402.0995). ^1H NMR ($CDCl_3$) δ 3.82, 3.88 (both s, 3H, OCH_3), 6.38 (dd, 1.7 and 2.5 Hz, 2H, Pz H^4), 6.90 (d, 8.3 Hz, 1H, Ph H^6), 7.03 (d, 2.1 Hz, 1H, Ph H^2), 7.16 (dd, 2.1 and 8.3 Hz, 1H, Ph H^5), 7.46 (s, 2H, Py $H^{3/5}$), 7.62 (d, 1.7 Hz, 2H, Pz H^3), 8.43 (d, 2.5 Hz, 2H, Pz H^5) ppm. ^{13}C NMR ($CDCl_3$) δ 56.0, 56.1 (both 1C, OCH_3) 105.6 (2C, Py $C^{3/5}$), 107.9 (2C, Pz C^4), 112.2 (1C, Ph C^2), 118.0 (1C, Ph C^5), 119.1 (1C, Ph C^1), 127.2 (2C, Pz C^5), 129.0 (1C, Ph C^6), 142.3 (2C, Pz C^3), 149.9, 150.0 (both 1C, Ph C^3 and C^4), 150.8 (2C, Py $C^{2/6}$), 157.4 (1C, Py C^4) ppm.

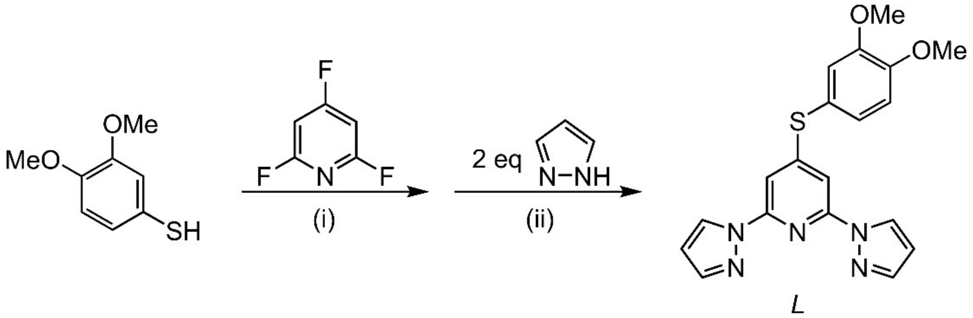

Scheme S1 Synthesis of *L*. Reagents and conditions: (i) NaH, thf, 298 k, 3 hrs. (ii) NaH (2 eq.), 298 K, 2 hrs. Purified yield 30 %.

Crystallographic refinement details
The structures were solved by direct methods *(SHELX-TL*[3]), and developed by full least-squares refinement on F^2 (*SHELXL2018*[4]). Crystallographic figures were produced using *XSEED*,[5] and other publication materials were prepared with *OLEX*2.[6] All non-H atoms in each structure were refined anisotropically while H atoms were placed in calculated positions and refined using a riding model.

Refinements of *L*. The asymmetric unit contains one molecule of the compound, on a general crystallographic site. No disorder is present in the model, and no restraints were applied to the refinement.

Refinements of $[FeL_2][BF_4]_2$ (1a), $[FeL_2][ClO_4]_2$ (1b) and $[Fe(bpp)_2][CF_3SO_3]_2$. The asymmetric unit of all these crystals contains half a formula unit of the compound, with Fe(1) lying on a crystallographic C_2 axis. No disorder is present in either model, and no restraints were applied to the refinements.

The dataset of **1a** was refined as a two-component twin. Two non-superimposable twin domains were resolved in the hkl file, with a population ratio of 0.73:0.27.

The maximum residual Fourier peak of +1.2 e Å^{-3} in **1b** is 1.0 Å from Fe(1).

Table S1. Experimental details for the single crystal structure determinations.

	L	[Fe*L*$_2$][BF$_4$]$_2$ (**1a**)[a]	[Fe*L*$_2$][ClO$_4$]$_2$ (**1b**)	[Fe(bpp)$_2$][CF$_3$SO$_3$]$_2$
formula	$C_{19}H_{17}N_5O_2S$	$C_{38}H_{34}B_2F_8FeN_{10}O_4S_2$	$C_{38}H_{34}Cl_2FeN_{10}O_{12}S_2$	$C_{24}H_{18}F_6FeN_{10}O_6S_2$
M_r	379.43	988.34	1013.62	776.45
crystal system	monoclinic	monoclinic	monoclinic	monoclinic
space group	*Cc*	*C*2/*c*	*C*2/*c*	*C*2/*c*
a / Å	25.2282(14)	16.0676(7)	16.1718(4)	14.3422(2)
b / Å	4.5977(3)	7.9446(3)	7.9654(3)	9.2026(1)
c / Å	15.1996(10)	32.1708(11)	32.0532(10)	21.9396(3)
α / deg	90	90	90	90
β / deg	92.079(4)	99.157(3)	98.560(3)	96.508(1)
γ / deg	90	90	90	90
V / Å^3	1761.87(19)	4054.3(3)	4082.9(2)	2877.05(6)
Z	4	4	4	4
T / K	120	120	120	120
$\mu\{Cu\text{-}K_\alpha\}$ / mm^{-1}	1.853	4.774	5.803	6.486
D_{calc} / g cm^{-3}	1.430	1.619	1.649	1.793
reflections collected	6311	4529	7835	5498
unique reflections	2999	4529[a]	4014	2768
R_{int}	0.026	–[a]	0.033	0.016
R_1 [$I > 2\sigma(I)$][b]	0.035	0.072	0.061	0.026
wR_2 [all data][c]	0.098	0.179	0.158	0.065
GOF	1.093	1.189	1.190	1.048
$\Delta\rho_{max}$, $\Delta\rho_{min}$ / e Å^{-3}	+0.34, −0.52	+0.84, −0.53	+1.25, −0.62	+0.36, −0.29
Flack parameter	0.027(18)	–	–	–
CCDC	2260605	2260606	2260607	2260608

[a]This dataset contains two non-superimposable twin domains, which were resolved in the *hkl* file. [b]$R = \Sigma [|F_o| - |F_c|] / \Sigma|F_o|$ [c]$wR = [\Sigma w(F_o^2 - F_c^2)^2 / \Sigma wF_o^4]^{1/2}$

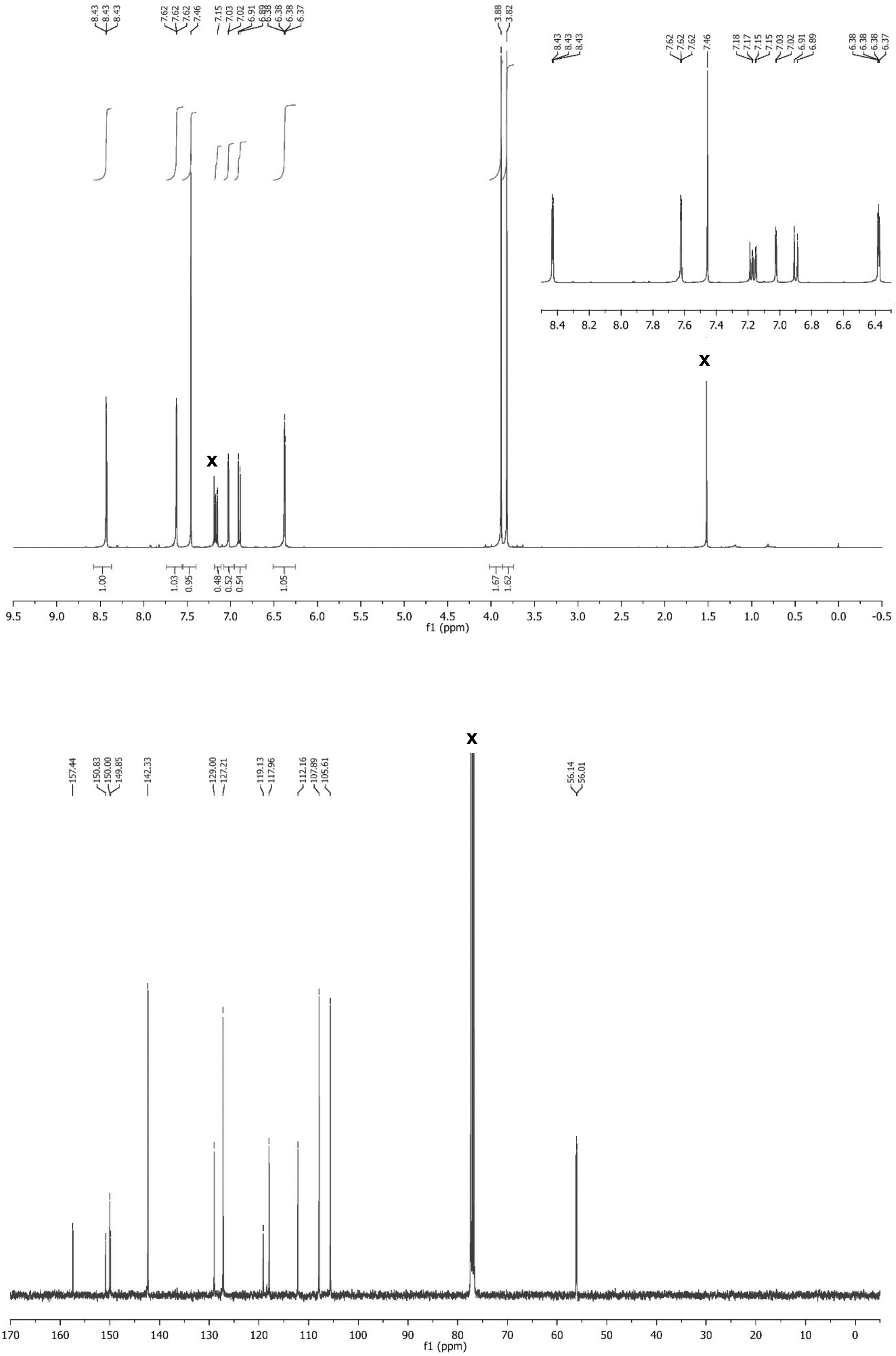

Figure S2 ^{1}H (top) and ^{13}C (bottom) NMR spectra of *L* ($CDCl_3$).

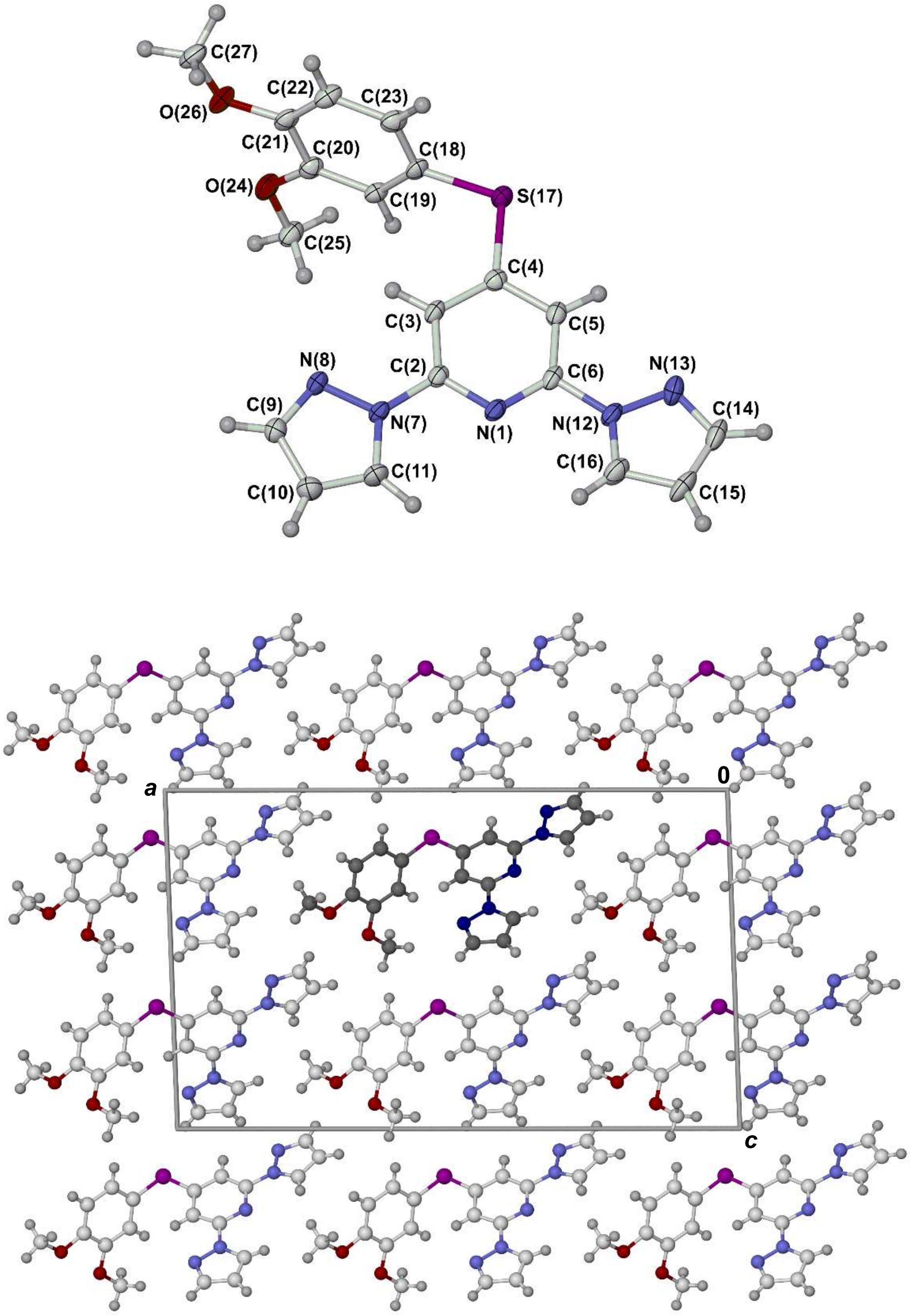

Figure S3 Top. View of the asymmetric unit of *L*. Displacement ellipsoids are at the 50 % probability level except for H atoms which have arbitrary radii. Bottom: packing diagram of *L* viewed along the [010] vector. One molecule in the packing diagram is highlighted with dark coloration.

Color code: C, white or dark gray; H, pale gray; N, pale or dark blue; O, red; S, purple.

The molecules associate into stacks by translation perpendicular to the view, *via* two unique face-to-face $\pi\cdots\pi$ contacts between molecules related by x, $1+y$, z. First is between the pyrazolylpyridine residues N(1)-C(11) on each molecule [interplanar distance 3.424(17) Å], and second is between their phenyl substituents [3.58(2) Å].

Definitions of the Structural Parameters in Tables S1 and S6-S14

V_{Oh} is the volume (in Å^3) of the FeN_6 coordination octahedron in the complex,[7] which is typically <10 Å^3 in low-spin $[FeL_2]^{2+}$ derivatives related to the compounds in this work, and ≥11 Å^3 in their high-spin form. Highly distorted high-spin complexes show V_{Oh} at the low end of the typical high-spin range.[8]

Σ and Θ are defined as follows:

$$\Sigma = \sum_{i=1}^{12} |90 - \beta_i| \qquad \Theta = \sum_{j=1}^{24} |60 - \gamma_j|$$

where β_i are the twelve *cis*-N–Fe–N angles about the iron atom and γ_i are the 24 unique N–Fe–N angles measured on the projection of two triangular faces of the octahedron along their common pseudo-threefold axis (Chart S1). A perfectly octahedral complex gives $\Sigma = \Theta = 0$.[7,9] Because the high-spin state of a complex has a much more plastic structure than the low-spin, this is reflected in Σ and Θ which are often much larger in the high-spin state.[10]

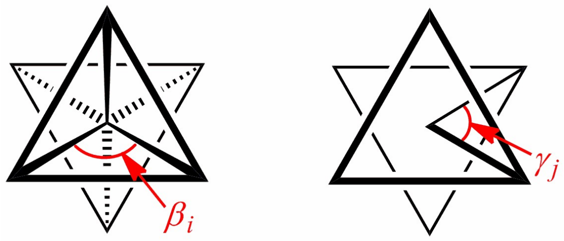

Chart S1. Angles used in the definitions of the coordination distortion parameters Σ and Θ.

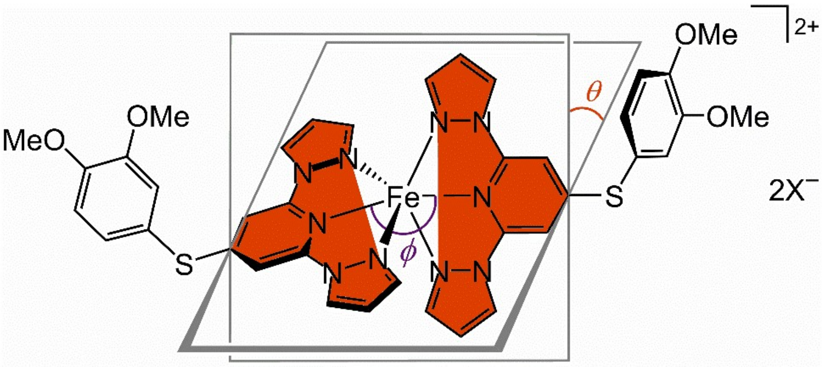

Chart S2. The Jahn-Teller distortion parameters θ and ϕ in $[Fe\mathit{L}_2]X_2$ (X^- = anion), adapted from Chart 2 in the main article. The planes of the two ligands used to measure the dihedral angle θ are shaded in red.

These two parameters define the magnitude of an angular Jahn-Teller distortion, that is often observed in high-spin $[Fe(bpp)_2]^{2+}$ (bpp = 2,6-di{pyrazol-1-yl}pyridine) derivatives like $[Fe\mathit{L}_2]^{2+}$ ($\theta \leq 90^o$, $\phi \leq 180^o$).[1,11] They are also a useful indicator of the molecular geometry, in defining the disposition of the two ligands around the metal ion.

Large changes in ϕ, particularly, between the spin states can lead to highly cooperative spin-transitions.[12-15] However, spin-crossover can be inhibited if θ and ϕ deviate more strongly from their ideal values in the high-spin state,[1,16] because the associated rearrangement to a more regular low-spin coordination geometry ($\theta \approx 90°$, $\phi \approx 180°$) cannot be accommodated by a rigid solid lattice.[11,17]

The computational part of this study considers the modified θ parameter θ', which is the dihedral angle between the planes formed by the three N-donor atoms on each bpp^R ligand (Chart S3). θ values for distorted $[Fe(bpp^R)_2]^{2+}$ complexes are influenced by the bowl-shaped or S-shaped ligand conformations that are often found in such complexes (Figure S11). These ligand conformations are influenced by crystallographic packing effects, which cannot be described by gas phase calculations.

The θ' parameter ignores the peripheral ligand atoms, thus removing that complication. While computed θ and θ' values are usually quite similar, crystallographically those parameters can differ by up to 15° for a given compound (Table S4). Hence, experimental θ' values give better agreement with the computational results.

An undistorted complex gives $\theta = \theta' = 90°$.

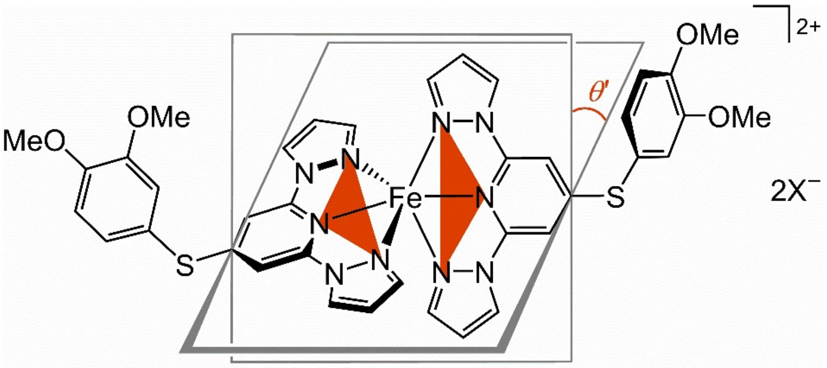

Chart S3. The molecular planes used to calculate the modified θ parameter θ'. θ' is the dihedral angle between the planes shown, which are defined by the three N-donor atoms of each ligand in the molecule.

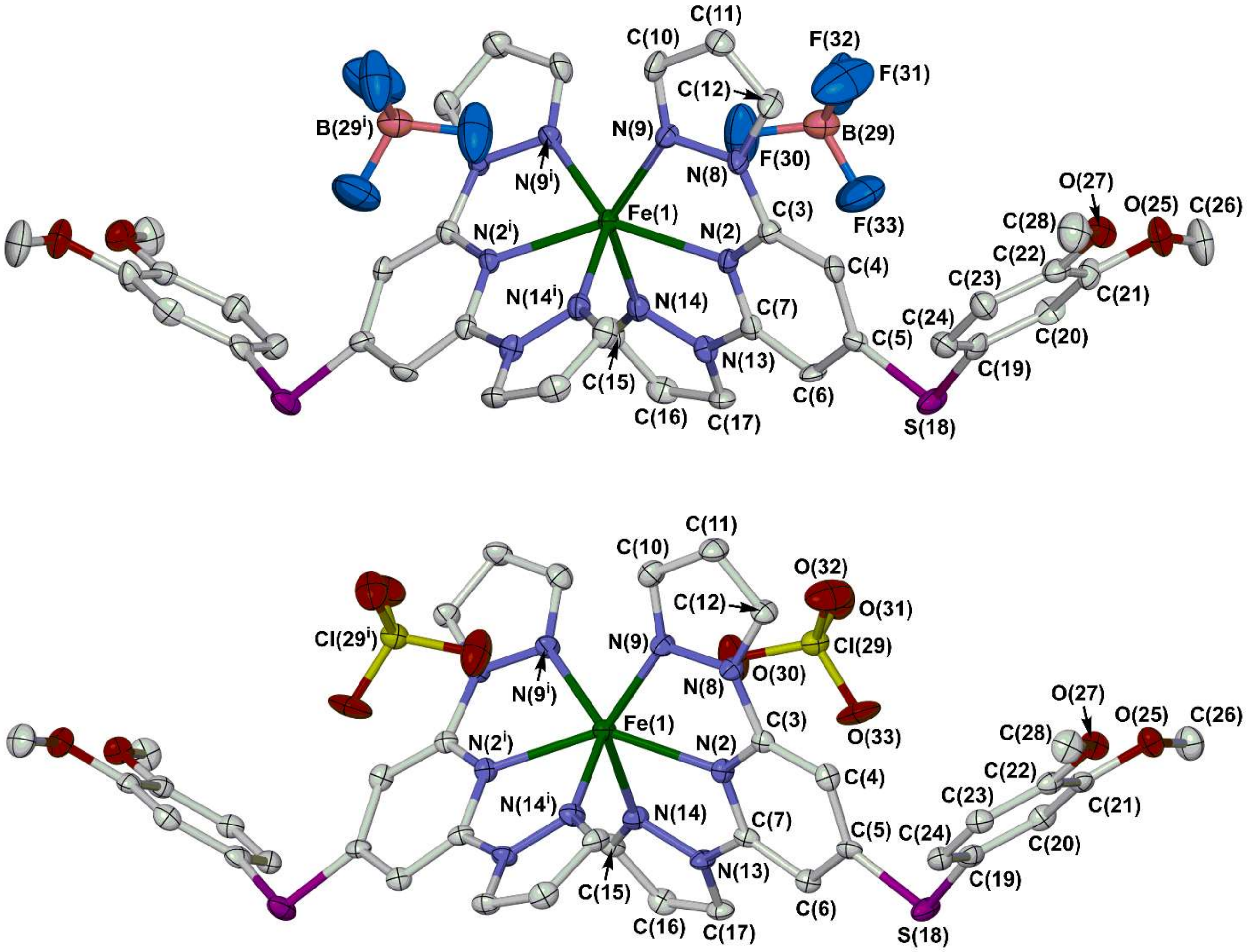

Figure S4 View of the formula units in the crystal structures of isostructural $[FeL_2][BF_4]_2$ (**1a**, top) and $[FeL_2][ClO_4]_2$ (**1b**, bottom), showing the complete atom numbering scheme. Displacement ellipsoids are at the 50 % probability level, and H atoms are omitted for clarity. Symmetry code: (i) 1–*x*, *y*, $^{3}/_{2}$–*z*.

Color code: C, white; B, pink; Cl, yellow; F, cyan; Fe, green; N, blue; O, red; S, purple.

Table S2 Selected bond lengths (Å), angles (°) and other structural parameters for $[FeL_2][BF_4]_2$ (**1a**) and $[FeL_2][ClO_4]_2$ (**1b**). Conformational data from the free ligand crystal structure are also included for comparison. See Figures S3 and S4 for the atom numbering schemes, and page S8 for definitions of the structural parameters listed. Symmetry code: (i) 1–*x*, *y*, $^3/_2$–*z*.

	**1a**	**1b**		*L*
Fe(1)–N(2)	2.163(5)	2.171(4)		
Fe(1)–N(9)	2.169(5)	2.180(4)		
Fe(1)–N(14)	2.218(6)	2.215(4)		
N(2)–Fe(1)–N(2^i)	143.6(3)	143.0(2)		
N(2)–Fe(1)–N(9)	72.3(2)	71.67(15)		
N(2)–Fe(1)–N(9^i)	136.0(2)	137.12(15)		
N(2)–Fe(1)–N(14)	70.98(19)	71.33(15)		
N(2)–Fe(1)–N(14^i)	85.0(2)	84.07(15)		
N(9)–Fe(1)–N(9^i)	96.9(3)	97.6(2)		
N(9)–Fe(1)–N(14)	139.04(19)	138.81(15)		
N(9)–Fe(1)–N(14^i)	97.0(2)	97.21(15)		
N(14)–Fe(1)–N(14^i)	97.3(3)	96.4(2)		
C(5)–S(18)–C(19)	104.9(3)	105.8(2)	C(4)–S(17)–C(18)	104.37(14)
C(4)–C(5)–S(18)–C(19)	3.6(7)	3.2(5)	C(3)–C(4)–S(17)–C(18)	0.2(3)
C(6)–C(5)–S(18)–C(19)	178.2(5)	177.9(4)	C(5)–C(4)–S(17)–C(18)	179.3(2)
β^a	73.8(2)	77.10(14)	β^a	75.22(8)
V_{Oh} / Å^3	11.02(2)	10.991(16)		
Σ / deg	203.6(7)	208.5(5)		
Θ / deg	500	506		
ϕ / deg	143.6(3)	143.0(2)		
θ / deg	60.53(6)	60.25(5)		
θ' / deg	67.79(19)	66.45(15)		

$^a\beta$ is the dihedral angle between the least squares planes of the ligand pyridyl ring and the phenyl substituent.

In addition to the Fe–N bonds in the Table, the anions form long contacts to the open face of Fe(1). The distances are Fe(1)···F(30) = 3.278(6) Å, and Fe(1)···O(30) = 3.212(4) Å. These are too long to be considered coordinative interactions, and are better described as weak electrostatic contacts. Similar Fe···anion contacts are also shown by other salts of $[Fe(bpp)_2]^{2+}$ derivatives with large angular geometric distortions.[16]

The phenylsulfanyl substituent conformations in the complex and the free ligand structures are essentially the same. The C–S–C group lies in the plane of the pyridyl ring [C–C–S–C torsions ≈ 0 or 180°], and the phenyl group and pyridyl ring are not quite perpendicular to each other [73.8(2) ≤ β ≤ 77.10(14)°].

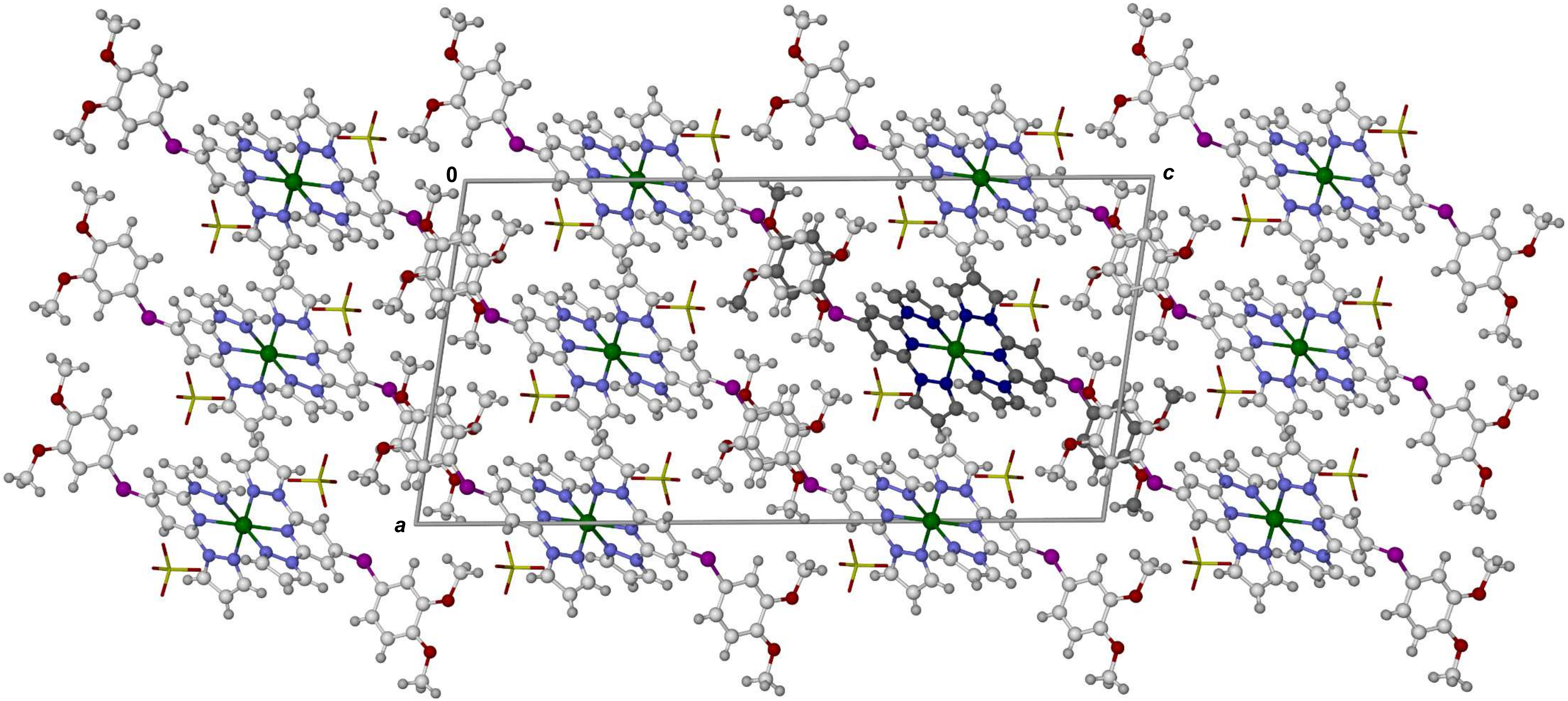

Figure S5 Packing diagram of **1b** viewed along the [010] vector. One molecule in the packing diagram is highlighted with dark coloration, and the ClO_4^- anions are de-emphasized for clarity.

Color code: C, white or dark gray; H, pale gray; Cl, yellow; Fe, green; N, pale or dark blue; O, red; S, purple.

The complex molecules associate into layers parallel to the (101) plane, *via* columns of face-to-face $\pi\cdots\pi$ stacking interactions between their phenyl rings which propagate along b (Figure S6).
The same mode of crystal packing is adopted by **1a**, which is isomorphous with **1b**.

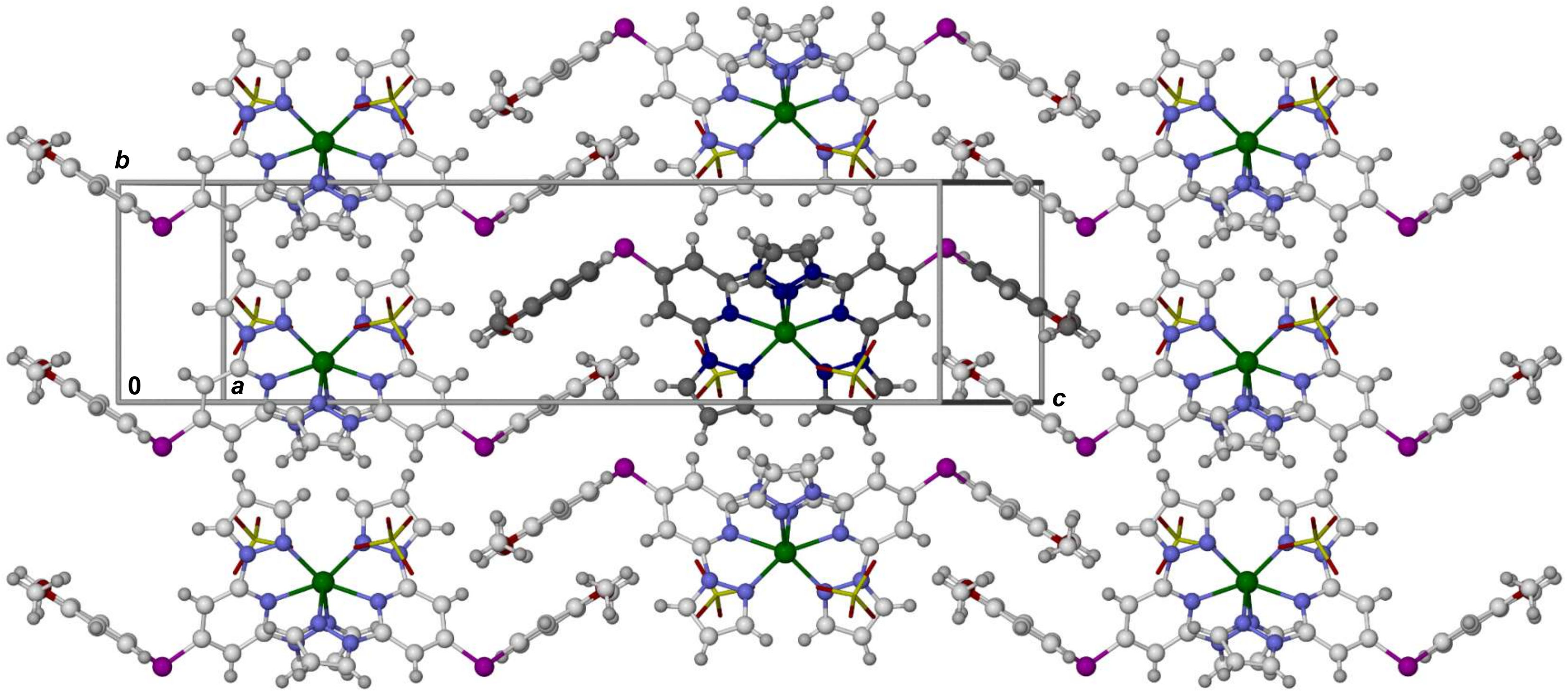

Figure S6 A cation layer in the crystal lattice of **1b**, showing the columns of $\pi\cdots\pi$ stacking interactions parallel to the unit cell *b* direction. Details as for Figure S5.

Color code: C, white or dark gray; H, pale gray; Cl, yellow; Fe, green; N, pale or dark blue; O, red; S, purple.

The stacked phenyl groups are related by crystallographic inversion centres alternating along *b* [symmetry codes $^1/_2$–*x*, $^1/_2$–*y*, 1–*z* and $^1/_2$–*x*, $^3/_2$–*y*, 1–*z*]. Alternate pairs of stacked rings are separated by 3.30(4) Å and 3.50(5) Å for **1a**, and by 3.29(4) Å and 3.46(5) Å for **1b**.

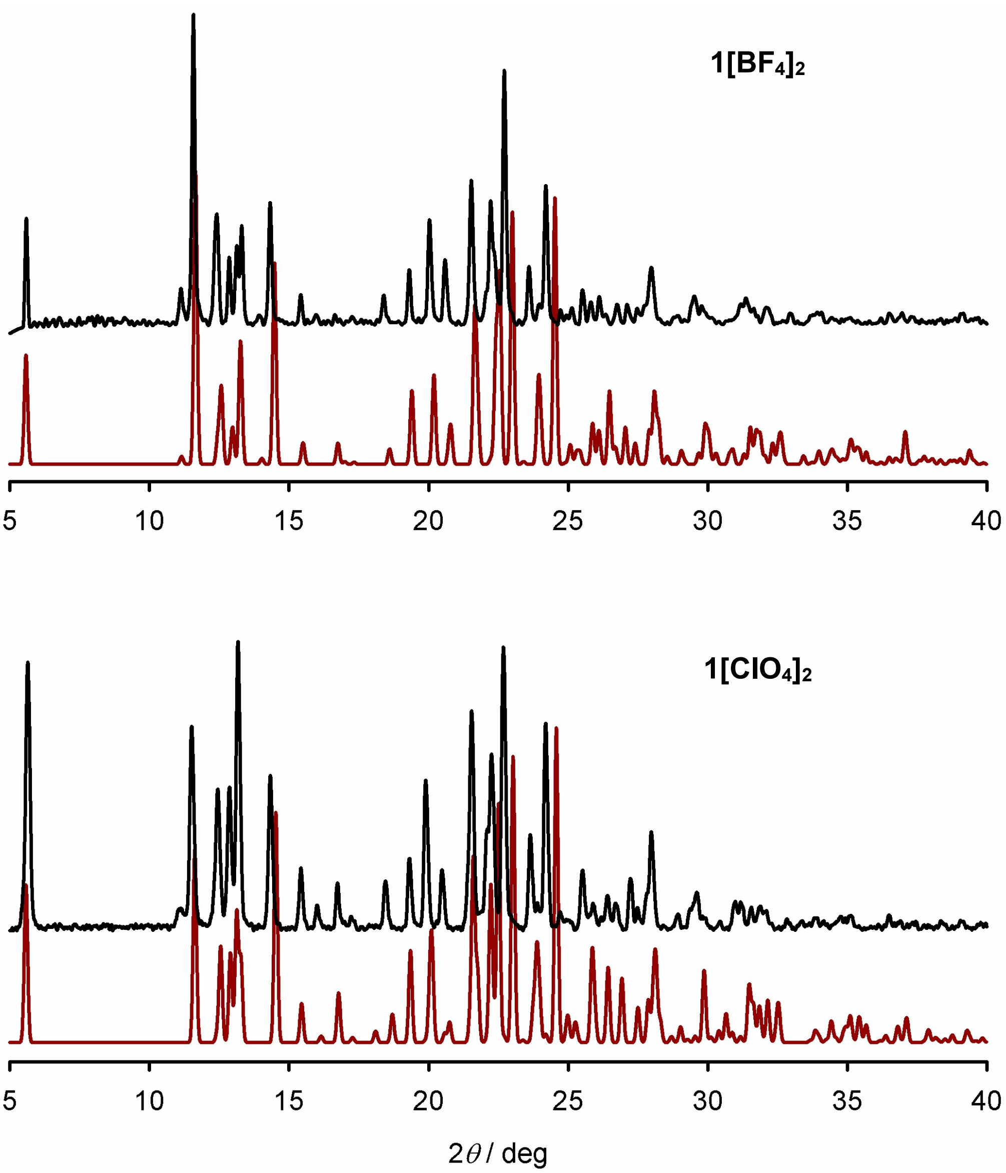

Figure S7 Measured room temperature X-ray powder diffraction data **1a** and **1b** (black), and simulations based on their single crystal structures at 120 K (red).

There is good correspondence between the experimental and simulated data, with minor differences in some peak positions that can be attributed to the different temperatures of the measurement and simulation.

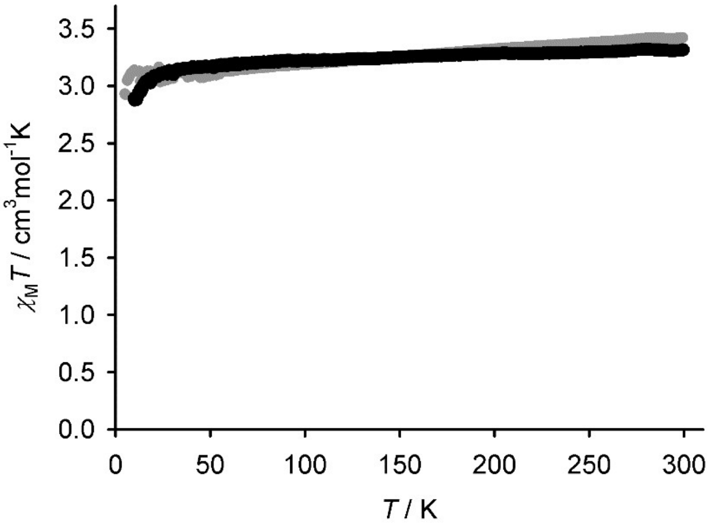

Figure S8 Solid state magnetic susceptibility data for $[FeL_2][BF_4]_2$ (**1a**, black) and $[FeL_2][ClO_4]_2$ (**1b**, gray), measured at a scan rate of 5 K min^{-1}.

The complexes are high-spin, as expected, with small zero-field splittings. That is consistent with other $[Fe(bpp)_2]^{2+}$ complex salts with large angular Jahn-Teller distortions.[18]

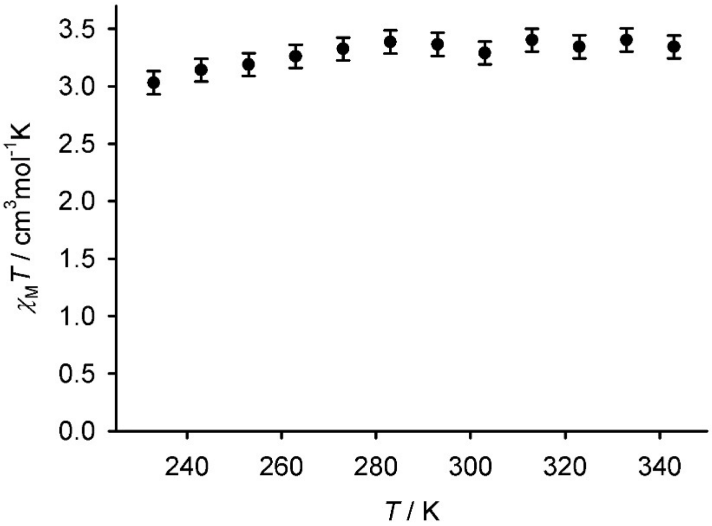

Figure S9 Variable temperature magnetic susceptibility data for $[FeL_2][ClO_4]_2$ (**1b**) in CD_3CN solution.

Our published correlation for pyridyl-substituted $[Fe(bpp)_2]^{2+}$ derivatives[19] predicts $T_{½}$ = 188 K for $[FeL_2]^{2+}$ bearing SAr (Ar = aryl) pyridyl substituents with a σ_P^+ Hammett parameter of −0.55.[20] These data may show the onset of an SCO equilibrium below 230 K which would be consistent with that, but if so it is barely outside experimental error.

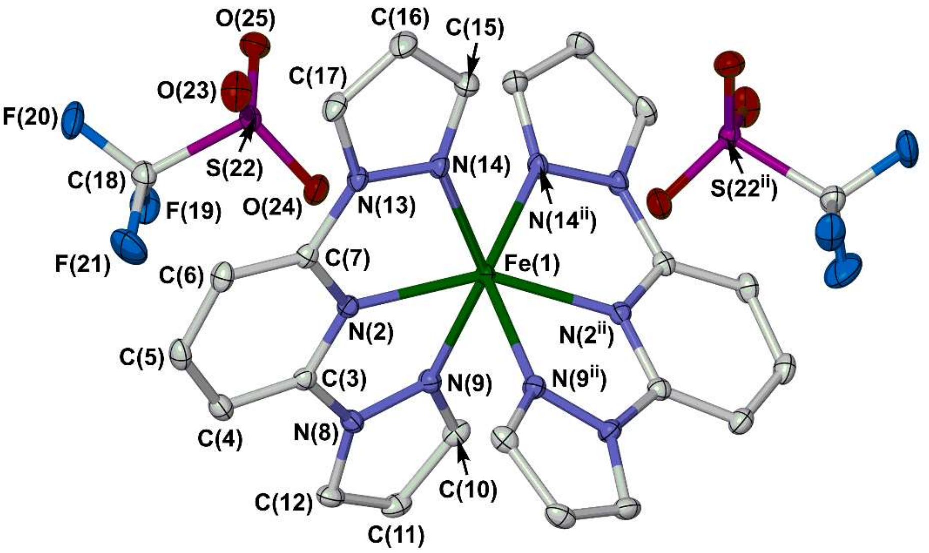

Figure S10 View of the formula unit in the crystal structure of $[Fe(bpp)_2][CF_3SO_3]_2$, showing the complete atom numbering scheme. Displacement ellipsoids are at the 50 % probability level, and H atoms are omitted for clarity. Symmetry code: (ii) $1-x, y, {}^1\!/_2-z$.

Color code: C, white; F, cyan; Fe, green; N, blue; O, red; S, purple.

This high-spin crystal is isomorphous with previously published $[Fe(bpp)_2][ClO_4]_2$, $[Fe(bpp)_2][PF_6]_2$ and $[Fe(bpp)_2][SbF_6]_2$.[11,18]

Table S3 Selected bond lengths (Å), angles (°) and other structural parameters for $[Fe(bpp)_2][CF_3SO_3]_2$. See Figure S10 for the atom numbering scheme, and page S8 for definitions of the structural parameters listed. Symmetry code: (ii) $1-x, y, {}^1\!/_2-z$.

Fe(1)–N(2)	2.1852(12)	Fe(1)–N(14)	2.1865(13)
Fe(1)–N(9)	2.2079(13)		
N(2)–Fe(1)–N(2^{ii})	152.08(7)	N(9)–Fe(1)–N(9^{ii})	85.40(7)
N(2)–Fe(1)–N(9)	71.59(5)	N(9)–Fe(1)–N(14)	140.12(5)
N(2)–Fe(1)–N(9^{ii})	87.78(5)	N(9)–Fe(1)–N(14^{ii})	107.94(5)
N(2)–Fe(1)–N(14)	71.60(5)	N(14)–Fe(1)–N(14^{ii})	85.96(7)
N(2)–Fe(1)–N(14^{ii})	131.96(5)		
V_{Oh} / Å^3	11.207(5)	ϕ / deg	152.08(7)
Σ / deg	206.50(17)	θ / deg	61.10(1)
Θ / deg	458	θ' / deg	63.07(5)

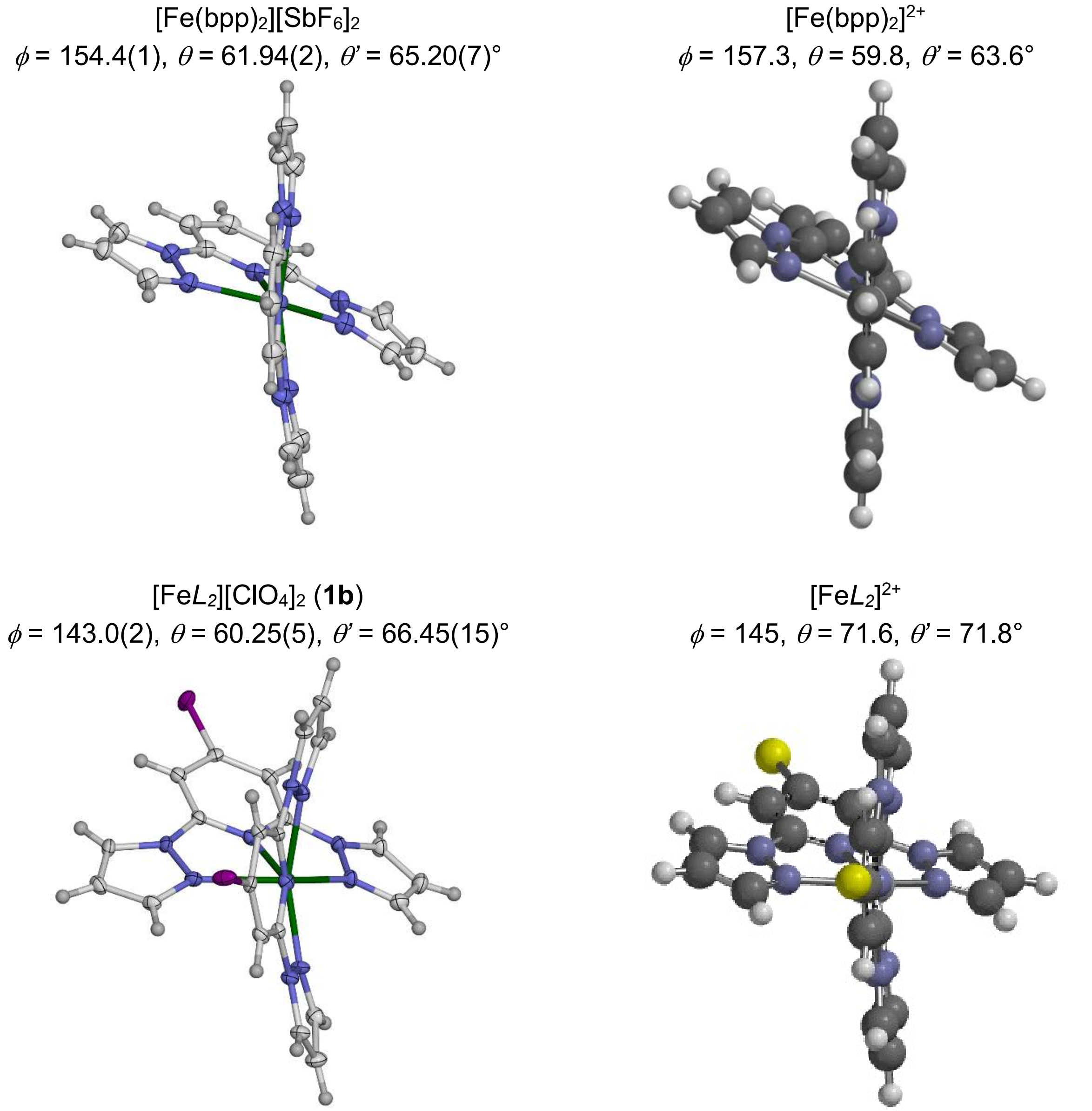

Figure S11 Comparison of the crystallographic (left) and computed (right) geometries of two [Fe(bpp)$_2$]$^{2+}$ derivatives with large angular distortions, illustrating how non-planar ligand conformations influence θ. Each view is oriented down an Fe–N{pyridyl} bond, and the dimethoxyphenyl substituents in **1b** and [FeL_2]$^{2+}$ are omitted for clarity.

Color code (crystallographic): C, white; H, pale gray; Fe, green; N, blue; S, purple.
Color code (DFT): C, dark gray; H, white; Fe, pale gray; N, blue; S, yellow.

[Fe(bpp)$_2$][SbF$_6$]$_2$ is described in ref. 18. The corresponding minimization of [Fe(bpp)$_2$]$^{2+}$ is taken from the computed distortion pathway B (Figure S14, Table S7).

The computed ligand conformations are often more planar than observed crystallographically in highly distorted complexes. The modified parameter θ' (Chart S3, page S9) is a better representation of the metal coordination geometry in these cases, and gives a better match between theory and experiment.

Table S4 Distortion parameters for literature salts of $[Fe(bpp)_2]^{2+}$ derivatives in their high-spin state. The ligand abbreviations are shown in Chart S4, at the foot of the Table, while ϕ, θ and θ' are defined on page S8. Compounds with no literature citation are from structures published before 2011, and which are not cited elsewhere in this document. These are tabulated and cited in ref. 10.

	Spin-state behavior	ϕ / deg	θ / deg	θ' / deg	Ref.
$[Fe(bpp)_2][ClO_4]_2$	High-spin	155.7(1)	66.24(2)	67.74(6)	18
$[Fe(bpp)_2][PF_6]_2$	High-spin	154.18(7)	62.64(1)	65.69(5)	11
$[Fe(bpp)_2][SbF_6]_2$	High-spin	154.4(1)	61.94(2)	65.20(7)	18
$[Fe(bpp)_2][I_3]_2$[a]	High-spin	156.4(4)-171.1(5)	84.9(1)-88.7(1)	86.2(4)-89.8(3)	
$[Fe(bpp)_2]I_{0.5}[I_3]_{1.5}$	High-spin	156.0(2)	89.92(5)	89.1(2)	21
$[Fe(bpp)_2][Co(C_2B_9H_{11})_2]_2\cdot CH_3NO_2$, molecule A[b]	High-spin	159.6(3)	87.03(9)	89.5(4)	21
$[Fe(bpp^{Me\text{-}3',3''})_2][PF_6]_2$	High-spin	178.0(1)	84.87(5)	88.2(1)	22
$[Fe(bpp^{Me\text{-}3',3''})_2][SbF_6]_2$	High-spin	178.0(1)	87.66(4)	89.6(1)	
$[Fe(bpp^{iPr\text{-}3',3''})_2][PF_6]_2\cdot CH_3CN\cdot 0.5(C_2H_5)_2$[c]	High-spin	175.0(1)-176.5(2)	84.19(5)-86.40(5)	87.1(1)-89.3(2)	
$[Fe(bpp^{CO_2Et\text{-}3',3''})_2][BF_4]_2$	High-spin	174.62(7)	89.39(2)	89.28(6)	
$[Fe(bpp^{CO_2Et\text{-}3',3''\text{-}Me_2\text{-}5',5''})_2][ClO_4]_2$	High-spin	157.61(11)	59.97(3)	76.05(9)	23
α-$[Fe(bpp^{Ph\text{-}3',3''})_2][BF_4]_2$	High-spin	176.3(2)	61.15(4)	78.2(1)	
β-$[Fe(bpp^{Ph\text{-}3',3''})_2][BF_4]_2$	High-spin	180	62.30(2)	78.57(4)	
$[Fe(bpp^{ferrocenyl\text{-}3',3''})_2][BF_4]_2\cdot 3CH_3CN$[d]	High-spin	180, 180	66.7, 69.4	81.4, 81.8	
$[Fe(bpp^{CH_2OH\text{-}3',3''})_2][BF_4]_2$	High-spin	164.7(1)	72.08(3)	78.98(7)	
α-$[Fe(bpp^{CH_2OH\text{-}3',3''})_2][ClO_4]_2$	High-spin	164.86(8)	85.48(2)	88.55(7)	
β-$[Fe(bpp^{CH_2OH\text{-}3',3''})_2][ClO_4]_2$	High-spin	163.62(7)	71.56(2)	79.13(4)	
$[Fe(bpp^{C_{12}H_{25}\text{-}4'})_2][BF_4]_2$	High-spin	171.8(1), 171.8(1)	86.95(3), 86.91(3)	88.0(1), 88.0(1)	24
$[Fe(bpp^{CH_2OH})_2][PMo_{12}O_{40}]_2$[e]	High-spin	156.4(3)	85.4(1)	81.7(4)	25
α-$[Fe(bpp^{CH_2SCN})_2][BF_4]_2$	High-spin	158.10(7)	78.8	77.9	
$[Fe(bpp^{E\text{-}CH=CHferrocenyl})_2][BF_4]_2\cdot 2(C_2H_5)_2O$	High-spin	175.4(2)	88.3	87.7	
$[Fe(bpp^{E\text{-}CH=CHferrocenyl})_2][I_3]_2$	High-spin	163.4(3)	85.7	85.2	
$[Fe(bpp^{E\text{-}CH=CHferrocenyl})_2][BPh_4]_2\cdot 3CH_3NO_2\cdot(C_2H_5)_2O$	High-spin	156.6(1)	86.6	83.6	
$[Fe(bpp^{E\text{-}CH=CHferrocenyl})_2][Ni(mnt)_2]_2$	High-spin	166.1(2)	74.4	77.2	
$[Fe(bpp^{Z\text{-}CH=CHPh})_2][BF_4]_2$	High-spin	154.6	75.7	78.7	26
$[Fe(bpp^{E\text{-}CH=CHPh})_2][BF_4]_2\cdot 3CH_3CN$	High-spin	166.8	82.9	82.8	26
$[Fe(bpp^{Z\text{-}CH=CHC_6H_4CN})_2][BF_4]_2$	High-spin	149.9	70.6	72.6	27
$[Fe(bpp^{E\text{-}CH=CHC_6H_4NO_2})_2][BF_4]_2\cdot H_2O$	High-spin	169.0	83.6	88.4	27
$[Fe(bpp^{pyren\text{-}1\text{-}yl})_2][ClO_4]_2$	High-spin	149.58(19)	66.9	73.4	28
$[Fe(bpp^{C\equiv CPh})_2][BF_4]_2\cdot CH_3NO_2$	High-spin	157.63(4)	70.16(2)	72.22(5)	

Table S4 continued.

	Spin-state behavior	ϕ / deg	θ / deg	θ' / deg	Ref.
$[Fe(bpp^{CO_2Me})_2][ClO_4]_2$	High-spin	158.77(5)	80.73(1)	82.13(3)	29
$[Fe(bpp^{CO_2C_6H_3\{OMe\}_2})_2][BF_4]_2 \cdot 2CH_3NO_2$	High-spin	166.4(1)	79.90(3)	83.85(9)	30
$[Fe(bpp^{NH_2})_2][BF_4]_2 \cdot 3CH_3NO_2$	High-spin	166.47(7)	89.33(2)	86.63(7)	20
$[Fe(bpp^{NH_2})_2][ClO_4]_2 \cdot 3CH_3NO_2$	High-spin	166.80(7)	88.57(2)	87.52(4)	20
$[Fe(bpp^{NH_2})_2]_2[BF_4]_2[SiF_6] \cdot 1.5H_2O \cdot 2CH_3OH$[c]	High-spin	154.7(2)-164.8(2)	78.04(5)-86.38(5)	78.2(1)-87.4(2)	31
$[Fe(bpp^{NMe_2})_2][BF_4]_2 \cdot xH_2O$	High-spin	164.38(8)	86.86(2)	89.17(6)	20
$[Fe(bpp^{NMe_2})_2][ClO_4]_2 \cdot xH_2O$	High-spin	164.33(8)	87.03(2)	89.20(6)	20
$[Fe(bpp^{NHC\{O\}Me})_2][BF_4]_2 \cdot (CH_3)_2CO$, phase 2 molecules A-J[f]	High-spin	161.4(2)-169.5(2)	79.80(6)-89.97(5)	79.6(3)-88.4(2)	32
$[Fe(bpp^{NHC\{O\}Me})_2][BF_4]_2 \cdot 2CH_3CN$	High-spin	166.03(7)	85.59(2)	86.17(6)	13
$[Fe(bpp^{NHC\{O\}Me})_2][BF_4]_2 \cdot 2CH_3NO_2$	High-spin	176.47(8)	79.88(1)	84.38(3)	13
$[Fe(bpp^{NHC\{O\}Me})_2][BF_4]_2 \cdot CH_3OH$	High-spin	158.3(1)	86.39(3)	84.0(1)	13
$[Fe(bpp^{NHC\{O\}Me})_2][ClO_4]_2 \cdot 2CH_3NO_2$	High-spin	175.3(1)	79.55(1)	84.69(4)	13
$[Fe(bpp^{NHC\{O\}Me})_2][ClO_4]_2 \cdot 0.5C_2H_5OH$	High-spin	164.47(9)	89.62(3)	87.89(8)	13
$[Fe(bpp^{pyrazolyl})_2][BF_4]_2 \cdot 0.5CH_3NO_2$, molecule B[e]	High-spin	167.36(18)	88.08(4)	89.3(1)	16
$[Fe(bpp^{imidazolyl})_2][BF_4]_2$	High-spin	151.26(9)	68.28(2)	70.21(7)	33
$[Fe(bpp^{imidazolyl})_2][ClO_4]_2$	High-spin	151.5(2)	68.41(4)	70.3(2)	33
$[Fe(bpp^{thiomorpholinyl})_2][BF_4]_2 \cdot H_2O$	High-spin	162.78(9)	86.41(3)	88.04(9)	34
$[Fe(bpp^{thiomorpholinyl})_2][ClO_4]_2 \cdot xH_2O$	High-spin	163.22(9)	88.04(3)	89.45(9)	34
$[Fe(bpp^{thiomorpholinyl})_2][CF_3SO_3]_2$	High-spin	161.95(9)	85.95(3)	86.0(1)	34
$[Fe(bpp^{OH})_2][BF_4]_2 \cdot 2CH_3OH$	High-spin	165.55(15)	85.17(3)	84.6(1)	35
$[Fe(bpp^{OH})_2][PF_6]_2 \cdot 2CH_3OH$	High-spin	166.68(6)	87.36(1)	88.65(3)	35
$[Fe(bpp^{OMe})_2][PF_6]_2$	High-spin	153.61(6)	80.80(1)	79.02(5)	19
$[Fe(bpp^{S\mathit{i}Pr})_2][BF_4]_2 \cdot C_2H_5CN$	High-spin	167.62(14)	87.28(4)	87.2(1)	36
$[Fe(bpp^{S\mathit{i}Pr})_2][ClO_4]_2 \cdot CH_3CN$	High-spin	167.15(10)	86.29(3)	88.16(8)	37
$[Fe(bpp^{S\mathit{i}Pr})_2][ClO_4]_2 \cdot (CH_3)_2CO$	High-spin	166.08(13)	84.15(4)	85.3(1)	37
$[Fe(bpp^{Me})_2][PF_6]_2$	High-spin	157.92(9)	67.70(2)	72.21(6)	12
$[Fe(bppyz)_2][SbF_6]_2$	High-spin	154.5(1)	59.84(3)	64.6(1)	
$[Fe(bppyz)_2][SbF_6]_2 \cdot 2CH_3NO_2$	High-spin	163.2(1)	84.66(4)	86.8(1)	
$[Fe(bppyz^{Cl\text{-}4'})_2][BF_4]_2 \cdot 3CH_3NO_2 \cdot (C_2H_5)_2O$, molecule 1[b]	High-spin	171.98(9)	89.23(2)	89.64(8)	38
$[Fe(bpp)_2][BF_4]_2$	SCO	172.98(7)	89.94(2)	89.40(8)	
$[Fe(bpp)_2][Ni(mnt)_2]_2 \cdot CH_3NO_2$	SCO	177.51(10)	76.0	80.5	39
$[Fe(bpp^{Me\text{-}3',3''})_2][BF_4]_2$	SCO	175.85(9)	76.4	83.2	
$[Fe(bpp^{Me\text{-}3',3''})_2][BF_4]_2 \cdot xH_2O$	SCO	175.63(9)	76.4	82.3	

Table S4 continued.

	Spin-state behavior	ϕ / deg	θ / deg	θ' / deg	Ref.
$[Fe(bpp^{Me-4',4''})_2][ClO_4]_2$	SCO	180	90	90	
$[Fe(bpp^{F})_2][BF_4]_2$	SCO	175.3(1)	88.89(4)	87.62(7)	19
$[Fe(bpp^{Br})_2][BF_4]_2$	SCO	175.9(2)	89.64(4)	88.90(9)	40
$[Fe(bpp^{Me})_2][ClO_4]_2$	SCO	163.7(2)	89.48(7)	89.8(2)	12
$[Fe(bpp^{CH_2OH})_2][BF_4]_2$	SCO	178.4(1)	89.71(4)	89.9(1)	
$[Fe(bpp^{CH_2OH})_2][ClO_4]_2$	SCO	177.4(3)	89.4	89.6	
$[Fe(bpp^{CO_2Et})_2][BF_4]_2 \cdot CH_3NO_2$	SCO	159.5(3)	81.4(1)	80.9(3)	14
$[Fe(bpp^{CO_2Et})_2][ClO_4]_2 \cdot CH_3NO_2$	SCO	158.45(8)	80.14(3)	79.68(8)	14
$[Fe(bpp^{CO_2Et})_2][ClO_4]_2 \cdot CH_3CN$	SCO	158.8(2)	78.73(6)	78.5(2)	41
$[Fe(bpp^{C\{S\}NHMe})_2][ClO_4]_2$	SCO	171.82(9)	79.79(3)	85.87(9)	42
β-$[Fe(bpp^{CH_2SCN})_2][BF_4]_2$	SCO	172.34(8)	88.5	88.7	
$[Fe(bpp^{E\text{-}CH=CHPh})_2][BF_4]_2 \cdot (CH_3)_2CO$	SCO	161.9	73.0	76.4	26
$[Fe(bpp^{C_6H_4C\equiv CH})_2][BF_4]_2$, polymorph C	SCO	172.1(2)	79.9	82.6	43
$[Fe(bpp^{NHC\{O\}Me})_2][BF_4]_2 \cdot (CH_3)_2CO$, phase 1	SCO	169.7(1)	88.50(4)	88.16(9)	32
$[Fe(bpp^{NHC\{O\}Me})_2][BF_4]_2 \cdot CH_3CN$	SCO	168.6(2)	85.48(9)	85.3(2)	13
$[Fe(bpp^{NHC\{O\}Me})_2][ClO_4]_2 \cdot CH_3CN$	SCO	167.8(2)	84.79(6)	84.8(2)	13
$[Fe(bpp^{NHC\{O\}Me})_2][BF_4]_2 \cdot C_2H_5CN$	SCO	165.78(8)	84.17(3)	84.5(1)	13
$[Fe(bpp^{NHC\{O\}Me})_2][ClO_4]_2 \cdot C_2H_5CN$	SCO	165.1(1)	84.53(4)	84.0(2)	13
$[Fe(bpp^{SMe})_2][BF_4]_2$, phase 1[d]	SCO	178.6(3), 168.3(2)	89.04(5), 86.55(5)	88.4(1), 85.7(1)	40
$[Fe(bpp^{SMe})_2][ClO_4]_2$, phase 1[d]	SCO	177.7(3), 168.9(2)	89.51(4), 87.14(4)	88.7(1), 86.4(1)	44
$[Fe(bpp^{S\textit{i}Pr})_2][BF_4]_2$	SCO	162.3(2)	83.52(5)	86.0(1)	36
$[Fe(bpp^{S\textit{i}Pr})_2][BF_4]_2 \cdot H_2O$	SCO	168.6(1)	86.61(3)	88.59(9)	36
$[Fe(bpp^{S\textit{i}Pr})_2][BF_4]_2 \cdot CH_3CN$	SCO	167.8(2)	86.89(6)	88.7(2)	36
$[Fe(bpp^{S\textit{i}Pr})_2][BF_4]_2 \cdot CH_3NO_2$	SCO	169.5(1)	85.54(4)	88.2(1)	36
$[Fe(bpp^{S\textit{i}Pr})_2][BF_4]_2 \cdot (CH_3)_2CO$	SCO	166.7(2)	86.32(4)	86.3(1)	36
$[Fe(bpp^{S\textit{i}Pr})_2][ClO_4]_2$	SCO	164.6(1)	85.08(4)	87.7(1)	37
$[Fe(bpp^{S\textit{i}Pr})_2][ClO_4]_2 \cdot H_2O$	SCO	169.2(1)	87.13(4)	89.0(1)	37
$[Fe(bpp^{S\textit{i}Pr})_2][ClO_4]_2 \cdot CH_3NO_2$	SCO	168.7(1)	85.15(3)	87.83(8)	37
$[Fe(bpp^{S\textit{t}Bu})_2][BF_4]_2 \cdot CH_3NO_2$, phase 3[g]	SCO	165.5(6)	77.85(7)	83.3(2)	45
$[Fe(bpp^{S\textit{t}Bu})_2][BF_4]_2 \cdot CH_3CN$[d]	SCO	168.1(2), 167.0(2)	77.09(6), 81.18(6)	83.1(2), 84.2(2)	45
$[Fe(bpp^{S\textit{t}Bu})_2][ClO_4]_2 \cdot CH_3NO_2$, phase 1	SCO	173.83(11)	88.81(4)	88.9(1)	45
$[Fe(bpp^{S\textit{t}Bu})_2][ClO_4]_2 \cdot CH_3CN$, phase 1	SCO	173.15(9)	86.77(3)	88.0(1)	45
$[Fe(bpp^{SS\textit{i}Pr})_2][BF_4]_2$	SCO	165.2(1)	82.11(4)	85.5(1)	15

Table S4 continued.

	Spin-state behavior	ϕ / deg	θ / deg	θ' / deg	Ref.
$[Fe(bpp^{SS\mathit{i}Pr})_2][ClO_4]_2$	SCO	166.4(1)	83.07(4)	86.2(1)	15
$[Fe(bpp^{SS\mathit{i}Pr})_2][BF_4]_2\cdot(CH_3)_2CO$	SCO	163.0(1)	82.60(5)	81.3(1)	15
$[Fe(bpp^{SS\mathit{i}Pr})_2][ClO_4]_2\cdot(CH_3)_2CO$	SCO	163.7(1)	82.77(4)	81.2(1)	15
$[Fe(bpp^{F\text{-}4',4''})_2][BF_4]_2$, phase 1	SCO	180	90	90	46
$[Fe(bpp^{F\text{-}4',4''})_2][ClO_4]_2$	SCO	178.3(1)	88.07(4)	89.0(1)	46
α-$[Fe(bpp^{Cl\text{-}4',4''})_2][BF_4]_2$	SCO	180	90	90	
$[Fe(\{bpp^{Br\text{-}4',4''})_2][BF_4]_2$	SCO	180	90	90	
$[Fe(bppyz)_2][BF_4]_2\cdot3CH_3NO_2$	SCO	173.2(1)	86.42(4)	88.4(1)	
$[Fe(bppyz^{Me\text{-}3',3''})_2][BF_4]_2$	SCO	180	90	90	
$[Fe(bppyz^{Me\text{-}3',3''})_2][ClO_4]_2$	SCO	180	90	90	
$[Fe(bppyz^{Me\text{-}4',4''})_2][BF_4]_2$	SCO	180	90	90	38

[a]There are six unique high-spin molecules in the asymmetric unit of this crystal. [b]There are two unique molecules in the asymmetric unit of this crystal, one of which is high-spin while the other is SCO-active. [c]There are four unique high-spin molecules in the asymmetric unit of this crystal. [d]There are two unique high-spin molecules in the asymmetric unit of this crystal. [e]There are two unique molecules in the asymmetric unit of this crystal, one of which is high-spin while the other is low-spin. [f]The low-temperature phase of this crystal contains twenty-four unique molecules, ten of which are high-spin. [g]Average values quoted for two ligand disorder orientations in the refinement.

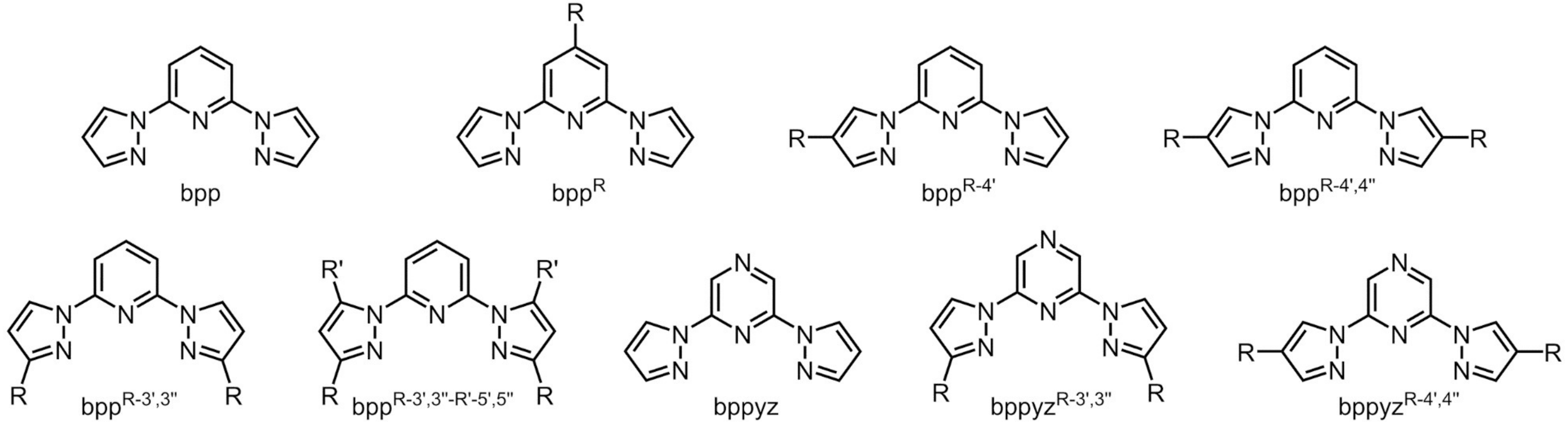

Chart S4 The ligand abbreviations used in Table S4.

[Fe(bpp)$_2$]$^{2+}$

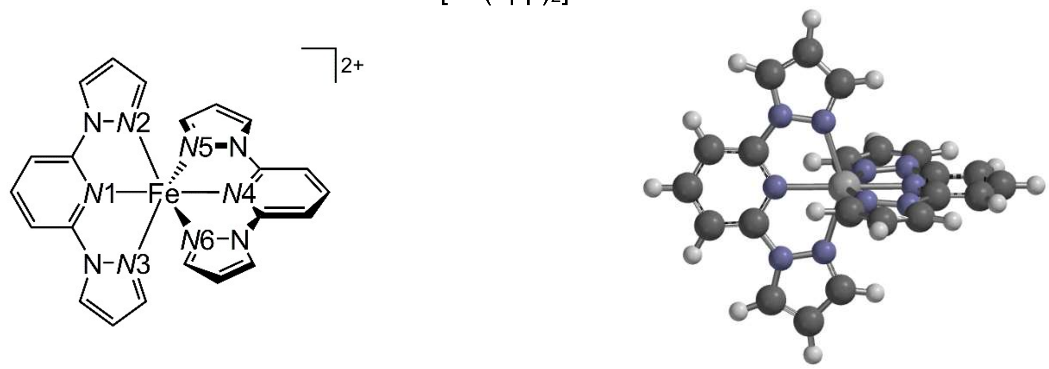

[Fe(bpp^{NO_2})$_2$]$^{2+}$

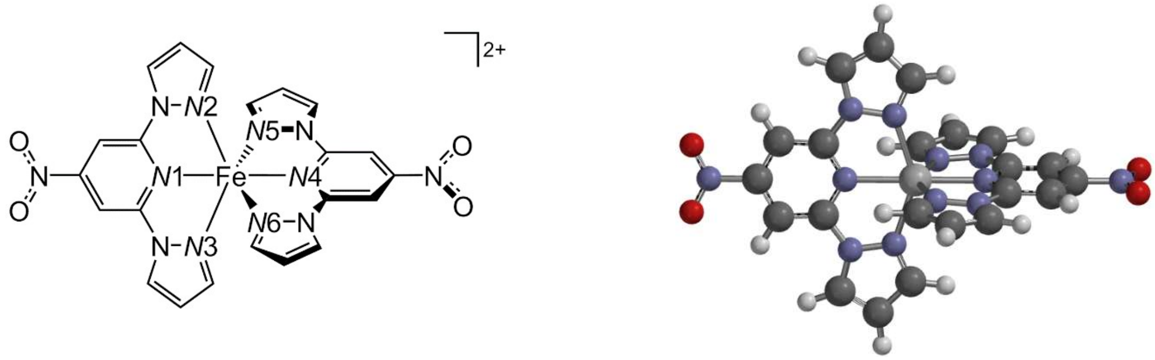

[Fe(bpp^{CN})$_2$]$^{2+}$

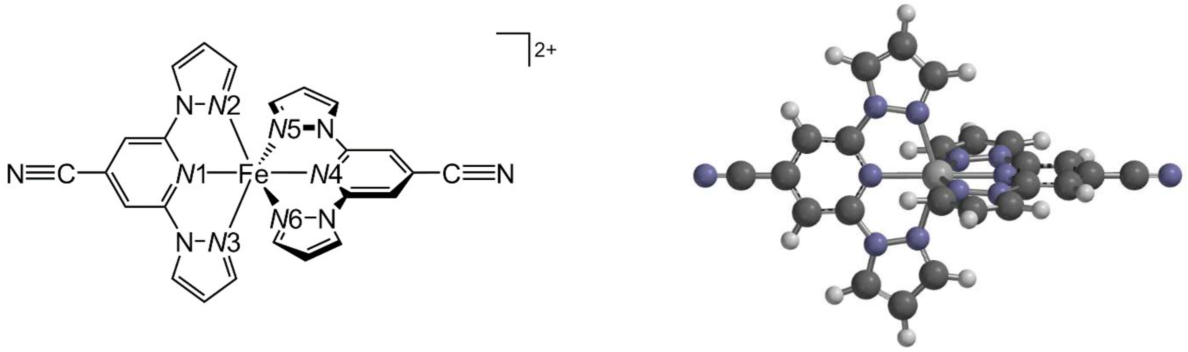

[Fe(bpp^{NMe_2})$_2$]$^{2+}$

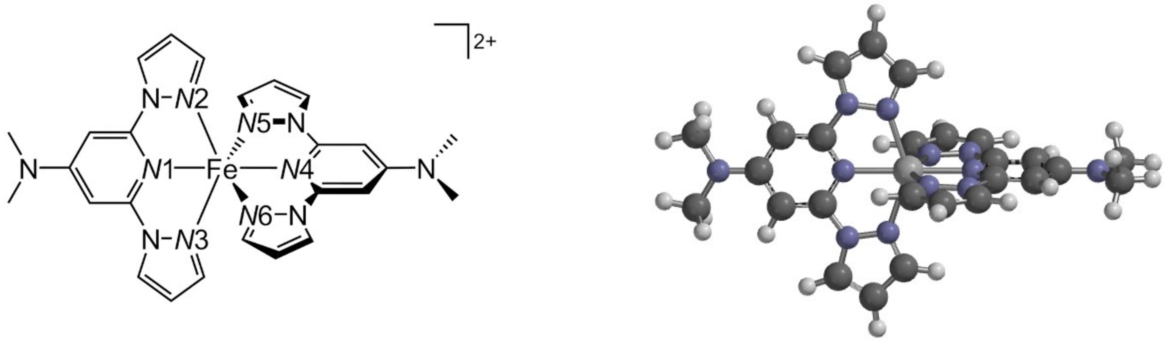

Figure S12 The minimized undistorted structures of the complexes computed in this study. The atom numbering used in Tables S6-S14 is also shown (which is different from the atom numbering in the crystal structures).

Color code: C, dark gray; H, white; Fe, pale gray; N, blue; O, red; S, yellow.

[Fe(bpp^{SMe})$_2$]$^{2+}$

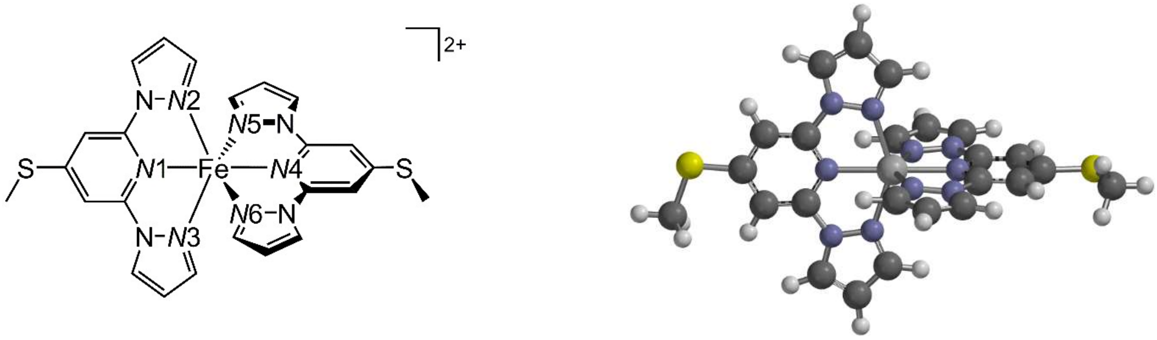

[Fe(bpp^{SPh})$_2$]$^{2+}$

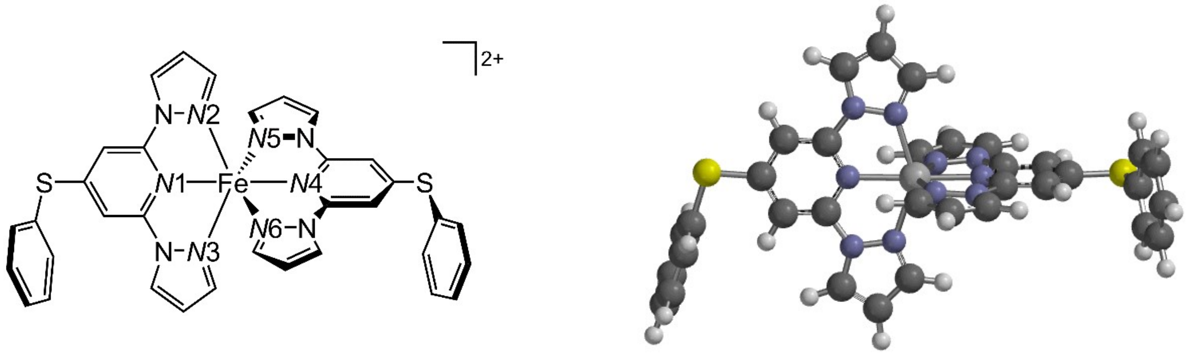

[Fe*L*$_2$]$^{2+}$

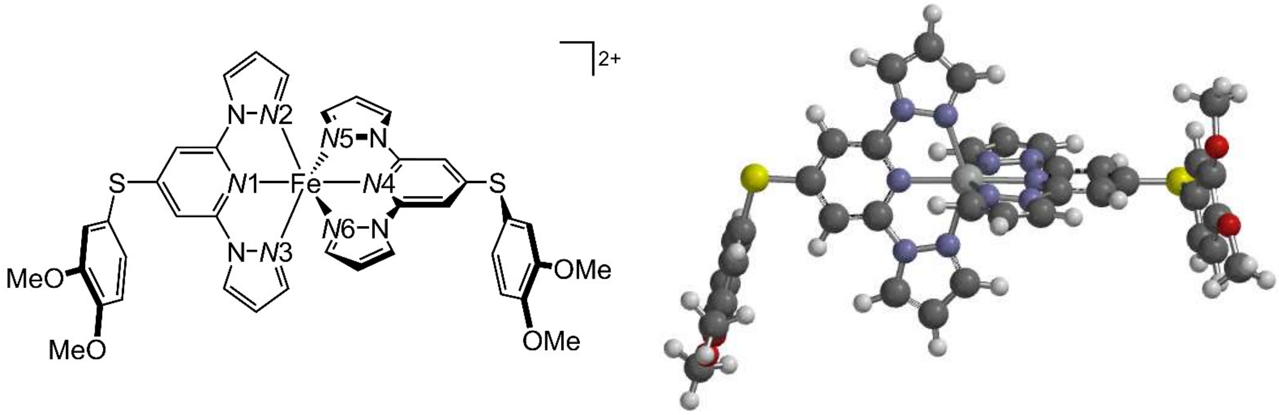

Figure S12 continued.

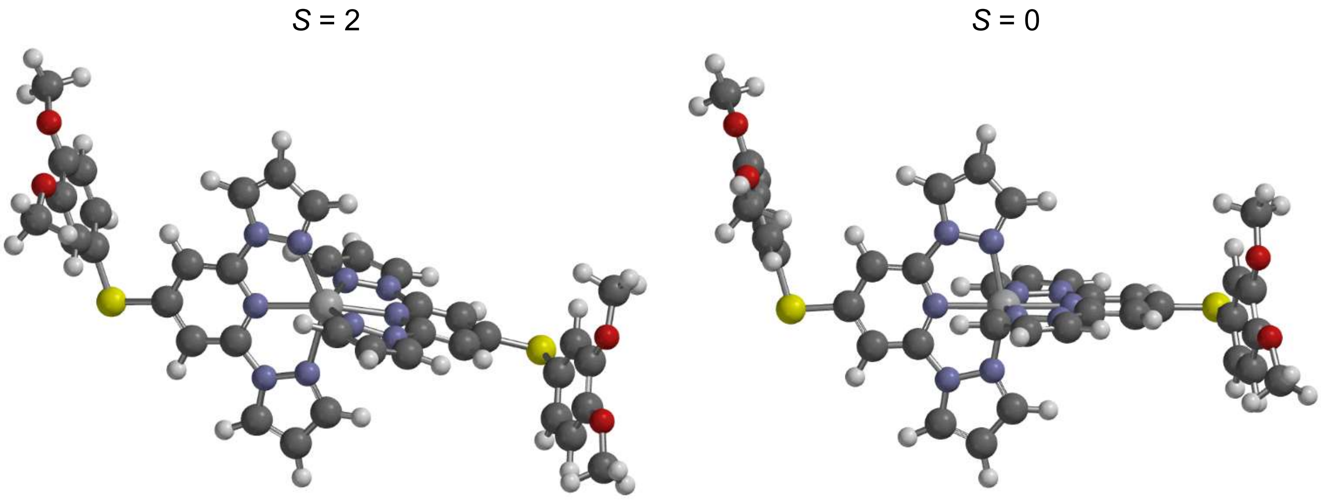

Figure S13 Freely minimized structures of the high-spin ($S = 2$) and low-spin ($S = 0$) states of $[FeL_2]^{2+}$. An overlay of these structures is shown in Figure 3 of the main article.

Color code: C, dark gray; H, white; Fe, pale gray; N, blue; O, red; S, yellow.

Table S5 Metric parameters and electronic energies for the freely minimized geometries of $[FeL_2]^{2+}$ (Å, °; Figure S13). The atom numbering is shown in Figure S12. ΔFe is the distance that the Fe atom protrudes from the plane of the three N donors of each ligand, and ϕ, θ and θ' are defined on page S8.

	High-spin	Low-spin
Fe–*N*1	2.143	1.918
Fe–*N*2	2.194	2.004
Fe–*N*3	2.192	2.005
Fe–*N*4	2.143	1.918
Fe–*N*5	2.194	2.004
Fe–*N*6	2.191	2.005
*N*1–Fe–*N*2	72.4	79.5
*N*1–Fe–*N*3	72.8	79.5
*N*1–Fe–*N*4 (ϕ)	163.1	179.7
*N*1–Fe–*N*5	96.2	100.3
*N*1–Fe–*N*6	119.9	100.7
*N*2–Fe–*N*3	143.8	158.9
*N*2–Fe–*N*4	96.2	100.3
*N*2–Fe–*N*5	97.3	91.8
*N*2–Fe–*N*6	96.2	91.9
*N*3–Fe–*N*4	120.0	100.7
*N*3–Fe–*N*5	96.2	91.9
*N*3–Fe–*N*6	92.4	92.0
*N*4–Fe–*N*5	72.4	79.5
*N*4–Fe–*N*6	72.8	79.5
*N*5–Fe–*N*6	143.8	158.9
ΔFe	0.317	0.003
θ	83.7	90.0
θ'	83.2	90.0
E (Ha)	−4376.35040	−4376.34650

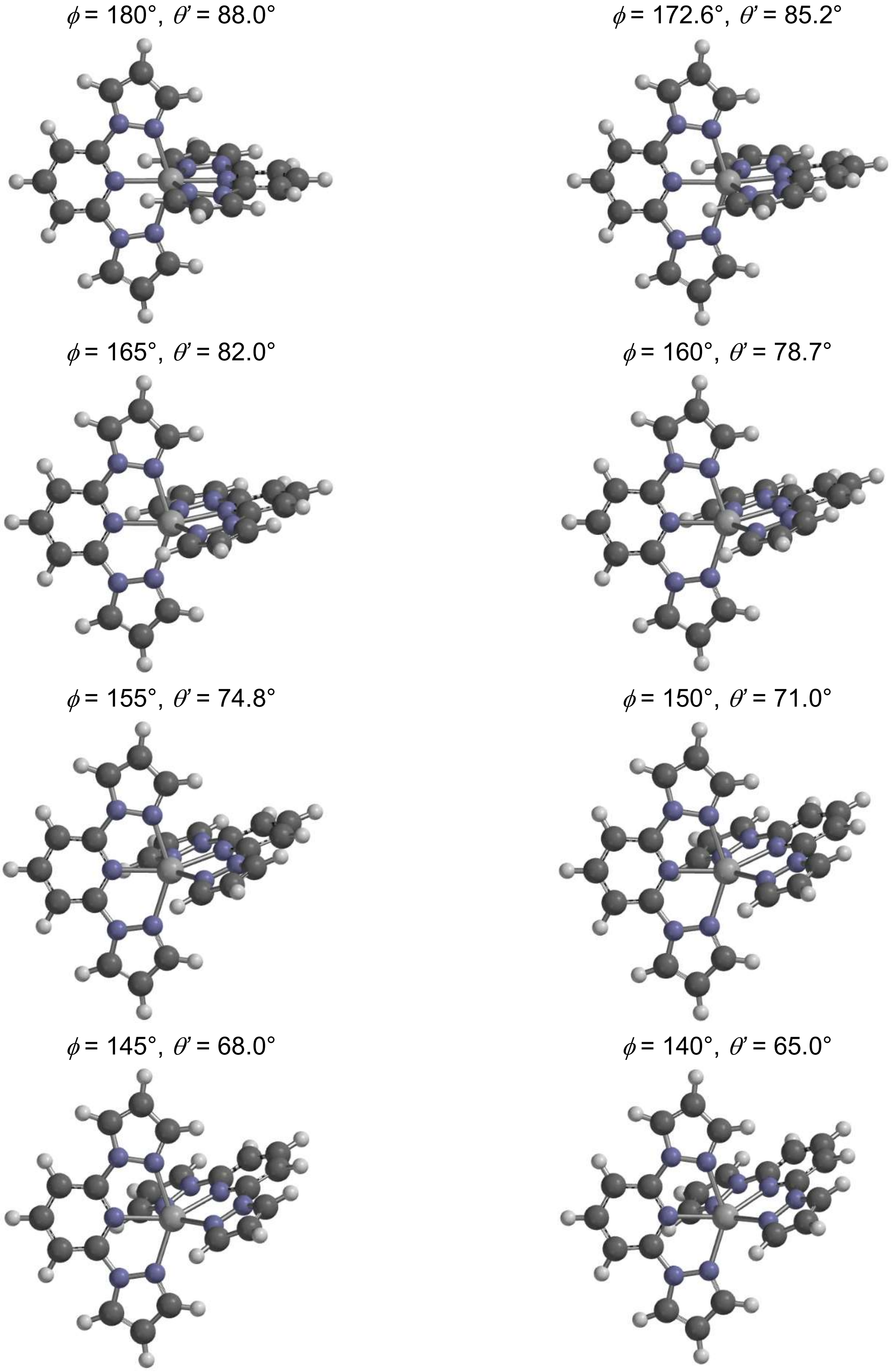

Figure S14 Computed structures for $[Fe(bpp)_2]^{2+}$ along the minimum energy distortion pathway A.

Color code: C, dark gray; H, white; Fe, pale gray; N, blue.

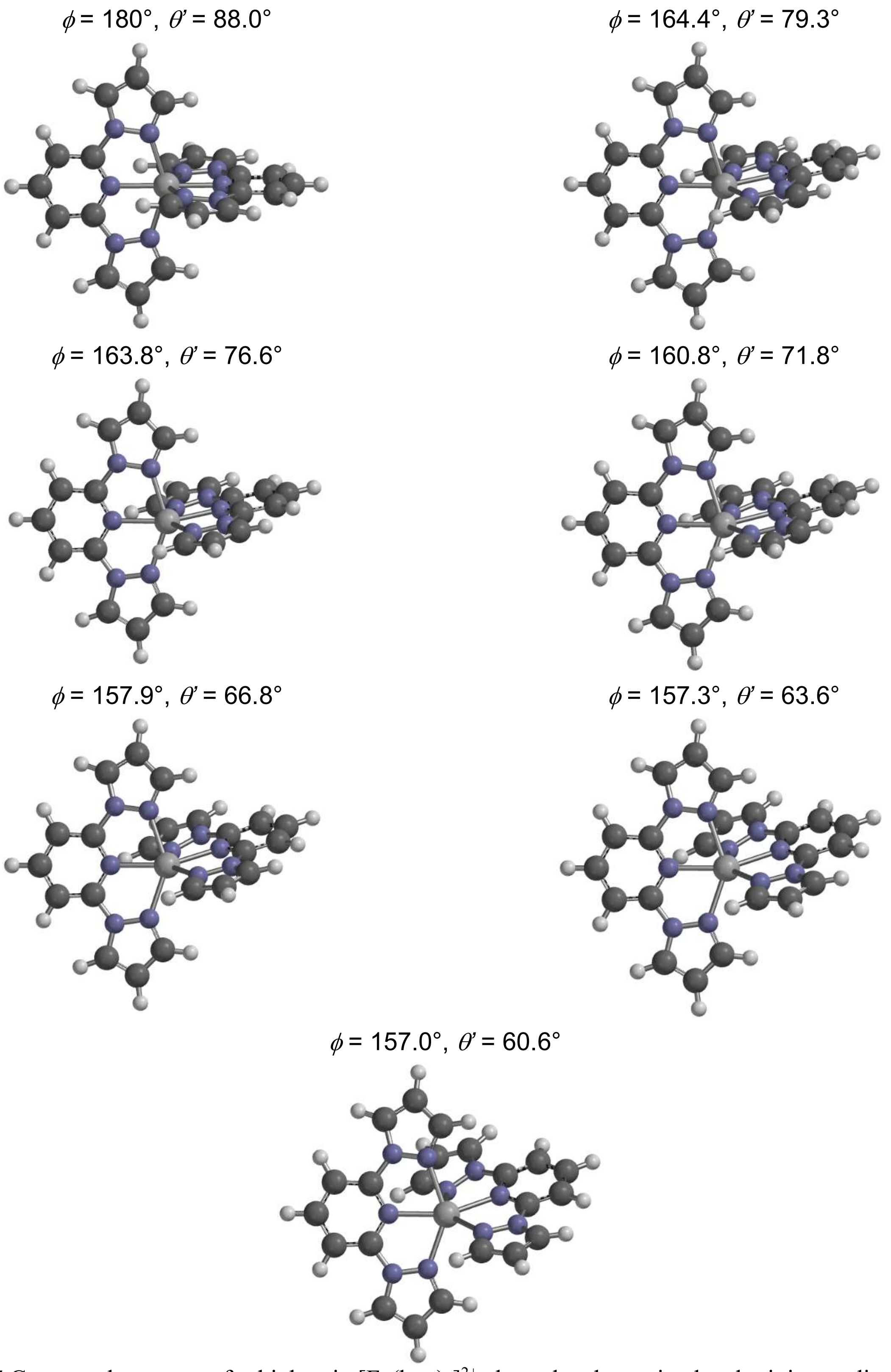

Figure S15 Computed structures for high-spin $[Fe(bpp)_2]^{2+}$ along the alternative local minimum distortion pathway B. This pathway was located in calculations where θ' was constrained across a range of values, but ϕ was freely minimized. ϕ adopts a narrower range of values in this pathway, compared to pathway A.

Color code: C, dark gray; H, white; Fe, pale gray; N, blue.

ϕ = 180°, θ' = 88.0°

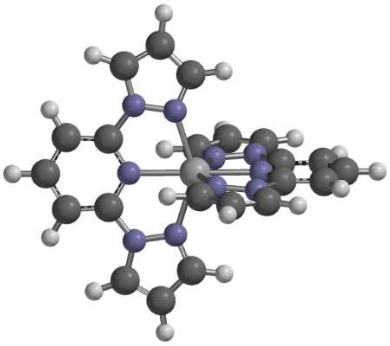

ϕ = 165°, θ' = 89.5°

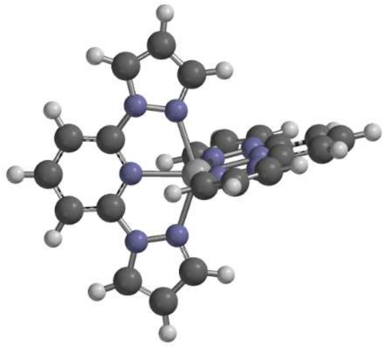

ϕ = 160°, θ' = 89.5°

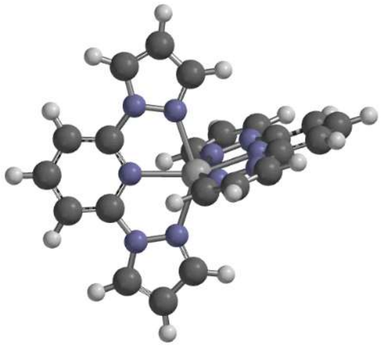

ϕ = 155°, θ' = 89.2°

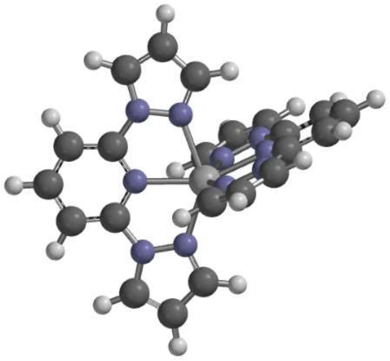

ϕ = 150°, θ' = 89.2°

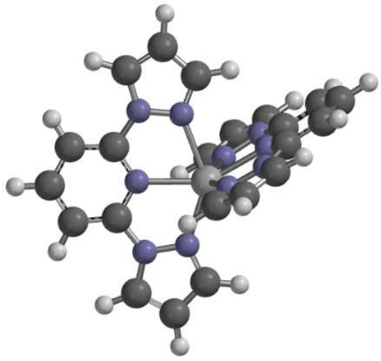

ϕ = 145°, θ' = 87.5°

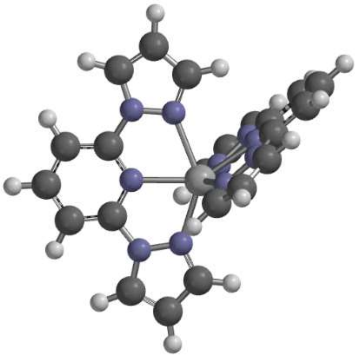

ϕ = 140°, θ' = 87.4°

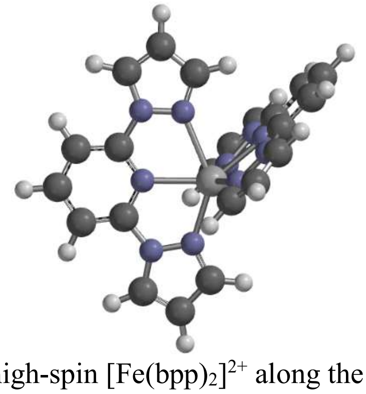

Figure S16 Computed structures for high-spin $[Fe(bpp)_2]^{2+}$ along the alternative local minimum distortion pathway C. This was accessed from initial models where ϕ varied, but θ' = 90°. The ϕ angle was fixed during these minimizations. θ' was allowed to refine, but remained near 90°.

Color code: C, dark gray; H, white; Fe, pale gray; N, blue.

ϕ = 180°, θ' = 88.0°

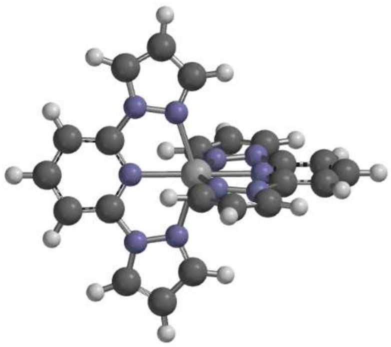

ϕ = 180.0°, θ' = 84.8°

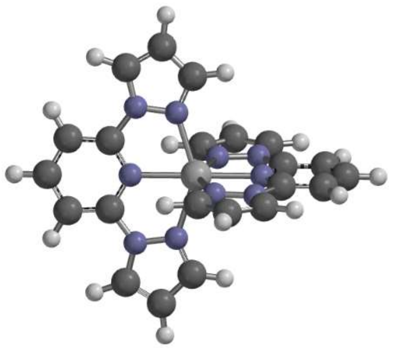

ϕ = 179.9°, θ' = 81.3°

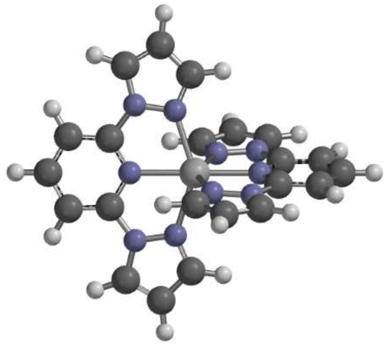

ϕ = 179.8°, θ' = 78.1°

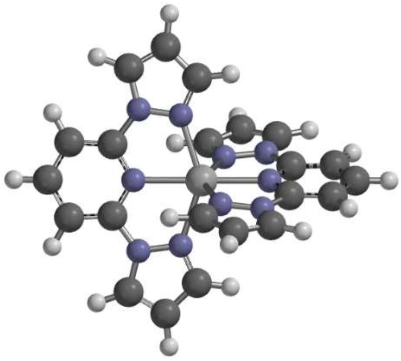

ϕ = 179.8°, θ' = 75.0°

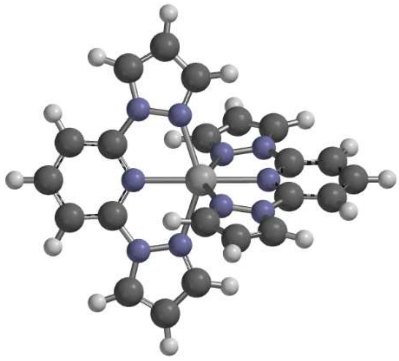

ϕ = 180.0°, θ' = 72.8°

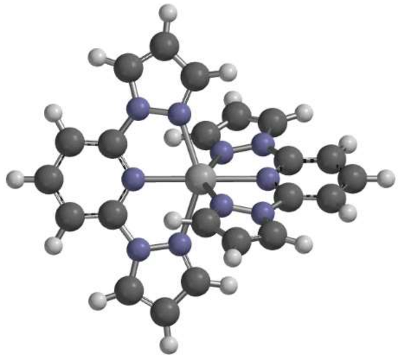

ϕ = 180.0°, θ' = 69.6°

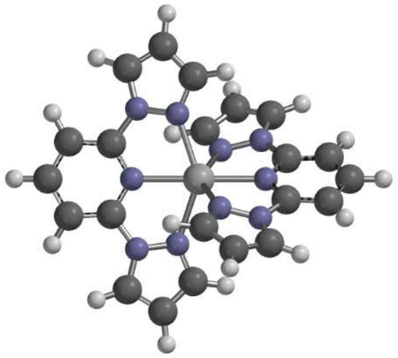

Figure S17 Computed structures for high-spin $[Fe(bpp)_2]^{2+}$ along the alternative local minimum distortion pathway D. This was accessed from initial models where θ' varied, but ϕ = 180°. θ' was constrained during these minimizations, across a range of values. ϕ was minimized, but remained near 180°.

Color code: C, dark gray; H, white; Fe, pale gray; N, blue.

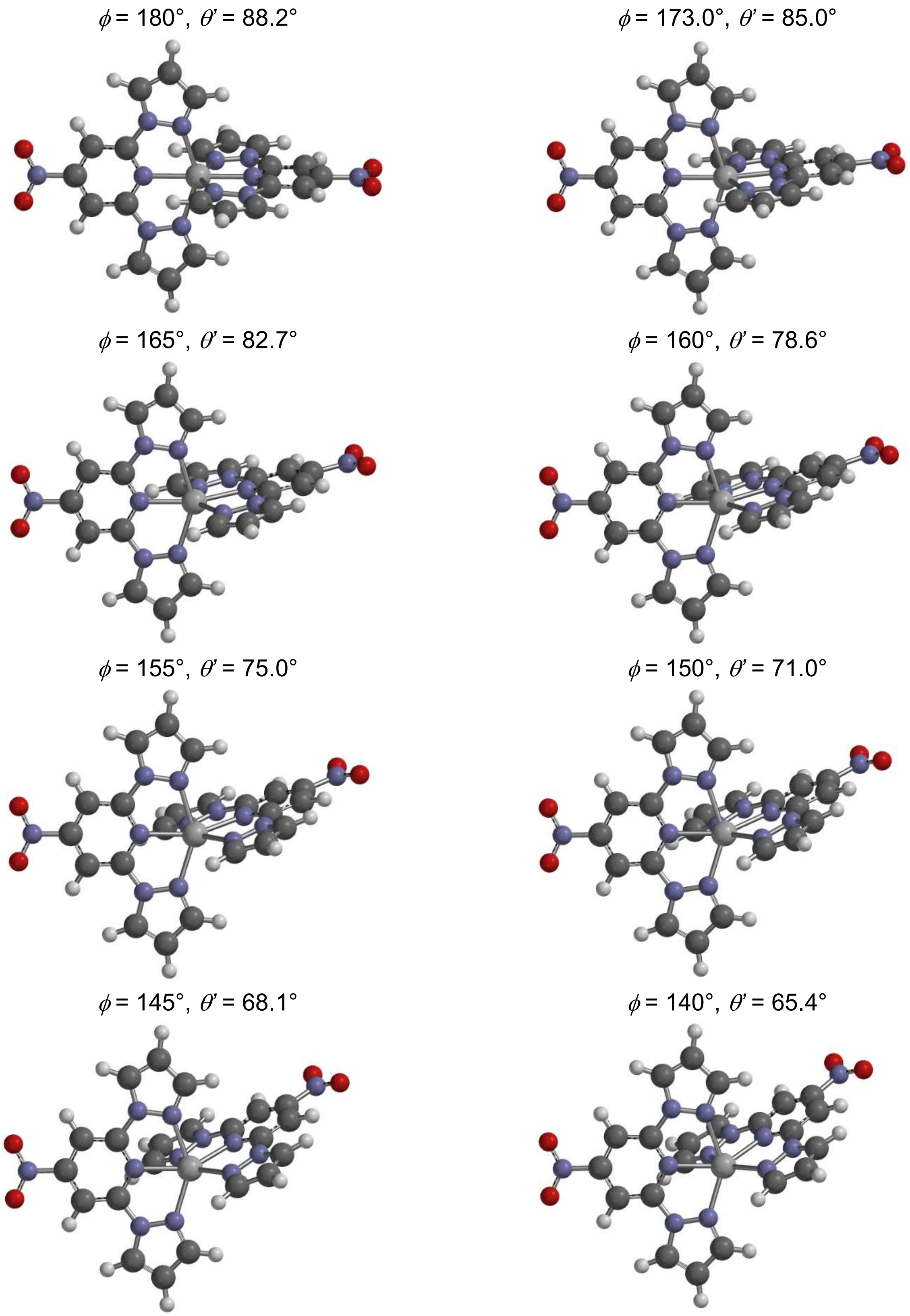

Figure S18 Computed structures for $[Fe(bpp^{NO_2})_2]^{2+}$ along the minimum energy distortion pathway A.

Color code: C, dark gray; H, white; Fe, pale gray; N, blue; O, red.

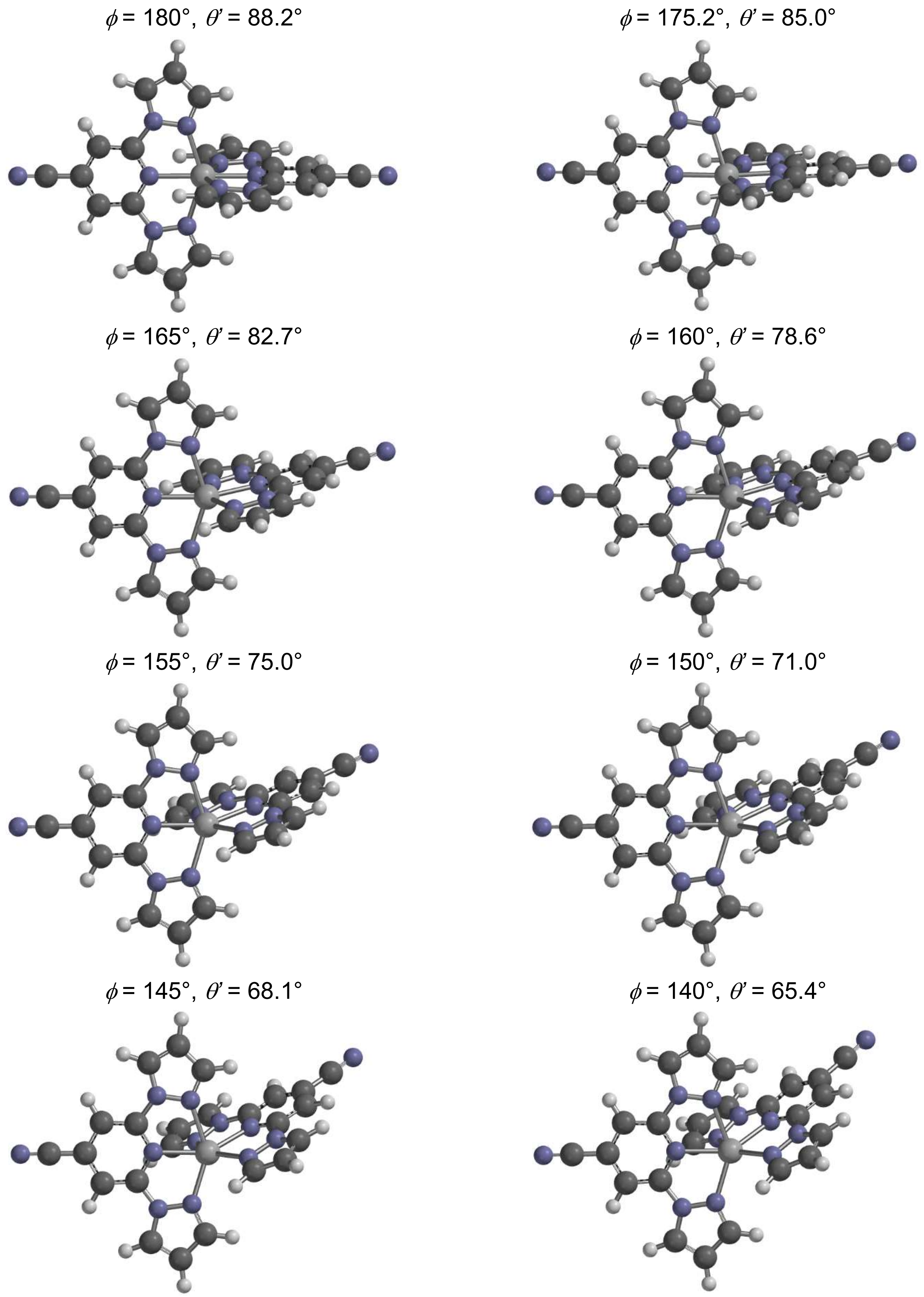

Figure S19 Computed structures for $[Fe(bpp^{CN})_2]^{2+}$ along the minimum energy distortion pathway A.

Color code: C, dark gray; H, white; Fe, pale gray; N, blue.

ϕ = 179.9°, θ' = 89.7°

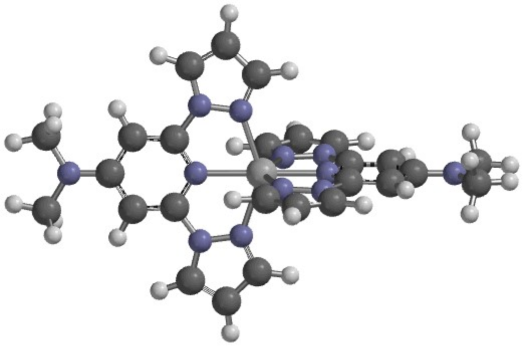

ϕ = 174.2°, θ' = 88.5°

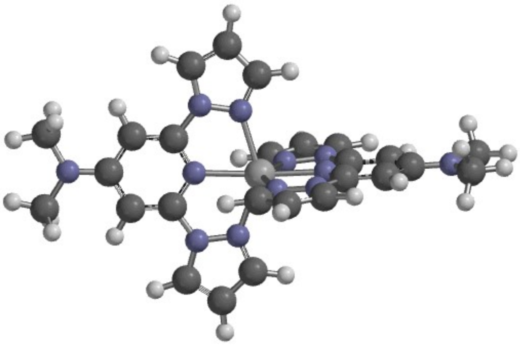

ϕ = 165°, θ' = 85.5°

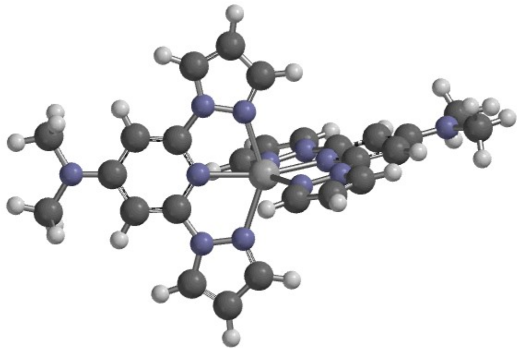

ϕ = 160°, θ' = 82.1°

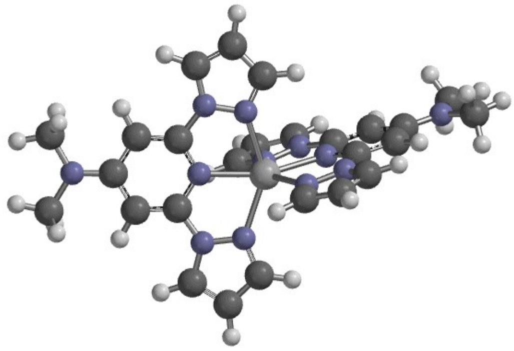

ϕ = 155°, θ' = 78.4°

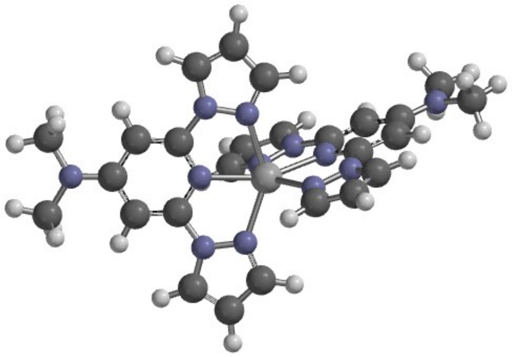

ϕ = 150°, θ' = 74.8°

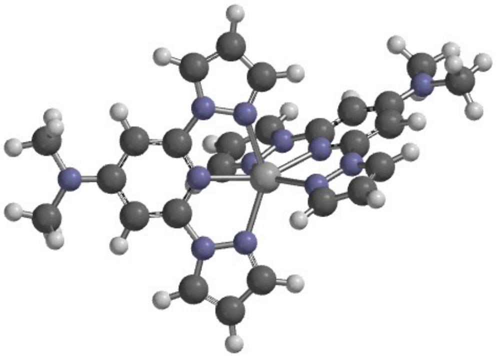

ϕ = 145°, θ' = 72.5°

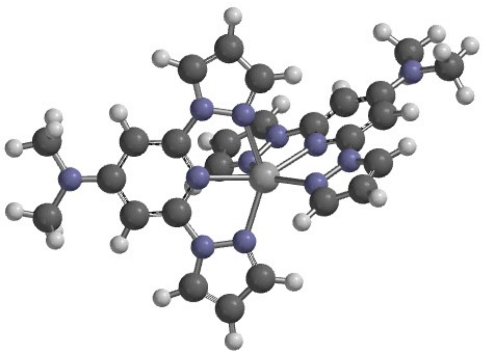

ϕ = 140°, θ' = 70.4°

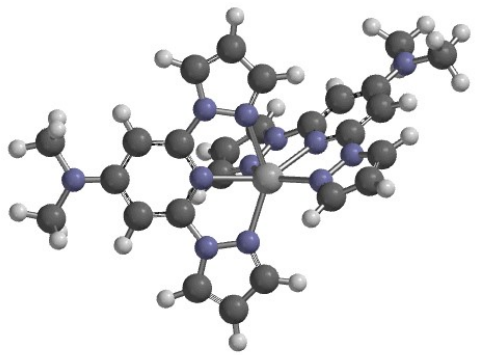

Figure S20 Computed structures for $[Fe(bpp^{NMe_2})_2]^{2+}$ along the minimum energy distortion pathway A.

Color code: C, dark gray; H, white; Fe, pale gray; N, blue.

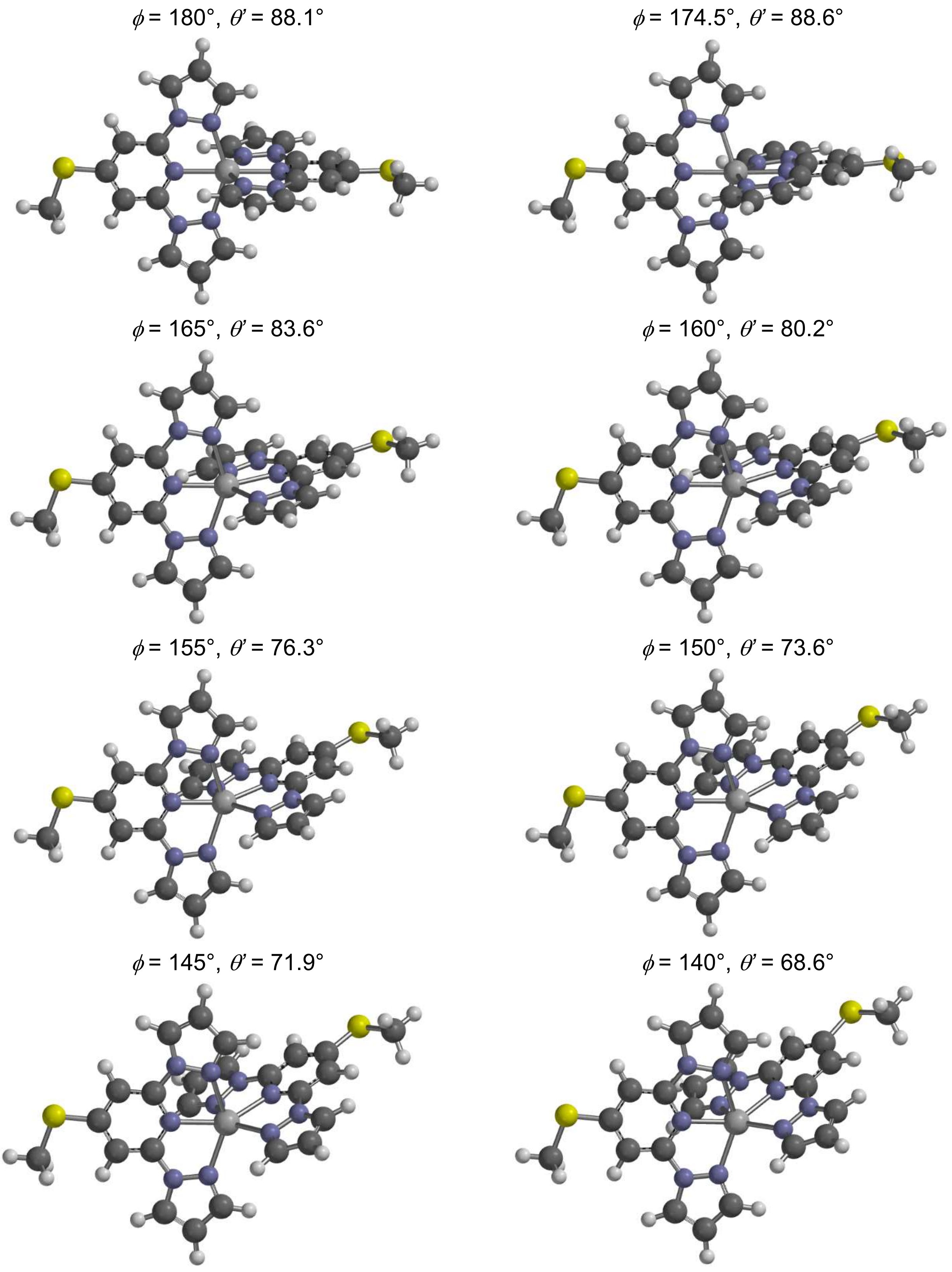

Figure S21 Computed structures for $[Fe(bpp^{SMe})_2]^{2+}$ along the minimum energy distortion pathway A.

Color code: C, dark gray; H, white; Fe, pale gray; N, blue; S, yellow.

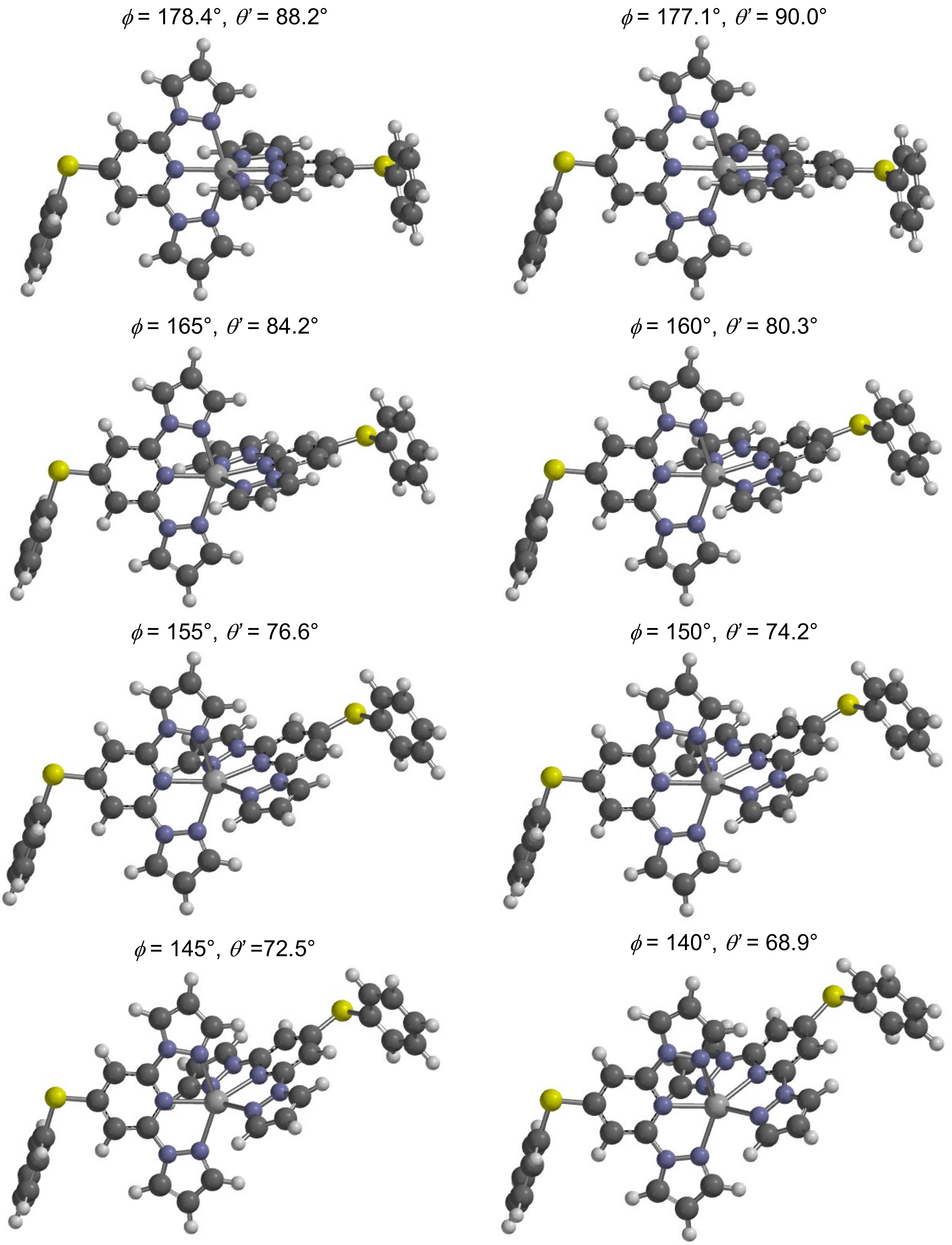

Figure S22 Computed structures for $[Fe(bpp^{SPh})_2]^{2+}$ along the minimum energy distortion pathway A.

Color code: C, dark gray; H, white; Fe, pale gray; N, blue; S, yellow.

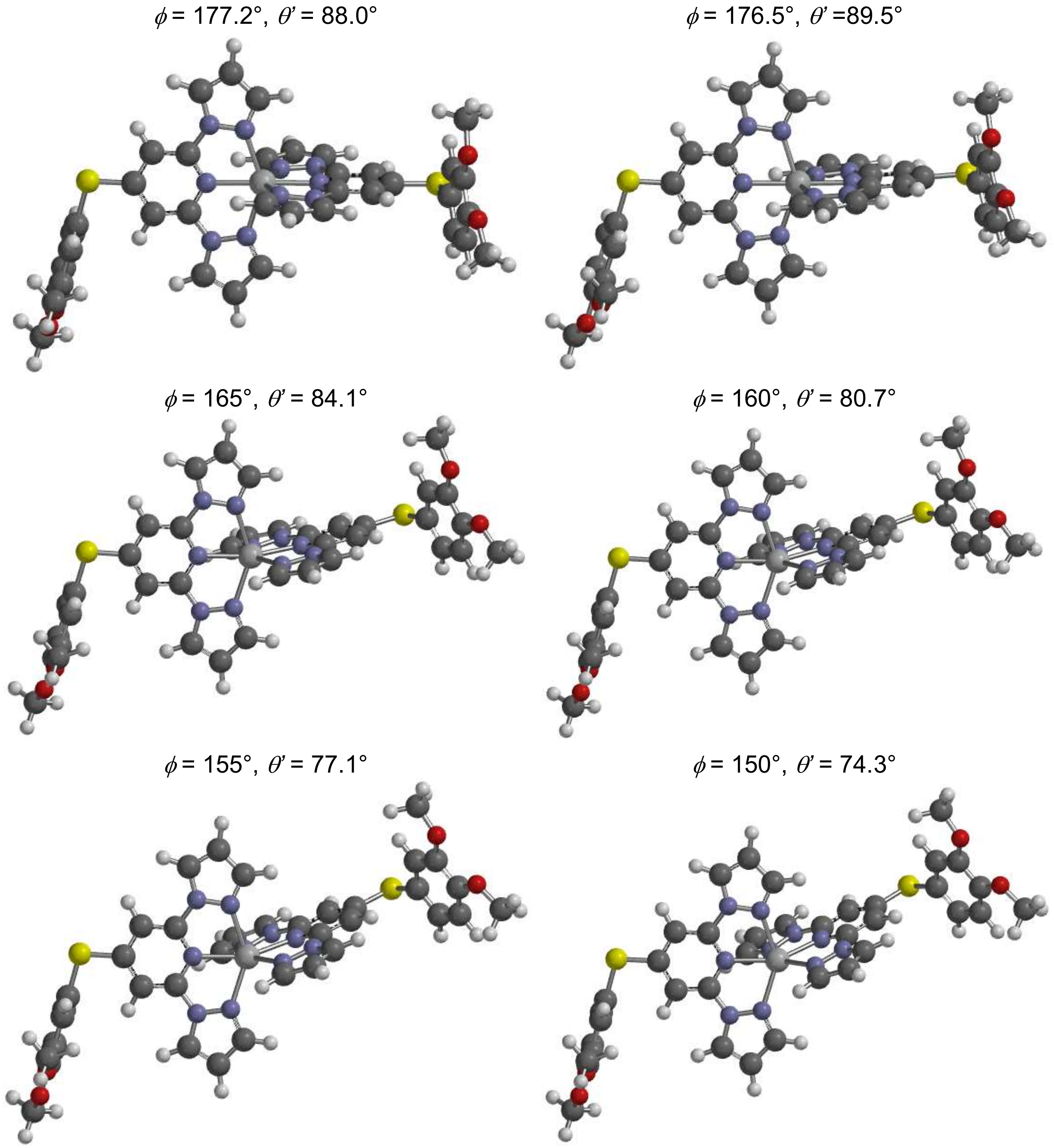

Figure S23 Computed structures for $[FeL_2]^{2+}$ along the minimum energy distortion pathway A.

Color code: C, dark gray; H, white; Fe, pale gray; N, blue; O, red; S, yellow.

ϕ = 145°, θ' = 71.8°

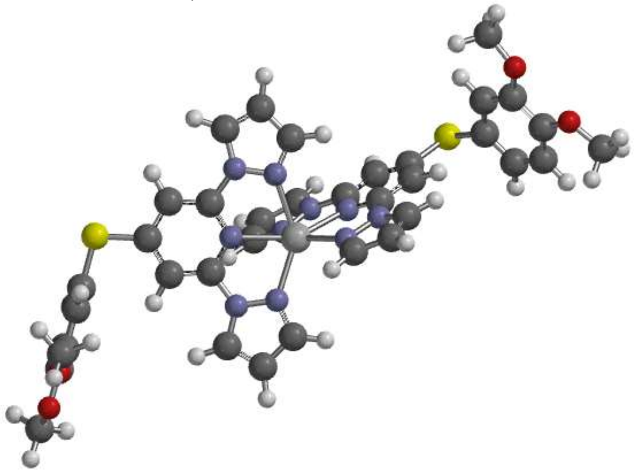

ϕ = 140°, θ' = 69.7°

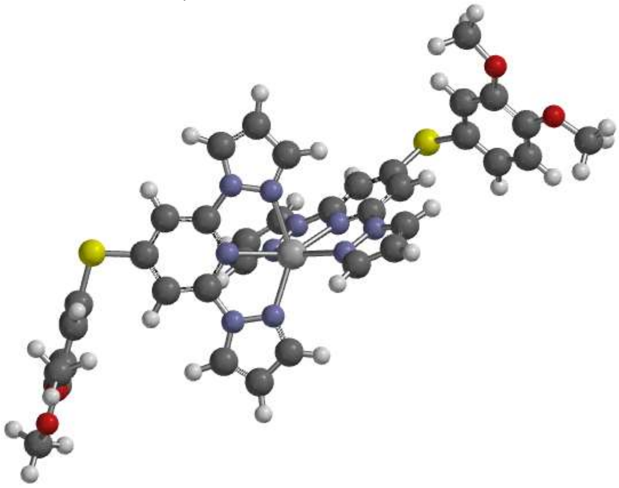

Figure S23 continued.

Table S6 Computed energies of minimized high-spin $[Fe(bpp)_2]^{2+}$ along distortion pathways A-D (Figures S13-S16). See page S8 for definitions of ϕ and θ'.

Pathway A								
ϕ / deg	180.0[a]	172.6	165[b]	160[b]	155[b]	150[b]	145[b]	140[b]
θ' / deg	88.0[a,c]	85.2	82.0	78.7	74.8	71.0	68.0	65.0
E / Ha	–2659.87910	–2659.87907	–2659.87910	–2659.87887	–2659.87828	–2659.87729	–2659.87551	–2659.87282
Pathway B								
ϕ / deg	180.0[a]	164.4	163.8	160.8	157.9	157.3	157.0	–
θ' / deg	88.0[a,c]	79.3	76.6[c]	71.8[c]	66.8[c]	63.6[c]	60.6[c]	–
E / Ha	–2659.87910	–2659.87880	–2659.87836	–2659.87709	–2659.87490	–2659.87195	–2659.86775	–
Pathway C								
ϕ / deg	180.0[a]	–	165[b]	160[b]	155[b]	150[b]	145[b]	140[b]
θ' / deg	88.0[a,c]	–	89.5	89.5	89.2	89.2	87.5	87.4
E / Ha	–2659.87910	–	–2659.87850	–2659.87799	–2659.87714	–2659.87585	–2659.87404	–2659.87105
Pathway D								
ϕ / deg	180.0[a]	180.0	179.9	179.8	179.8	180.0	180.0	–
θ' / deg	88.0[a,c]	84.8[c]	81.3[c]	78.1[c]	75.0[c]	72.2[c]	69.6[c]	–
E / Ha	–2659.87910	–2659.87902	–2659.87806	–2659.87633	–2659.87367	–2659.87004	–2659.86560	–

[a]This is the "undistorted" minimization of this molecule where θ' was constrained to be near 90° – see the text for more details. [b]Fixed value. [c]Constrained during the minimization.

Table S7 Computed metric parameters for the energy-minimized geometries of $[Fe(bpp)_2]^{2+}$ along distortion pathway A (Å, °; Figure S14). The atom numbering is shown in Figure S12, and crystallographic data are included in square brackets for comparison, where this is available. ΔFe is the distance that the Fe atom protrudes from the plane of the three N donors of each ligand, and ϕ, θ and θ' are defined on page S8.

ϕ	180[a]	172.6 [173.15(10)][b]	165[c]	160[c]	155[c]	150[c]	145[c]	140[c]
Fe–*N*1	2.163	2.165 [2.1248(19)]	2.167	2.171	2.175	2.179	2.184	2.189
Fe–*N*2	2.198	2.198 [2.193(2)]	2.208	2.207	2.213	2.227	2.247	2.273
Fe–*N*3	2.198	2.196 [2.175(3)]	2.189	2.187	2.182	2.172	2.161	2.152
Fe–*N*4	2.163	2.165 [2.127(2)]	2.167	2.171	2.175	2.179	2.184	2.189
Fe–*N*5	2.198	2.198 [2.185(3)]	2.208	2.207	2.213	2.227	2.247	2.273
Fe–*N*6	2.198	2.197 [2.184(3)]	2.189	2.186	2.182	2.172	2.161	2.153
*N*1–Fe–*N*2	72.9	72.8 [73.47(8)]	72.5	72.3	71.9	71.4	70.8	70.2
*N*1–Fe–*N*3	72.9	72.9 [73.65(9)]	73.0	72.9	72.9	73.1	73.4	73.7
*N*1–Fe–*N*4	180.0	172.6 [173.15(10)]	165.0	160.0	155.0	150.0	145.0	140.0
*N*1–Fe–*N*5	107.1	101.9 [100.21(10)]	96.7	93.5	90.3	87.2	84.2	81.5
*N*1–Fe–*N*6	107.1	112.6 [113.14(9)]	118.2	122.4	126.6	130.4	134.0	137.5
*N*2–Fe–*N*3	145.8	145.5 [147.08(9)]	145.1	144.1	143.2	142.3	141.7	140.9
*N*2–Fe–*N*4	107.1	101.9 [104.24(8)]	96.7	93.5	90.3	87.1	84.2	81.5
*N*2–Fe–*N*5	96.8	92.1 [92.88(9)]	90.1	90.8	90.3	89.4	88.8	89.0
*N*2–Fe–*N*6	93.1	98.5 [98.46(9)]	99.3	99.8	100.5	101.0	100.8	100.1
*N*3–Fe–*N*4	107.1	112.6 [108.21(9)]	118.2	122.4	126.5	130.5	134.1	137.5
*N*3–Fe–*N*5	93.1	98.5 [91.12(10)]	99.3	99.8	100.5	101.1	100.8	100.1
*N*3–Fe–*N*6	96.8	91.1 [95.95(9)]	91.9	91.5	91.6	92.5	94.3	96.4
*N*4–Fe–*N*5	72.9	72.8 [73.31(10)]	72.5	72.3	71.9	71.4	70.8	70.2
*N*4–Fe–*N*6	72.9	72.9 [73.44(9)]	73.0	72.9	72.9	73.1	73.4	73.7
*N*5–Fe–*N*6	145.8	145.5 [146.58(9)]	145.1	144.1	143.2	142.4	141.7	140.9
ΔFe	0	0.10 [0.129(3)]	0.15	0.23	0.29	0.33	0.36	0.40
θ	89.0	87.0 [89.99(3)]	84.1	82.2	78.9	74.2	71.3	68.8
θ'	88.0[d]	85.2 [89.40(8)]	82.0	78.7	74.8	71.0	68.0	65.0

[a]This is the "undistorted" minimization of this molecule where θ' was constrained to be near 90° – see the main article for more details. [b]Crystallographic data from $[Fe(bpp)_2]_2[BF_4]_2$, ref. 11. [c]Fixed value. [d]Constrained during the minimization.

Table S8 Computed metric parameters for the energy-minimized geometries of $[Fe(bpp)_2]^{2+}$ along distortion pathway B (Å, °; Figure S15). Experimental crystallographic data are also included in square brackets for comparison, where this is available. The atom numbering in the table is shown in Figure S12. Other details as for Table S7.

θ'	88.0[a,b]	79.3[b]	76.6[b]	71.8[b]	66.8 [67.74(6)][b,c]	63.6[b]	60.6[b]
Fe–*N*1	2.163	2.172	2.176	2.181	2.191 [2.1676(16)]	2.196	2.206
Fe–*N*2	2.198	2.205	2.207	2.215	2.213 [2.2085(18)]	2.251	2.275
Fe–*N*3	2.198	2.193	2.192	2.194	2.191 [2.1808(18)]	2.195	2.201
Fe–*N*4	2.163	2.172	2.175	2.181	2.191 [2.1676(16)]	2.196	2.206
Fe–*N*5	2.198	2.205	2.207	2.215	2.232 [2.2085(18)]	2.252	2.275
Fe–*N*6	2.198	2.193	2.192	2.193	2.191 [2.1808(18)]	2.195	2.201
*N*1–Fe–*N*2	72.9	72.4	72.3	72.1	71.6 [72.05(6)]	71.2	70.6
*N*1–Fe–*N*3	72.9	72.8	72.6	72.4	72.2 [72.29(6)]	72.0	71.6
*N*1–Fe–*N*4	180.0	164.4	163.8	160.8	157.9 [155.65(10)]	157.3	157.0
*N*1–Fe–*N*5	107.1	96.2	95.6	93.3	91.1 [90.24(6)]	90.7	90.7
*N*1–Fe–*N*6	107.1	119.2	120.1	123.1	126.2 [127.92(6)]	127.2	128.2
*N*2–Fe–*N*3	145.8	144.6	144.3	143.7	142.8 [141.63(6)]	142.1	141.2
*N*2–Fe–*N*4	107.1	96.2	95.6	93.3	91.1 [90.24(6)]	90.7	90.7
*N*2–Fe–*N*5	96.8	88.5	86.0	82.6	79.0 [87.83(10)]	75.9	73.1
*N*2–Fe–*N*6	93.1	101.5	103.8	106.9	110.2 [106.03(6)]	113.1	116.0
*N*3–Fe–*N*4	107.1	119.2	120.1	123.0	126.2 [127.92(6)]	127.2	128.1
*N*3–Fe–*N*5	93.1	101.5	103.8	106.9	110.2 [106.03(6)]	113.1	116.0
*N*3–Fe–*N*6	96.8	89.9	88.2	86.3	84.6 [85.24(9)]	83.0	81.6
*N*4–Fe–*N*5	72.9	72.4	72.3	72.1	71.6 [72.05(6)]	71.2	70.6
*N*4–Fe–*N*6	72.9	72.8	72.6	72.4	72.2 [72.29(6)]	72.0	71.6
*N*5–Fe–*N*6	145.8	144.6	144.3	143.7	142.8 [141.63(6)]	142.1	141.2
ΔFe	0	0.17	0.18	0.21	0.24 [0.374(2)]	0.24	0.25
ϕ	180.0	164.4	163.8	160.8	157.9 [155.65(10)]	157.3	157.0
θ	89.0	79.8	77.3	70.8	65.1 [66.24(2)]	59.8	55.3

[a]This is the "undistorted" minimization of this molecule where *θ'* was constrained to be near 90° – see the main article for more details. [b]Constrained during the minimization – see the main article for more details. [c]Crystallographic data from $[Fe(bpp)_2]_2[ClO_4]_2$, ref. 18.

Table S9 Computed metric parameters for the energy-minimized geometries of $[Fe(bpp)_2]^{2+}$ along distortion pathway C (Å, °; Figure S16). Experimental crystallographic data are also included in square brackets for comparison, where this is available. The atom numbering in the table is shown in Figure S12. Other details as for Table S7.

ϕ	180[a]	165[b]	160 [159.6(3)][b,c]	155 [155.97(16)][b,d]	150[b]	145[b]	140[b]
Fe–*N*1	2.163	2.158	2.162 [2.163(8)]	2.169 [2.141(4)]	2.177	2.191	2.193
Fe–*N*2	2.198	2.210	2.209 [2.202(9)]	2.208 [2.185(5)]	2.205	2.193	2.190
Fe–*N*3	2.198	2.209	2.208 [2.172(8)]	2.205 [2.181(4)]	2.200	2.183	2.177
Fe–*N*4	2.163	2.187	2.190 [2.129(7)]	2.198 [2.159(4)]	2.206	2.216	2.232
Fe–*N*5	2.198	2.234	2.251 [2.231(8)]	2.276 [2.231(5)]	2.320	2.367	2.451
Fe–*N*6	2.198	2.161	2.153 [2.180(7)]	2.150 [2.171(5)]	2.140	2.157	2.137
*N*1–Fe–*N*2	72.9	73.0	72.9 [74.6(3)]	72.7 [72.45(16)]	72.5	71.7	71.7
*N*1–Fe–*N*3	72.9	73.1	73.0 [71.7(3)]	72.9 [73.22(16)]	72.6	72.0	72.1
*N*1–Fe–*N*4	180.0	165.0	160.0 [159.6(3)]	155.0 [155.97(16)]	150.0	145.0	140.0
*N*1–Fe–*N*5	107.1	93.6	89.0 [88.1(3)]	84.6 [86.29(16)]	80.4	76.6	73.1
*N*1–Fe–*N*6	107.1	122.2	127.0 [124.7(3)]	131.9 [129.20(16)]	136.7	141.5	145.9
*N*2–Fe–*N*3	145.8	146.1	145.9 [145.3(3)]	145.5 [142.24(17)]	144.9	140.9	141.3
*N*2–Fe–*N*4	107.1	105.6	105.1 [112.3(3)]	104.8 [102.44(16)]	104.9	106.0	104.7
*N*2–Fe–*N*5	96.8	90.2	89.6 [92.4(3)]	89.0 [102.14(17)]	88.4	92.4	90.9
*N*2–Fe–*N*6	93.1	99.7	100.2 [100.4(3)]	100.7 [90.41(17)]	101.6	99.2	99.9
*N*3–Fe–*N*4	107.1	106.8	106.9 [102.2(3)]	107.1 [115.31(16)]	106.8	112.0	111.7
*N*3–Fe–*N*5	93.1	90.5	89.9 [94.3(3)]	89.6 [90.82(16)]	88.8	92.9	91.3
*N*3–Fe–*N*6	96.8	99.4	100.1 [92.2(3)]	100.7 [99.32(16)]	101.5	100.2	102.4
*N*4–Fe–*N*5	72.9	71.5	71.0 [72.8(3)]	70.5 [71.62(16)]	69.6	68.6	67.1
*N*4–Fe–*N*6	72.9	72.8	73.0 [74.1(3)]	73.0 [73.31(16)]	73.3	73.4	73.9
*N*5–Fe–*N*6	145.8	144.2	144.0 [146.9(3)]	143.5 [144.51(16)]	142.9	142.0	141.0
ΔFe	0	0.05	0.01 [0.11(2)]	0.03 [0.276(7)]	0.05	0.21	0.19
θ	89.0	89.4	89.4 [87.03(9)]	89.3 [89.92(5)]	89.3	87.9	88.1
θ'	88.0[e]	89.5	89.5 [89.5(4)]	89.2 [89.12(16)]	89.2	87.5	87.4

[a]This is the "undistorted" minimization of this molecule where θ' was constrained to be near 90° – see the main article for more details. [b]Fixed value. [c]Crystallographic data from $[Fe(bpp)_2]_2[Co(C_2B_9H_{11})_2]_2\cdot MeNO_2$, ref. 21. [d]Crystallographic data from $[Fe(bpp)_2]_2I_{0.5}[I_3]_{1.5}$, ref. 21. [e]Constrained during the minimization.

Table S10 Computed metric parameters for the energy-minimized geometries of $[Fe(bpp)_2]^{2+}$ along distortion pathway D (Å, °; Figure S17). Experimental crystallographic data are also included in square brackets for comparison, where this is available. The atom numbering in the table is shown in Figure S12. Other details as for Table S7.

θ'	88.0[a,b]	84.8[b]	81.3 [80.5][b,c]	78.1[b]	75.0[b]	72.2[b]	69.6[b]
Fe–*N*1	2.163	2.165	2.169 [2.127(3)]	2.174	2.176	2.177	2.176
Fe–*N*2	2.198	2.200	2.203 [2.204(3)]	2.206	2.214	2.224	2.237
Fe–*N*3	2.198	2.200	2.201 [2.185(3)]	2.207	2.215	2.224	2.237
Fe–*N*4	2.163	2.165	2.171 [2.129(3)]	2.173	2.176	2.177	2.176
Fe–*N*5	2.198	2.200	2.203 [2.172(3)]	2.207	2.214	2.224	2.237
Fe–*N*6	2.198	2.200	2.201 [2.197(3)]	2.206	2.214	2.224	2.237
*N*1–Fe–*N*2	72.9	72.9	72.7 [73.29(10)]	72.6	72.4	72.1	71.9
*N*1–Fe–*N*3	72.9	72.9	72.8 [73.37(10)]	72.5	72.4	72.1	71.9
*N*1–Fe–*N*4	180.0	180.0	179.9 [177.51(10)]	179.8	179.8	180.0	180.0
*N*1–Fe–*N*5	107.1	107.1	107.4 [106.32(10)]	107.6	107.8	107.9	108.1
*N*1–Fe–*N*6	107.1	107.1	107.3 [107.11(10)]	107.3	107.5	107.9	108.1
*N*2–Fe–*N*3	145.8	145.7	145.5 [146.63(11)]	145.1	144.7	144.3	143.8
*N*2–Fe–*N*4	107.1	107.2	107.4 [109.12(10)]	107.3	107.8	107.9	108.1
*N*2–Fe–*N*5	96.8	90.2	87.1 [85.54(10)]	84.4	81.8	79.4	77.4
*N*2–Fe–*N*6	93.1	99.8	103.1 [104.38(10)]	106.2	109.1	111.8	114.3
*N*3–Fe–*N*4	107.1	107.1	107.1 [104.21(10)]	107.6	107.4	107.9	108.1
*N*3–Fe–*N*5	93.1	99.8	103.1 [102.67(10)]	106.1	109.1	111.8	114.3
*N*3–Fe–*N*6	96.8	90.2	87.1 [86.56(10)]	84.4	81.7	79.4	77.4
*N*4–Fe–*N*5	72.9	72.9	72.7 [73.47(10)]	72.6	72.3	72.1	71.9
*N*4–Fe–*N*6	72.9	72.9	72.7 [73.11(10)]	72.6	72.3	72.1	71.9
*N*5–Fe–*N*6	145.8	145.8	145.4 [146.56(10)]	145.1	144.7	144.3	143.8
ΔFe	0	0	0 [0.03]	0	0	0	0
ϕ	180.0	180.0	179.9 [177.51(10)]	179.8	179.8	180.0	180.0
θ	89.0	85.3	80.3 [76.0]	75.2	69.7	64.1	58.3

[a]This is the "undistorted" minimization of this molecule where *θ'* was constrained to be near 90° – see the main article for more details. [b]Constrained during the minimization – see the main article for more details. [c]Crystallographic data from $[Fe(bpp)_2]_2[Ni(mnt)_2]_2{\cdot}MeNO_2$ at 200 K, ref. 38.

Table S11 Computed energies of the other minimized high-spin complexes along distortion pathway A (Figures S18-S23). See page S8 for definitions of ϕ and θ'.

$[Fe(bpp^{NO_2})_2]^{2+}$								
ϕ / deg	180.0[a]	173.0	165[b]	160[b]	155[b]	150[b]	145[b]	140[b]
θ' / deg	88.2[a,c]	85.0	81.7	78.6	75.0	71.0	68.1	65.4
E / Ha	–3068.81149	–3068.81148	–3068.81144	–3068.81123	–3068.81071	–3068.80965	–3068.80789	–3068.80527
$[Fe(bpp^{CN})_2]^{2+}$								
ϕ / deg	180.0[a]	175.2	165[b]	160[b]	155[b]	150[b]	145[b]	140[b]
θ' / deg	88.2[a,c]	85.6	81.6	78.5	75.0	71.0	68.2	65.5
E / Ha	–2844.29171	–2844.29177	–2844.29169	–2844.29149	–2844.29093	–2844.28991	–2844.28814	–2844.28548
$[Fe(bpp^{NMe_2})_2]^{2+}$								
ϕ / deg	179.9[a]	174.2	165[b]	160[b]	155[b]	150[b]	145[b]	140[b]
θ' / deg	89.7[a,c]	88.5	85.5	82.1	78.4	74.8	72.5	70.4
E / Ha	–2927.85514	–2927.85521	–2927.85532	–2927.85510	–2927.85446	–2927.85358	–2927.85198	–2927.84970
$[Fe(bpp^{SMe})_2]^{2+}$								
ϕ / deg	180.0[a]	174.5	165[b]	160[b]	155[b]	150[b]	145[b]	140[b]
θ' / deg	88.1[a,c]	88.6	83.6	80.2	76.3	73.6	71.9	68.6
E / Ha	–3534.90455	–3534.90498	–3534.90503	–3534.90470	–3534.90436	–3534.90346	–3534.90184	–3534.89966
$[Fe(bpp^{SPh})_2]^{2+}$								
ϕ / deg	178.4[a]	177.1	165[b]	160[b]	155[b]	150[b]	145[b]	140[b]
θ' / deg	88.2[a,c]	90.0	84.2	80.3	76.6	74.2	72.5	68.9
E / Ha	–3918.33485	–3918.33527	–3918.33552	–3918.33518	–3918.33500	–3918.33411	–3918.33259	–3918.33047
$[FeL_2]^{2+}$								
ϕ / deg	177.2[a]	176.5	165[b]	160[b]	155[b]	150[b]	145[b]	140[b]
θ' / deg	88.0[a,c]	89.5	84.1	80.7	77.1	74.3	71.8	69.7
E / Ha	–4376.40742	-4376.40785	–4376.40815	–4376.40796	–4376.40764	–4376.40685	–4376.40540	–4376.40324

[a]This is the "undistorted" minimization of this molecule where θ' was constrained to be near, but not exactly, 90° – see the text for more details. [b]Fixed value.
[c]Constrained during the minimization.

Table S12 Computed metric parameters for the energy-minimized geometries of $[Fe(bpp^{NO2})_2]^{2+}$ (Å, °; Figure S18). The atom numbering in the table is shown in Figure S12. Other details as for Table S7.

ϕ	180[a]	173.0	165[b]	160[b]	155[b]	150[b]	145[b]	140[b]
Fe–*N*1	2.162	2.169	2.172	2.175	2.179	2.182	2.186	2.192
Fe–*N*2	2.203	2.202	2.207	2.210	2.219	2.231	2.251	2.274
Fe–*N*3	2.203	2.200	2.192	2.188	2.178	2.172	2.163	2.154
Fe–*N*4	2.170	2.168	2.172	2.175	2.179	2.182	2.186	2.192
Fe–*N*5	2.198	2.204	2.207	2.210	2.220	2.230	2.251	2.274
Fe–*N*6	2.198	2.199	2.192	2.188	2.178	2.173	2.163	2.154
*N*1–Fe–*N*2	73.0	72.8	72.5	72.2	71.8	71.4	70.8	70.2
*N*1–Fe–*N*3	73.0	72.9	72.9	72.9	72.9	73.0	73.4	73.7
*N*1–Fe–*N*4	180.0	173.0	165.0	160.0	155.0	150.0	145.0	140.0
*N*1–Fe–*N*5	107.2	102.1	96.8	93.6	90.3	87.1	84.0	81.3
*N*1–Fe–*N*6	107.2	112.4	118.5	122.4	126.5	130.5	134.0	137.4
*N*2–Fe–*N*3	146.0	145.5	144.8	144.0	143.2	142.4	141.9	141.1
*N*2–Fe–*N*4	107.0	102.2	96.8	93.5	90.3	87.1	84.0	81.3
*N*2–Fe–*N*5	96.7	91.1	90.7	90.5	89.8	88.9	88.0	88.5
*N*2–Fe–*N*6	93.3	98.9	99.5	100.0	100.6	101.2	100.9	100.0
*N*3–Fe–*N*4	107.0	112.3	118.5	122.5	126.5	130.5	134.0	137.4
*N*3–Fe–*N*5	93.3	98.9	99.5	100.0	100.6	101.2	100.9	100.0
*N*3–Fe–*N*6	96.7	91.3	91.4	91.4	91.9	92.6	94.6	96.9
*N*4–Fe–*N*5	72.8	72.8	72.5	72.2	71.8	71.4	70.8	70.2
*N*4–Fe–*N*6	72.8	72.9	72.9	72.9	72.9	73.0	73.4	73.7
*N*5–Fe–*N*6	145.5	145.5	144.8	144.0	143.2	142.4	141.9	141.1
ΔFe	0	0.08	0.17	0.23	0.28	0.32	0.35	0.38
θ	89.3	86.0	84.0	81.9	78.5	74.0	71.3	68.7
θ'	88.2[a]	85.0	81.7	78.6	75.0	71.0	68.1	65.4

[a]This is the "undistorted" minimization of this molecule where θ' was constrained to be near 90° – see the main article for more details. [b]Fixed value.

Table S13 Computed metric parameters for the energy-minimized geometries of $[Fe(bpp^{CN})_2]^{2+}$ (Å, °; Figure S19). The atom numbering in the table is shown in Figure S12. Other details as for Table S7.

ϕ	180[a]	175.2	165[b]	160[b]	155[b]	150[b]	145[b]	140[b]
Fe–*N*1	2.167	2.167	2.172	2.175	2.178	2.182	2.185	2.191
Fe–*N*2	2.199	2.200	2.204	2.210	2.217	2.230	2.249	2.274
Fe–*N*3	2.199	2.201	2.191	2.186	2.178	2.172	2.162	2.152
Fe–*N*4	2.164	2.170	2.172	2.175	2.178	2.182	2.185	2.191
Fe–*N*5	2.199	2.196	2.204	2.210	2.217	2.230	2.249	2.274
Fe–*N*6	2.199	2.199	2.191	2.186	2.178	2.172	2.162	2.152
*N*1–Fe–*N*2	72.8	72.8	72.4	72.2	71.8	71.4	70.8	70.2
*N*1–Fe–*N*3	72.8	72.8	72.8	72.8	72.9	73.0	73.4	73.7
*N*1–Fe–*N*4	180.0	175.2	165.0	160.0	155.0	150.0	145.0	140.0
*N*1–Fe–*N*5	107.1	103.8	96.8	93.6	90.3	87.1	84.0	81.3
*N*1–Fe–*N*6	107.1	110.9	118.6	122.5	126.5	130.5	134.0	137.3
*N*2–Fe–*N*3	145.6	145.5	144.6	143.9	143.1	142.4	141.9	141.1
*N*2–Fe–*N*4	107.2	103.9	96.8	93.6	90.3	87.1	84.0	81.3
*N*2–Fe–*N*5	96.6	91.9	91.1	90.6	89.8	88.7	88.0	88.4
*N*2–Fe–*N*6	93.4	98.7	99.5	100.1	100.6	101.3	100.9	99.9
*N*3–Fe–*N*4	107.2	110.6	118.6	122.5	126.5	130.5	134.0	137.3
*N*3–Fe–*N*5	93.4	98.7	99.5	100.1	100.6	101.3	100.9	99.9
*N*3–Fe–*N*6	96.6	91.1	91.2	91.3	92.0	92.7	94.6	95.9
*N*4–Fe–*N*5	72.9	72.7	72.4	72.2	71.8	71.4	70.8	70.2
*N*4–Fe–*N*6	72.9	72.7	72.8	72.8	72.9	73.0	73.4	73.7
*N*5–Fe–*N*6	145.8	145.3	144.6	143.9	143.1	142.4	141.9	141.1
ΔFe	0	0.08	0.17	0.22	0.27	0.31	0.34	0.38
θ	89.2	87.0	84.0	81.8	78.4	74.0	71.3	68.8
θ'	88.2[a]	85.6	81.6	78.5	75.0	71.0	68.2	65.5

[a]This is the "undistorted" minimization of this molecule where θ' was constrained to be near 90° – see the main article for more details. [b]Fixed value.

Table S14 Computed metric parameters for the energy-minimized geometries of $[Fe(bpp^{NMe2})_2]^{2+}$ (Å, °; Figure S20). Experimental crystallographic data are included in square brackets for comparison, where this is available. The atom numbering in the table is shown in Figure S12. Other details as for Table S7.

ϕ	180[a]	174.2	165[b,c]	160[b]	155[b]	150[b]	145[b]	140[b]
Fe–*N*1	2.154	2.155	2.154 [2.119(2)]	2.157	2.158	2.161	2.163	2.169
Fe–*N*2	2.216	2.216	2.217 [2.191(2)]	2.221	2.228	2.242	2.262	2.287
Fe–*N*3	2.216	2.208	2.206 [2.201(2)]	2.202	2.194	2.185	2.174	2.165
Fe–*N*4	2.154	2.155	2.154 [2.118(2)]	2.158	2.158	2.161	2.164	2.167
Fe–*N*5	2.216	2.208	2.218 [2.171(2)]	2.223	2.229	2.242	2.264	2.284
Fe–*N*6	2.216	2.216	2.206 [2.203(2)]	2.201	2.194	2.185	2.174	2.166
*N*1–Fe–*N*2	72.2	72.2	71.8 [72.97(8)]	71.6	71.4	70.9	70.5	69.8
*N*1–Fe–*N*3	72.2	72.1	72.4 [73.22(8)]	72.4	72.5	72.7	73.0	73.2
*N*1–Fe–*N*4	179.9	174.2	165.0 [164.33(8)]	160.0	155.0	150.0	145.0	140.0
*N*1–Fe–*N*5	107.9	103.9	98.0 [103.19(8)]	94.8	91.6	88.5	85.6	83.4
*N*1–Fe–*N*6	107.7	112.1	118.6 [111.98(8)]	122.6	126.4	130.0	133.3	136.0
*N*2–Fe–*N*3	144.3	143.9	143.3 [146.19(8)]	142.7	142.0	141.5	141.1	140.1
*N*2–Fe–*N*4	107.8	112.1	98.0 [91.93(8)]	94.9	91.7	88.5	85.6	83.2
*N*2–Fe–*N*5	95.6	95.8	96.9 [94.53(8)]	96.2	95.5	93.9	93.7	95.1
*N*2–Fe–*N*6	95.1	92.6	95.8 [95.37(8)]	96.4	96.9	97.4	96.5	94.7
*N*3–Fe–*N*4	107.9	103.9	118.7 [121.77(8)]	122.5	126.3	130.0	133.2	136.2
*N*3–Fe–*N*5	95.1	97.7	95.8 [93.04(8)]	96.4	96.9	97.4	96.5	94.8
*N*3–Fe–*N*6	95.6	95.8	94.3 [97.18(8)]	94.5	95.0	96.3	98.8	101.8
*N*4–Fe–*N*5	72.2	72.1	71.8 [73.22(8)]	71.6	71.3	70.9	70.4	69.9
*N*4–Fe–*N*6	72.2	72.2	72.4 [72.79(8)]	72.4	72.5	72.7	72.9	73.2
*N*5–Fe–*N*6	144.3	143.9	143.3 [144.83(8)]	142.6	142.0	141.5	141.0	140.2
ΔFe	0	0.15	0.22 [0.128(3)]	0.27	0.32	0.34	0.36	0.40
θ	89.3	89.1	86.8 [87.03(2)]	84.4	80.9	76.5	74.1	72.3
θ’	89.7[a]	88.5	85.5 [89.20(6)]	82.1	78.4	74.8	72.5	70.4

[a]This is the “undistorted” minimization of this molecule where θ’ was constrained to be near 90° – see the main article for more details. [b]Fixed value.
[c]Crystallographic data from $[Fe(bpp^{NMe2})_2]_2[ClO_4]_2 \cdot xH_2O$, ref. 19.

Table S15 Computed metric parameters for the energy-minimized geometries of $[Fe(bpp^{SMe})_2]^{2+}$ (Å, °; Figure S21). Experimental crystallographic data are included in square brackets for comparison, where this is available. The atom numbering in the table is shown in Figure S12. Other details as for Table S7.

ϕ	180[a]	174.5[b]	165[b,c]	160[c]	155[c]	150[c]	145[c]	140[c]
Fe–*N*1	2.149	2.175 [2.115(4)]	2.165 [2.126(4)]	2.169	2.170	2.173	2.179	2.181
Fe–*N*2	2.207	2.220 [2.185(5)]	2.206 [2.182(5)]	2.212	2.220	2.236	2.260	2.280
Fe–*N*3	2.201	2.187 [2.172(5)]	2.210 [2.168(5)]	2.202	2.192	2.185	2.173	2.169
Fe–*N*4	2.151	2.152 [2.115(4)]	2.165 [2.120(4)]	2.168	2.169	2.173	2.176	2.181
Fe–*N*5	2.206	2.214 [2.185(5)]	2.206 [2.165(5)]	2.211	2.218	2.233	2.249	2.276
Fe–*N*6	2.199	2.203 [2.172(4)]	2.209 [2.172(5)]	2.201	2.192	2.185	2.176	2.169
*N*1–Fe–*N*2	72.8	71.9 [73.17(16)]	72.0 [73.44(17)]	71.7	71.4	71.0	70.4	69.9
*N*1–Fe–*N*3	73.0	72.1 [73.61(17)]	72.2 [72.76(18)]	72.3	72.3	72.5	72.8	72.9
*N*1–Fe–*N*4	180.0	174.5 [177.7(3)]	165.0 [168.89(19)]	160.0	155.0	150.0	145.0	140.0
*N*1–Fe–*N*5	107.2	103.0 [105.13(17)]	97.8 [101.06(17)]	95.2	92.0	88.8	86.3	83.1
*N*1–Fe–*N*6	107.0	112.3 [108.06(17)]	119.2 [112.82(18)]	122.5	126.5	130.1	133.0	136.8
*N*2–Fe–*N*3	145.8	143.8 [146.78(16)]	142.9 [145.96(18)]	142.4	141.7	141.2	140.7	139.8
*N*2–Fe–*N*4	107.2	110.9 [105.13(17)]	98.0 [97.03(18)]	94.1	90.8	87.9	85.0	82.8
*N*2–Fe–*N*5	96.8	92.8 [92.2(2)]	97.1 [94.8(2)]	95.8	94.4	93.8	94.3	94.2
*N*2–Fe–*N*6	93.2	91.9 [95.88(18)]	96.9 [96.79(19)]	97.8	98.6	98.1	96.6	96.0
*N*3–Fe–*N*4	107.0	105.3 [108.06(17)]	119.0 [117.01(18)]	123.4	127.5	130.9	134.2	137.0
*N*3–Fe–*N*5	93.2	98.3 [95.88(18)]	97.1 [95.50(18)]	97.7	98.6	98.2	96.7	96.1
*N*3–Fe–*N*6	96.7	98.7 [94.7(3)]	92.1 [92.53(18)]	92.5	93.2	95.3	98.4	100.7
*N*4–Fe–*N*5	72.8	72.4 [73.17(16)]	71.9 [73.60(17)]	71.8	71.5	71.1	70.6	70.0
*N*4–Fe–*N*6	73.0	72.7 [73.61(17)]	72.2 [73.36(17)]	72.3	72.3	72.5	72.7	72.9
*N*5–Fe–*N*6	145.7	144.1 [146.78(16)]	142.9 [146.05(17)]	142.2	141.5	141.1	140.5	139.8
ΔFe	0	0.14 [0.013(5)]	0.26 [0.154(8)]	0.29	0.33	0.36	0.38	0.42
θ	89.0	88.9 [89.51(4)]	85.1 [87.14(4)]	82.5	76.5	74.4	73.2	68.3
θ’	88.1[a]	88.6 [88.7(1)]	83.6 [86.4(1)]	80.2	76.3	73.6	71.9	68.6

[a]This is the "undistorted" minimization of this molecule where *θ’* was constrained to be near 90° – see the main article for more details. [b]Crystallographic data from molecules A and B in phase 1 of $[Fe(bpp^{SMe})_2]_2[ClO_4]_2$, ref. 43. [c]Fixed value.

Table S16 Computed metric parameters for the energy-minimized geometries of $[Fe(bpp^{SPh})_2]^{2+}$ (Å, °; Figure S22). The atom numbering in the table is shown in Figure S12. Other details as for Table S7.

ϕ	178.4[a]	177.1	165[b]	160[b]	155[b]	150[b]	145[b]	140[b]
Fe–*N*1	2.147	2.171	2.162	2.168	2.168	2.169	2.175	2.177
Fe–*N*2	2.205	2.221	2.209	2.215	2.223	2.240	2.263	2.279
Fe–*N*3	2.205	2.189	2.210	2.203	2.195	2.184	2.177	2.168
Fe–*N*4	2.147	2.147	2.162	2.167	2.167	2.169	2.173	2.178
Fe–*N*5	2.204	2.214	2.209	2.213	2.220	2.238	2.251	2.284
Fe–*N*6	2.205	2.213	2.210	2.204	2.196	2.184	2.177	2.169
*N*1–Fe–*N*2	72.9	72.0	72.0	71.6	71.4	71.0	70.4	70.0
*N*1–Fe–*N*3	72.9	72.2	72.4	72.3	72.4	72.7	72.9	73.1
*N*1–Fe–*N*4	178.4	177.1	165.0	160.0	155.0	150.0	145.0	140.0
*N*1–Fe–*N*5	107.3	105.2	97.9	95.3	92.3	89.0	86.5	82.7
*N*1–Fe–*N*6	106.8	109.0	119.0	122.5	126.1	129.7	132.7	136.8
*N*2–Fe–*N*3	145.9	144.2	143.1	142.4	141.9	141.5	140.9	140.1
*N*2–Fe–*N*4	105.5	105.9	98.0	94.1	90.6	87.8	85.0	83.0
*N*2–Fe–*N*5	96.6	91.7	97.4	95.7	94.5	94.0	94.6	93.7
*N*2–Fe–*N*6	93.3	91.8	96.4	97.8	98.3	97.7	96.1	96.1
*N*3–Fe–*N*4	108.6	109.9	118.9	123.4	127.5	130.7	134.0	136.6
*N*3–Fe–*N*5	93.3	98.8	96.6	97.7	98.4	97.8	96.3	96.0
*N*3–Fe–*N*6	96.6	98.6	92.5	92.3	93.4	95.6	98.8	100.7
*N*4–Fe–*N*5	72.9	72.7	71.9	71.7	71.5	71.0	70.6	69.9
*N*4–Fe–*N*6	72.9	72.8	72.4	72.3	72.4	72.7	72.8	73.1
*N*5–Fe–*N*6	145.9	144.9	143.1	142.2	141.6	141.2	140.7	140.1
ΔFe	0	0.10	0.25	0.29	0.33	0.35	0.38	0.41
θ	88.9	89.7	85.5	82.9	76.7	75.0	73.9	68.3
θ'	88.2[a]	90.0	84.2	80.3	76.6	74.2	72.5	68.9

[a]This is the "undistorted" minimization of this molecule where θ' was constrained to be near 90° – see the main article for more details. [b]Fixed value.

Table S17 Computed metric parameters for the energy-minimized geometries of $[FeL_2]^{2+}$ (Å, °; Figure S23). Experimental crystallographic data are included in square brackets for comparison, where this is available. The atom numbering in the table is shown in Figure S12. Other details as for Table S7.

ϕ	177.2[a]	176.5	165[b]	160[b]	155[b]	150[b]	145[b,c]	140[b]
Fe–*N*1	2.145	2.168	2.161	2.163	2.165	2.168	2.171 [2.171(4)]	2.174
Fe–*N*2	2.206	2.224	2.211	2.216	2.222	2.242	2.262 [2.215(4)]	2.279
Fe–*N*3	2.205	2.192	2.208	2.203	2.196	2.186	2.177 [2.180(4)]	2.167
Fe–*N*4	2.145	2.147	2.161	2.162	2.163	2.166	2.170 [2.171(4)]	2.175
Fe–*N*5	2.204	2.216	2.209	2.215	2.224	2.234	2.255 [2.215(4)]	2.289
Fe–*N*6	2.206	2.210	2.210	2.202	2.195	2.188	2.176 [2.180(4)]	2.168
*N*1–Fe–*N*2	73.0	72.2	72.0	71.7	71.5	71.0	70.5 [71.33(15)]	70.0
*N*1–Fe–*N*3	73.0	72.1	72.4	72.5	72.5	72.7	73.0 [71.67(15)]	73.1
*N*1–Fe–*N*4	177.2	176.5	165.0	160.0	155.0	150.0	145.0 [143.0(2)]	140.0
*N*1–Fe–*N*5	109.5	104.4	97.6	95.4	91.8	89.6	86.0 [84.07(15)]	82.4
*N*1–Fe–*N*6	104.6	110.5	119.0	122.1	126.4	129.1	133.0 [137.12(15)]	136.7
*N*2–Fe–*N*3	145.9	144.2	143.1	142.8	142.1	141.7	141.2 [138.81(15)]	140.2
*N*2–Fe–*N*4	105.6	109.6	98.1	93.9	91.1	87.0	84.8 [84.07(15)]	83.5
*N*2–Fe–*N*5	96.6	92.3	97.1	95.6	94.7	93.5	93.2 [96.4(2)]	94.2
*N*2–Fe–*N*6	93.2	92.1	96.4	97.6	98.0	97.8	97.0 [97.21(15)]	95.4
*N*3–Fe–*N*4	108.5	106.3	118.7	123.3	126.9	131.2	133.9 [137.12(15)]	135.9
*N*3–Fe–*N*5	93.2	98.7	96.8	97.4	97.8	98.0	97.0 [97.21(15)]	95.4
*N*3–Fe–*N*6	96.7	98.2	92.6	92.9	93.8	95.7	98.2 [97.6(2)]	101.2
*N*4–Fe–*N*5	73.0	72.6	72.0	71.8	71.5	71.2	70.6 [71.33(15)]	69.9
*N*4–Fe–*N*6	72.9	72.7	72.4	72.5	72.5	72.6	72.9 [71.67(15)]	73.2
*N*5–Fe–*N*6	145.9	144.4	143.2	142.5	141.9	141.3	140.9 [138.81(15)]	140.5
ΔFe	0.03	0.14	0.25	0.29	0.33	0.35	0.37 [0.480(4)]	0.40
θ	89.0	89.6	85.1	82.0	77.3	74.6	71.6 [60.25(5)]	68.9
θ'	88.0[a]	89.5	84.1	80.7	77.1	74.3	71.8 [66.45(15)]	69.7

[a]This is the “undistorted” minimization of this molecule where θ' was constrained to be near 90° – see the main article for more details. [b]Fixed value.
[c]Crystallographic data from $[FeL_2]_2[ClO_4]_2$, Table S2.

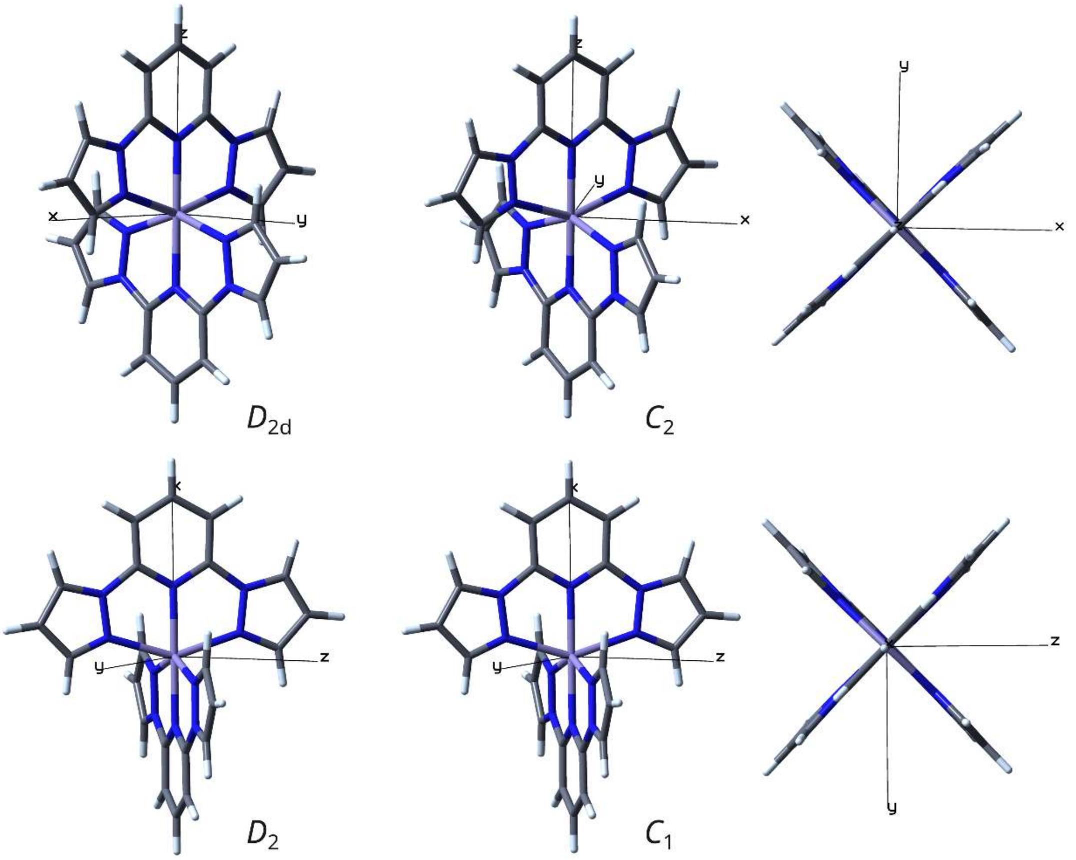

Figure S24 Molecular structures of the high-spin DFT-calculated cation $[Fe(bpp)_2]^{2+}$ with point group symmetries ranging from C_1 to D_{2d}. The directions of the Cartesian axes for each point group are indicated. The natural atomic *d*-orbitals do not line up with the Cartesian axes for the most part, making orbital assignment in terms of ligand field theory difficult.

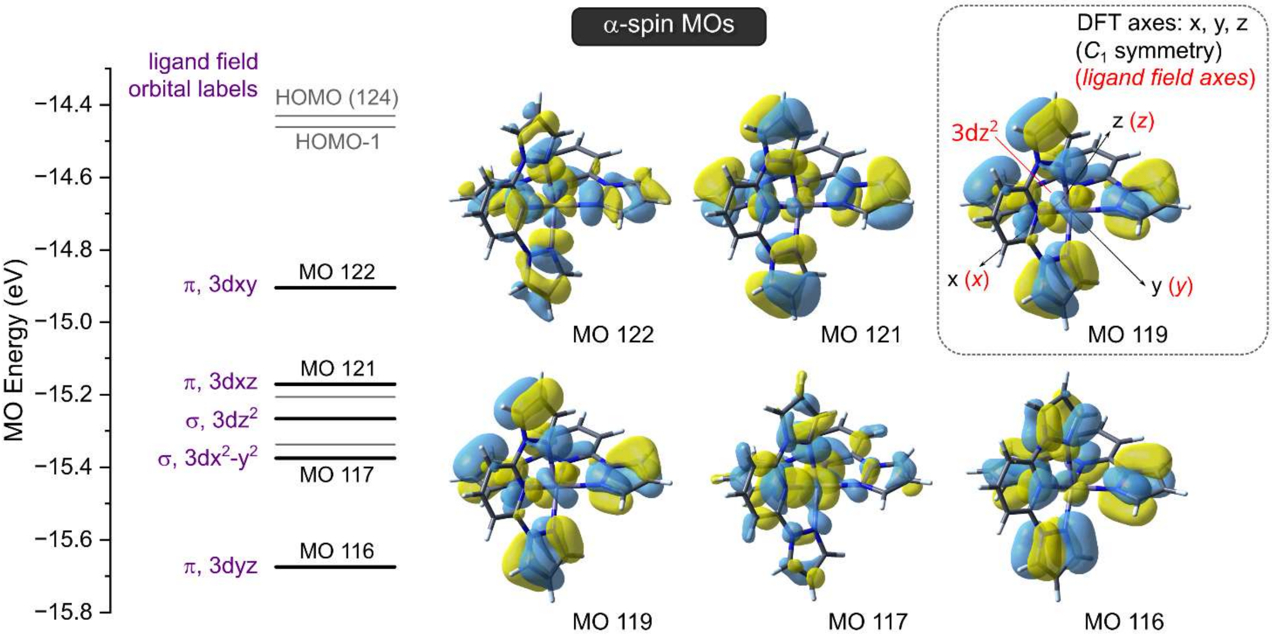

Figure S25 Energy level scheme (drawn to scale) for the top 9 singly occupied molecular orbitals (α-spin) of high-spin $[Fe(bpp)_2]^{2+}$ for the structure with $\phi = 140°$. The MOs with substantial *d*-orbital character are indicated, while the inset shows the Cartesian axis convention used to assign the *d*-orbital labels. The structure has C_1 symmetry (Figure S24). Note that the distortion of the molecular geometry places the center of gravity for the Cartesian axis system at a position significantly offset from the Fe(II) ion.

The corresponding data for the undistorted minimization of $[Fe(bpp)_2]^{2+}$ are shown in Figure 6 (main article).

Table S18 Atomic coordinates for freely minimized $[FeL_2]^{2+}$ in its high- and low-spin states.

$[FeL_2]^{2+}$, high-spin; ϕ = 163.1° (minimized), θ' = 83.2° (minimized)

Fe	0.000000	0.290900	0.000200
N	-2.017800	0.605500	-0.649200
C	-3.019200	-0.047800	-0.064600
C	-4.348100	0.213200	-0.313100
C	-4.649200	1.221200	-1.239200
C	-3.596700	1.912200	-1.866900
C	-2.311200	1.561100	-1.530500
N	-2.575700	-1.042800	0.827300
N	-1.240600	-1.225700	0.982000
C	-1.115800	-2.240500	1.818500
H	-0.133600	-2.584300	2.104300
C	-2.373800	-2.729000	2.218400
H	-2.583100	-3.545300	2.888700
C	-3.282100	-1.943700	1.561200
H	-4.359700	-1.963100	1.556000
N	-1.160100	2.163400	-2.078600
N	0.052200	1.740000	-1.646800
C	0.939300	2.439000	-2.333000
H	1.993800	2.282800	-2.164000
C	0.309100	3.326700	-3.222600
H	0.770500	4.017100	-3.908000
C	-1.032100	3.120400	-3.033400
H	-1.887400	3.574700	-3.505900
N	2.017900	0.606100	0.648900
C	3.019200	-0.047700	0.064800
C	4.348000	0.213100	0.313300
C	4.649300	1.221600	1.238800
C	3.596900	1.913000	1.866200
C	2.311300	1.562100	1.529900
N	2.575500	-1.043200	-0.826500
N	1.240400	-1.226300	-0.980700
C	1.115400	-2.241500	-1.816700
H	0.133200	-2.585500	-2.101900
C	2.373300	-2.730100	-2.216800
H	2.582500	-3.546700	-2.886800
C	3.281700	-1.944400	-1.560200
H	4.359300	-1.963600	-1.555500
N	1.160300	2.164800	2.077500
N	-0.052000	1.740800	1.646400
C	-0.939100	2.440400	2.332200
H	-1.993600	2.283900	2.163700
C	-0.308700	3.329000	3.220800
H	-0.770000	4.020100	3.905600
C	1.032400	3.122700	3.031400
H	1.887800	3.577700	3.503200
H	5.143800	-0.331400	-0.175300
H	3.805400	2.693300	2.585600
H	-5.143800	-0.330900	0.175800
H	-3.805100	2.692200	-2.586600
S	-6.280300	1.678200	-1.666300
S	6.280400	1.678600	1.665800

C	-7.263000	0.576400	-0.675800
C	-7.695800	0.975700	0.573900
C	-7.608100	-0.684200	-1.189300
C	-8.474000	0.106500	1.340100
H	-7.448100	1.958000	0.956400
C	-8.382300	-1.549000	-0.435400
H	-7.283000	-0.961600	-2.183000
C	-8.821800	-1.147600	0.857600
H	-8.822800	0.431500	2.310800
C	7.263000	0.576500	0.675600
C	7.695600	0.975400	-0.574400
C	7.608300	-0.683900	1.189400
C	8.473700	0.105900	-1.340500
H	7.447800	1.957600	-0.957200
C	8.382500	-1.548800	0.435700
H	7.283500	-0.961000	2.183300
C	8.821700	-1.147900	-0.857500
H	8.822300	0.430700	-2.311300
O	-8.779400	-2.768100	-0.820800
O	-9.565600	-2.045400	1.505400
O	9.565500	-2.045900	-1.505200
O	8.779800	-2.767800	0.821400
C	10.128100	-1.690200	-2.758200
H	10.717100	-2.548800	-3.069800
H	9.347800	-1.494800	-3.501300
H	10.779400	-0.816100	-2.662500
C	8.485800	-3.187600	2.140800
H	8.941500	-4.168700	2.247700
H	8.917400	-2.503500	2.878700
H	7.404600	-3.271200	2.300300
C	- 10.128500	-1.689300	2.758200
H	- 10.717600	-2.547800	3.070000
H	-9.348400	-1.493600	3.501400
H	- 10.779800	-0.815300	2.662100
C	-8.485300	-3.188300	-2.139900
H	-8.940900	-4.169500	-2.246500
H	-8.916800	-2.504500	-2.878100
H	-7.404000	-3.272000	-2.299400

$[FeL_2]^{2+}$, low-spin; ϕ = 179.7° (minimized), θ' = 90.0° (minimized)

Fe	0.000000	1.049400	0.000000
N	1.845800	1.054600	0.522700
C	2.712800	0.248100	-0.084000
C	4.051400	0.214900	0.232800
C	4.496300	1.079300	1.246000
C	3.577700	1.932700	1.888400
C	2.265500	1.877600	1.482800
N	2.075200	-0.531000	-1.063400
N	0.735800	-0.344000	-1.240300
C	0.383300	-1.176200	-2.201000
H	-0.644100	-1.216200	-2.527900
C	1.490300	-1.915900	-2.661300
H	1.505300	-2.665300	-3.434200
C	2.550300	-1.478300	-1.914100

H	3.590100	-1.762800	-1.920100
N	1.202200	2.645300	1.987000
N	-0.030600	2.444000	1.438400
C	-0.841900	3.270500	2.069500
H	-1.888700	3.299600	1.809800
C	-0.146900	4.021000	3.037800
H	-0.545500	4.769400	3.701300
C	1.151500	3.596300	2.956600
H	2.029600	3.893800	3.506200
N	-1.845800	1.054600	-0.522600
C	-2.713000	0.248600	0.084400
C	-4.051500	0.215300	-0.232400
C	-4.496300	1.079300	-1.246000
C	-3.577600	1.932200	-1.888900
C	-2.265400	1.877200	-1.483200
N	-2.075500	-0.530100	1.064300
N	-0.736000	-0.343300	1.241000
C	-0.383700	-1.175000	2.202200
H	0.643700	-1.215000	2.529100
C	-1.490800	-1.914300	2.662900
H	-1.506000	-2.663300	3.436200
C	-2.550800	-1.477000	1.915400
H	-3.590600	-1.761300	1.921500
N	-1.202000	2.644500	-1.987700
N	0.030700	2.443200	-1.439100
C	0.842200	3.269300	-2.070600
H	1.889000	3.298400	-1.810900
C	0.147300	4.019400	-3.039200
H	0.546000	4.767500	-3.703100
C	-1.151200	3.595000	-2.957800
H	-2.029300	3.892300	-3.507600
H	-4.746900	-0.441900	0.271500
H	-3.903800	2.606100	-2.670000
H	4.746700	-0.442700	-0.270800
H	3.904000	2.606900	2.669200
S	6.164900	1.167800	1.764500
S	-6.164900	1.167600	-1.764700
C	6.959200	-0.034600	0.723900
C	7.512500	0.355700	-0.480600
C	7.038300	-1.369100	1.153900
C	8.143500	-0.594200	-1.285300
H	7.471700	1.390400	-0.797500
C	7.667000	-2.314300	0.362400
H	6.626200	-1.643900	2.115600
C	8.227700	-1.920600	-0.885100
H	8.588100	-0.279300	-2.219500
C	-6.959300	-0.034700	-0.724000
C	-7.512900	0.355800	0.480200
C	-7.038000	-1.369300	-1.153800
C	-8.143900	-0.594000	1.284900
H	-7.472400	1.390500	0.796800
C	-7.666700	-2.314500	-0.362200
H	-6.625600	-1.644200	-2.115300
C	-8.227800	-1.920500	0.885000

H	-8.588900	-0.279100	2.219000
O	7.811700	-3.609500	0.670300
O	8.814900	-2.899300	-1.575100
O	-8.815000	-2.899200	1.575000
O	-7.811100	-3.609700	-0.669900
C	-9.486500	-2.575000	2.782300
H	-9.913300	-3.508200	3.140500
H	-8.789200	-2.182700	3.529900
H	-10.288100	-1.851800	2.604000
C	-7.394100	-4.050100	-1.948400
H	-7.655300	-5.104100	-1.998300
H	-7.916500	-3.507700	-2.743300
H	-6.310700	-3.939400	-2.074100
C	9.486000	-2.575200	-2.782600
H	9.912900	-3.508400	-3.140700
H	8.788500	-2.183200	-3.530100
H	10.287500	-1.851800	-2.604600
C	7.395100	-4.049600	1.949000
H	7.656400	-5.103700	1.999000
H	7.917500	-3.507000	2.743600
H	6.311700	-3.939100	2.074900

Table S19 Atomic coordinates for DFT-minimized $[Fe(bpp)_2]^{2+}$ along the different distortion pathways.

$[Fe(bpp)_2]^{2+}$, undistorted – ϕ = 180° (minimized), θ' = 88.0° (constrained)

Fe	0.000023	-0.000028	-0.000018
N	0.000007	0.000016	2.162744
C	0.798839	0.827258	2.828682
C	0.840795	0.870256	4.211964
C	-0.000041	0.000071	4.894937
H	-0.000067	0.000097	5.978052
C	-0.840843	-0.870149	4.211968
H	-1.496668	-1.542219	4.746869
C	-0.798844	-0.827206	2.828686
N	1.590265	1.632995	1.990666
N	1.511275	1.459353	0.645550
C	2.361196	2.326717	0.123911
H	2.484637	2.382753	-0.946747
C	3.003817	3.075885	1.125244
H	3.741330	3.850825	1.001203
C	2.489367	2.605078	2.302645
H	2.694034	2.889840	3.321666
N	-1.590245	-1.632971	1.990685
N	-1.511209	-1.459412	0.645565
C	-2.361111	-2.326811	0.123944
H	-2.484516	-2.382901	-0.946716
C	-3.003765	-3.075922	1.125284
H	-3.741273	-3.850874	1.001264
C	-2.489359	-2.605037	2.302681
H	-2.694072	-2.889741	3.321708
N	0.000027	0.000015	-2.162716
C	-0.798822	0.827265	-2.828637
C	-0.840776	0.870266	-4.211919
H	-1.496587	1.542355	-4.746816
C	0.000057	0.000082	-4.894896
H	0.000069	0.000109	-5.978011
C	0.840873	-0.870138	-4.211943
C	0.798880	-0.827211	-2.828662
N	-1.590263	1.632992	-1.990633
N	-1.511284	1.459404	-0.645504
C	-2.361235	2.326758	-0.123904
H	-2.484677	2.382832	0.946751
C	-3.003882	3.075866	-1.125265
H	-3.741419	3.850791	-1.001255
C	-2.489419	2.605020	-2.302648
H	-2.694103	2.889732	-3.321678
N	1.590282	-1.632987	-1.990677
N	1.511291	-1.459436	-0.645549
C	2.361174	-2.326876	-0.123976
H	2.484613	-2.382979	0.946680
C	3.003755	-3.076016	-1.125348
H	3.741231	-3.851002	-1.001358
C	2.489343	-2.605092	-2.302717
H	2.694008	-2.889796	-3.321753
H	1.496694	-1.542199	-4.746860
H	1.496602	1.542347	4.746860

$[Fe(bpp)_2]^{2+}$, pathway A; ϕ = 172.6° (minimized), θ' = 85.2° (minimized)

Fe	0.000024	0.000078	-0.125957
N	2.151760	0.194718	0.013966
C	2.936332	-0.552188	-0.755664
C	4.318259	-0.499767	-0.682717
C	4.866914	0.379376	0.242837
H	5.943735	0.453055	0.333125
C	4.058614	1.168264	1.052286
H	4.490129	1.851349	1.769864
C	2.689859	1.034438	0.891861
N	2.224307	-1.378852	-1.642726
N	0.867837	-1.306066	-1.664176
C	0.479261	-2.157976	-2.596313
H	-0.571510	-2.282467	-2.808674
C	1.580491	-2.797995	-3.192463
H	1.571348	-3.536781	-3.975989
C	2.676412	-2.274531	-2.561950
H	3.726933	-2.471744	-2.699888
N	1.734663	1.758696	1.627932
N	0.416214	1.526701	1.399608
C	-0.235005	2.337689	2.214667
H	-1.314225	2.340129	2.223601
C	0.654045	3.110435	2.982265
H	0.414693	3.853539	3.724058
C	1.901491	2.713722	2.581897
H	2.878041	3.039700	2.900652
N	-2.151818	-0.194762	0.013913
C	-2.936424	0.552079	-0.755726
C	-4.318353	0.499541	-0.682846
H	-4.951478	1.105888	-1.314751
C	-4.866977	-0.379661	0.242667
H	-5.943795	-0.453436	0.332905
C	-4.058638	-1.168481	1.052144
C	-2.689891	-1.034520	0.891774
N	-2.224415	1.378821	-1.642730
N	-0.867939	1.306163	-1.664050
C	-0.479342	2.158116	-2.596144
H	0.571436	2.282712	-2.808408
C	-1.580581	2.798031	-3.192395
H	-1.571430	3.536819	-3.975918
C	-2.676517	2.274458	-2.561990
H	-3.727044	2.471565	-2.700029
N	-1.734629	-1.758669	1.627873
N	-0.416219	-1.526490	1.399540
C	0.235136	-2.337357	2.214620
H	1.314356	-2.339643	2.223556
C	-0.653812	-3.110206	2.982242
H	-0.414350	-3.853252	3.724057
C	-1.901322	-2.713685	2.581863
H	-2.877825	-3.039790	2.900631
H	-4.490115	-1.851613	1.769701
H	4.951365	-1.106152	-1.314603

[Fe(bpp)$_2$]$^{2+}$, pathway A; ϕ = 165° (fixed), θ' = 82.0° (minimized)

Fe	-0.000001	0.248092	0.000006
N	2.117856	-0.034770	0.361544
C	3.002260	0.703465	-0.300285
C	4.369276	0.542238	-0.149619
C	4.793138	-0.436826	0.740862
C	3.879533	-1.207936	1.449030
C	2.536877	-0.957451	1.220652
N	2.409940	1.658530	-1.146160
N	1.055546	1.769281	-1.166991
C	0.790438	2.757134	-2.004834
H	-0.232727	3.043978	-2.193306
C	1.971434	3.299691	-2.541161
H	2.067040	4.108381	-3.245951
C	2.983448	2.576857	-1.969540
H	4.052190	2.651194	-2.086622
N	1.487034	-1.636628	1.863970
N	0.207433	-1.312127	1.548925
C	-0.552700	-2.080044	2.309451
H	-1.627590	-2.010577	2.241185
C	0.226154	-2.913353	3.132057
H	-0.114755	-3.638094	3.851968
C	1.522402	-2.602695	2.821024
H	2.450087	-2.991699	3.207630
N	-2.117855	-0.034758	-0.361547
C	-3.002262	0.703654	0.300072
C	-4.369276	0.542465	0.149355
C	-4.793133	-0.436778	-0.740930
C	-3.879525	-1.208109	-1.448851
C	-2.536871	-0.957631	-1.220457
N	-2.409956	1.658810	1.145852
N	-1.055537	1.769146	1.167166
C	-0.790414	2.756641	2.005432
H	0.232781	3.042998	2.194478
C	-1.971430	3.299456	2.541451
H	-2.067026	4.107912	3.246512
C	-2.983490	2.577489	1.968821
H	-4.052265	2.652378	2.085257
N	-1.487026	-1.636950	-1.863623
N	-0.207423	-1.311971	-1.549074
C	0.552700	-2.079467	-2.310030
H	1.627590	-2.009435	-2.242348
C	-0.226160	-2.913089	-3.132312
H	0.114741	-3.637567	-3.852490
C	-1.522402	-2.603473	-2.820214
H	-2.450090	-2.993157	-3.206130
H	5.853829	-0.597377	0.890106
H	-5.853824	-0.597298	-0.890212
H	5.083378	1.148134	-0.688837
H	4.213953	-1.963437	2.145557
H	-5.083380	1.148490	0.688427
H	-4.213942	-1.963715	-2.145264

[Fe(bpp)$_2$]$^{2+}$, pathway A; ϕ = 160° (fixed), θ' = 78.7° (minimized)

Fe	0.000094	-0.000551	-0.331618
N	2.112383	0.330297	0.045980
C	3.016126	-0.333768	-0.665420
C	4.377499	-0.206334	-0.445094
C	4.773063	0.664882	0.562696
C	3.838557	1.379071	1.302681
C	2.504722	1.174632	0.992435
N	2.449887	-1.153007	-1.658455
N	1.105769	-1.109827	-1.857237
C	0.866823	-1.933770	-2.863059
H	-0.143155	-2.072577	-3.217197
C	2.054868	-2.522678	-3.331017
H	2.169796	-3.229632	-4.135212
C	3.043529	-1.998795	-2.542948
H	4.108571	-2.164027	-2.548937
N	1.435379	1.831476	1.627202
N	0.169998	1.561806	1.218138
C	-0.613495	2.330006	1.954750
H	-1.683486	2.296066	1.817708
C	0.136281	3.111710	2.851243
H	-0.227021	3.827914	3.568674
C	1.439706	2.767387	2.613461
H	2.352804	3.117487	3.066312
N	-2.112198	-0.331209	0.044768
C	-3.015867	0.332870	-0.666751
C	-4.377170	0.208843	-0.443657
C	-4.772838	-0.659150	0.566861
C	-3.838649	-1.376849	1.303827
C	-2.504800	-1.175727	0.990956
N	-2.449656	1.151330	-1.660421
N	-1.105643	1.109087	-1.858386
C	-0.866368	1.933600	-2.863698
H	0.143734	2.073327	-3.216887
C	-2.054360	2.521955	-3.332290
H	-2.169185	3.229346	-4.136143
C	-3.043188	1.996412	-2.545639
H	-4.108295	2.160567	-2.552660
N	-1.435684	-1.833591	1.625047
N	-0.170280	-1.563039	1.217277
C	0.612986	-2.330762	1.954534
H	1.683036	-2.295862	1.818644
C	-0.137001	-3.113156	2.850067
H	0.226121	-3.829039	3.567873
C	-1.440367	-2.770445	2.610296
H	-2.353626	-3.121667	3.061726
H	5.828194	0.796591	0.768598
H	-5.827841	-0.788052	0.775021
H	-5.108025	0.748299	-1.029019
H	-4.151030	-2.061730	2.079140
H	4.150813	2.064899	2.077189
H	5.108491	-0.744614	-1.031358

$[Fe(bpp)_2]^{2+}$, pathway A; ϕ = 155° (fixed), θ' = 74.8° (minimized)

Fe	-0.000008	-0.000403	-0.417394
N	2.086783	0.392952	0.053452
C	3.036575	-0.231612	-0.633012
C	4.383176	-0.082641	-0.345826
C	4.712232	0.769232	0.701809
C	3.729366	1.448452	1.411491
C	2.416335	1.225257	1.033145
N	2.533439	-1.035029	-1.672062
N	1.200645	-1.003940	-1.938327
C	1.024601	-1.805630	-2.975469
H	0.035635	-1.948717	-3.383012
C	2.243618	-2.365094	-3.397944
H	2.409044	-3.049308	-4.212911
C	3.183335	-1.848186	-2.547582
H	4.249590	-1.998360	-2.504145
N	1.304316	1.849096	1.625666
N	0.068513	1.567714	1.143286
C	-0.766662	2.308913	1.850657
H	-1.827029	2.260755	1.655073
C	-0.080272	3.084045	2.801651
H	-0.493209	3.780636	3.511428
C	1.239791	2.764754	2.628454
H	2.121302	3.120073	3.136436
N	-2.086835	-0.393443	0.053310
C	-3.036509	0.231342	-0.633106
C	-4.383116	0.083153	-0.345519
C	-4.712315	-0.768188	0.702515
C	-3.729620	-1.448188	1.411694
C	-2.416541	-1.225722	1.032972
N	-2.533157	1.034899	-1.671967
N	-1.200223	1.003360	-1.938290
C	-1.024072	1.804974	-2.975499
H	-0.035100	1.947653	-3.383282
C	-2.243014	2.365161	-3.397628
H	-2.408266	3.049539	-4.212513
C	-3.182869	1.848892	-2.546945
H	-4.249089	1.999748	-2.503130
N	-1.304627	-1.849600	1.625623
N	-0.068759	-1.568345	1.143274
C	0.766310	-2.309553	1.850736
H	1.826675	-2.261569	1.655165
C	0.079757	-3.084378	2.801908
H	0.492607	-3.780860	3.511832
C	-1.240262	-2.764873	2.628745
H	-2.121815	-3.119873	3.136874
H	5.753497	0.916938	0.960418
H	-5.753580	-0.915297	0.961455
H	-5.152158	0.590083	-0.910629
H	-3.990809	-2.123045	2.214249
H	5.152338	-0.589251	-0.911064
H	3.990435	2.123484	2.213937

$[Fe(bpp)_2]^{2+}$, pathway A; ϕ = 150° (fixed), θ' = 71.0° (minimized)

Fe	0.000836	0.000318	-0.507283
N	-0.364462	2.073304	0.056541
C	0.258217	3.038314	-0.609667
C	0.153992	4.373683	-0.257447
C	-0.653406	4.675364	0.832998
C	-1.333819	3.677432	1.520381
C	-1.156122	2.377849	1.076583
N	1.011953	2.565143	-1.699605
N	0.959380	1.242524	-2.009577
C	1.715486	1.096446	-3.085229
H	1.835093	0.120083	-3.529537
C	2.266260	2.325060	-3.489392
H	2.916088	2.514530	-4.327046
C	1.792331	3.238677	-2.586940
H	1.950923	4.302374	-2.516314
N	-1.788537	1.254338	1.636754
N	-1.565242	0.040081	1.075620
C	-2.302977	-0.811125	1.767437
H	-2.297278	-1.860034	1.512547
C	-3.018314	-0.156660	2.785567
H	-3.699715	-0.587973	3.499315
C	-2.664845	1.161259	2.671937
H	-2.971608	2.022918	3.242026
N	0.363262	-2.073144	0.056908
C	-0.260964	-3.037592	-0.608659
C	-0.160126	-4.372697	-0.254267
C	0.645821	-4.674651	0.837184
C	1.328168	-3.677319	1.523540
C	1.153728	-2.377938	1.077748
N	-1.012427	-2.564140	-1.700034
N	-0.960624	-1.241163	-2.008437
C	-1.714093	-1.094767	-3.085931
H	-1.833699	-0.118068	-3.529521
C	-2.262618	-2.323616	-3.492628
H	-2.910317	-2.512892	-4.331981
C	-1.789271	-3.237796	-2.590420
H	-1.946389	-4.301847	-2.521590
N	1.788499	-1.255096	1.636591
N	1.566528	-0.040642	1.075221
C	2.308220	0.809307	1.764330
H	2.304634	1.857969	1.508434
C	3.024348	0.153957	2.781308
H	3.708551	0.584203	3.493015
C	2.666901	-1.163098	2.670093
H	2.972671	-2.024933	3.240444
H	-0.668984	-5.153976	-0.800769
H	1.970190	-3.918117	2.358860
H	0.661121	5.155304	-0.805058
H	-1.977025	3.917926	2.354878
H	0.755175	-5.704974	1.148457
H	-0.765515	5.705895	1.142615

[Fe(bpp)$_2$]$^{2+}$, pathway A; ϕ = 145° (fixed), θ' = 68.0° (minimized)

Fe	0.000071	0.000590	-0.593971
N	2.055313	0.338414	0.063059
C	3.032973	-0.290921	-0.578277
C	4.355101	-0.223585	-0.171371
C	4.629505	0.557752	0.945285
C	3.620215	1.249108	1.603840
C	2.335885	1.107941	1.105499
N	2.588980	-1.013008	-1.702892
N	1.280247	-0.932442	-2.063612
C	1.164405	-1.662041	-3.161405
H	0.203837	-1.756952	-3.644863
C	2.399418	-2.223003	-3.529376
H	2.612045	-2.856522	-4.373828
C	3.283950	-1.785254	-2.580651
H	4.341495	-1.963601	-2.472829
N	1.205592	1.759265	1.626800
N	0.012219	1.573345	1.010790
C	-0.849174	2.323666	1.675968
H	-1.886124	2.348667	1.377225
C	-0.221882	3.010070	2.730609
H	-0.666490	3.693786	3.433916
C	1.090548	2.625241	2.668386
H	1.934696	2.903338	3.277982
N	-2.055141	-0.338836	0.062492
C	-3.033049	0.290152	-0.578917
C	-4.355178	0.221908	-0.172245
C	-4.629291	-0.559529	0.944392
C	-3.619791	-1.250332	1.603196
C	-2.335554	-1.108539	1.104855
N	-2.589492	1.012885	-1.703109
N	-1.280634	0.933384	-2.063538
C	-1.165213	1.663147	-3.161228
H	-0.204558	1.758568	-3.644489
C	-2.400433	2.223538	-3.529160
H	-2.613568	2.857161	-4.373349
C	-3.284903	1.784714	-2.580904
H	-4.342674	1.962034	-2.473667
N	-1.205205	-1.759227	1.626594
N	-0.011565	-1.571786	1.011669
C	0.850006	-2.321410	1.677338
H	1.887114	-2.345225	1.379390
C	0.222691	-3.008848	2.731278
H	0.667398	-3.692404	3.434719
C	-1.090079	-2.625359	2.668038
H	-1.934362	-2.904598	3.276805
H	5.650153	0.641758	1.297775
H	-5.649859	-0.643945	1.296909
H	-5.148080	0.734734	-0.697823
H	-3.840251	-1.875549	2.456580
H	5.147843	-0.736685	-0.696936
H	3.840848	1.874433	2.457051

$[Fe(bpp)_2]^{2+}$, pathway A; $\phi = 140°$ (fixed), $\theta' = 65.0°$ (minimized)

Fe	-0.000093	0.000400	-0.679069
N	2.031642	0.319400	0.069279
C	3.021828	-0.320737	-0.541968
C	4.326650	-0.290534	-0.078797
C	4.571009	0.465317	1.062285
C	3.550892	1.170948	1.687697
C	2.286518	1.069586	1.131424
N	2.612811	-1.016735	-1.696976
N	1.322396	-0.908600	-2.113792
C	1.244176	-1.617792	-3.228317
H	0.304041	-1.690290	-3.754022
C	2.484877	-2.192335	-3.551965
H	2.724978	-2.814556	-4.397692
C	3.333053	-1.785093	-2.557564
H	4.382045	-1.982074	-2.407343
N	1.152939	1.746477	1.608625
N	-0.016896	1.593631	0.941414
C	-0.884330	2.366917	1.571493
H	-1.906846	2.421902	1.230578
C	-0.284192	3.035489	2.653171
H	-0.739360	3.731236	3.337618
C	1.018373	2.614659	2.646113
H	1.843687	2.869401	3.290548
N	-2.031841	-0.318747	0.069696
C	-3.021929	0.321991	-0.540963
C	-4.326739	0.292704	-0.077394
C	-4.571415	-0.463958	1.063178
C	-3.551371	-1.170785	1.687368
C	-2.286747	-1.069384	1.131518
N	-2.612380	1.017675	-1.696331
N	-1.322062	0.908678	-2.113433
C	-1.243764	1.616412	-3.228905
H	-0.303750	1.688609	-3.754902
C	-2.484682	2.190388	-3.553273
H	-2.724491	2.811307	-4.400202
C	-3.332585	1.785367	-2.557641
H	-4.381437	1.983373	-2.406619
N	-1.153019	-1.746654	1.608306
N	0.017020	-1.593205	0.941491
C	0.884514	-2.366890	1.571156
H	1.907331	-2.421474	1.230622
C	0.283949	-3.036445	2.652030
H	0.739118	-3.732571	3.336032
C	-1.018676	-2.615757	2.645065
H	-1.844174	-2.870894	3.289185
H	5.577189	0.518734	1.459646
H	-5.577749	-0.517229	1.460502
H	-5.129879	0.815088	-0.577576
H	-3.749292	-1.778633	2.558598
H	5.129796	-0.812262	-0.579521
H	3.748868	1.777944	2.559626

[Fe(bpp)$_2$]$^{2+}$, pathway B; ϕ = 164.4° (minimized), θ' = 79.3° (constrained)

Fe	0.000000	0.000000	-0.260475
N	-2.134138	-0.280065	0.034319
C	-2.999328	0.367170	-0.738385
C	-4.370470	0.252579	-0.580465
C	-4.819562	-0.588530	0.430050
C	-3.926254	-1.287067	1.233174
H	-4.280252	-1.951997	2.007972
C	-2.577568	-1.096054	0.984292
N	-2.382714	1.164601	-1.718863
N	-1.027346	1.158951	-1.812690
C	-0.735747	1.955170	-2.827185
H	0.293896	2.116269	-3.107459
C	-1.900847	2.491920	-3.403000
H	-1.974149	3.169794	-4.236658
C	-2.931531	1.958390	-2.677575
H	-3.997290	2.087706	-2.772006
N	-1.545620	-1.739308	1.691408
N	-0.260213	-1.516089	1.319005
C	0.479904	-2.246772	2.134309
H	1.554965	-2.238073	2.038697
C	-0.319072	-2.963488	3.042580
H	0.002153	-3.640851	3.815761
C	-1.605685	-2.615393	2.730038
H	-2.542111	-2.923419	3.165527
N	2.134138	0.280065	0.034319
C	2.999328	-0.367170	-0.738385
C	4.370470	-0.252579	-0.580465
H	5.069092	-0.783608	-1.211304
C	4.819562	0.588531	0.430050
H	5.884138	0.708745	0.588694
C	3.926254	1.287067	1.233174
C	2.577568	1.096054	0.984292
N	2.382714	-1.164601	-1.718863
N	1.027346	-1.158951	-1.812690
C	0.735747	-1.955170	-2.827186
H	-0.293896	-2.116269	-3.107459
C	1.900847	-2.491920	-3.403000
H	1.974149	-3.169794	-4.236658
C	2.931531	-1.958390	-2.677575
H	3.997290	-2.087706	-2.772006
N	1.545620	1.739308	1.691408
N	0.260213	1.516089	1.319005
C	-0.479904	2.246772	2.134310
H	-1.554965	2.238073	2.038697
C	0.319072	2.963488	3.042580
H	-0.002153	3.640851	3.815761
C	1.605685	2.615393	2.730038
H	2.542111	2.923419	3.165527
H	4.280252	1.951997	2.007972
H	-5.069092	0.783608	-1.211304
H	-5.884138	-0.708745	0.588694

[Fe(bpp)$_2$]$^{2+}$, pathway B; ϕ = 163.8° (minimized), θ' = 76.6° (constrained)

Fe	0.000336	0.001212	-0.271409
N	-2.153831	-0.007161	0.032421
C	-2.937609	0.720814	-0.755242
C	-4.312259	0.774061	-0.597123
C	-4.856602	0.021650	0.436241
H	-5.927676	0.033680	0.596905
C	-4.051000	-0.755689	1.259423
H	-4.479636	-1.351278	2.052710
C	-2.690350	-0.738027	1.003547
N	-2.231050	1.421709	-1.748470
N	-0.887051	1.248411	-1.841098
C	-0.500880	1.991250	-2.864528
H	0.540299	2.019704	-3.147076
C	-1.591484	2.659724	-3.447831
H	-1.582062	3.330385	-4.290434
C	-2.678467	2.270529	-2.712548
H	-3.719971	2.531584	-2.805893
N	-1.744128	-1.493490	1.719160
N	-0.443983	-1.446258	1.334266
C	0.198562	-2.268071	2.145311
H	1.263765	-2.405690	2.039126
C	-0.679746	-2.861786	3.069045
H	-0.444686	-3.570331	3.845259
C	-1.910483	-2.344587	2.767081
H	-2.874907	-2.519638	3.214983
N	2.153803	0.006935	0.037614
C	2.939606	-0.712456	-0.755885
C	4.313869	-0.767276	-0.595036
H	4.946442	-1.352676	-1.246925
C	4.855660	-0.025799	0.447534
H	5.926340	-0.039464	0.610659
C	4.047984	0.742781	1.276894
C	2.687942	0.727559	1.017665
N	2.235531	-1.402776	-1.758258
N	0.891619	-1.229159	-1.851754
C	0.507998	-1.960833	-2.884159
H	-0.532571	-1.986718	-3.169190
C	1.600182	-2.622281	-3.472523
H	1.592863	-3.283625	-4.322472
C	2.685429	-2.240631	-2.730746
H	3.727274	-2.500045	-2.824851
N	1.739835	1.475039	1.739142
N	0.440596	1.431532	1.350757
C	-0.204169	2.244013	2.169428
H	-1.269186	2.382373	2.062346
C	0.671763	2.827765	3.101733
H	0.434550	3.527454	3.885298
C	1.903408	2.314443	2.796877
H	2.866714	2.484894	3.248941
H	4.474639	1.329958	2.077483
H	-4.943224	1.366284	-1.244397

[Fe(bpp)$_2$]$^{2+}$, pathway B; ϕ = 160.8° (minimized), θ' = 71.8° (constrained)

Fe	0.000270	-0.000815	0.335200
N	-2.146690	-0.125393	-0.027341
C	-2.916937	-0.844767	0.780852
C	-4.281658	-0.980473	0.587878
C	-4.831483	-0.323340	-0.505603
H	-5.894901	-0.402040	-0.695289
C	-4.041010	0.445055	-1.351533
H	-4.474840	0.971929	-2.189417
C	-2.690083	0.516564	-1.055699
N	-2.205117	-1.456898	1.827329
N	-0.871308	-1.226011	1.932432
C	-0.474597	-1.900023	2.999143
H	0.561676	-1.872391	3.299290
C	-1.548766	-2.580271	3.598573
H	-1.528672	-3.206723	4.474342
C	-2.636657	-2.272454	2.826344
H	-3.668781	-2.569493	2.916210
N	-1.764893	1.287259	-1.782338
N	-0.492289	1.382277	-1.323593
C	0.131404	2.187083	-2.165781
H	1.172964	2.424698	-2.011459
C	-0.732838	2.627945	-3.183848
H	-0.507738	3.289965	-4.002916
C	-1.934543	2.034057	-2.906534
H	-2.880646	2.089350	-3.419625
N	2.146813	0.124888	-0.029701
C	2.918741	0.837368	0.783005
C	4.283349	0.973278	0.589372
H	4.904013	1.553596	1.257101
C	4.831209	0.324068	-0.509817
H	5.894479	0.403179	-0.700161
C	4.038951	-0.436957	-1.360713
C	2.688309	-0.509360	-1.063821
N	2.208829	1.442143	1.835066
N	0.874875	1.211984	1.940019
C	0.480197	1.878403	3.012235
H	-0.555748	1.849677	3.313407
C	1.555818	2.552981	3.615438
H	1.537433	3.172949	4.495845
C	2.642447	2.249736	2.839644
H	3.675009	2.544938	2.930512
N	1.761415	-1.273170	-1.795531
N	0.489418	-1.370836	-1.335706
C	-0.136416	-2.167531	-2.183990
H	-1.178005	-2.405369	-2.030175
C	0.725783	-2.600277	-3.207275
H	0.498647	-3.254691	-4.031870
C	1.928553	-2.010131	-2.926574
H	2.873797	-2.061741	-3.441635
H	4.471176	-0.957762	-2.203215
H	-4.900957	-1.566452	1.251922

[Fe(bpp)$_2$]$^{2+}$, pathway B; ϕ = 157.9° (minimized), θ' = 66.8° (constrained)

Fe	0.000000	-0.000001	0.399987
N	-2.129267	0.296257	-0.020351
C	-3.044535	-0.195347	0.807027
C	-4.404723	-0.058594	0.585430
C	-4.789944	0.621401	-0.562924
H	-5.843719	0.750790	-0.777235
C	-3.844876	1.151504	-1.432690
H	-4.146611	1.701870	-2.312369
C	-2.513209	0.966751	-1.101146
N	-2.490644	-0.886073	1.898878
N	-1.139966	-0.935752	2.020120
C	-0.904277	-1.641557	3.114195
H	0.111826	-1.821869	3.430039
C	-2.103536	-2.056984	3.717638
H	-2.224509	-2.642310	4.613523
C	-3.094488	-1.550600	2.919739
H	-4.166856	-1.617984	3.003523
N	-1.439634	1.497928	-1.837523
N	-0.188584	1.407025	-1.323012
C	0.598367	2.019459	-2.189675
H	1.658875	2.086769	-1.999712
C	-0.135889	2.519012	-3.280340
H	0.232515	3.065068	-4.132342
C	-1.432930	2.165432	-3.022721
H	-2.335045	2.341255	-3.585554
N	2.129267	-0.296257	-0.020350
C	3.044534	0.195348	0.807027
C	4.404723	0.058595	0.585430
H	5.142002	0.457779	1.267491
C	4.789944	-0.621400	-0.562924
C	3.844875	-1.151503	-1.432690
C	2.513208	-0.966750	-1.101146
N	2.490644	0.886074	1.898878
N	1.139966	0.935751	2.020122
C	0.904277	1.641556	3.114197
H	-0.111826	1.821866	3.430042
C	2.103536	2.056985	3.717638
H	2.224510	2.642310	4.613523
C	3.094488	1.550602	2.919738
H	4.166856	1.617988	3.003520
N	1.439633	-1.497927	-1.837524
N	0.188583	-1.407026	-1.323013
C	-0.598367	-2.019462	-2.189677
H	-1.658874	-2.086774	-1.999713
C	0.135891	-2.519014	-3.280341
H	-0.232513	-3.065070	-4.132343
C	1.432931	-2.165432	-3.022723
H	2.335046	-2.341253	-3.585555
H	4.146610	-1.701869	-2.312369
H	-5.142003	-0.457778	1.267491
H	5.843718	-0.750789	-0.777235

[Fe(bpp)$_2$]$^{2+}$, pathway B; ϕ = 157.3° (minimized), θ' = 63.6° (constrained)

Fe	-0.000293	0.000036	-0.415404
N	0.147767	2.148233	0.016787
C	0.759037	2.959073	-0.839454
C	0.901677	4.318269	-0.615625
C	0.382261	4.819085	0.571090
C	-0.268832	3.986913	1.473055
H	-0.698064	4.382133	2.382671
C	-0.371190	2.648534	1.133238
N	1.282072	2.290465	-1.960059
N	1.101502	0.949691	-2.059640
C	1.696152	0.591023	-3.186264
H	1.686895	-0.443210	-3.494446
C	2.273051	1.698543	-3.829861
H	2.822176	1.712967	-4.756231
C	1.987002	2.765848	-3.020797
H	2.231060	3.810476	-3.124228
N	-1.077515	1.700355	1.893212
N	-1.302349	0.476162	1.358323
C	-2.024163	-0.175733	2.252391
H	-2.342712	-1.187643	2.052601
C	-2.274826	0.620769	3.384602
H	-2.836746	0.361148	4.265880
C	-1.655146	1.812106	3.119886
H	-1.587603	2.715083	3.704258
N	-0.146923	-2.148213	0.017650
C	-0.756285	-2.959867	-0.839220
C	-0.897492	-4.319239	-0.615523
H	-1.391613	-4.969855	-1.322846
C	-0.379033	-4.819380	0.571886
H	-0.470294	-5.876119	0.790967
C	0.269652	-3.986310	1.474724
C	0.370843	-2.647812	1.134989
N	-1.279032	-2.292053	-1.960458
N	-1.100136	-0.951094	-2.060118
C	-1.694665	-0.593215	-3.187049
H	-1.686504	0.441003	-3.495312
C	-2.269908	-1.701489	-3.830782
H	-2.818606	-1.716684	-4.757392
C	-1.982931	-2.768369	-3.021458
H	-2.225716	-3.813291	-3.124900
N	1.074716	-1.698760	1.896128
N	1.299401	-0.474506	1.361383
C	2.018744	0.178324	2.256758
H	2.336814	1.190461	2.057335
C	2.267863	-0.617592	3.389704
H	2.827701	-0.357182	4.272075
C	1.649946	-1.809626	3.124018
H	1.582090	-2.712500	3.708513
H	0.698029	-4.380917	2.385007
H	1.397563	4.968234	-1.322312
H	0.474562	5.875726	0.790247

$[Fe(bpp)_2]^{2+}$, pathway B; ϕ = 157.0° (minimized), θ' = 60.6° (constrained)

Fe	-0.000333	-0.000080	0.430777
N	-0.138933	2.157005	-0.009050
C	0.322941	2.645797	-1.155713
C	0.219393	3.983521	-1.498323
C	-0.361129	4.831937	-0.564416
C	-0.816539	4.345011	0.653500
H	-1.259323	5.006101	1.384694
C	-0.687679	2.983860	0.874316
N	0.991986	1.690890	-1.939615
N	1.270058	0.484140	-1.392761
C	1.954434	-0.173499	-2.311693
H	2.306240	-1.173621	-2.108109
C	2.122596	0.601031	-3.474439
H	2.639270	0.332526	-4.380420
C	1.495764	1.786247	-3.199908
H	1.377044	2.674927	-3.797850
N	-1.180916	2.328359	2.015777
N	-1.054038	0.980751	2.095298
C	-1.631651	0.633235	3.234691
H	-1.656207	-0.403815	3.532289
C	-2.142880	1.756077	3.906548
H	-2.664190	1.782257	4.848590
C	-1.836249	2.820094	3.100278
H	-2.033939	3.872638	3.221057
N	0.138069	-2.157099	-0.010876
C	-0.318140	-2.644246	-1.160522
C	-0.215175	-3.981908	-1.503541
H	-0.594525	-4.363528	-2.440718
C	0.358855	-4.832047	-0.567205
H	0.444785	-5.889146	-0.786546
C	0.808832	-4.346744	0.653371
C	0.681079	-2.985551	0.874538
N	-0.980961	-1.687581	-1.947575
N	-1.260802	-0.481088	-1.401166
C	-1.938427	0.178560	-2.323661
H	-2.289988	1.179000	-2.121161
C	-2.100214	-0.594364	-3.488334
H	-2.610620	-0.324087	-4.397336
C	-1.476815	-1.780792	-3.211159
H	-1.355396	-2.668918	-3.809381
N	1.169588	-2.331779	2.019015
N	1.044770	-0.984035	2.098873
C	1.617635	-0.638320	3.241213
H	1.642599	0.398473	3.539676
C	2.123656	-1.762522	3.914697
H	2.640414	-1.790308	4.859196
C	1.818949	-2.825426	3.106229
H	2.014123	-3.878420	3.227185
H	1.246805	-5.009076	1.386340
H	0.603027	4.366535	-2.433186
H	-0.447841	5.889029	-0.783483

$[Fe(bpp)_2]^{2+}$, pathway C; $\phi = 165°$ (fixed), $\theta' = 89.5°$ (minimized)

Fe	0.202160	-0.046409	-0.210789
N	0.104830	2.137141	-0.280099
C	0.891129	2.799596	-1.120758
C	0.890627	4.179279	-1.222815
C	0.014486	4.870300	-0.393548
C	-0.814887	4.193885	0.490006
H	-1.496332	4.732761	1.132898
C	-0.727978	2.810031	0.505728
N	1.714253	1.951853	-1.887932
N	1.625934	0.610934	-1.697375
C	2.493862	0.069742	-2.538714
H	2.612971	-1.002146	-2.578750
C	3.156298	1.057476	-3.284880
H	3.911178	0.919228	-4.040374
C	2.633179	2.244669	-2.843720
H	2.847557	3.259146	-3.137891
N	-1.499329	1.985379	1.338489
N	-1.331494	0.643415	1.259079
C	-2.172360	0.126572	2.134799
H	-2.229197	-0.943790	2.261866
C	-2.900764	1.133163	2.798155
H	-3.653151	1.012928	3.559211
C	-2.444768	2.306906	2.265214
H	-2.719036	3.327883	2.473679
N	-0.082657	-2.131820	0.266923
C	-0.965141	-2.855610	-0.417416
C	-1.158286	-4.206890	-0.182648
H	-1.874033	-4.791366	-0.742948
C	-0.382873	-4.791978	0.810423
C	0.542593	-4.047999	1.531843
C	0.654926	-2.704268	1.215283
N	-1.670823	-2.119572	-1.383512
N	-1.389117	-0.800313	-1.544437
C	-2.207828	-0.374626	-2.488626
H	-2.178039	0.657290	-2.803103
C	-3.033397	-1.414753	-2.956375
H	-3.791402	-1.364500	-3.719710
C	-2.666369	-2.511468	-2.226686
H	-3.035374	-3.523805	-2.245790
N	1.540459	-1.818974	1.851655
N	1.585443	-0.523576	1.445115
C	2.470032	0.064259	2.229245
H	2.688539	1.113606	2.103338
C	3.016731	-0.844599	3.155001
H	3.758160	-0.652264	3.912015
C	2.401043	-2.035389	2.885183
H	2.508734	-3.002638	3.347911
H	1.145143	-4.510198	2.300615
H	1.535568	4.706994	-1.911014
H	-0.021310	5.952065	-0.438490
H	-0.499891	-5.847549	1.024726

$[Fe(bpp)_2]^{2+}$, pathway C; ϕ = 160° (fixed), θ' = 89.5° (minimized)

Fe	0.261047	-0.061796	-0.281227
N	0.112204	2.123582	-0.278511
C	0.872445	2.824068	-1.113075
C	0.842170	4.205377	-1.180350
C	-0.036313	4.857168	-0.322149
C	-0.839189	4.141420	0.554489
H	-1.523321	4.649333	1.219390
C	-0.723555	2.759590	0.534368
N	1.702765	2.014465	-1.914074
N	1.643831	0.666955	-1.761416
C	2.513098	0.167890	-2.627515
H	2.653623	-0.899721	-2.699527
C	3.146425	1.189981	-3.351997
H	3.895304	1.088553	-4.119218
C	2.603674	2.353096	-2.872004
H	2.793816	3.379592	-3.140003
N	-1.467225	1.900607	1.356394
N	-1.276654	0.565319	1.238432
C	-2.094897	0.011051	2.112556
H	-2.131421	-1.063163	2.212359
C	-2.831384	0.986370	2.813077
H	-3.570323	0.833100	3.581348
C	-2.403414	2.181605	2.305539
H	-2.691657	3.191823	2.545194
N	-0.091376	-2.123231	0.268754
C	-1.009198	-2.835381	-0.379687
C	-1.258375	-4.166453	-0.088037
H	-2.002648	-4.742245	-0.619523
C	-0.500039	-4.743573	0.922767
C	0.463603	-4.011667	1.605599
C	0.630937	-2.687952	1.232997
N	-1.690772	-2.109543	-1.370223
N	-1.347204	-0.814740	-1.593734
C	-2.158336	-0.389933	-2.544820
H	-2.082982	0.624539	-2.905144
C	-3.038873	-1.408316	-2.957129
H	-3.803578	-1.354498	-3.713499
C	-2.714541	-2.489124	-2.184774
H	-3.131450	-3.482416	-2.155562
N	1.560163	-1.816406	1.823878
N	1.661524	-0.544832	1.357883
C	2.583034	0.035257	2.104091
H	2.850182	1.065748	1.926899
C	3.099070	-0.856443	3.063813
H	3.860272	-0.666322	3.801503
C	2.419748	-2.025441	2.859929
H	2.485676	-2.974113	3.366850
H	1.053915	-4.468685	2.386916
H	1.465847	4.764264	-1.863477
H	-0.095271	5.938822	-0.339346
H	-0.660377	-5.783581	1.180998

[Fe(bpp)$_2$]$^{2+}$, pathway C; ϕ = 155° (fixed), θ' = 89.2° (minimized)

Fe	-0.320565	0.079589	-0.362597
N	-0.106874	-2.105716	-0.271107
C	-0.834461	-2.853751	-1.095295
C	-0.771287	-4.234798	-1.114638
C	0.105201	-4.835219	-0.217833
C	0.873581	-4.071459	0.648641
H	1.555588	-4.539327	1.343990
C	0.726583	-2.694615	0.579331
N	-1.669228	-2.095550	-1.941526
N	-1.645795	-0.742435	-1.842021
C	-2.506889	-0.297502	-2.745176
H	-2.668835	0.763039	-2.862668
C	-3.100946	-1.361601	-3.440582
H	-3.834754	-1.308552	-4.227020
C	-2.544860	-2.491948	-2.900808
H	-2.708024	-3.532305	-3.130357
N	1.433157	-1.794140	1.388169
N	1.212945	-0.468832	1.227396
C	1.999298	0.128794	2.102402
H	2.009417	1.205873	2.174011
C	2.743706	-0.807243	2.846326
H	3.461836	-0.614534	3.625388
C	2.356231	-2.026030	2.363581
H	2.663780	-3.021682	2.637593
N	0.078782	2.120961	0.251170
C	1.045586	2.813690	-0.343826
C	1.361343	4.111191	0.022503
H	2.145008	4.672113	-0.466040
C	0.617010	4.673759	1.051973
C	-0.394491	3.960327	1.683448
C	-0.625754	2.669982	1.236957
N	1.707412	2.101493	-1.357074
N	1.304946	0.837174	-1.644731
C	2.105257	0.419561	-2.608097
H	1.983885	-0.571091	-3.018741
C	3.040407	1.410707	-2.961097
H	3.810685	1.355398	-3.711691
C	2.760412	2.467435	-2.140157
H	3.224709	3.436461	-2.058818
N	-1.602873	1.815246	1.772178
N	-1.752933	0.573663	1.243582
C	-2.716220	0.003423	1.942892
H	-3.028153	-1.003107	1.709776
C	-3.207796	0.870134	2.937384
H	-3.991210	0.680769	3.651688
C	-2.470981	2.013797	2.803424
H	-2.502715	2.938758	3.355386
H	-0.970870	4.405294	2.481724
H	-1.368289	-4.830776	-1.789923
H	0.828142	5.688262	1.367211
H	0.189896	-5.914681	-0.196303

$[Fe(bpp)_2]^{2+}$, pathway C; $\phi = 150°$ (fixed), $\theta' = 89.2°$ (minimized)

Fe	0.374854	-0.097900	-0.423283
N	0.104171	2.085537	-0.269132
C	0.816514	2.863389	-1.080886
C	0.733279	4.243012	-1.073703
C	-0.147448	4.813835	-0.162241
C	-0.898298	4.021530	0.692612
H	-1.583346	4.465126	1.400555
C	-0.731698	2.648157	0.597494
N	1.662388	2.140715	-1.948377
N	1.665116	0.785484	-1.884248
C	2.529605	0.379651	-2.803154
H	2.709266	-0.674426	-2.949941
C	3.100906	1.472292	-3.472120
H	3.831374	1.453597	-4.263251
C	2.529727	2.577887	-2.897328
H	2.675581	3.626571	-3.098567
N	-1.420827	1.728675	1.397621
N	-1.185332	0.409293	1.217429
C	-1.960041	-0.207197	2.089741
H	-1.959716	-1.285154	2.149297
C	-2.710071	0.710215	2.851645
H	-3.420463	0.498927	3.632980
C	-2.338135	1.939129	2.384135
H	-2.653462	2.927639	2.674432
N	-0.096634	-2.109348	0.263412
C	-1.102337	-2.778115	-0.291655
C	-1.497317	-4.028198	0.153964
H	-2.317128	-4.567079	-0.298699
C	-0.786838	-4.571199	1.217490
C	0.269137	-3.884229	1.804129
C	0.578316	-2.641116	1.277974
N	-1.717071	-2.090507	-1.350146
N	-1.262302	-0.855877	-1.682857
C	-2.011362	-0.461359	-2.696062
H	-1.843486	0.504318	-3.147746
C	-2.965579	-1.438783	-3.036192
H	-3.705150	-1.396516	-3.817862
C	-2.749792	-2.462965	-2.156748
H	-3.244422	-3.414440	-2.050553
N	1.610977	-1.818351	1.755391
N	1.802823	-0.604944	1.178567
C	2.824225	-0.064355	1.816624
H	3.176585	0.916864	1.537719
C	3.311664	-0.923424	2.819754
H	4.133403	-0.752662	3.494618
C	2.514903	-2.032325	2.752030
H	2.526610	-2.942626	3.328589
H	0.818357	-4.311373	2.630788
H	1.318531	4.859533	-1.740651
H	-0.248625	5.891169	-0.119716
H	-1.061433	-5.548078	1.596126

$[Fe(bpp)_2]^{2+}$, pathway C; ϕ = 145° (fixed), θ' = 87.5° (minimized)

Fe	0.367795	-0.099365	-0.487396
N	0.064658	2.075984	-0.194147
C	0.741138	2.903940	-0.993554
C	0.640480	4.280585	-0.916987
C	-0.217371	4.799069	0.044193
C	-0.933440	3.958166	0.881979
H	-1.604736	4.359326	1.627129
C	-0.754295	2.594030	0.719440
N	1.567433	2.252333	-1.928450
N	1.597729	0.897697	-1.951818
C	2.434377	0.570685	-2.923119
H	2.629719	-0.467907	-3.142645
C	2.961301	1.717261	-3.542403
H	3.662876	1.763480	-4.358222
C	2.389831	2.770447	-2.881602
H	2.505848	3.832341	-3.022087
N	-1.417782	1.638498	1.495627
N	-1.187912	0.330837	1.244454
C	-1.941493	-0.324761	2.108382
H	-1.945667	-1.403941	2.121681
C	-2.669719	0.554691	2.933048
H	-3.359031	0.305803	3.722171
C	-2.308139	1.803844	2.515313
H	-2.614186	2.777331	2.860801
N	-0.112662	-2.097434	0.273629
C	-1.151772	-2.754102	-0.225201
C	-1.608075	-3.942686	0.319555
H	-2.458810	-4.472728	-0.084205
C	-0.909138	-4.439051	1.414685
C	0.200454	-3.775101	1.926130
C	0.565287	-2.595355	1.299300
N	-1.720234	-2.117831	-1.340741
N	-1.153760	-0.968354	-1.789049
C	-1.836356	-0.631163	-2.869197
H	-1.573737	0.261281	-3.416473
C	-2.857573	-1.561254	-3.136258
H	-3.566972	-1.548976	-3.946437
C	-2.750383	-2.497748	-2.144661
H	-3.318479	-3.394835	-1.959818
N	1.668578	-1.806068	1.661328
N	1.927784	-0.687738	0.936742
C	3.042626	-0.187816	1.439017
H	3.462397	0.716452	1.025522
C	3.522572	-0.978386	2.499641
H	4.406893	-0.820826	3.093752
C	2.623872	-2.003871	2.610342
H	2.599957	-2.849623	3.278050
H	0.750144	-4.175249	2.766022
H	1.197020	4.933395	-1.573461
H	-1.229200	-5.367374	1.871767
H	-0.329842	5.872066	0.138228

[Fe(bpp)$_2$]$^{2+}$, pathway C; ϕ = 140° (fixed), θ' = 87.4° (minimized)

Fe	0.419790	-0.123447	-0.588471
N	0.064013	2.037513	-0.158795
C	0.708880	2.909599	-0.939587
C	0.586479	4.280845	-0.814045
C	-0.259930	4.753690	0.179426
C	-0.942839	3.870611	0.997748
H	-1.606436	4.234037	1.768738
C	-0.746097	2.513807	0.785280
N	1.533764	2.315750	-1.916710
N	1.591682	0.965158	-2.005050
C	2.430994	0.699097	-2.993976
H	2.648107	-0.324099	-3.260224
C	2.928134	1.883493	-3.561966
H	3.624643	1.982900	-4.377369
C	2.332940	2.893168	-2.854237
H	2.424018	3.962289	-2.950547
N	-1.387297	1.532663	1.544752
N	-1.153768	0.234415	1.255960
C	-1.888083	-0.445549	2.117779
H	-1.888954	-1.524566	2.108744
C	-2.607851	0.408699	2.976720
H	-3.281603	0.135750	3.771318
C	-2.261497	1.669298	2.583665
H	-2.568422	2.631343	2.958539
N	-0.086928	-2.100656	0.212082
C	-1.154071	-2.736320	-0.251073
C	-1.625151	-3.907496	0.319697
H	-2.497237	-4.422765	-0.057181
C	-0.913294	-4.406201	1.405138
C	0.218979	-3.757887	1.885189
C	0.597049	-2.594626	1.234290
N	-1.738106	-2.095811	-1.356330
N	-1.155631	-0.965722	-1.832109
C	-1.872600	-0.608761	-2.883733
H	-1.605263	0.273340	-3.445293
C	-2.931541	-1.508194	-3.104825
H	-3.673290	-1.475957	-3.884902
C	-2.812005	-2.445274	-2.114903
H	-3.399807	-3.323435	-1.902845
N	1.717863	-1.818520	1.568335
N	1.987115	-0.718753	0.819856
C	3.103785	-0.215121	1.315088
H	3.531562	0.676651	0.883040
C	3.576170	-0.987113	2.392905
H	4.460795	-0.823633	2.984940
C	2.670645	-2.004350	2.522169
H	2.639299	-2.835913	3.207167
H	0.775446	-4.158084	2.720716
H	1.118262	4.965610	-1.458480
H	-0.389435	5.820656	0.313103
H	-1.243282	-5.322129	1.880265

[Fe(bpp)$_2$]$^{2+}$, pathway D; ϕ = 180.0° (minimized), θ' = 84.8° (constrained)

Fe	0.000543	-0.000582	-0.000134
N	0.003012	-0.000034	2.165074
C	-0.842793	0.778292	2.831828
C	-0.886375	0.817951	4.215428
C	0.006245	0.000780	4.897740
H	0.007553	0.001135	5.980827
C	0.897212	-0.816839	4.213808
H	1.590497	-1.450464	4.748246
C	0.850326	-0.778016	2.830291
N	-1.673927	1.545018	1.995906
N	-1.551302	1.417191	0.649225
C	-2.445636	2.239972	0.130035
H	-2.546856	2.319901	-0.941416
C	-3.159405	2.916460	1.135035
H	-3.943092	3.645036	1.013336
C	-2.641881	2.447181	2.311806
H	-2.889081	2.687584	3.332804
N	1.679500	-1.545192	1.992854
N	1.553777	-1.418053	0.646399
C	2.446945	-2.241086	0.125587
H	2.545709	-2.321554	-0.946052
C	3.162990	-2.917082	1.129262
H	3.946384	-3.645735	1.006148
C	2.648158	-2.447210	2.306972
H	2.897673	-2.687103	3.327528
N	-0.003281	0.000290	-2.164892
C	-0.850895	-0.777806	-2.829667
C	-0.898750	-0.816244	-4.213162
H	-1.592252	-1.449893	-4.747285
C	-0.008468	0.001780	-4.897499
H	-0.010575	0.002433	-5.980587
C	0.884518	0.818941	-4.215649
C	0.841994	0.778933	-2.832026
N	-1.679265	-1.545486	-1.991921
N	-1.552694	-1.418914	-0.645451
C	-2.445194	-2.242543	-0.124480
H	-2.543218	-2.323550	0.947187
C	-3.161639	-2.918383	-1.128005
H	-3.944654	-3.647413	-1.004711
C	-2.647770	-2.447775	-2.305828
H	-2.897846	-2.687331	-3.326325
N	1.673729	1.545513	-1.996582
N	1.552321	1.417437	-0.649785
C	2.447228	2.240022	-0.131307
H	2.549429	2.319732	0.940067
C	3.160144	2.916651	-1.136810
H	3.944013	3.645126	-1.015684
C	2.641503	2.447655	-2.313184
H	2.887827	2.688236	-3.334351
H	1.575985	1.453134	-4.751756
H	-1.578361	1.451904	4.751155

[Fe(bpp)$_2$]$^{2+}$, pathway D; ϕ = 179.9° (minimized), θ' = 81.3° (constrained)

Fe	0.005691	0.002763	0.000852
N	-0.104284	-0.008238	2.167144
C	0.585784	0.880829	2.874399
C	0.550796	0.922202	4.258040
C	-0.240010	-0.025067	4.896395
C	-0.964229	-0.962830	4.170326
H	-1.579197	-1.697112	4.670794
C	-0.862014	-0.904173	2.790952
N	1.353397	1.751924	2.080617
N	1.331740	1.602859	0.731019
C	2.135183	2.538543	0.256507
H	2.287486	2.629109	-0.808057
C	2.692968	3.306232	1.294315
H	3.380516	4.130979	1.211211
C	2.170427	2.777691	2.443325
H	2.319669	3.053388	3.474406
N	-1.549439	-1.762064	1.913967
N	-1.386396	-1.602640	0.575462
C	-2.145879	-2.525543	0.011609
H	-2.186723	-2.606760	-1.063784
C	-2.815597	-3.295416	0.979003
H	-3.499003	-4.112003	0.817879
C	-2.415210	-2.776977	2.180558
H	-2.677778	-3.055108	3.188083
N	0.113391	0.009197	-2.167013
C	-0.586179	0.895927	-2.867248
C	-0.549483	0.950547	-4.250503
H	-1.118078	1.677311	-4.812848
C	0.252566	0.018222	-4.896750
H	0.308117	0.022146	-5.978407
C	0.984908	-0.918727	-4.178477
C	0.879549	-0.874098	-2.798429
N	-1.366186	1.749613	-2.066736
N	-1.336094	1.594655	-0.718345
C	-2.162028	2.506138	-0.235344
H	-2.312689	2.587010	0.830219
C	-2.741861	3.265316	-1.267147
H	-3.449832	4.071859	-1.177233
C	-2.208841	2.757368	-2.420837
H	-2.368153	3.035313	-3.449799
N	1.571521	-1.735806	-1.928769
N	1.413960	-1.582511	-0.589062
C	2.171132	-2.511466	-0.032050
H	2.215654	-2.597929	1.042774
C	2.835809	-3.277875	-1.005666
H	3.517352	-4.097243	-0.850906
C	2.431184	-2.753680	-2.203376
H	2.687749	-3.029091	-3.213178
H	1.608811	-1.641510	-4.684514
H	1.112982	1.649167	4.826493
H	-0.293161	-0.032439	5.978128

$[Fe(bpp)_2]^{2+}$, pathway D; ϕ = 179.8° (minimized), θ' = 78.1° (constrained)

Fe	-0.000018	-0.003829	0.000193
N	0.008984	0.000842	-2.173338
C	-0.918575	-0.672679	-2.845937
C	-0.967274	-0.696757	-4.229741
C	0.019084	0.008775	-4.907172
C	1.000816	0.709842	-4.218021
H	1.764569	1.260505	-4.748279
C	0.942163	0.677299	-2.834967
N	-1.813363	-1.371863	-2.016296
N	-1.631035	-1.330196	-0.671740
C	-2.584870	-2.086755	-0.158258
H	-2.654879	-2.215134	0.911007
C	-3.399048	-2.633318	-1.166071
H	-4.247155	-3.286761	-1.049703
C	-2.878815	-2.154677	-2.337869
H	-3.185335	-2.313660	-3.358708
N	1.831546	1.370436	-1.994641
N	1.638712	1.321516	-0.651794
C	2.588474	2.075358	-0.126848
H	2.649786	2.198493	0.943579
C	3.411424	2.625990	-1.125291
H	4.259098	3.278093	-0.998747
C	2.901124	2.152647	-2.303606
H	3.216577	2.315723	-3.321073
N	-0.008969	0.000858	2.173073
C	-0.942205	0.677333	2.834761
C	-1.000831	0.709814	4.217822
H	-1.764595	1.260420	4.748122
C	-0.019076	0.008755	4.906946
H	-0.022963	0.012247	5.989997
C	0.967328	-0.696728	4.229531
C	0.918664	-0.672661	2.845729
N	-1.831702	1.370558	1.994569
N	-1.639101	1.321723	0.651661
C	-2.588938	2.075562	0.126925
H	-2.650439	2.198713	-0.943487
C	-3.411677	2.626245	1.125562
H	-4.259333	3.278410	0.999209
C	-2.901185	2.152847	2.303771
H	-3.216439	2.315934	3.321300
N	1.813573	-1.371902	2.016201
N	1.631350	-1.330410	0.671598
C	2.585195	-2.087035	0.158293
H	2.655263	-2.215537	-0.910950
C	3.399289	-2.633520	1.166253
H	4.247376	-3.287017	1.050044
C	2.878993	-2.154711	2.337958
H	3.185430	-2.313607	3.358836
H	1.727045	-1.244217	4.768698
H	-1.726996	-1.244268	-4.768878
H	0.022953	0.012302	-5.990219

$[Fe(bpp)_2]^{2+}$, pathway D; ϕ = 179.8° (minimized), θ' = 75.0° (constrained)

Fe	-0.004253	-0.000187	-0.000105
N	0.000237	-0.001309	-2.175759
C	-0.967269	-0.619411	-2.845488
C	-1.020056	-0.644609	-4.229050
C	0.005996	-0.002865	-4.910860
C	1.029169	0.639651	-4.225463
H	1.821841	1.145125	-4.758267
C	0.970513	0.616112	-2.842114
N	-1.893387	-1.273384	-2.013508
N	-1.677913	-1.283701	-0.673613
C	-2.666304	-1.992923	-0.158274
H	-2.719771	-2.148720	0.908359
C	-3.536596	-2.456689	-1.160629
H	-4.421876	-3.058321	-1.041829
C	-3.013648	-1.977458	-2.331030
H	-3.353108	-2.085356	-3.348143
N	1.892957	1.271395	-2.007052
N	1.670571	1.285399	-0.668238
C	2.655827	1.996744	-0.149978
H	2.703430	2.156190	0.916408
C	3.532011	2.456655	-1.149041
H	4.416932	3.058232	-1.027300
C	3.015025	1.974326	-2.320791
H	3.359770	2.079218	-3.336446
N	0.000328	0.001417	2.176253
C	-0.967316	0.619333	2.845905
C	-1.019935	0.645000	4.229491
H	-1.810515	1.150853	4.765022
C	0.006254	0.003561	4.911391
C	1.029503	-0.638912	4.226046
C	0.970814	-0.615535	2.842674
N	-1.893031	1.273641	2.013662
N	-1.677871	1.282312	0.673854
C	-2.667131	1.989888	0.157651
H	-2.721335	2.143585	-0.909235
C	-3.537558	2.454320	1.159525
H	-4.423675	3.054591	1.040080
C	-3.012212	1.979620	2.330727
H	-3.350395	2.090335	3.347959
N	1.892754	-1.271503	2.007516
N	1.670286	-1.284823	0.668837
C	2.655974	-1.995280	0.149936
H	2.703829	-2.153619	-0.916592
C	3.532651	-2.455041	1.148598
H	4.418270	-3.055490	1.026385
C	3.013945	-1.975944	2.320947
H	3.357907	-2.082642	3.336678
H	1.822230	-1.144223	4.758920
H	-1.810626	-1.150443	-4.764623
H	0.008533	0.004290	5.994468
H	0.008223	-0.003406	-5.993861

$[Fe(bpp)_2]^{2+}$, pathway D; ϕ = 180.0° (minimized), θ' = 72.2° (constrained)

Fe	-0.000254	-0.000030	0.000000
N	0.000028	0.000126	2.177215
C	-1.005318	0.555590	2.846120
C	-1.062863	0.575782	4.229472
C	0.000422	0.000352	4.913378
H	0.000578	0.000442	5.996455
C	1.063503	-0.575205	4.229260
H	1.883813	-1.033712	4.762721
C	1.005555	-0.555248	2.845922
N	-1.960481	1.165558	2.013362
N	-1.710959	1.246613	0.682248
C	-2.730707	1.910840	0.167554
H	-2.766539	2.107935	-0.892986
C	-3.656616	2.272573	1.162097
H	-4.576958	2.818833	1.041928
C	-3.132792	1.781325	2.327198
H	-3.503822	1.823609	3.338190
N	1.960441	-1.165412	2.012992
N	1.710488	-1.246791	0.682002
C	2.730042	-1.911180	0.167133
H	2.765509	-2.108522	-0.893366
C	3.656242	-2.272725	1.161425
H	4.576517	-2.819059	1.041090
C	3.132802	-1.781190	2.326575
H	3.504137	-1.823284	3.337449
N	0.000006	-0.000077	-2.177216
C	-1.005350	-0.555512	-2.846130
C	-1.062924	-0.575627	-4.229482
H	-1.883098	-1.034182	-4.763109
C	0.000340	-0.000149	-4.913379
H	0.000472	-0.000173	-5.996456
C	1.063439	0.575364	-4.229252
C	1.005521	0.555328	-2.845913
N	-1.960474	-1.165553	-2.013387
N	-1.710930	-1.246683	-0.682301
C	-2.730663	-1.910941	-0.167610
H	-2.766473	-2.108070	0.892917
C	-3.656552	-2.272671	-1.162127
H	-4.576867	-2.818972	-1.041964
C	-3.132739	-1.781380	-2.327214
H	-3.503759	-1.823668	-3.338192
N	1.960444	1.165418	-2.012967
N	1.710511	1.246722	-0.681951
C	2.730082	1.911077	-0.167077
H	2.765573	2.108383	0.893434
C	3.656305	2.272621	-1.161394
H	4.576610	2.818909	-1.041049
C	3.132851	1.781136	-2.326558
H	3.504196	1.823228	-3.337445
H	1.883739	1.033897	-4.762706
H	-1.883030	1.034358	4.763094

$[Fe(bpp)_2]^{2+}$, pathway D; $\phi = 180.0°$ (minimized), $\theta' = 69.6°$ (constrained)

Fe	0.000232	-0.000004	0.000000
N	-0.000011	-0.000088	2.175869
C	-1.038901	0.488922	2.845408
C	-1.099004	0.503502	4.228540
C	-0.000332	-0.000212	4.913115
H	-0.000458	-0.000259	5.996186
C	1.098502	-0.503859	4.228753
H	1.945811	-0.911129	4.761649
C	1.038724	-0.489149	2.845608
N	-2.022989	1.051557	2.012612
N	-1.745901	1.213189	0.694473
C	-2.796836	1.829006	0.182277
H	-2.819618	2.073665	-0.868719
C	-3.771486	2.076204	1.165243
H	-4.724708	2.562555	1.044118
C	-3.243152	1.568460	2.321318
H	-3.640704	1.536775	3.322615
N	2.023025	-1.051673	2.012994
N	1.746288	-1.213100	0.694764
C	2.797372	-1.828807	0.182739
H	2.820432	-2.073284	-0.868294
C	3.771761	-2.076155	1.165911
H	4.725023	-2.562471	1.044957
C	3.243113	-1.568610	2.321932
H	3.640402	-1.537084	3.323338
N	-0.000019	0.000102	-2.175869
C	-1.038911	-0.488902	-2.845408
C	-1.099022	-0.503460	-4.228541
H	-1.946451	-0.910689	-4.761279
C	-0.000356	0.000266	-4.913115
H	-0.000489	0.000330	-5.996185
C	1.098483	0.503900	-4.228752
C	1.038714	0.489168	-2.845608
N	-2.022992	-1.051556	-2.012618
N	-1.745905	-1.213205	-0.694489
C	-2.796831	-1.829037	-0.182296
H	-2.819601	-2.073707	0.868699
C	-3.771477	-2.076233	-1.165252
H	-4.724695	-2.562594	-1.044132
C	-3.243149	-1.568473	-2.321322
H	-3.640703	-1.536784	-3.322618
N	2.023023	1.051674	-2.012989
N	1.746284	1.213084	-0.694747
C	2.797377	1.828775	-0.182721
H	2.820449	2.073242	0.868315
C	3.771769	2.076127	-1.165903
H	4.725036	2.562432	-1.044943
C	3.243116	1.568597	-2.321928
H	3.640402	1.537075	-3.323335
H	1.945789	0.911178	-4.761646
H	-1.946430	0.910738	4.761277

Table S20 Atomic coordinates for the other DFT-minimized $[Fe(bpp^R)_2]^{2+}$ molecules in this study.

$[Fe(bpp^{NO2})_2]^{2+}$, undistorted – ϕ = 180° (minimized), θ' = 88.2° (constrained)

Fe	0.000019	0.000003	0.001614
N	0.000002	-0.000001	2.163861
C	0.828884	-0.801612	2.825759
C	0.875697	-0.846367	4.210118
C	-0.000003	0.000003	4.863361
C	-0.875707	0.846364	4.210112
H	-1.534714	1.488570	4.777002
C	-0.828889	0.801604	2.825755
N	1.629728	-1.595372	1.994297
N	1.466707	-1.511522	0.646280
C	2.331780	-2.367699	0.132455
H	2.397203	-2.491942	-0.937657
C	3.069724	-3.017627	1.139070
H	3.839973	-3.760735	1.018468
C	2.594220	-2.503626	2.313227
H	2.867838	-2.713587	3.334661
N	-1.629743	1.595351	1.994291
N	-1.466676	1.511548	0.646276
C	-2.331719	2.367750	0.132445
H	-2.397091	2.492042	-0.937664
C	-3.069707	3.017635	1.139055
H	-3.839949	3.760750	1.018451
C	-2.594291	2.503547	2.313212
H	-2.867982	2.713439	3.334640
N	-0.000003	0.000006	-2.167945
C	0.826844	0.802329	-2.830164
C	0.873957	0.847971	-4.214198
H	1.531909	1.491724	-4.780577
C	-0.000001	-0.000010	-4.867690
C	-0.873970	-0.847974	-4.214190
C	-0.826859	-0.802313	-2.830157
N	1.627441	1.595140	-1.996750
N	1.459551	1.509198	-0.649939
C	2.323264	2.363520	-0.130169
H	2.384281	2.486168	0.940370
C	3.065061	3.014555	-1.133150
H	3.835062	3.757265	-1.008585
C	2.595187	2.501294	-2.310273
H	2.873703	2.711546	-3.330344
N	-1.627465	-1.595107	-1.996735
N	-1.459522	-1.509210	-0.649927
C	-2.323199	-2.363565	-0.130152
H	-2.384159	-2.486266	0.940384
C	-3.065046	-3.014557	-1.133126
H	-3.835036	-3.757277	-1.008557
C	-2.595269	-2.501202	-2.310248
H	-2.873864	-2.711380	-3.330313
H	-1.531916	-1.491738	-4.780562
H	1.534705	-1.488568	4.777012
N	-0.000002	0.000011	6.358439
O	0.779590	-0.750272	6.891734

O	-0.779590	0.750296	6.891733
N	0.000010	-0.000029	-6.362744
O	-0.777162	-0.752762	-6.896130
O	0.777194	0.752691	-6.896135

$[Fe(bpp^{NO2})_2]^{2+}$, pathway A; ϕ = 173.0° (minimized), θ' = 85.0° (minimized)

Fe	-0.000463	0.006568	0.137619
N	-0.026870	2.162176	-0.101333
C	0.768498	2.739703	-0.995748
C	0.778915	4.108665	-1.210017
C	-0.092113	4.840868	-0.425871
C	-0.926979	4.276927	0.520074
H	-1.582664	4.902810	1.108633
C	-0.846164	2.898764	0.641936
N	1.583291	1.836438	-1.690947
N	1.484676	0.510218	-1.407734
C	2.364779	-0.085223	-2.192360
H	2.479657	-1.157871	-2.158261
C	3.048850	0.846845	-2.994443
H	3.817783	0.653776	-3.723486
C	2.525153	2.061879	-2.649144
H	2.751084	3.052882	-3.008531
N	-1.602576	2.152235	1.555048
N	-1.419007	0.805372	1.617192
C	-2.224204	0.382555	2.575490
H	-2.262400	-0.666799	2.825035
C	-2.944697	1.448345	3.145854
H	-3.670616	1.404497	3.940339
C	-2.521900	2.561427	2.473767
H	-2.802305	3.596926	2.580913
N	0.025454	-2.161275	0.110271
C	-0.744559	-2.823509	-0.746921
C	-0.751969	-4.206842	-0.827674
H	-1.362978	-4.773383	-1.515971
C	0.093348	-4.858885	0.049633
C	0.900149	-4.205368	0.961506
C	0.819938	-2.821974	0.946078
N	-1.535833	-1.992672	-1.551171
N	-1.440613	-0.644876	-1.397915
C	-2.294131	-0.129714	-2.264688
H	-2.406042	0.941047	-2.340930
C	-2.957172	-1.136306	-2.990686
H	-3.703059	-1.015350	-3.758193
C	-2.448993	-2.311438	-2.510723
H	-2.668062	-3.333016	-2.776476
N	1.551803	-1.990081	1.803643
N	1.371967	-0.643409	1.727795
C	2.152107	-0.129329	2.662096
H	2.187826	0.939487	2.807978
C	2.852029	-1.134793	3.354494
H	3.556177	-1.013921	4.160523
C	2.443198	-2.308026	2.783901
H	2.716088	-3.328129	3.001088
H	1.536003	-4.771717	1.627126

H	1.411273	4.607022	-1.931020
N	0.135474	-6.352744	0.012148
O	0.882490	-6.885090	0.795597
O	-0.580447	-6.886502	-0.798813
N	-0.130990	6.324032	-0.608524
O	0.614247	6.778684	-1.441094
O	-0.904453	6.928096	0.092896

$[Fe(bpp^{NO2})_2]^{2+}$, pathway A; ϕ = 165° (fixed), θ' = 81.7° (minimized)

Fe	0.000013	0.000050	-0.300815
N	2.150059	0.112586	-0.017348
C	2.948828	-0.650376	-0.756138
C	4.325414	-0.664672	-0.594213
C	4.817934	0.176034	0.385887
C	4.020215	0.994325	1.162885
C	2.660247	0.920637	0.905649
N	2.267891	-1.416733	-1.711663
N	0.920409	-1.270755	-1.831690
C	0.564351	-2.067215	-2.824314
H	-0.471526	-2.129438	-3.120975
C	1.676072	-2.742550	-3.361276
H	1.689890	-3.450758	-4.172687
C	2.745344	-2.299724	-2.633014
H	3.793202	-2.543853	-2.702427
N	1.696200	1.682526	1.579537
N	0.387600	1.521899	1.249822
C	-0.276315	2.369054	2.015684
H	-1.351486	2.432095	1.944898
C	0.593239	3.093955	2.851225
H	0.338403	3.850080	3.574648
C	1.843692	2.633595	2.543629
H	2.810252	2.909236	2.933654
N	-2.150091	-0.112607	-0.017382
C	-2.948880	0.650345	-0.756152
C	-4.325471	0.664585	-0.594259
C	-4.817983	-0.176167	0.385804
C	-4.020241	-0.994424	1.162816
C	-2.660273	-0.920666	0.905606
N	-2.267946	1.416770	-1.711629
N	-0.920466	1.270794	-1.831645
C	-0.564398	2.067209	-2.824303
H	0.471476	2.129402	-3.120981
C	-1.676115	2.742548	-3.361267
H	-1.689930	3.450733	-4.172699
C	-2.745381	2.299812	-2.632937
H	-3.793229	2.543990	-2.702313
N	-1.696182	-1.682484	1.579520
N	-0.387612	-1.521773	1.249765
C	0.276397	-2.368885	2.015599
H	1.351567	-2.431863	1.944762
C	-0.593082	-3.093819	2.851186
H	-0.338172	-3.849920	3.574609
C	-1.843575	-2.633498	2.543672
H	-2.810102	-2.909157	2.933767

H	5.001952	-1.271871	-1.179021
H	4.465360	1.637144	1.909112
H	-5.002016	1.271772	-1.179073
H	-4.465366	-1.637275	1.909028
N	6.294841	0.207768	0.612235
O	6.957038	-0.518484	-0.087128
O	6.686258	0.955600	1.473837
N	-6.294895	-0.207981	0.612104
O	-6.686300	-0.955834	1.473693
O	-6.957108	0.518235	-0.087282

$[Fe(bpp^{NO2})_2]^{2+}$, pathway A; ϕ = 160° (fixed), θ' = 78.6° (minimized)

Fe	-0.000034	-0.001484	-0.405374
N	2.116210	0.332367	-0.028057
C	3.017339	-0.332198	-0.743482
C	4.380104	-0.210529	-0.524869
C	4.744311	0.658064	0.486744
C	3.837354	1.382642	1.236863
C	2.504347	1.176829	0.920733
N	2.455501	-1.146738	-1.735999
N	1.109239	-1.107329	-1.932662
C	0.873653	-1.928980	-2.940967
H	-0.135688	-2.071751	-3.295828
C	2.064396	-2.510383	-3.414577
H	2.180295	-3.212851	-4.222807
C	3.052314	-1.986655	-2.627349
H	4.118575	-2.146479	-2.636939
N	1.440301	1.831827	1.555454
N	0.172346	1.558925	1.150833
C	-0.607404	2.323748	1.894661
H	-1.678553	2.289103	1.765841
C	0.145164	3.107493	2.788451
H	-0.217238	3.821237	3.509036
C	1.447757	2.771806	2.540943
H	2.362888	3.127140	2.986623
N	-2.116156	-0.332856	-0.027252
C	-3.017004	0.332522	-0.742251
C	-4.379772	0.211816	-0.523294
C	-4.744301	-0.656532	0.488416
C	-3.837620	-1.381066	1.238935
C	-2.504567	-1.176249	0.922382
N	-2.454886	1.147008	-1.734652
N	-1.108852	1.105201	-1.933076
C	-0.873290	1.925476	-2.942448
H	0.135730	2.065845	-3.299194
C	-2.063603	2.509365	-3.414188
H	-2.179340	3.211747	-4.222523
C	-3.051181	1.989306	-2.624094
H	-4.116980	2.152328	-2.631185
N	-1.440653	-1.831220	1.557404
N	-0.172815	-1.561469	1.150061
C	0.606474	-2.328504	1.892058
H	1.677381	-2.297274	1.760315
C	-0.146131	-3.109492	2.788241

H	0.216029	-3.824007	3.508181
C	-1.448198	-2.768254	2.545661
H	-2.363125	-3.119269	2.995159
H	-5.137984	0.735326	-1.088410
H	-4.185714	-2.055083	2.008839
H	4.185194	2.057096	2.006504
H	5.138533	-0.733771	-1.089930
N	6.201205	0.833304	0.776929
O	6.961928	0.190558	0.096029
O	6.479989	1.598932	1.666524
N	-6.201294	-0.831338	0.778371
O	-6.480532	-1.597438	1.667420
O	-6.961643	-0.187812	0.097796

$[Fe(bpp^{NO2})_2]^{2+}$, pathway A; $\phi = 155°$ (fixed), $\theta' = 75.0°$ (minimized)

Fe	-0.000008	-0.000015	-0.513486
N	2.119048	0.186899	-0.041889
C	2.998583	-0.528232	-0.734109
C	4.355145	-0.519424	-0.450177
C	4.737110	0.293224	0.600779
C	3.854150	1.069824	1.326955
C	2.525273	0.977914	0.943739
N	2.421246	-1.273873	-1.771329
N	1.093540	-1.120236	-2.028013
C	0.840016	-1.900586	-3.064999
H	-0.160166	-1.953527	-3.467486
C	2.000534	-2.569402	-3.496003
H	2.097192	-3.264081	-4.313464
C	2.989504	-2.142628	-2.653272
H	4.038610	-2.389019	-2.618009
N	1.485594	1.702031	1.542559
N	0.225558	1.550837	1.058187
C	-0.526668	2.370849	1.770541
H	-1.586265	2.437971	1.575546
C	0.236058	3.065798	2.726986
H	-0.104647	3.796993	3.440641
C	1.514919	2.611346	2.555888
H	2.427754	2.868713	3.068691
N	-2.119032	-0.186873	-0.041876
C	-2.998565	0.528253	-0.734107
C	-4.355125	0.519455	-0.450166
C	-4.737084	-0.293180	0.600804
C	-3.854128	-1.069782	1.326979
C	-2.525249	-0.977891	0.943756
N	-2.421231	1.273869	-1.771346
N	-1.093530	1.120214	-2.028051
C	-0.840008	1.900581	-3.065027
H	0.160172	1.953516	-3.467520
C	-2.000521	2.569420	-3.496006
H	-2.097178	3.264117	-4.313452
C	-2.989491	2.142629	-2.653282
H	-4.038594	2.389029	-2.618010
N	-1.485589	-1.702033	1.542574
N	-0.225536	-1.550896	1.058213

C	0.526638	-2.370945	1.770575
H	1.586234	-2.438115	1.575591
C	-0.236130	-3.065867	2.727008
H	0.104535	-3.797082	3.440663
C	-1.514966	-2.611358	2.555897
H	-2.427817	-2.868692	3.068686
H	-5.096151	1.086903	-0.995375
H	-4.215187	-1.698477	2.128688
H	5.096168	-1.086878	-0.995384
H	4.215204	1.698526	2.128661
N	6.186254	0.343558	0.963556
O	6.925217	-0.344083	0.303136
O	6.480710	1.064257	1.884865
N	-6.186227	-0.343501	0.963593
O	-6.480684	-1.064201	1.884899
O	-6.925187	0.344152	0.303181

$[Fe(bpp^{NO2})_2]^{2+}$, pathway A; ϕ = 150° (fixed), θ' = 71.0° (minimized)

Fe	0.000100	-0.001083	-0.618502
N	2.075856	0.365600	-0.053731
C	3.038651	-0.256434	-0.724259
C	4.376320	-0.154987	-0.375409
C	4.646936	0.651406	0.714459
C	3.674289	1.337201	1.417248
C	2.375345	1.155812	0.969192
N	2.569346	-1.008486	-1.810837
N	1.244092	-0.959928	-2.119135
C	1.102169	-1.714624	-3.195988
H	0.126900	-1.837547	-3.642131
C	2.333483	-2.260504	-3.602072
H	2.524113	-2.907828	-4.441569
C	3.246086	-1.787697	-2.699747
H	4.310586	-1.943904	-2.629245
N	1.257012	1.783138	1.533130
N	0.039718	1.560672	0.974140
C	-0.806742	2.299433	1.669968
H	-1.856535	2.299545	1.418465
C	-0.148422	3.012680	2.688379
H	-0.577866	3.693531	3.403973
C	1.167820	2.658609	2.572102
H	2.031724	2.963488	3.140558
N	-2.075734	-0.365778	-0.053729
C	-3.038062	0.257910	-0.723385
C	-4.375527	0.159089	-0.373000
C	-4.646455	-0.646616	0.717300
C	-3.674309	-1.333845	1.419408
C	-2.375528	-1.154946	0.969904
N	-2.568541	1.008787	-1.810667
N	-1.243258	0.959645	-2.118838
C	-1.101471	1.711201	-3.197849
H	-0.126251	1.832834	-3.644462
C	-2.332755	2.256381	-3.605004
H	-2.523454	2.901593	-4.446112
C	-3.245162	1.786606	-2.700906

H	-4.309549	1.943551	-2.630357
N	-1.257631	-1.783853	1.533008
N	-0.040257	-1.562246	0.973731
C	0.805340	-2.303965	1.667430
H	1.854947	-2.305434	1.415145
C	0.146502	-3.017852	2.685062
H	0.575270	-3.700785	3.399075
C	-1.169075	-2.660638	2.570915
H	-2.033029	-2.964642	3.139765
H	-5.180994	0.654031	-0.896976
H	-3.948925	-1.964746	2.252999
H	5.182132	-0.648432	-0.900262
H	3.948642	1.969023	2.250231
N	6.069717	0.801359	1.147087
O	6.888619	0.187230	0.508994
O	6.265545	1.519760	2.095950
N	-6.069047	-0.793700	1.151544
O	-6.265211	-1.511381	2.100885
O	-6.887470	-0.178236	0.514127

$[Fe(bpp^{NO2})_2]^{2+}$, pathway A; ϕ = 145° (fixed), θ' = 68.1° (minimized)

Fe	0.000006	-0.000008	-0.722529
N	2.068423	0.262811	-0.065128
C	3.019990	-0.402341	-0.709614
C	4.346039	-0.386029	-0.306304
C	4.618775	0.385022	0.808488
C	3.660387	1.116716	1.483457
C	2.372202	1.020150	0.980391
N	2.553799	-1.108822	-1.828932
N	1.246223	-0.985872	-2.189477
C	1.109402	-1.713038	-3.286160
H	0.147731	-1.777771	-3.772345
C	2.326159	-2.315334	-3.652875
H	2.517586	-2.956458	-4.496934
C	3.223571	-1.909133	-2.703768
H	4.274381	-2.123964	-2.593240
N	1.271442	1.706477	1.507311
N	0.067777	1.562146	0.896746
C	-0.761148	2.342177	1.568168
H	-1.798114	2.408181	1.275264
C	-0.104304	3.005025	2.621218
H	-0.521870	3.703053	3.327235
C	1.192343	2.574644	2.553359
H	2.049239	2.822214	3.159063
N	-2.068411	-0.262810	-0.065105
C	-3.019972	0.402355	-0.709587
C	-4.346023	0.386034	-0.306283
C	-4.618768	-0.385046	0.808486
C	-3.660391	-1.116768	1.483434
C	-2.372202	-1.020190	0.980379
N	-2.553778	1.108856	-1.828888
N	-1.246225	0.985837	-2.189499
C	-1.109425	1.712973	-3.286200
H	-0.147782	1.777629	-3.772454

C	-2.326168	2.315336	-3.652854
H	-2.517607	2.956445	-4.496921
C	-3.223536	1.909259	-2.703653
H	-4.274317	2.124186	-2.593045
N	-1.271455	-1.706525	1.507294
N	-0.067765	-1.562118	0.896803
C	0.761165	-2.342086	1.568299
H	1.798159	-2.408017	1.275480
C	0.104295	-3.004971	2.621308
H	0.521867	-3.702952	3.327368
C	-1.192392	-2.574740	2.553304
H	-2.049324	-2.822395	3.158921
H	-5.142852	0.916409	-0.808327
H	-3.937501	-1.718837	2.337308
H	5.142868	-0.916394	-0.808357
H	3.937488	1.718764	2.337350
N	-6.028771	-0.442027	1.300749
O	-6.226651	-1.127591	2.273235
O	-6.836248	0.206210	0.682027
N	6.028777	0.442001	1.300756
O	6.836249	-0.206266	0.682059
O	6.226659	1.127594	2.273221

$[Fe(bpp^{NO2})_2]^{2+}$, pathway A; ϕ = 140° (fixed), θ' = 65.4° (minimized)

Fe	0.000000	0.000000	-0.821080
N	0.304463	-2.037557	-0.071236
C	-0.341816	-3.021182	-0.686509
C	-0.323160	-4.328398	-0.226809
C	0.431629	-4.546629	0.911015
C	1.149124	-3.556065	1.553553
C	1.052413	-2.291745	0.993435
N	-1.034265	-2.611064	-1.837363
N	-0.928237	-1.317496	-2.250623
C	-1.646403	-1.236624	-3.359468
H	-1.725037	-0.295342	-3.882308
C	-2.220920	-2.477770	-3.684799
H	-2.848094	-2.714520	-4.527849
C	-1.803800	-3.330959	-2.700027
H	-1.995503	-4.381992	-2.554065
N	1.732282	-1.166794	1.473884
N	1.586654	0.006875	0.808125
C	2.367164	0.865623	1.440656
H	2.433571	1.888459	1.101721
C	3.034466	0.257137	2.519821
H	3.735482	0.705944	3.203369
C	2.605480	-1.041502	2.511388
H	2.857044	-1.870885	3.152649
N	-0.304463	2.037557	-0.071236
C	0.341816	3.021182	-0.686509
C	0.323160	4.328398	-0.226809
C	-0.431629	4.546629	0.911015
C	-1.149124	3.556065	1.553553
C	-1.052413	2.291745	0.993435
N	1.034265	2.611064	-1.837363

N	0.928237	1.317496	-2.250623
C	1.646403	1.236624	-3.359468
H	1.725037	0.295342	-3.882308
C	2.220920	2.477770	-3.684799
H	2.848094	2.714520	-4.527849
C	1.803800	3.330959	-2.700027
H	1.995503	4.381992	-2.554065
N	-1.732282	1.166794	1.473884
N	-1.586654	-0.006875	0.808125
C	-2.367164	-0.865623	1.440656
H	-2.433571	-1.888459	1.101721
C	-3.034466	-0.257137	2.519821
H	-3.735482	-0.705944	3.203369
C	-2.605480	1.041502	2.511388
H	-2.857044	1.870885	3.152649
H	0.838990	5.151826	-0.700450
H	-1.741053	3.794783	2.425862
H	-0.838990	-5.151826	-0.700450
H	1.741053	-3.794783	2.425862
N	-0.489063	5.934070	1.465899
O	-1.162063	6.085911	2.455724
O	0.146991	6.772258	0.874944
N	0.489063	-5.934070	1.465899
O	-0.146991	-6.772258	0.874944
O	1.162063	-6.085911	2.455724

$[Fe(bpp^{CN})_2]^{2+}$, undistorted − ϕ = 180° (minimized), θ' = 88.2° (constrained)

Fe	0.000000	0.000001	0.001154
N	0.000000	0.000000	2.164693
C	0.869011	-0.755546	2.828531
C	0.917175	-0.797622	4.210913
C	-0.000003	-0.000004	4.896123
C	-0.917178	0.797618	4.210913
H	-1.620186	1.414347	4.752076
C	-0.869011	0.755546	2.828531
N	1.712715	-1.504725	1.994998
N	1.542624	-1.428057	0.647603
C	2.452731	-2.233412	0.129432
H	2.521941	-2.351431	-0.941115
C	3.228284	-2.842531	1.133037
H	4.038582	-3.541288	1.009666
C	2.727348	-2.357948	2.309359
H	3.016487	-2.554842	3.328946
N	-1.712712	1.504728	1.994999
N	-1.542624	1.428058	0.647604
C	-2.452733	2.233410	0.129433
H	-2.521946	2.351425	-0.941115
C	-3.228286	2.842529	1.133038
H	-4.038585	3.541284	1.009667
C	-2.727340	2.357957	2.309360
H	-3.016474	2.554857	3.328948
N	0.000001	0.000000	-2.165535
C	0.781935	0.844663	-2.829647
C	0.825053	0.892336	-4.212004

H	1.456794	1.582069	-4.752948
C	0.000000	0.000000	-4.897501
C	-0.825053	-0.892337	-4.212003
C	-0.781933	-0.844663	-2.829646
N	1.540308	1.679763	-1.995627
N	1.378069	1.585024	-0.648758
C	2.197686	2.481563	-0.128930
H	2.253287	2.605442	0.941762
C	2.904945	3.169960	-1.131603
H	3.637332	3.949794	-1.007143
C	2.458204	2.636381	-2.308655
H	2.725171	2.863492	-3.328012
N	-1.540306	-1.679764	-1.995625
N	-1.378068	-1.585021	-0.648756
C	-2.197687	-2.481558	-0.128927
H	-2.253289	-2.605434	0.941765
C	-2.904946	-3.169956	-1.131599
H	-3.637334	-3.949788	-1.007138
C	-2.458200	-2.636384	-2.308652
H	-2.725164	-2.863498	-3.328009
H	-1.456794	-1.582070	-4.752947
H	1.620181	-1.414353	4.752076
C	-0.000005	-0.000006	6.327865
N	-0.000007	-0.000008	7.477645
C	-0.000001	0.000000	-6.329233
N	-0.000002	0.000000	-7.479012

$[Fe(bpp^{CN})_2]^{2+}$, pathway A; ϕ = 175.2° (minimized), θ' = 85.6° (minimized)

Fe	-0.000516	-0.088899	0.000574
N	2.165870	-0.001090	-0.083862
C	2.764519	0.861897	-0.897886
C	4.140687	0.986182	-0.973351
C	4.892874	0.150842	-0.146597
C	4.277147	-0.763003	0.709384
H	4.870767	-1.407582	1.341515
C	2.893380	-0.793950	0.696358
N	1.868499	1.626553	-1.660121
N	0.533911	1.437763	-1.485459
C	-0.055372	2.273786	-2.321905
H	-1.132603	2.316915	-2.372489
C	0.889732	3.015980	-3.053239
H	0.704959	3.766476	-3.803305
C	2.105541	2.578016	-2.605173
H	3.104084	2.869423	-2.887958
N	2.123919	-1.658618	1.489059
N	0.769787	-1.628739	1.367958
C	0.318223	-2.551667	2.198246
H	-0.743111	-2.727467	2.284801
C	1.373560	-3.190919	2.874558
H	1.308306	-3.977041	3.607890
C	2.509669	-2.599013	2.396396
H	3.546341	-2.777291	2.632030
N	-2.163681	0.003364	0.079649
C	-2.761968	0.864659	0.896136

C	-4.138480	0.986968	0.973017
H	-4.626903	1.689142	1.633275
C	-4.890724	0.151202	0.146607
C	-4.275309	-0.762509	-0.709476
C	-2.891337	-0.792087	-0.698592
N	-1.867192	1.629096	1.659592
N	-0.531853	1.441079	1.489061
C	0.054537	2.278316	2.326123
H	1.131602	2.322455	2.379303
C	-0.893008	3.019669	3.055205
H	-0.710674	3.770422	3.805607
C	-2.107212	2.580189	2.604389
H	-3.106685	2.870656	2.884806
N	-2.122773	-1.658049	-1.490570
N	-0.768349	-1.630413	-1.370059
C	-0.318961	-2.553278	-2.201475
H	0.742100	-2.730047	-2.289359
C	-1.375797	-3.191446	-2.876744
H	-1.312172	-3.977444	-3.610353
C	-2.510689	-2.599158	-2.396422
H	-3.547834	-2.776632	-2.630582
H	-4.869400	-1.408412	-1.339782
H	4.628708	1.689792	-1.632330
C	6.322531	0.233256	-0.177275
N	7.470049	0.300221	-0.200953
C	-6.320581	0.232157	0.179221
N	-7.468115	0.298280	0.204556

$[Fe(bpp^{CN})_2]^{2+}$, pathway A; ϕ = 165° (fixed), θ' = 81.6° (minimized)

Fe	-0.000002	0.278315	0.000003
N	2.148456	-0.005168	0.143890
C	2.959664	0.730464	-0.608612
C	4.333991	0.566716	-0.603634
C	4.845646	-0.416086	0.244770
C	4.005267	-1.184885	1.051044
C	2.648716	-0.927034	0.959305
N	2.288436	1.685380	-1.386793
N	0.940188	1.810757	-1.252406
C	0.594218	2.804753	-2.051382
H	-0.439945	3.105816	-2.120691
C	1.712955	3.336285	-2.719019
H	1.735720	4.147142	-3.427609
C	2.775034	2.600895	-2.270689
H	3.823897	2.664838	-2.511156
N	1.671623	-1.600181	1.708147
N	0.366538	-1.266023	1.529831
C	-0.312150	-2.028825	2.368155
H	-1.387726	-1.952888	2.416666
C	0.543899	-2.868135	3.104103
H	0.276158	-3.590591	3.856674
C	1.801396	-2.567484	2.658144
H	2.761467	-2.964210	2.946021
N	-2.148452	-0.005167	-0.143885
C	-2.959662	0.730467	0.608614

C	-4.333989	0.566720	0.603630
C	-4.845641	-0.416081	-0.244777
C	-4.005259	-1.184883	-1.051044
C	-2.648707	-0.927035	-0.959301
N	-2.288438	1.685383	1.386798
N	-0.940188	1.810766	1.252415
C	-0.594226	2.804764	2.051390
H	0.439936	3.105832	2.120701
C	-1.712967	3.336292	2.719025
H	-1.735737	4.147149	3.427615
C	-2.775041	2.600896	2.270695
H	-3.823904	2.664834	2.511162
N	-1.671615	-1.600185	-1.708140
N	-0.366527	-1.266027	-1.529832
C	0.312152	-2.028827	-2.368165
H	1.387727	-1.952890	-2.416686
C	-0.543903	-2.868136	-3.104108
H	-0.276168	-3.590591	-3.856682
C	-1.801395	-2.567492	-2.658132
H	-2.761467	-2.964220	-2.946000
H	4.996372	1.165170	-1.212393
H	4.414337	-1.937038	1.710108
H	-4.996373	1.165176	1.212384
H	-4.414327	-1.937035	-1.710110
C	6.260090	-0.635524	0.293212
N	7.395371	-0.813332	0.330307
C	-6.260085	-0.635516	-0.293230
N	-7.395365	-0.813318	-0.330333

$[Fe(bpp^{CN})_2]^{2+}$, pathway A; $\phi = 160°$ (fixed), $\theta' = 78.5°$ (minimized)

Fe	0.000005	0.376327	0.000032
N	2.133561	-0.001282	0.183949
C	2.987050	0.711213	-0.542943
C	4.353201	0.490469	-0.518358
C	4.809519	-0.524914	0.322972
C	3.924769	-1.266807	1.106433
C	2.581859	-0.950539	0.997251
N	2.368312	1.704527	-1.316817
N	1.029382	1.904047	-1.177267
C	0.735894	2.917860	-1.972777
H	-0.280300	3.276071	-2.036791
C	1.880667	3.389330	-2.641424
H	1.946348	4.200337	-3.347162
C	2.901657	2.593683	-2.200192
H	3.951632	2.599420	-2.444456
N	1.564897	-1.586746	1.725093
N	0.282041	-1.177749	1.545149
C	-0.443744	-1.917290	2.364917
H	-1.513770	-1.782224	2.408739
C	0.359601	-2.815715	3.090454
H	0.047119	-3.535479	3.828211
C	1.634840	-2.577642	2.656482
H	2.569685	-3.032633	2.941297
N	-2.133574	-0.001266	-0.183965

C	-2.987075	0.711224	0.542916
C	-4.353228	0.490491	0.518302
C	-4.809539	-0.524880	-0.323045
C	-3.924777	-1.266772	-1.106493
C	-2.581868	-0.950513	-0.997276
N	-2.368345	1.704525	1.316814
N	-1.029407	1.904010	1.177308
C	-0.735917	2.917819	1.972822
H	0.280284	3.276004	2.036870
C	-1.880700	3.389325	2.641426
H	-1.946385	4.200339	3.347155
C	-2.901697	2.593702	2.200163
H	-3.951681	2.599472	2.444386
N	-1.564886	-1.586723	-1.725090
N	-0.282040	-1.177742	-1.545069
C	0.443791	-1.917295	-2.364789
H	1.513822	-1.782246	-2.408542
C	-0.359521	-2.815711	-3.090372
H	-0.047003	-3.535482	-3.828106
C	-1.634788	-2.577607	-2.656491
H	-2.569623	-3.032577	-2.941369
H	-5.049629	1.071799	1.105495
H	-4.291198	-2.042080	-1.763897
H	4.291202	-2.042119	1.763825
H	5.049597	1.071779	-1.105555
C	-6.211741	-0.804689	-0.389873
N	-7.337883	-1.031134	-0.441197
C	6.211721	-0.804732	0.389775
N	7.337863	-1.031183	0.441080

$[Fe(bpp^{CN})_2]^{2+}$, pathway A; ϕ = 155° (fixed), θ' = 75.0° (minimized)

Fe	0.000000	0.476411	0.000002
N	2.113923	0.005000	0.229982
C	3.010361	0.695496	-0.465382
C	4.363899	0.408585	-0.430566
C	4.759728	-0.650656	0.387405
C	3.830158	-1.365641	1.143416
C	2.505816	-0.981014	1.027332
N	2.449108	1.737279	-1.219797
N	1.117761	1.990318	-1.097357
C	0.879100	3.028481	-1.880956
H	-0.121246	3.426582	-1.957783
C	2.053958	3.466018	-2.519202
H	2.164952	4.285740	-3.209044
C	3.035530	2.627363	-2.067698
H	4.090627	2.599733	-2.287326
N	1.450489	-1.577872	1.733044
N	0.194301	-1.094148	1.553088
C	-0.577357	-1.808410	2.353281
H	-1.638643	-1.615211	2.393266
C	0.169387	-2.763413	3.066925
H	-0.187829	-3.477621	3.789625
C	1.459366	-2.587000	2.647107
H	2.365835	-3.096858	2.930719

N	-2.113924	0.005000	-0.229981
C	-3.010362	0.695497	0.465381
C	-4.363900	0.408586	0.430564
C	-4.759728	-0.650656	-0.387406
C	-3.830157	-1.365641	-1.143416
C	-2.505816	-0.981014	-1.027332
N	-2.449109	1.737281	1.219795
N	-1.117762	1.990316	1.097360
C	-0.879101	3.028477	1.880962
H	0.121247	3.426573	1.957795
C	-2.053960	3.466018	2.519203
H	-2.164954	4.285740	3.209044
C	-3.035533	2.627370	2.067690
H	-4.090632	2.599746	2.287310
N	-1.450488	-1.577871	-1.733043
N	-0.194300	-1.094147	-1.553085
C	0.577359	-1.808409	-2.353277
H	1.638646	-1.615211	-2.393259
C	-0.169385	-2.763412	-3.066923
H	0.187833	-3.477618	-3.789623
C	-1.459364	-2.586997	-2.647108
H	-2.365833	-3.096853	-2.930723
H	-5.095110	0.970782	0.993538
H	-4.149460	-2.173118	-1.786560
H	5.095109	0.970781	-0.993540
H	4.149461	-2.173118	1.786559
C	6.145248	-1.001820	0.460731
N	7.257851	-1.286812	0.516105
C	-6.145248	-1.001820	-0.460733
N	-7.257851	-1.286813	-0.516108

$[Fe(bpp^{CN})_2]^{2+}$, pathway A; $\phi = 150°$ (fixed), $\theta' = 71.0°$ (minimized)

Fe	0.000000	0.000000	0.576052
N	2.089172	-0.278891	0.011294
C	3.026233	0.382825	0.680249
C	4.364621	0.338955	0.329984
C	4.699359	-0.454231	-0.768853
C	3.727125	-1.178208	-1.460220
C	2.423784	-1.054865	-1.011547
N	2.524872	1.114037	1.768731
N	1.202615	1.011197	2.075437
C	1.027588	1.757578	3.153160
H	0.047166	1.840169	3.597188
C	2.234685	2.352454	3.562241
H	2.397339	3.005815	4.402941
C	3.166946	1.919046	2.660075
H	4.223909	2.120191	2.592754
N	1.331811	-1.730736	-1.574746
N	0.105730	-1.555400	-1.018621
C	-0.710729	-2.325635	-1.716137
H	-1.760552	-2.363822	-1.467636
C	-0.023392	-3.014897	-2.731704
H	-0.424959	-3.712246	-3.447575
C	1.278310	-2.611745	-2.611329

H	2.153776	-2.886256	-3.177284
N	-2.089172	0.278891	0.011294
C	-3.026233	-0.382825	0.680249
C	-4.364621	-0.338955	0.329984
C	-4.699359	0.454231	-0.768853
C	-3.727125	1.178208	-1.460220
C	-2.423784	1.054865	-1.011547
N	-2.524872	-1.114037	1.768731
N	-1.202615	-1.011197	2.075437
C	-1.027588	-1.757578	3.153160
H	-0.047166	-1.840169	3.597188
C	-2.234685	-2.352454	3.562241
H	-2.397339	-3.005815	4.402941
C	-3.166946	-1.919046	2.660075
H	-4.223909	-2.120191	2.592754
N	-1.331811	1.730736	-1.574746
N	-0.105730	1.555400	-1.018621
C	0.710729	2.325635	-1.716137
H	1.760552	2.363822	-1.467636
C	0.023392	3.014897	-2.731704
H	0.424959	3.712246	-3.447575
C	-1.278310	2.611745	-2.611329
H	-2.153776	2.886256	-3.177284
H	-5.129848	-0.875298	0.872594
H	-4.000468	1.804270	-2.297424
H	5.129848	0.875298	0.872594
H	4.000468	-1.804270	-2.297424
C	-6.066723	0.534882	-1.186599
N	-7.163841	0.595524	-1.525003
C	6.066723	-0.534882	-1.186599
N	7.163841	-0.595524	-1.525003

$[Fe(bpp^{CN})_2]^{2+}$, pathway A; $\phi = 145°$ (fixed), $\theta' = 68.2°$ (minimized)

Fe	0.000000	0.000000	-0.672162
N	2.059690	0.318807	-0.015010
C	3.030209	-0.319418	-0.658352
C	4.353000	-0.268826	-0.253860
C	4.635364	0.508201	0.871010
C	3.628671	1.211186	1.533715
C	2.345369	1.082814	1.030696
N	2.583223	-1.036731	-1.780543
N	1.272176	-0.952493	-2.137902
C	1.152722	-1.683347	-3.234126
H	0.191436	-1.776854	-3.716323
C	2.385318	-2.249214	-3.604853
H	2.593202	-2.884624	-4.449314
C	3.273832	-1.813084	-2.660449
H	4.331273	-1.995267	-2.556486
N	1.225466	1.741717	1.556153
N	0.026424	1.561859	0.946249
C	-0.825016	2.319381	1.615048
H	-1.863528	2.353498	1.322298
C	-0.187609	3.003959	2.665914
H	-0.624823	3.691884	3.369927

C	1.121129	2.611743	2.598201
H	1.969499	2.887621	3.203374
N	-2.059690	-0.318807	-0.015010
C	-3.030210	0.319418	-0.658352
C	-4.353001	0.268826	-0.253860
C	-4.635364	-0.508201	0.871010
C	-3.628672	-1.211186	1.533715
C	-2.345369	-1.082814	1.030696
N	-2.583223	1.036731	-1.780543
N	-1.272176	0.952493	-2.137902
C	-1.152722	1.683347	-3.234126
H	-0.191436	1.776854	-3.716323
C	-2.385318	2.249214	-3.604854
H	-2.593202	2.884624	-4.449315
C	-3.273832	1.813084	-2.660450
H	-4.331273	1.995266	-2.556487
N	-1.225466	-1.741717	1.556153
N	-0.026424	-1.561858	0.946249
C	0.825016	-2.319381	1.615047
H	1.863528	-2.353497	1.322298
C	0.187609	-3.003958	2.665914
H	0.624824	-3.691884	3.369927
C	-1.121129	-2.611743	2.598201
H	-1.969498	-2.887621	3.203374
H	-5.145221	0.787475	-0.774515
H	-3.862230	-1.826951	2.390386
H	5.145221	-0.787475	-0.774514
H	3.862230	1.826951	2.390386
C	5.983875	0.595105	1.345356
N	7.065587	0.660310	1.729488
C	-5.983875	-0.595105	1.345356
N	-7.065587	-0.660310	1.729488

$[Fe(bpp^{CN})_2]^{2+}$, pathway A; ϕ = 140° (fixed), θ' = 65.5° (minimized)

Fe	0.000000	0.000000	-0.766597
N	-2.026125	-0.365492	-0.017245
C	-3.028963	0.252705	-0.629995
C	-4.333329	0.194646	-0.170971
C	-4.560434	-0.569408	0.975324
C	-3.520717	-1.254379	1.604681
C	-2.261013	-1.120984	1.046356
N	-2.637786	0.960772	-1.779792
N	-1.341953	0.890311	-2.193600
C	-1.281775	1.607541	-3.303978
H	-0.343550	1.709236	-3.828237
C	-2.537485	2.149601	-3.628180
H	-2.791486	2.769306	-4.471698
C	-3.376753	1.715722	-2.638925
H	-4.431195	1.884569	-2.491174
N	-1.115207	-1.766906	1.528201
N	0.053948	-1.584294	0.863887
C	0.936169	-2.339160	1.494746
H	1.960533	-2.373101	1.155602
C	0.349054	-3.023991	2.574515

H	0.819427	-3.710485	3.258290
C	-0.961552	-2.632896	2.567663
H	-1.780723	-2.908671	3.211826
N	2.026125	0.365492	-0.017245
C	3.028963	-0.252705	-0.629995
C	4.333329	-0.194646	-0.170971
C	4.560434	0.569408	0.975324
C	3.520717	1.254379	1.604681
C	2.261013	1.120984	1.046356
N	2.637786	-0.960772	-1.779792
N	1.341953	-0.890311	-2.193600
C	1.281775	-1.607541	-3.303978
H	0.343550	-1.709236	-3.828237
C	2.537485	-2.149601	-3.628180
H	2.791486	-2.769306	-4.471698
C	3.376753	-1.715721	-2.638925
H	4.431195	-1.884569	-2.491174
N	1.115207	1.766906	1.528201
N	-0.053948	1.584294	0.863887
C	-0.936169	2.339160	1.494746
H	-1.960533	2.373101	1.155602
C	-0.349054	3.023991	2.574515
H	-0.819427	3.710485	3.258290
C	0.961552	2.632896	2.567663
H	1.780723	2.908671	3.211826
H	5.152338	-0.696901	-0.665461
H	3.712515	1.861803	2.477452
H	-5.152338	0.696901	-0.665461
H	-3.712515	-1.861803	2.477452
C	5.887271	0.662844	1.507002
N	6.951148	0.733113	1.937009
C	-5.887271	-0.662844	1.507002
N	-6.951148	-0.733113	1.937009

$[Fe(bpp^{NMe2})_2]^{2+}$, undistorted – ϕ = 179.9° (minimized), θ' = 89.7° (constrained)

Fe	-0.003566	0.000316	0.000008
N	-0.001375	-0.000489	2.153690
C	0.804468	0.809181	2.847473
C	0.857575	0.857940	4.215217
C	0.001184	-0.001482	4.957128
C	-0.856744	-0.860189	4.216178
H	-1.533163	-1.534874	4.715446
C	-0.805878	-0.810751	2.848352
N	1.605552	1.622172	2.019499
N	1.490264	1.489644	0.677713
C	2.333103	2.361114	0.156407
H	2.425387	2.446857	-0.915257
C	3.012970	3.073590	1.162260
H	3.756624	3.842856	1.040822
C	2.522407	2.575038	2.338237
H	2.756029	2.826599	3.359143
N	-1.608631	-1.623061	2.021355
N	-1.498124	-1.487640	0.679434
C	-2.342995	-2.357786	0.159232

H	-2.439312	-2.441036	-0.912273
C	-3.019217	-3.072517	1.165970
H	-3.763353	-3.841468	1.045491
C	-2.524075	-2.576932	2.341268
H	-2.753698	-2.831066	3.362443
N	-0.000528	0.000015	-2.153704
C	-0.805799	0.809174	-2.848832
C	-0.856117	0.858409	-4.216612
H	-1.531033	1.534270	-4.716272
C	0.003073	0.000368	-4.956898
C	0.860727	-0.857558	-4.214615
C	0.806392	-0.809027	-2.846996
N	-1.610033	1.620535	-2.022340
N	-1.499049	1.486516	-0.680308
C	-2.345224	2.355797	-0.160804
H	-2.441336	2.440037	0.910643
C	-3.022917	3.068406	-1.168054
H	-3.768231	3.836299	-1.048116
C	-2.527905	2.571864	-2.342977
H	-2.758806	2.824042	-3.364346
N	1.608012	-1.620884	-2.018402
N	1.492537	-1.487366	-0.676721
C	2.336555	-2.357268	-0.154669
H	2.429005	-2.441924	0.917066
C	3.017287	-3.069756	-1.159914
H	3.761872	-3.838022	-1.037845
C	2.525971	-2.572936	-2.336327
H	2.759699	-2.825252	-3.357025
H	1.537795	-1.532368	-4.712801
H	1.533343	1.533825	4.713742
N	0.004531	0.000030	-6.296533
N	0.002386	-0.001862	6.296322
C	0.889833	0.890377	7.035490
H	1.938854	0.681300	6.807498
H	0.742072	0.735008	8.100502
H	0.670117	1.938867	6.814503
C	-0.886812	-0.891398	7.036637
H	-0.730281	-0.743411	8.101448
H	-0.675599	-1.939925	6.808280
H	-1.936099	-0.674121	6.817098
C	-0.886599	0.886804	-7.037821
H	-0.744588	0.722601	-8.102297
H	-0.666417	1.937258	-6.826427
H	-1.934164	0.678793	-6.802774
C	0.900917	-0.884000	-7.034721
H	1.947596	-0.667776	-6.802713
H	0.755442	-0.727212	-8.099836
H	0.688090	-1.934544	-6.816761

$[Fe(bpp^{NMe2})_2]^{2+}$, pathway A; ϕ = 174.2° (minimized), θ' = 88.5° (minimized)

Fe	0.170812	0.000029	0.000009
N	0.062356	-0.075081	2.151418
C	-0.786945	-0.895873	2.774195
C	-0.932506	-0.969393	4.133647

C	-0.126163	-0.124094	4.945175
C	0.785983	0.740273	4.279572
H	1.434431	1.396946	4.836540
C	0.828756	0.713023	2.910269
N	-1.526868	-1.694336	1.876188
N	-1.281800	-1.569411	0.551233
C	-2.082528	-2.432282	-0.046757
H	-2.071789	-2.520214	-1.122278
C	-2.862245	-3.133635	0.891002
H	-3.599191	-3.895880	0.702555
C	-2.478230	-2.636841	2.107996
H	-2.807839	-2.884551	3.103289
N	1.701903	1.519651	2.152258
N	1.702282	1.384856	0.805316
C	2.618483	2.223104	0.359911
H	2.809061	2.299351	-0.699402
C	3.229135	2.918281	1.420319
H	4.006304	3.661413	1.363658
C	2.620297	2.441939	2.548946
H	2.773893	2.687693	3.586321
N	0.062400	0.075188	-2.151402
C	0.828833	-0.712872	-2.910269
C	0.786092	-0.740063	-4.279576
H	1.434598	-1.396654	-4.836591
C	-0.126051	0.124321	-4.945158
C	-0.932413	0.969582	-4.133623
C	-0.786913	0.895976	-2.774168
N	1.702003	-1.519486	-2.152270
N	1.702357	-1.384733	-0.805323
C	2.618580	-2.222960	-0.359926
H	2.809146	-2.299230	0.699388
C	3.229284	-2.918070	-1.420349
H	4.006491	-3.661164	-1.363699
C	2.620448	-2.441716	-2.548973
H	2.774080	-2.687421	-3.586355
N	-1.526921	1.694347	-1.876145
N	-1.281912	1.569357	-0.551185
C	-2.082758	2.432112	0.046824
H	-2.072094	2.519967	1.122354
C	-2.862499	3.133447	-0.890927
H	-3.599543	3.895596	-0.702464
C	-2.478360	2.636779	-2.107939
H	-2.807946	2.884514	-3.103233
H	-1.635934	1.656812	-4.574827
H	-1.636046	-1.656607	4.574844
N	-0.218808	-0.146696	6.281551
N	-0.218620	0.147052	-6.281499
C	0.621275	-0.726286	-7.096215
H	0.397179	-0.556176	-8.145523
H	0.425347	-1.780088	-6.876722
H	1.682608	-0.511749	-6.939908
C	-1.161724	1.043860	-6.941859
H	-2.191819	0.818062	-6.649635
H	-1.082280	0.911647	-8.017758

H	-0.938450	2.090545	-6.713670
C	-1.162165	-1.043455	6.941768
H	-2.192268	-0.817396	6.649524
H	-1.082694	-0.911337	8.017682
H	-0.939079	-2.090227	6.713500
C	0.621783	0.725525	7.096203
H	0.397305	0.556215	8.145472
H	0.427545	1.779065	6.876371
H	1.682348	0.509450	6.940331

$[Fe(bpp^{NMe2})_2]^{2+}$, pathway A; $\phi = 165°$ (fixed), $\theta' = 85.5°$ (minimized)

Fe	0.001050	-0.000312	0.442332
N	-0.373412	-2.102726	0.161426
C	0.304705	-3.017011	0.859258
C	0.170800	-4.370303	0.690079
C	-0.753888	-4.842282	-0.281838
C	-1.486186	-3.865027	-1.011350
C	-1.252552	-2.542637	-0.741834
N	1.176081	-2.443620	1.808728
N	1.193125	-1.095399	1.941633
C	2.041945	-0.840963	2.920136
H	2.229774	0.178776	3.218940
C	2.592211	-2.029352	3.436149
H	3.308933	-2.135232	4.232841
C	2.014726	-3.030882	2.704163
H	2.136409	-4.099767	2.758677
N	-1.932006	-1.490617	-1.391598
N	-1.645976	-0.220013	-1.025562
C	-2.437281	0.549855	-1.749578
H	-2.394707	1.622220	-1.636308
C	-3.251472	-0.218958	-2.601433
H	-3.991198	0.128888	-3.302380
C	-2.899902	-1.517249	-2.345061
H	-3.267114	-2.439826	-2.762648
N	0.374257	2.102634	0.160909
C	-0.303508	3.016251	0.860061
C	-0.173540	4.369510	0.688611
C	0.747546	4.841983	-0.286599
C	1.481840	3.865738	-1.015493
C	1.251550	2.543371	-0.743769
N	-1.170552	2.441921	1.812947
N	-1.186680	1.093631	1.945004
C	-2.033432	0.838191	2.925060
H	-2.220801	-0.181894	3.222979
C	-2.581851	2.026051	3.444162
H	-3.296313	2.131100	4.242986
C	-2.004589	3.028425	2.713184
H	-2.124465	4.097361	2.770571
N	1.932155	1.492097	-1.393470
N	1.644281	0.220949	-1.030789
C	2.432279	-0.548357	-1.759035
H	2.386483	-1.621047	-1.650295
C	3.247789	0.221489	-2.608690
H	3.985674	-0.125663	-3.311924

C	2.902828	1.519905	-2.344118
H	3.274410	2.443209	-2.756215
H	0.743643	-5.057855	1.291045
H	-2.214727	-4.155021	-1.751164
H	-0.747013	5.056707	1.289325
H	2.207965	4.156432	-1.757342
N	-0.935302	-6.152194	-0.493847
N	0.923175	6.152814	-0.503041
C	-1.888082	-6.613755	-1.498324
H	-1.873322	-7.699644	-1.528887
H	-1.622301	-6.245850	-2.493303
H	-2.905963	-6.297652	-1.252136
C	-0.169605	-7.137060	0.263432
H	-0.453168	-8.133834	-0.062671
H	-0.378717	-7.061508	1.334449
H	0.904214	-7.019803	0.092249
C	1.882859	6.615505	-1.500336
H	1.624243	6.247397	-2.497127
H	2.899359	6.300631	-1.247232
H	1.866721	7.701377	-1.531018
C	0.165616	7.136542	0.263725
H	0.385733	7.059636	1.332410
H	-0.909831	7.018909	0.103347
H	0.445080	8.133809	-0.064403

$[Fe(bpp^{NMe2})_2]^{2+}$, pathway A; ϕ = 160° (fixed), θ' = 82.1° (minimized)

Fe	0.000432	-0.000833	-0.582513
N	2.096348	0.343115	-0.207899
C	3.027559	-0.336390	-0.880227
C	4.374406	-0.228393	-0.653105
C	4.819015	0.669363	0.356324
C	3.824277	1.408378	1.054511
C	2.511302	1.202925	0.724166
N	2.478502	-1.181514	-1.867690
N	1.139555	-1.150405	-2.075543
C	0.910684	-1.982359	-3.075093
H	-0.096869	-2.131859	-3.431545
C	2.106383	-2.566913	-3.532004
H	2.231838	-3.279108	-4.329921
C	3.084735	-2.032551	-2.738058
H	4.150185	-2.191470	-2.734371
N	1.442874	1.892276	1.335505
N	0.187199	1.644514	0.897034
C	-0.601182	2.438835	1.597677
H	-1.666891	2.423775	1.427655
C	0.139838	3.216288	2.506320
H	-0.227114	3.949545	3.204323
C	1.441309	2.837945	2.311144
H	2.348704	3.173009	2.785261
N	-2.096576	-0.345694	-0.207893
C	-3.027216	0.333245	-0.881416
C	-4.374355	0.226949	-0.653208
C	-4.820181	-0.669677	0.356585
C	-3.825623	-1.407933	1.055474

C	-2.512046	-1.203204	0.725808
N	-2.477668	1.173508	-1.872698
N	-1.138698	1.142985	-2.078189
C	-0.908587	1.973850	-3.078571
H	0.099607	2.124201	-3.432778
C	-2.104058	2.555564	-3.539540
H	-2.228779	3.266036	-4.339092
C	-3.083859	2.017505	-2.749930
H	-4.149934	2.172193	-2.751205
N	-1.444423	-1.892232	1.338607
N	-0.187870	-1.641697	0.904704
C	0.600518	-2.431020	1.611085
H	1.666952	-2.412049	1.446278
C	-0.141805	-3.209138	2.518111
H	0.224656	-3.939013	3.219915
C	-1.444411	-2.840820	2.311464
H	-2.353134	-3.181448	2.779048
H	-5.076945	0.795550	-1.240778
H	-4.096630	-2.119785	1.818489
H	4.094817	2.122352	1.815688
H	5.077351	-0.798189	-1.238983
N	-6.122108	-0.823153	0.629119
N	6.122159	0.820776	0.631374
C	-6.556293	-1.766926	1.654763
H	-6.150042	-1.499860	2.634477
H	-6.258686	-2.788855	1.402276
H	-7.640360	-1.741679	1.724481
C	-7.127681	-0.061814	-0.105954
H	-7.113041	-0.311374	-1.171064
H	-6.975645	1.013913	0.018985
H	-8.112653	-0.306487	0.282275
C	7.128277	0.073725	-0.117315
H	8.114275	0.327587	0.262226
H	7.100416	0.327565	-1.181041
H	6.989017	-1.004152	0.004546
C	6.556912	1.769875	1.651711
H	7.640068	1.734726	1.730757
H	6.140578	1.513250	2.629829
H	6.270616	2.792670	1.389629

$[Fe(bpp^{NMe2})_2]^{2+}$, pathway A; ϕ = 155° (fixed), θ' = 78.4° (minimized)

Fe	-0.000115	-0.735628	0.001612
N	2.066335	-0.269399	-0.409748
C	3.043101	-0.914777	0.230070
C	4.375272	-0.620871	0.098836
C	4.750027	0.433252	-0.779890
C	3.706611	1.100785	-1.479191
C	2.415633	0.701800	-1.254317
N	2.559448	-1.950373	1.058167
N	1.230602	-2.217185	1.052127
C	1.066496	-3.246748	1.863554
H	0.079342	-3.650841	2.027393
C	2.295160	-3.664234	2.407494
H	2.472867	-4.473227	3.095669

C	3.224871	-2.817856	1.866298
H	4.293358	-2.773565	1.996194
N	1.303656	1.272495	-1.908188
N	0.078507	0.762315	-1.647268
C	-0.762196	1.440720	-2.406346
H	-1.817212	1.215332	-2.373780
C	-0.086066	2.405825	-3.175040
H	-0.503669	3.101320	-3.883157
C	1.232381	2.266586	-2.831500
H	2.107151	2.793636	-3.174274
N	-2.066363	-0.267804	0.410224
C	-3.043047	-0.913735	-0.229145
C	-4.375294	-0.620335	-0.097767
C	-4.750166	0.433678	0.781061
C	-3.706844	1.102261	1.479501
C	-2.415806	0.703862	1.254210
N	-2.559191	-1.949768	-1.056589
N	-1.230724	-2.219255	-1.046885
C	-1.067030	-3.251590	-1.854709
H	-0.080477	-3.658793	-2.014509
C	-2.295245	-3.667427	-2.401029
H	-2.473042	-4.477921	-3.087416
C	-3.224145	-2.815950	-1.866470
H	-4.291950	-2.768329	-2.000750
N	-1.303799	1.275467	1.907282
N	-0.078770	0.764354	1.647581
C	0.761872	1.443584	2.406010
H	1.816803	1.217646	2.374272
C	0.085917	2.410417	3.172655
H	0.503587	3.107020	3.879640
C	-1.232419	2.271316	2.828668
H	-2.107047	2.799608	3.169883
H	-5.119984	-1.187457	-0.632577
H	-3.924470	1.890361	2.182058
H	5.120000	-1.187624	0.633927
H	3.924083	1.889072	-2.181610
N	6.034653	0.772656	-0.953435
N	-6.035118	0.771677	0.955989
C	6.397999	1.851487	-1.866527
H	7.475369	1.989347	-1.841161
H	5.932078	2.794515	-1.567632
H	6.112620	1.613179	-2.895411
C	7.089947	0.062917	-0.237797
H	8.050601	0.496763	-0.500880
H	7.111742	-0.995678	-0.512771
H	6.964523	0.156048	0.844602
C	-7.089681	0.064665	0.236546
H	-7.107708	-0.995853	0.504197
H	-6.966215	0.165665	-0.845447
H	-8.051261	0.493373	0.504582
C	-6.397949	1.857408	1.861055
H	-5.931689	2.798075	1.555376
H	-6.112632	1.626404	2.891622
H	-7.475288	1.995332	1.834858

[Fe(bpp^{NMe2})$_2$]$^{2+}$, pathway A; ϕ = 150° (fixed), θ' = 74.8° (minimized)

Fe	0.000041	-0.001232	-0.888203
N	2.052494	0.380123	-0.328512
C	3.043314	-0.262307	-0.948363
C	4.362650	-0.175400	-0.588003
C	4.707492	0.660765	0.510096
C	3.650581	1.365660	1.149633
C	2.374937	1.185888	0.683438
N	2.589717	-1.046836	-2.031323
N	1.271362	-1.016671	-2.346235
C	1.137218	-1.788636	-3.410368
H	0.163733	-1.927636	-3.855034
C	2.374797	-2.330218	-3.802160
H	2.576024	-2.989531	-4.629462
C	3.277726	-1.831905	-2.901707
H	4.342633	-1.974754	-2.823863
N	1.252884	1.849098	1.221112
N	0.049238	1.636409	0.642633
C	-0.806220	2.390235	1.308150
H	-1.849646	2.391565	1.031938
C	-0.161102	3.108177	2.331983
H	-0.595999	3.801978	3.031305
C	1.154307	2.737510	2.244373
H	2.009913	3.039433	2.825125
N	-2.052701	-0.382428	-0.329096
C	-3.043092	0.261476	-0.948078
C	-4.362229	0.176500	-0.586529
C	-4.707255	-0.659159	0.511904
C	-3.650767	-1.365271	1.150823
C	-2.375303	-1.187368	0.683444
N	-2.589342	1.044949	-2.031721
N	-1.270832	1.015216	-2.345850
C	-1.136635	1.785606	-3.411129
H	-0.162996	1.924578	-3.855461
C	-2.374346	2.325827	-3.804375
H	-2.575590	2.983735	-4.632788
C	-3.277460	1.827993	-2.903857
H	-4.342556	1.969957	-2.827013
N	-1.253707	-1.852240	1.220003
N	-0.049964	-1.639622	0.641730
C	0.804892	-2.395612	1.305567
H	1.848246	-2.397462	1.029115
C	0.159294	-3.114767	2.328246
H	0.593659	-3.810292	3.026185
C	-1.155809	-2.742820	2.241448
H	-2.011641	-3.045202	2.821628
H	-5.119721	0.714551	-1.133211
H	-3.847909	-2.037771	1.970013
H	5.120461	-0.712277	-1.135419
H	3.847485	2.038661	1.968479
N	5.977941	0.789046	0.916224
N	-5.977701	-0.786167	0.918737
C	6.312870	1.672518	2.028412

H	7.381516	1.616861	2.216317
H	5.795500	1.367801	2.942355
H	6.064235	2.712693	1.797889
C	7.050246	0.081981	0.223528
H	7.994813	0.301901	0.713274
H	7.127963	0.402888	-0.819413
H	6.896959	-1.000157	0.261734
C	-6.312239	-1.664813	2.034840
H	-5.792515	-1.358010	2.946737
H	-6.066215	-2.706305	1.807481
H	-7.380429	-1.606310	2.224480
C	-7.049176	-0.074257	0.229763
H	-7.129353	-0.392273	-0.813860
H	-6.893176	1.007396	0.270524
H	-7.993513	-0.293259	0.720367

$[Fe(bpp^{NMe2})_2]^{2+}$, pathway A; ϕ = 145° (fixed), θ' = 72.5° (minimized)

Fe	-0.000897	-0.001872	-1.027843
N	2.032257	0.357974	-0.378473
C	3.034658	-0.298836	-0.963523
C	4.338760	-0.248538	-0.545493
C	4.655382	0.567936	0.575975
C	3.588160	1.290730	1.177022
C	2.329646	1.146013	0.654570
N	2.612585	-1.060062	-2.076707
N	1.307927	-1.009273	-2.441384
C	1.204735	-1.765263	-3.520817
H	0.246808	-1.887575	-4.002679
C	2.449659	-2.315492	-3.873455
H	2.674749	-2.964820	-4.702478
C	3.323746	-1.839214	-2.933220
H	4.383720	-1.994101	-2.819336
N	1.202610	1.832492	1.148741
N	0.020821	1.652976	0.517556
C	-0.844221	2.421206	1.153186
H	-1.874934	2.450030	0.833879
C	-0.226809	3.116220	2.209659
H	-0.674614	3.815393	2.895371
C	1.081916	2.715011	2.174924
H	1.918944	2.992449	2.793706
N	-2.032177	-0.357585	-0.375999
C	-3.035047	0.297318	-0.962505
C	-4.339271	0.246605	-0.545248
C	-4.655697	-0.569287	0.576614
C	-3.588017	-1.288901	1.180608
C	-2.329309	-1.143185	0.659137
N	-2.613139	1.058145	-2.076075
N	-1.309484	1.001553	-2.444980
C	-1.208017	1.751245	-3.528722
H	-0.251781	1.866914	-4.015617
C	-2.452052	2.305695	-3.878248
H	-2.677824	2.952191	-4.709313
C	-3.323317	1.841843	-2.929226
H	-4.381360	2.005123	-2.809281

N	-1.201103	-1.825055	1.157364
N	-0.019893	-1.648425	0.523771
C	0.845018	-2.415598	1.160598
H	1.875016	-2.447276	0.839074
C	0.228937	-3.105066	2.221504
H	0.677321	-3.801877	2.909233
C	-1.078429	-2.699225	2.190375
H	-1.913870	-2.970357	2.814067
H	-5.106813	0.794117	-1.067867
H	-3.765171	-1.948308	2.014823
H	5.105967	-0.797752	-1.066828
H	3.765272	1.952241	2.009583
N	-5.910520	-0.665847	1.037050
N	5.909651	0.662605	1.037761
C	-6.996210	0.054146	0.379912
H	-7.120847	-0.274824	-0.655944
H	-6.822263	1.133616	0.401291
H	-7.924449	-0.143971	0.908425
C	-6.216059	-1.530826	2.171650
H	-5.656028	-1.226352	3.060165
H	-5.996478	-2.577957	1.943216
H	-7.274820	-1.453009	2.402349
C	6.216059	1.527945	2.171909
H	7.272420	1.440102	2.409826
H	5.647712	1.231418	3.057789
H	6.008110	2.576616	1.939448
C	6.995663	-0.057387	0.381048
H	7.922786	0.137318	0.912818
H	7.124028	0.274518	-0.653398
H	6.819877	-1.136588	0.398661

$[Fe(bpp^{NMe2})_2]^{2+}$, pathway A; ϕ = 140° (fixed), θ' = 70.4° (minimized)

Fe	-0.000962	-0.004659	-1.162958
N	2.009447	0.335826	-0.422349
C	3.022035	-0.339297	-0.966977
C	4.307412	-0.321669	-0.492272
C	4.593627	0.482699	0.645940
C	3.517850	1.227311	1.202632
C	2.280465	1.114124	0.624446
N	2.634167	-1.085366	-2.104001
N	1.347870	-1.009318	-2.526032
C	1.279600	-1.755984	-3.615233
H	0.341684	-1.859459	-4.139103
C	2.529136	-2.324580	-3.916853
H	2.779728	-2.971230	-4.740636
C	3.368853	-1.869198	-2.935674
H	4.420450	-2.041855	-2.777978
N	1.151702	1.831668	1.065689
N	-0.002218	1.685617	0.377600
C	-0.871958	2.484797	0.967205
H	-1.884499	2.546491	0.598181
C	-0.285295	3.166278	2.049816
H	-0.743475	3.883755	2.709291
C	1.009858	2.723459	2.081189

H	1.824253	2.978000	2.738799
N	-2.009363	-0.337344	-0.420481
C	-3.021163	0.338887	-0.965108
C	-4.306269	0.324368	-0.489476
C	-4.593550	-0.478890	0.649173
C	-3.518660	-1.224493	1.206173
C	-2.281156	-1.113596	0.627700
N	-2.632489	1.084010	-2.102483
N	-1.348102	0.998233	-2.530273
C	-1.281330	1.737043	-3.624562
H	-0.345969	1.830410	-4.154933
C	-2.528526	2.313261	-3.921893
H	-2.779246	2.956796	-4.748090
C	-3.364338	1.875287	-2.929496
H	-4.412601	2.060478	-2.764004
N	-1.152342	-1.830058	1.071140
N	0.000837	-1.688207	0.380439
C	0.869440	-2.488189	0.970350
H	1.880964	-2.553594	0.599003
C	0.283480	-3.164707	2.056479
H	0.741337	-3.881348	2.717080
C	-1.009512	-2.715902	2.091606
H	-1.822394	-2.964244	2.753442
H	-5.081913	0.888447	-0.981586
H	-3.674324	-1.879066	2.048404
H	5.083617	-0.885329	-0.983826
H	3.672795	1.883689	2.043560
N	-5.828445	-0.541493	1.165486
N	5.829377	0.548219	1.161143
C	6.926002	-0.194344	0.548590
H	7.834099	-0.020177	1.118883
H	7.104487	0.136073	-0.478884
H	6.726725	-1.269617	0.556275
C	6.105656	1.400878	2.312590
H	7.149247	1.290131	2.593430
H	5.494938	1.111380	3.172247
H	5.928815	2.454817	2.078136
C	-6.106626	-1.398847	2.313020
H	-5.501551	-1.108639	3.176499
H	-5.923400	-2.451219	2.076522
H	-7.152185	-1.294392	2.588925
C	-6.924832	0.200621	0.551979
H	-7.106202	-0.133222	-0.473973
H	-6.722802	1.275373	0.555335
H	-7.832255	0.031105	1.124735

$[Fe(bpp^{SMe})_2]^{2+}$, undistorted − ϕ = 180.0° (minimized), θ' = 88.1° (constrained)

Fe	-0.000437	0.000215	-0.173030
N	-2.148223	-0.066619	-0.179810
C	-2.805777	-0.881810	0.636459
C	-4.182986	-0.971183	0.677614
C	-4.914731	-0.150350	-0.192999
C	-4.219176	0.716731	-1.057289
H	-4.762222	1.364550	-1.731836

C	-2.846537	0.712507	-1.006667
N	-1.952779	-1.656642	1.447569
N	-0.609751	-1.537157	1.287881
C	-0.069869	-2.374547	2.155266
H	1.003548	-2.465257	2.219384
C	-1.058316	-3.051531	2.891489
H	-0.919339	-3.790123	3.662744
C	-2.246067	-2.567409	2.413274
H	-3.260762	-2.803813	2.687565
N	-2.031124	1.534233	-1.810334
N	-0.685342	1.494795	-1.636075
C	-0.187685	2.357497	-2.504857
H	0.879091	2.510992	-2.559816
C	-1.206533	2.967586	-3.257191
H	-1.103597	3.705863	-4.034363
C	-2.368889	2.419816	-2.784604
H	-3.393468	2.593390	-3.069781
N	2.149124	0.067252	-0.165592
C	2.801265	0.879129	0.657356
C	4.178317	0.969799	0.706563
H	4.666726	1.652425	1.385186
C	4.916380	0.150475	-0.159884
C	4.226100	-0.713689	-1.031013
C	2.852850	-0.708733	-0.990477
N	1.942890	1.650806	1.465863
N	0.601123	1.531425	1.296318
C	0.055100	2.365454	2.163175
H	-1.018720	2.456205	2.219906
C	1.038316	3.038509	2.910192
H	0.893900	3.773154	3.684224
C	2.229530	2.558618	2.436414
H	3.242292	2.794476	2.718283
N	2.042155	-1.526591	-1.802915
N	0.695376	-1.487855	-1.635594
C	0.202376	-2.347153	-2.510322
H	-0.864088	-2.500455	-2.571602
C	1.225413	-2.954595	-3.259207
H	1.126670	-3.690180	-4.039469
C	2.385146	-2.406802	-2.780163
H	3.411138	-2.578110	-3.061626
H	4.773535	-1.359882	-1.703617
H	-4.677115	-1.653665	1.352224
S	6.651164	0.112971	-0.250078
S	-6.649100	-0.117598	-0.297392
C	7.183031	1.311243	0.994242
H	6.863859	1.015298	1.993694
H	6.851010	2.318904	0.744024
H	8.271951	1.286975	0.956654
C	-7.184433	-1.316185	0.945018
H	-6.854572	-2.324676	0.695806
H	-8.273277	-1.287291	0.909212
H	-6.864461	-1.021396	1.944007

$[Fe(bpp^{SMe})_2]^{2+}$, pathway A; ϕ = 174.5° (minimized), θ' = 88.6° (minimized)

Fe	-0.038629	-0.133958	0.215017
N	-2.174509	-0.027589	-0.028833
C	-2.719877	0.827039	-0.890157
C	-4.081152	1.011187	-1.020408
C	-4.924492	0.246909	-0.200291
C	-4.350121	-0.667440	0.703916
H	-4.980291	-1.274793	1.339173
C	-2.980705	-0.762086	0.742823
N	-1.767128	1.519701	-1.660505
N	-0.452154	1.252348	-1.461614
C	0.199577	1.999051	-2.333328
H	1.277539	1.964530	-2.371799
C	-0.687409	2.765041	-3.113223
H	-0.446425	3.460684	-3.899182
C	-1.933299	2.430600	-2.658133
H	-2.907957	2.767022	-2.970564
N	-2.281341	-1.644917	1.586644
N	-0.926148	-1.672900	1.518591
C	-0.545776	-2.611366	2.366433
H	0.503660	-2.827285	2.495195
C	-1.653661	-3.205591	2.999266
H	-1.651976	-3.993257	3.733622
C	-2.743846	-2.566916	2.474168
H	-3.796055	-2.703391	2.662403
N	2.126385	-0.043231	0.401511
C	2.908591	-0.817990	-0.342783
C	4.288909	-0.767360	-0.305371
H	4.884790	-1.416521	-0.928477
C	4.886462	0.150695	0.568880
C	4.059817	0.971313	1.361136
C	2.701120	0.827799	1.232672
N	2.190206	-1.699041	-1.170068
N	0.835701	-1.671250	-1.126802
C	0.430950	-2.597519	-1.974142
H	-0.624047	-2.772077	-2.119411
C	1.524696	-3.243237	-2.583837
H	1.502121	-4.036473	-3.311843
C	2.630225	-2.645465	-2.046122
H	3.678683	-2.824295	-2.217208
N	1.760746	1.581134	1.964516
N	0.444235	1.353794	1.743439
C	-0.204414	2.173012	2.554078
H	-1.283529	2.179075	2.564134
C	0.690147	2.944688	3.314016
H	0.457416	3.691210	4.054362
C	1.935389	2.538984	2.911297
H	2.914760	2.857044	3.229222
H	4.494350	1.687540	2.045160
H	-4.481211	1.717410	-1.731643
S	-6.658643	0.333765	-0.212930
S	6.599767	0.363229	0.761596
C	-7.027197	1.570359	-1.479165
H	-6.675247	1.251346	-2.460297
H	-6.635381	2.550043	-1.205604

H	-8.115327	1.627288	-1.503790
C	7.319502	-0.829139	-0.391145
H	8.396973	-0.703826	-0.285591
H	7.043748	-0.602548	-1.421137
H	7.061485	-1.852377	-0.117870

$[Fe(bpp^{SMe})_2]^{2+}$, pathway A; ϕ = 165° (fixed), θ' = 83.6° (minimized)

Fe	-0.002530	-0.000423	0.200510
N	-0.377315	-2.113188	-0.086116
C	0.298286	-3.019304	0.613755
C	0.174011	-4.380677	0.418124
C	-0.721281	-4.819754	-0.567721
C	-1.449570	-3.861739	-1.299803
C	-1.238693	-2.535996	-1.013441
N	1.144086	-2.450499	1.582968
N	1.138152	-1.102781	1.738727
C	1.969996	-0.852835	2.732451
H	2.139855	0.164291	3.050654
C	2.526980	-2.042054	3.240897
H	3.231443	-2.149664	4.048318
C	1.976220	-3.040007	2.485211
H	2.110542	-4.108135	2.527383
N	-1.910484	-1.478610	-1.655913
N	-1.638544	-0.210750	-1.263650
C	-2.415304	0.565057	-1.998969
H	-2.379717	1.636398	-1.873591
C	-3.208787	-0.195230	-2.876256
H	-3.934666	0.159322	-3.588281
C	-2.861054	-1.495958	-2.627199
H	-3.218849	-2.414801	-3.061887
N	0.380139	2.113451	-0.078125
C	-0.298547	3.019338	0.618919
C	-0.171443	4.380839	0.425888
C	0.730493	4.820305	-0.553700
C	1.463944	3.862439	-1.280874
C	1.249735	2.536604	-0.997585
N	-1.153193	2.450189	1.579999
N	-1.155116	1.101830	1.729364
C	-1.997515	0.851383	2.714099
H	-2.174928	-0.166359	3.026140
C	-2.555905	2.040637	3.220864
H	-3.269193	2.147698	4.020563
C	-1.989195	3.039734	2.478735
H	-2.117132	4.108419	2.526059
N	1.923345	1.479291	-1.638226
N	1.639559	0.210969	-1.257061
C	2.413607	-0.565604	-1.994818
H	2.368171	-1.637579	-1.878565
C	3.216266	0.195013	-2.863425
H	3.941176	-0.159828	-3.576301
C	2.883948	1.496873	-2.599571
H	3.254776	2.416405	-3.021716
H	0.740281	-5.080868	1.013040
H	-2.156215	-4.174534	-2.056453

H	-0.742551	5.080689	1.016542
H	2.174895	4.175354	-2.033420
S	1.032323	6.484042	-0.948848
S	-1.024514	-6.483685	-0.961225
C	0.046880	-7.403258	0.167858
H	1.099055	-7.187039	-0.017051
H	-0.138131	-8.451567	-0.064552
H	-0.226539	-7.225106	1.207803
C	-0.037221	7.404239	0.181496
H	0.234616	7.222837	1.221308
H	-1.089862	7.191684	-0.004973
H	0.151214	8.452503	-0.048361

[Fe(bpp^{SMe})$_2$]$^{2+}$, pathway A; ϕ = 160° (fixed), θ' = 80.2° (minimized)

Fe	-0.309063	-0.001986	-0.021043
N	0.057105	2.119257	0.241479
C	-0.637773	3.010855	-0.457569
C	-0.411029	4.371389	-0.396509
C	0.602232	4.825617	0.459733
C	1.325482	3.884047	1.217945
C	1.005768	2.557601	1.070682
N	-1.636846	2.427200	-1.258188
N	-1.839184	1.088637	-1.169568
C	-2.852437	0.824338	-1.973663
H	-3.208139	-0.189269	-2.077773
C	-3.326151	1.995480	-2.595552
H	-4.138251	2.089335	-3.296587
C	-2.529028	2.997225	-2.113889
H	-2.534771	4.056079	-2.312965
N	1.631358	1.515664	1.781219
N	1.185997	0.250887	1.589478
C	1.909336	-0.508093	2.393213
H	1.741734	-1.573952	2.421994
C	2.836477	0.259926	3.119816
H	3.553533	-0.081218	3.847185
C	2.629946	1.547797	2.702705
H	3.111390	2.466943	2.993754
N	0.077440	-2.122009	-0.259491
C	-0.595068	-3.011135	0.463598
C	-0.339698	-4.367445	0.432491
C	0.678544	-4.819131	-0.419473
C	1.375377	-3.880572	-1.205897
C	1.027761	-2.558086	-1.087627
N	-1.598578	-2.428035	1.259069
N	-1.822930	-1.094762	1.144721
C	-2.837206	-0.831254	1.948027
H	-3.209559	0.178181	2.032682
C	-3.286443	-1.996432	2.598354
H	-4.092320	-2.089105	3.306690
C	-2.476083	-2.994787	2.131717
H	-2.463339	-4.049346	2.352178
N	1.626086	-1.518959	-1.825144
N	1.160399	-0.258297	-1.653339
C	1.859302	0.496537	-2.482359

H	1.671692	1.558246	-2.531396
C	2.792498	-0.269373	-3.203599
H	3.495071	0.069632	-3.945960
C	2.612653	-1.552146	-2.759516
H	3.106183	-2.468480	-3.039011
H	-0.913404	-5.054657	1.035226
H	2.151703	-4.206466	-1.884669
H	2.098292	4.210600	1.900420
H	-1.004611	5.059800	-0.978277
S	1.154315	-6.480142	-0.595854
S	1.036487	6.492644	0.680265
C	-0.095246	7.390416	-0.406419
H	0.060748	7.125163	-1.452039
H	0.165630	8.440325	-0.273355
H	-1.132546	7.250909	-0.102182
C	0.039189	-7.377749	0.508098
H	0.323366	-8.424421	0.399559
H	-1.000246	-7.267908	0.198915
H	0.186854	-7.085514	1.547702

$[Fe(bpp^{SMe})_2]^{2+}$, pathway A; ϕ = 155° (fixed), θ' = 76.3° (minimized)

Fe	-0.005261	-0.015232	-0.466001
N	2.067124	0.446946	-0.015763
C	3.042937	-0.152980	-0.688184
C	4.380048	-0.022156	-0.369960
C	4.713433	0.798490	0.717915
C	3.681225	1.450786	1.419978
C	2.389160	1.239835	1.006990
N	2.572369	-0.932168	-1.761660
N	1.237125	-0.961025	-2.003795
C	1.085765	-1.705491	-3.084431
H	0.098255	-1.881252	-3.482935
C	2.326225	-2.172489	-3.558593
H	2.513858	-2.795940	-4.416410
C	3.251149	-1.656213	-2.693239
H	4.324740	-1.745690	-2.678468
N	1.264887	1.838146	1.605807
N	0.044946	1.579605	1.077888
C	-0.811413	2.281779	1.798479
H	-1.865912	2.241966	1.571287
C	-0.152392	3.008616	2.806325
H	-0.585734	3.668010	3.539159
C	1.173089	2.701498	2.651629
H	2.040261	3.032980	3.198774
N	-2.069909	-0.465461	0.022891
C	-3.053777	0.179076	-0.593442
C	-4.380949	0.071596	-0.228335
C	-4.693946	-0.774436	0.846264
C	-3.653568	-1.475254	1.487196
C	-2.373019	-1.283996	1.030728
N	-2.601890	0.980916	-1.658339
N	-1.273793	0.994011	-1.939605
C	-1.141739	1.767502	-3.002174
H	-0.163382	1.938808	-3.424485

C	-2.387421	2.267608	-3.426233
H	-2.589409	2.918091	-4.260406
C	-3.295350	1.741938	-2.548565
H	-4.366501	1.847345	-2.499504
N	-1.241947	-1.928762	1.565421
N	-0.035251	-1.675451	1.004261
C	0.829363	-2.425089	1.664458
H	1.875796	-2.398439	1.401341
C	0.189125	-3.180660	2.663207
H	0.632290	-3.880315	3.351479
C	-1.133483	-2.838669	2.569363
H	-1.988521	-3.175653	3.132052
H	-5.149028	0.615111	-0.757166
H	-3.871134	-2.145417	2.307698
H	5.140738	-0.527734	-0.945148
H	3.913578	2.101679	2.251829
S	-6.295870	-1.040427	1.462951
S	6.331623	1.093116	1.276219
C	7.367942	0.125463	0.154616
H	8.387987	0.300950	0.496589
H	7.281374	0.481559	-0.872137
H	7.154786	-0.940789	0.233414
C	-7.350321	-0.020989	0.405568
H	-7.106365	1.037270	0.500765
H	-8.360236	-0.178672	0.784347
H	-7.313677	-0.353847	-0.631912

$[Fe(bpp^{SMe})_2]^{2+}$, pathway A; ϕ = 150° (fixed), θ' = 73.6° (minimized)

Fe	-0.577571	-0.002155	-0.022295
N	-0.007600	-2.069248	-0.372448
C	-0.625085	-3.042127	0.287396
C	-0.226383	-4.363603	0.244908
C	0.883013	-4.683845	-0.552514
C	1.524036	-3.656301	-1.271014
C	1.033082	-2.379900	-1.145048
N	-1.730251	-2.586938	1.031690
N	-2.049706	-1.268692	0.978898
C	-3.139367	-1.133918	1.713953
H	-3.592714	-0.161459	1.831119
C	-3.544470	-2.368984	2.252972
H	-4.393128	-2.567695	2.885439
C	-2.625661	-3.272467	1.793162
H	-2.550703	-4.336350	1.945940
N	1.559684	-1.263841	-1.820257
N	0.948955	-0.066680	-1.650313
C	1.610441	0.781423	-2.417056
H	1.311605	1.817890	-2.455940
C	2.661167	0.140354	-3.098181
H	3.362146	0.572041	-3.792386
C	2.600475	-1.164859	-2.689127
H	3.206908	-2.014713	-2.956135
N	-0.023056	2.065162	0.353950
C	-0.649901	3.042068	-0.291736
C	-0.275589	4.369017	-0.215801

C	0.819099	4.691422	0.600623
C	1.468220	3.660088	1.306062
C	1.001353	2.378389	1.147477
N	-1.738414	2.585422	-1.059494
N	-2.049096	1.264317	-1.023931
C	-3.127067	1.129030	-1.775764
H	-3.572439	0.154771	-1.907850
C	-3.532507	2.366104	-2.309916
H	-4.372957	2.564604	-2.953341
C	-2.624684	3.271064	-1.831639
H	-2.552566	4.336185	-1.976863
N	1.540341	1.258691	1.806450
N	0.950425	0.055875	1.609702
C	1.624426	-0.797301	2.360021
H	1.344305	-1.839648	2.374809
C	2.661427	-0.152947	3.058838
H	3.368049	-0.587316	3.745602
C	2.577651	1.159947	2.679626
H	3.168126	2.014273	2.967227
H	-0.814674	5.131762	-0.756906
H	2.300695	3.884883	1.958927
H	-0.757953	-5.124044	0.796647
H	2.368174	-3.880375	-1.909006
S	1.537133	-6.281240	-0.746850
S	1.447268	6.294474	0.830936
C	0.373400	7.333112	-0.187456
H	-0.657976	7.302182	0.163953
H	0.449065	7.070211	-1.242572
H	0.755858	8.344219	-0.053105
C	0.479415	-7.314604	0.293649
H	0.545664	-7.025087	1.342464
H	0.880732	-8.321485	0.184824
H	-0.550817	-7.311160	-0.062316

$[Fe(bpp^{SMe})_2]^{2+}$, pathway A; ϕ = 145° (fixed), θ' = 71.9° (minimized)

Fe	-0.009667	0.690009	-0.015731
N	2.052537	0.055097	0.285235
C	3.023234	0.668155	-0.382418
C	4.340557	0.255554	-0.365464
C	4.661644	-0.864446	0.416552
C	3.639347	-1.497714	1.148478
C	2.366558	-0.991553	1.048509
N	2.571503	1.785883	-1.111682
N	1.262323	2.134266	-1.024593
C	1.132797	3.227055	-1.756787
H	0.167883	3.701757	-1.849540
C	2.361534	3.603818	-2.329047
H	2.562065	4.447201	-2.967959
C	3.256179	2.665668	-1.891736
H	4.313528	2.566530	-2.073525
N	1.260384	-1.507636	1.745272
N	0.071103	-0.874487	1.613261
C	-0.766436	-1.527985	2.397977
H	-1.796190	-1.212222	2.469973

C	-0.126391	-2.598174	3.050063
H	-0.551793	-3.298793	3.748500
C	1.167352	-2.557397	2.604602
H	2.012938	-3.182642	2.839700
N	-2.058534	0.015757	-0.299881
C	-3.031371	0.580054	0.405780
C	-4.334037	0.122781	0.411028
C	-4.636096	-0.989499	-0.389564
C	-3.611589	-1.571434	-1.160480
C	-2.354834	-1.023276	-1.079761
N	-2.597218	1.694041	1.151218
N	-1.301923	2.086312	1.040387
C	-1.190341	3.171713	1.785816
H	-0.239607	3.676976	1.862421
C	-2.416549	3.498658	2.393054
H	-2.629193	4.325792	3.049084
C	-3.288653	2.533536	1.968555
H	-4.336415	2.394056	2.177512
N	-1.247091	-1.482554	-1.813424
N	-0.076767	-0.813116	-1.686999
C	0.765291	-1.414250	-2.507954
H	1.782165	-1.061503	-2.590448
C	0.146137	-2.482976	-3.182070
H	0.578683	-3.146088	-3.912040
C	-1.137440	-2.501710	-2.706638
H	-1.966817	-3.147628	-2.943772
H	-5.094750	0.612147	1.000090
H	-3.823545	-2.414420	-1.804025
H	5.097836	0.784898	-0.923559
H	3.864534	-2.347261	1.778735
S	-6.209081	-1.714490	-0.522032
S	6.254727	-1.538280	0.573743
C	-7.254134	-0.688263	0.538308
H	-7.296178	0.340028	0.178251
H	-6.937313	-0.739400	1.580305
H	-8.249596	-1.124900	0.456985
C	7.285660	-0.472650	-0.461141
H	7.293269	0.552624	-0.090307
H	6.985960	-0.521951	-1.508306
H	8.291824	-0.881666	-0.368517

[Fe(bpp^{SMe})$_2$]$^{2+}$, pathway A; ϕ = 140° (fixed), θ' = 68.6° (minimized)

Fe	-0.214485	-0.076207	-0.799217
N	-0.225943	-2.029924	0.170629
C	-1.252726	-2.843165	-0.046667
C	-1.469367	-4.007644	0.662645
C	-0.535800	-4.348219	1.653596
C	0.568933	-3.501984	1.865167
C	0.671987	-2.370165	1.093401
N	-2.105425	-2.395521	-1.076902
N	-1.813753	-1.228212	-1.705171
C	-2.734470	-1.082503	-2.642551
H	-2.720497	-0.212607	-3.281431
C	-3.635823	-2.161921	-2.638271

H	-4.481488	-2.318793	-3.286343
C	-3.200605	-2.980979	-1.631883
H	-3.581441	-3.926954	-1.283993
N	1.736728	-1.457234	1.177585
N	1.736575	-0.397932	0.335178
C	2.853722	0.257803	0.592912
H	3.091422	1.157060	0.044991
C	3.596570	-0.375237	1.606693
H	4.545618	-0.075937	2.018260
C	2.851846	-1.469246	1.955769
H	3.035018	-2.242573	2.683538
N	0.178293	2.018178	-0.333448
C	0.937069	2.724547	-1.163226
C	1.420964	3.984085	-0.871348
C	1.070206	4.544682	0.366511
C	0.240568	3.809633	1.233852
C	-0.175901	2.564909	0.827971
N	1.203335	2.054419	-2.375322
N	0.690186	0.811172	-2.559501
C	1.046105	0.450789	-3.780519
H	0.757911	-0.516763	-4.162427
C	1.793352	1.462726	-4.409066
H	2.212256	1.454977	-5.401140
C	1.869272	2.470383	-3.485880
H	2.333566	3.440882	-3.544579
N	-1.027307	1.737761	1.579874
N	-1.383210	0.542847	1.053371
C	-2.207237	0.001327	1.932174
H	-2.635963	-0.971156	1.742947
C	-2.400842	0.846427	3.040605
H	-3.020531	0.675337	3.904525
C	-1.632429	1.948582	2.779145
H	-1.480805	2.851700	3.347161
H	2.040065	4.520080	-1.574694
H	-0.062233	4.227606	2.184262
H	-2.321680	-4.636524	0.455007
H	1.314507	-3.752638	2.607371
S	-0.625297	-5.765467	2.654152
S	1.579264	6.109452	0.923871
C	2.571066	6.760683	-0.440203
H	3.457014	6.150655	-0.617376
H	2.891407	7.747199	-0.105521
H	1.973554	6.878857	-1.344297
C	-2.114749	-6.615046	2.081327
H	-2.023831	-6.914573	1.037100
H	-3.008860	-6.015877	2.254493
H	-2.175680	-7.513755	2.694894

$[Fe(bpp^{SPh})_2]^{2+}$, undistorted – ϕ = 178.4° (minimized), θ' = 88.2° (constrained)

Fe	-0.044219	0.003372	0.631350
N	0.124095	2.143865	0.668836
C	0.975212	2.771827	-0.136096
C	1.140464	4.139546	-0.152486
C	0.355795	4.897656	0.726071

C	-0.554666	4.240997	1.572432
H	-1.176574	4.807554	2.252157
C	-0.622838	2.869034	1.499720
N	1.703709	1.893256	-0.962981
N	1.513804	0.556454	-0.828396
C	2.317352	-0.009337	-1.711936
H	2.351223	-1.084598	-1.796981
C	3.039418	0.956885	-2.434232
H	3.764082	0.795227	-3.214157
C	2.622851	2.159122	-1.927569
H	2.908236	3.167099	-2.180649
N	-1.492630	2.085185	2.285507
N	-1.524257	0.742877	2.087957
C	-2.419667	0.276185	2.940592
H	-2.629488	-0.781810	2.974823
C	-2.981985	1.313281	3.704867
H	-3.731938	1.236988	4.473838
C	-2.367998	2.452847	3.257621
H	-2.488720	3.480293	3.559291
N	-0.165174	-2.140378	0.628535
C	-0.993025	-2.771674	-0.197882
C	-1.122809	-4.142459	-0.246230
H	-1.812625	-4.630019	-0.920284
C	-0.327425	-4.899574	0.623449
C	0.554289	-4.238982	1.496623
C	0.587879	-2.864412	1.454916
N	-1.737841	-1.893624	-1.010671
N	-1.586582	-0.555552	-0.842276
C	-2.403099	0.008842	-1.714890
H	-2.467514	1.084448	-1.773568
C	-3.094192	-0.959342	-2.464358
H	-3.820258	-0.798981	-3.243254
C	-2.645793	-2.161419	-1.985281
H	-2.902034	-3.170558	-2.263690
N	1.425092	-2.076249	2.271085
N	1.421222	-0.729297	2.106426
C	2.289984	-0.258474	2.983809
H	2.469205	0.803874	3.046109
C	2.869849	-1.297583	3.732114
H	3.605777	-1.219059	4.514298
C	2.293471	-2.442551	3.249985
H	2.437377	-3.473019	3.530363
H	1.181072	-4.804438	2.172767
H	1.849189	4.623133	-0.809449
S	-0.361119	-6.645879	0.696901
S	0.434676	6.640150	0.842632
C	-1.599661	-7.061543	-0.519420
C	-3.515527	-7.770417	-2.387877
C	-1.215280	-7.317028	-1.834086
C	-2.932849	-7.172926	-0.128730
C	-3.890472	-7.524423	-1.071460
C	-2.181613	-7.669358	-2.768055
H	-0.171569	-7.254530	-2.118369
H	-3.214021	-6.998716	0.903146

H	-4.927148	-7.623485	-0.772999
H	-1.889259	-7.881894	-3.789280
H	-4.263465	-8.059367	-3.116567
C	1.694257	7.054522	-0.352447
C	3.640653	7.759600	-2.189706
C	3.027175	7.119313	0.049623
C	1.325688	7.355031	-1.662217
C	2.307237	7.705183	-2.580868
C	4.000029	7.469350	-0.877856
H	3.296241	6.910837	1.078345
H	0.282579	7.327920	-1.954144
H	2.027832	7.951959	-3.598012
H	5.037006	7.532573	-0.570880
H	4.401313	8.046667	-2.905898

$[Fe(bpp^{SPh})_2]^{2+}$, pathway A; ϕ = 177.1° (minimized), θ' = 90.0° (minimized)

Fe	-0.064954	0.007451	0.583179
N	-2.205116	0.104240	0.445357
C	-2.792753	0.930530	-0.418272
C	-4.155939	0.993391	-0.603951
C	-4.956429	0.138424	0.164928
C	-4.343773	-0.732945	1.083006
H	-4.943212	-1.396805	1.690908
C	-2.972472	-0.703703	1.180114
N	-1.881036	1.742916	-1.118022
N	-0.557897	1.627862	-0.843130
C	0.043552	2.521248	-1.606706
H	1.116957	2.625841	-1.569048
C	-0.885979	3.230683	-2.390198
H	-0.689557	4.014947	-3.101506
C	-2.104301	2.708634	-2.049927
H	-3.096526	2.952427	-2.392740
N	-2.235287	-1.512217	2.066523
N	-0.884935	-1.388341	2.092445
C	-0.467358	-2.227075	3.022509
H	0.587271	-2.313709	3.234388
C	-1.546195	-2.908254	3.615891
H	-1.512491	-3.647287	4.398386
C	-2.658783	-2.423804	2.983328
H	-3.702093	-2.655779	3.120438
N	2.090073	-0.086010	0.830461
C	2.822927	-0.916910	0.095542
C	4.188198	-1.046705	0.213847
H	4.754579	-1.732332	-0.400111
C	4.831158	-0.251810	1.172655
C	4.065597	0.628903	1.954344
C	2.706607	0.669067	1.738831
N	2.049219	-1.658064	-0.821948
N	0.709213	-1.460517	-0.844648
C	0.242386	-2.270798	-1.780314
H	-0.815578	-2.298693	-1.991316
C	1.280946	-3.004591	-2.377561
H	1.205594	-3.736569	-3.163712
C	2.419829	-2.587004	-1.739759

H	3.449113	-2.878454	-1.871343
N	1.830174	1.507555	2.450087
N	0.508933	1.470237	2.153101
C	-0.063060	2.347973	2.955075
H	-1.129938	2.503982	2.910019
C	0.886174	2.970596	3.788216
H	0.715555	3.723960	4.538485
C	2.083364	2.409969	3.437692
H	3.078518	2.584595	3.811810
H	4.540523	1.253194	2.698899
H	-4.604704	1.676115	-1.310925
S	-6.699064	0.084929	0.060714
S	6.553005	-0.284013	1.469809
C	7.135128	-1.481717	0.280941
C	8.103466	-3.325573	-1.541425
C	7.226432	-2.823703	0.644834
C	7.538199	-1.052513	-0.982021
C	8.020313	-1.982870	-1.893819
C	7.709657	-3.745248	-0.275423
H	6.936236	-3.139194	1.640043
H	7.794826	-4.788615	0.003276
C	-7.053480	1.324562	-1.174053
C	-7.658010	3.235843	-3.082658
C	-7.295551	2.638227	-0.777557
C	-7.127847	0.955738	-2.515753
C	-7.427457	1.920138	-3.469670
C	-7.594838	3.593915	-1.740350
H	-7.262629	2.905701	0.271956
H	-6.965614	-0.075285	-2.806949
H	-7.795289	4.615062	-1.439346
H	8.493425	-4.045805	-2.250820
H	8.347119	-1.655659	-2.873451
H	-7.904727	3.981964	-3.828507
H	-7.497897	1.639438	-4.513572
H	7.488503	-0.001688	-1.241523

$[Fe(bpp^{SPh})_2]^{2+}$, pathway A; ϕ = 165° (fixed), θ' = 84.2° (minimized)

Fe	-0.003440	-0.001282	-0.118972
N	-0.392847	-2.108499	-0.404922
C	0.281355	-3.018848	0.293651
C	0.153532	-4.377012	0.098009
C	-0.745688	-4.810237	-0.884660
C	-1.475805	-3.854353	-1.613713
C	-1.258530	-2.527767	-1.328172
N	1.134590	-2.454818	1.258972
N	1.142121	-1.107369	1.413436
C	1.984131	-0.866807	2.401560
H	2.166077	0.148469	2.718987
C	2.534259	-2.061352	2.904334
H	3.244495	-2.175516	3.705690
C	1.966519	-3.054276	2.153884
H	2.087211	-4.124474	2.192369
N	-1.926168	-1.467664	-1.971648
N	-1.646974	-0.201011	-1.581791

C	-2.419629	0.578169	-2.317803
H	-2.378834	1.649354	-2.192619
C	-3.215268	-0.179203	-3.195950
H	-3.938142	0.178301	-3.909520
C	-2.874022	-1.481572	-2.945340
H	-3.235070	-2.399499	-3.379152
N	0.391282	2.106532	-0.397440
C	-0.285671	3.016499	0.298777
C	-0.153205	4.375025	0.108493
C	0.751763	4.809463	-0.868357
C	1.484159	3.853715	-1.595505
C	1.263216	2.526856	-1.314381
N	-1.148222	2.452072	1.255503
N	-1.161424	1.104298	1.405056
C	-2.012255	0.862832	2.385356
H	-2.199468	-0.152927	2.698106
C	-2.564474	2.057225	2.886305
H	-3.282160	2.170835	3.681072
C	-1.986205	3.051025	2.145193
H	-2.104506	4.121400	2.186060
N	1.932470	1.467218	-1.956997
N	1.647789	0.200183	-1.572787
C	2.422535	-0.578871	-2.306803
H	2.378234	-1.650309	-2.185102
C	3.224530	0.179135	-3.178567
H	3.950091	-0.178003	-3.889585
C	2.885931	1.481685	-2.925112
H	3.251815	2.400004	-3.354021
H	0.714598	-5.092731	0.681574
H	-2.185553	-4.165439	-2.368211
H	-0.716862	5.088465	0.692101
H	2.198431	4.165384	-2.345484
S	1.047330	6.487744	-1.249712
S	-1.037941	-6.488953	-1.266283
C	0.048455	-7.343269	-0.136594
C	1.711642	-8.727923	1.589589
C	-0.425344	-7.720925	1.118113
C	1.342691	-7.665696	-0.540279
C	2.174053	-8.357192	0.331597
C	0.415064	-8.412405	1.981408
H	-1.443163	-7.489172	1.408480
H	1.688957	-7.391186	-1.529708
H	3.177600	-8.621501	0.020806
H	0.050386	-8.720324	2.953878
H	2.358796	-9.279737	2.260806
C	-0.039386	7.345654	-0.123445
C	-1.701234	8.733438	1.601573
C	-1.330363	7.675376	-0.530551
C	0.432009	7.717691	1.134122
C	-0.407857	8.410155	1.997061
C	-2.160844	8.369206	0.340548
H	-1.674118	7.406375	-1.522288
H	1.447668	7.479871	1.427424
H	-0.045300	8.712199	2.972165

H	-3.161673	8.640393	0.026920
H	-2.348139	9.285879	2.272501

$[Fe(bpp^{SPh})_2]^{2+}$, pathway A; ϕ = 160° (fixed), θ' = 80.3° (minimized)

Fe	0.001023	0.000593	-0.026311
N	-2.118920	0.365961	0.243482
C	-3.013573	-0.331849	-0.451882
C	-4.371528	-0.110227	-0.386607
C	-4.823102	0.900189	0.472597
C	-3.886181	1.622862	1.232560
C	-2.557551	1.306954	1.079188
N	-2.430382	-1.326314	-1.259157
N	-1.092420	-1.529534	-1.172866
C	-0.831154	-2.540192	-1.982209
H	0.181815	-2.896722	-2.089510
C	-2.003837	-3.008993	-2.603919
H	-2.099921	-3.817807	-3.308362
C	-3.004317	-2.211643	-2.117718
H	-4.064451	-2.211610	-2.311917
N	-1.515099	1.936179	1.786973
N	-0.249418	1.499686	1.585499
C	0.511296	2.226976	2.383901
H	1.578570	2.067179	2.402710
C	-0.257790	3.147569	3.118279
H	0.083938	3.866748	3.843235
C	-1.547948	2.929424	2.714172
H	-2.468682	3.402150	3.014207
N	2.119276	0.387618	-0.271153
C	3.012766	-0.287090	0.447537
C	4.367405	-0.041175	0.405451
C	4.814950	0.978110	-0.445298
C	3.879158	1.679729	-1.226195
C	2.554635	1.334377	-1.101694
N	2.431246	-1.283594	1.253330
N	1.099379	-1.512085	1.140558
C	0.840674	-2.525490	1.947229
H	-0.166647	-2.903557	2.031558
C	2.007533	-2.964658	2.600847
H	2.103716	-3.766563	3.313143
C	3.001980	-2.147476	2.135306
H	4.056321	-2.121168	2.357417
N	1.514032	1.935550	-1.836012
N	0.253756	1.471996	-1.659698
C	-0.502678	2.168827	-2.488819
H	-1.564783	1.982463	-2.533615
C	0.262717	3.098992	-3.214948
H	-0.077382	3.800096	-3.958141
C	1.546472	2.918980	-2.773521
H	2.462829	3.410909	-3.055544
H	5.071327	-0.612528	0.993620
H	4.203653	2.455417	-1.906474
H	-4.212358	2.393497	1.917772
H	-5.075359	-0.694013	-0.962754
S	6.491394	1.442554	-0.608190

S	-6.506283	1.321216	0.679244
C	7.322955	0.293599	0.475650
C	8.675292	-1.466754	2.128647
C	7.552184	0.640247	1.805168
C	7.779102	-0.919565	-0.037797
C	8.452301	-1.801278	0.797588
C	8.229037	-0.248550	2.630812
H	7.220644	1.600097	2.182793
H	7.618265	-1.164228	-1.081170
H	8.817277	-2.742015	0.403583
H	8.424499	0.018613	3.662256
H	9.213967	-2.151131	2.773121
C	-7.332480	0.172225	-0.408321
C	-8.670535	-1.586397	-2.074867
C	-7.764327	-1.054352	0.092506
C	-7.578263	0.532690	-1.732125
C	-8.245921	-0.355853	-2.565034
C	-8.432586	-1.934126	-0.749452
H	-7.592344	-1.309550	1.131351
H	-7.262101	1.501465	-2.100567
H	-8.450141	-0.079338	-3.592333
H	-8.783091	-2.884131	-0.364680
H	-9.203830	-2.270178	-2.724432

$[Fe(bpp^{SPh})_2]^{2+}$, pathway A; ϕ = 155° (fixed), θ' = 76.6° (minimized)

Fe	-0.003973	-0.031896	-0.241820
N	2.062301	0.446986	0.205062
C	3.042810	-0.153207	-0.464046
C	4.376898	-0.016601	-0.147319
C	4.704925	0.813923	0.932459
C	3.674477	1.465549	1.633348
C	2.381287	1.243965	1.223784
N	2.577284	-0.939209	-1.535218
N	1.243742	-0.977394	-1.779938
C	1.099502	-1.725524	-2.859473
H	0.113968	-1.909668	-3.258964
C	2.344498	-2.182731	-3.330445
H	2.538534	-2.805628	-4.187166
C	3.264477	-1.657079	-2.464090
H	4.339225	-1.734879	-2.443993
N	1.254212	1.834871	1.826200
N	0.035134	1.564365	1.304091
C	-0.825675	2.254249	2.031024
H	-1.880787	2.201942	1.809089
C	-0.169294	2.987166	3.036271
H	-0.605800	3.640197	3.772877
C	1.158981	2.695815	2.873336
H	2.025725	3.035379	3.416142
N	-2.065209	-0.487011	0.249216
C	-3.052222	0.160894	-0.362442
C	-4.376262	0.053429	0.004208
C	-4.685097	-0.798620	1.073088
C	-3.647789	-1.501749	1.711703
C	-2.366070	-1.306853	1.254758

N	-2.604334	0.966485	-1.426483
N	-1.278992	0.982721	-1.714079
C	-1.152881	1.756117	-2.777893
H	-0.176833	1.929118	-3.204672
C	-2.401741	2.253758	-3.194434
H	-2.609380	2.904398	-4.027017
C	-3.304756	1.724599	-2.312313
H	-4.376233	1.825167	-2.254014
N	-1.233323	-1.950648	1.788797
N	-0.028094	-1.696383	1.226275
C	0.839056	-2.442317	1.887223
H	1.884944	-2.413967	1.622033
C	0.201433	-3.195718	2.889223
H	0.646462	-3.892148	3.579513
C	-1.122459	-2.858139	2.794199
H	-1.976661	-3.195032	3.358164
H	-5.157846	0.593944	-0.510368
H	-3.864855	-2.176479	2.528696
H	5.152336	-0.518351	-0.708310
H	3.904778	2.120907	2.462372
S	-6.303751	-1.060809	1.674990
S	6.338938	1.112899	1.472352
C	-7.293523	-0.033372	0.601949
C	-8.881426	1.540057	-1.030431
C	-7.586036	1.274735	0.982550
C	-7.801302	-0.565675	-0.581600
C	-8.593572	0.230228	-1.398982
C	-8.381480	2.060554	0.158257
H	-7.208663	1.666743	1.919601
H	-7.589529	-1.593479	-0.851637
H	-9.000552	-0.179403	-2.315573
H	-8.624083	3.074342	0.453017
H	-9.512000	2.152214	-1.664166
C	7.311969	0.109513	0.361919
C	8.877899	-1.429176	-1.323748
C	7.791334	0.662025	-0.824245
C	7.622139	-1.201433	0.718397
C	8.405345	-1.970093	-0.132921
C	8.573588	-0.116503	-1.667919
H	7.566148	1.691635	-1.075851
H	7.267548	-1.608764	1.657793
H	8.660959	-2.986112	0.142506
H	8.959471	0.308800	-2.586486
H	9.500155	-2.027636	-1.978429

$[Fe(bpp^{SPh})_2]^{2+}$, $\phi = 150°$ (fixed), $\theta' = 74.2°$ (minimized)

Fe	-0.040216	0.006003	-0.377494
N	-0.397560	2.068154	0.192089
C	0.266479	3.042598	-0.422398
C	0.224919	4.361631	-0.025836
C	-0.581924	4.681962	1.074492
C	-1.308727	3.660027	1.711243
C	-1.178453	2.380994	1.223928
N	1.016744	2.588160	-1.524197

N	0.967488	1.271017	-1.845182
C	1.707921	1.140548	-2.932404
H	1.830104	0.169156	-3.386631
C	2.244958	2.377793	-3.332772
H	2.880325	2.579456	-4.178479
C	1.777415	3.279111	-2.414797
H	1.922048	4.344130	-2.334825
N	-1.856369	1.265656	1.749502
N	-1.676607	0.066429	1.147500
C	-2.434297	-0.784358	1.816053
H	-2.461494	-1.823696	1.526023
C	-3.123592	-0.141004	2.860003
H	-3.814122	-0.573462	3.564068
C	-2.731676	1.168877	2.784730
H	-3.010643	2.022196	3.380757
N	0.349280	-2.054815	0.175195
C	-0.295292	-3.036018	-0.449960
C	-0.209486	-4.361360	-0.083216
C	0.616737	-4.680883	1.002420
C	1.318930	-3.651388	1.654115
C	1.148622	-2.367270	1.193558
N	-1.075572	-2.582010	-1.531187
N	-1.047967	-1.261705	-1.842931
C	-1.807097	-1.132618	-2.917063
H	-1.945990	-0.160101	-3.364127
C	-2.339458	-2.372763	-3.314983
H	-2.988446	-2.575445	-4.150079
C	-1.850704	-3.274262	-2.408313
H	-1.988535	-4.340188	-2.328591
N	1.801461	-1.245101	1.736351
N	1.596591	-0.042383	1.150354
C	2.338992	0.814368	1.828251
H	2.345840	1.857724	1.551399
C	3.041282	0.171671	2.863951
H	3.723654	0.608713	3.573114
C	2.670822	-1.143617	2.776104
H	2.962029	-1.997697	3.365073
H	-0.744283	-5.137269	-0.611985
H	1.977779	-3.875755	2.482010
H	0.779560	5.131813	-0.542584
H	-1.952732	3.886291	2.550252
S	-0.766264	6.297089	1.712708
S	0.851782	-6.302987	1.604941
C	0.281380	7.266039	0.640250
C	1.889722	8.811288	-0.996739
C	-0.248505	7.806770	-0.529649
C	1.604638	7.504605	1.006027
C	2.407806	8.279755	0.179459
C	0.564905	8.577804	-1.349903
H	-1.287509	7.637075	-0.786007
H	1.994621	7.101959	1.933285
H	3.434039	8.480724	0.461979
H	0.158666	9.010824	-2.255918
H	2.517048	9.424303	-1.632873

C	-0.182343	-7.279291	0.525876
C	-1.763285	-8.852176	-1.113135
C	-1.507107	-7.525656	0.880565
C	0.362648	-7.826469	-0.634431
C	-0.436930	-8.611295	-1.455153
C	-2.296808	-8.313100	0.052868
H	-1.908478	-7.120164	1.801707
H	1.402410	-7.650634	-0.883562
H	-0.018134	-9.048701	-2.353338
H	-3.323867	-8.520312	0.327742
H	-2.379175	-9.476760	-1.749202

$[Fe(bpp^{SPh})_2]^{2+}$, pathway A; $\phi = 145°$ (fixed), $\theta' = 72.5°$ (minimized)

Fe	0.511195	0.009275	-0.020614
N	-0.121052	-2.048685	0.285972
C	0.490143	-3.021377	-0.384482
C	0.081967	-4.336962	-0.363888
C	-1.028200	-4.658573	0.428726
C	-1.660037	-3.641509	1.164662
C	-1.159588	-2.365170	1.057175
N	1.604356	-2.570974	-1.120687
N	1.954324	-1.262799	-1.039630
C	3.042643	-1.136099	-1.779482
H	3.516724	-0.171680	-1.879656
C	3.415538	-2.367787	-2.347405
H	4.255481	-2.571477	-2.989761
C	2.479772	-3.260994	-1.899439
H	2.377457	-4.320007	-2.071390
N	-1.676184	-1.259476	1.755513
N	-1.051452	-0.067651	1.614023
C	-1.710042	0.771957	2.392085
H	-1.402171	1.804831	2.453506
C	-2.772704	0.128974	3.053198
H	-3.474652	0.554578	3.750110
C	-2.720462	-1.169094	2.621311
H	-3.336286	-2.018482	2.867088
N	-0.163744	2.054911	-0.307546
C	0.397718	3.029100	0.401704
C	-0.057139	4.329568	0.405867
C	-1.163050	4.631190	-0.400790
C	-1.742849	3.612277	-1.176479
C	-1.196217	2.353086	-1.092978
N	1.509614	2.595665	1.151218
N	1.900908	1.300397	1.047052
C	2.980366	1.190700	1.802199
H	3.483473	0.239611	1.887359
C	3.304191	2.419360	2.405744
H	4.126486	2.633667	3.067208
C	2.347677	3.293163	1.963554
H	2.208305	4.343863	2.159164
N	-1.651150	1.247140	-1.832962
N	-0.988061	0.075469	-1.698867
C	-1.592099	-0.769780	-2.514747
H	-1.245260	-1.789352	-2.587889

C	-2.656690	-0.149777	-3.194191
H	-3.322528	-0.584020	-3.920605
C	-2.658558	1.141626	-2.739425
H	-3.292251	1.976113	-2.991010
H	0.419121	5.100386	0.994604
H	-2.580640	3.826580	-1.826086
H	0.597841	-5.104977	-0.922156
H	-2.503466	-3.869046	1.802309
S	-1.874913	6.221686	-0.522165
S	-1.680200	-6.271251	0.584577
C	-0.592596	-7.239299	-0.448760
C	1.056422	-8.799634	-2.032816
C	0.558981	-7.798579	0.101252
C	-0.933466	-7.466662	-1.780604
C	-0.100831	-8.248550	-2.571553
C	1.385016	-8.576769	-0.699932
H	0.797383	-7.638027	1.146008
H	-1.846939	-7.049336	-2.187579
H	-0.365444	-8.440262	-3.604419
H	2.276456	-9.024395	-0.277302
H	1.695914	-9.419669	-2.649685
C	-0.799890	7.211984	0.503633
C	0.832976	8.804305	2.072919
C	-1.135868	7.436546	1.836797
C	0.338533	7.789602	-0.055145
C	1.156739	8.583290	0.738591
C	-0.311458	8.235317	2.620187
H	-2.039153	7.004617	2.251191
H	0.572252	7.630779	-1.101251
H	2.037718	9.044516	0.308687
H	-0.572610	8.425720	3.654203
H	1.466211	9.436333	2.683980

$[Fe(bpp^{SPh})_2]^{2+}$, pathway A; ϕ = 140° (fixed), θ' = 68.9° (minimized)

Fe	-0.191139	-0.058665	-0.686246
N	-0.192924	-2.010888	0.278839
C	-1.221064	-2.825516	0.062755
C	-1.438364	-3.987361	0.770179
C	-0.503663	-4.327250	1.757977
C	0.600571	-3.484750	1.972990
C	0.703056	-2.350410	1.201755
N	-2.076795	-2.377990	-0.965334
N	-1.790963	-1.209338	-1.592491
C	-2.714753	-1.067710	-2.528146
H	-2.705314	-0.198035	-3.167359
C	-3.611791	-2.150397	-2.520978
H	-4.458597	-2.310285	-3.166723
C	-3.171160	-2.968088	-1.514962
H	-3.545063	-3.915557	-1.162607
N	1.764756	-1.433848	1.292290
N	1.761424	-0.369956	0.456374
C	2.873050	0.291734	0.722395
H	3.106444	1.195995	0.180725
C	3.614971	-0.342094	1.736612

H	4.559735	-0.038735	2.154917
C	2.876205	-1.443532	2.075371
H	3.060246	-2.219469	2.800079
N	0.192678	2.032580	-0.218467
C	0.952312	2.740926	-1.048448
C	1.433393	3.998627	-0.758994
C	1.078282	4.558805	0.476011
C	0.253420	3.826489	1.346768
C	-0.159853	2.578265	0.942330
N	1.222495	2.071086	-2.260082
N	0.714561	0.826803	-2.445973
C	1.073712	0.470585	-3.667823
H	0.789032	-0.496990	-4.052219
C	1.818731	1.486026	-4.292370
H	2.239705	1.481483	-5.283526
C	1.889589	2.492718	-3.366799
H	2.350000	3.465832	-3.418496
N	-1.004221	1.747566	1.699087
N	-1.357056	0.550739	1.175232
C	-2.173520	0.005593	2.058701
H	-2.598291	-0.969190	1.871913
C	-2.363167	0.849644	3.168847
H	-2.975796	0.675436	4.037128
C	-1.601579	1.955460	2.902486
H	-1.448772	2.858563	3.470132
H	2.054770	4.548073	-1.451609
H	-0.050329	4.245216	2.296572
H	-2.288554	-4.625395	0.576220
H	1.345939	-3.735859	2.715292
S	-0.616185	-5.759241	2.752781
S	1.595260	6.141011	1.008720
C	-2.079120	-6.560711	2.117195
C	-4.336236	-7.855053	1.172139
C	-1.962777	-7.460078	1.058960
C	-3.312950	-6.315633	2.716190
C	-4.442871	-6.965894	2.235968
C	-3.099589	-8.102711	0.585806
H	-0.992762	-7.663103	0.620814
H	-3.384537	-5.636204	3.557303
H	-5.403816	-6.790612	2.704406
H	-3.015523	-8.811555	-0.229096
H	-5.217761	-8.370143	0.809474
C	2.551354	6.722836	-0.382003
C	4.039923	7.669048	-2.514919
C	3.928025	6.510084	-0.406595
C	1.915545	7.420120	-1.407571
C	2.667030	7.888200	-2.477935
C	4.669791	6.984738	-1.481105
H	4.413938	5.992190	0.411864
H	0.848558	7.603413	-1.361533
H	2.181804	8.439726	-3.274133
H	5.742406	6.834225	-1.502256
H	4.624143	8.048410	-3.344799

[FeL_2]$^{2+}$, undistorted − ϕ = 177.2° (minimized), θ' = 88.0° (constrained)

Fe	-0.051082	0.003366	1.172109
N	-0.283877	-2.128498	1.219319
C	-1.157767	-2.734388	0.421066
C	-1.367036	-4.095101	0.413151
C	-0.600111	-4.874254	1.289776
C	0.336138	-4.242343	2.127041
H	0.946108	-4.825404	2.803692
C	0.444286	-2.873267	2.049252
N	-1.860422	-1.837415	-0.409520
N	-1.626101	-0.507243	-0.285119
C	-2.411264	0.078307	-1.172442
H	-2.408817	1.153442	-1.265615
C	-3.166383	-0.868896	-1.885836
H	-3.886405	-0.689610	-2.666179
C	-2.788466	-2.080815	-1.370873
H	-3.105432	-3.081409	-1.616058
N	1.341342	-2.112297	2.828333
N	1.420762	-0.774173	2.617501
C	2.333651	-0.331751	3.464651
H	2.581487	0.718256	3.487656
C	2.858319	-1.380599	4.239262
H	3.610926	-1.323934	5.007302
C	2.202009	-2.501546	3.804441
H	2.284054	-3.529614	4.116697
N	0.079898	2.144574	1.153411
C	0.907021	2.764993	0.317588
C	1.039922	4.133587	0.251800
H	1.724841	4.613379	-0.432947
C	0.253488	4.904146	1.118660
C	-0.623656	4.255556	2.005684
C	-0.664029	2.880828	1.977171
N	1.648220	1.875778	-0.487638
N	1.495577	0.540295	-0.303925
C	2.313159	-0.034822	-1.168798
H	2.376344	-1.111103	-1.214756
C	3.006569	0.924183	-1.927462
H	3.734533	0.754777	-2.702616
C	2.558220	2.132312	-1.462662
H	2.816431	3.138507	-1.750091
N	-1.499630	2.103382	2.806579
N	-1.497025	0.754819	2.658941
C	-2.358736	0.295180	3.549226
H	-2.536946	-0.766376	3.626287
C	-2.933016	1.343725	4.288346
H	-3.662821	1.275441	5.077178
C	-2.361152	2.482582	3.786002
H	-2.503255	3.516891	4.052786
H	-1.242852	4.830562	2.680857
H	-2.094231	-4.563200	-0.235334
S	0.300780	6.651435	1.160764
S	-0.744590	-6.612377	1.407077
C	1.525221	7.029845	-0.071754
C	3.453235	7.624039	-1.991374

C	1.139044	7.271697	-1.375813
C	2.877312	7.092385	0.302549
C	3.843625	7.387177	-0.643235
C	2.109724	7.565832	-2.334918
H	0.093426	7.245966	-1.656089
H	3.150656	6.928994	1.336308
H	1.799046	7.769905	-3.350535
C	-2.013516	-6.973629	0.215244
C	-3.993310	-7.557076	-1.654437
C	-3.335802	-7.023116	0.612659
C	-1.652419	-7.225187	-1.118190
C	-2.628278	-7.516156	-2.055643
C	-4.324666	-7.311506	-0.329104
H	-3.608440	-6.851392	1.646465
H	-0.607515	-7.210041	-1.397647
H	-5.355953	-7.362928	-0.007382
O	4.452895	7.901663	-2.829838
O	5.157725	7.482337	-0.403910
O	-4.859727	-7.849206	-2.625696
O	-2.405093	-7.779167	-3.349681
C	-1.066404	-7.883037	-3.796100
H	-1.124730	-8.157150	-4.846451
H	-0.525963	-8.660779	-3.246864
H	-0.539104	-6.926719	-3.700413
C	5.608872	7.387341	0.933989
H	5.413763	6.392325	1.350628
H	5.141423	8.150924	1.564121
H	6.682087	7.556723	0.897305
C	4.141367	8.248521	-4.169799
H	3.500148	9.134435	-4.207088
H	3.658010	7.416215	-4.692196
H	5.093556	8.469419	-4.645043
C	-6.229945	-8.005924	-2.292845
H	-6.728438	-8.276720	-3.219926
H	-6.653474	-7.070998	-1.911186
H	-6.367342	-8.804883	-1.557845

$[FeL_2]^{2+}$, pathway A; $\phi = 176.5°$ (minimized), $\theta' = 89.5°$ (minimized)

Fe	0.055543	0.005654	1.286064
N	-2.112562	0.013224	1.264177
C	-2.784988	1.006671	0.687245
C	-4.160076	1.053745	0.613880
C	-4.879870	-0.005973	1.178906
C	-4.177285	-1.058927	1.792385
H	-4.709769	-1.888020	2.238403
C	-2.805314	-0.989537	1.803377
N	-1.951847	2.010054	0.160671
N	-0.609722	1.878381	0.288041
C	-0.090376	2.954225	-0.271966
H	0.980511	3.085243	-0.297413
C	-1.096485	3.802549	-0.774524
H	-0.974315	4.745864	-1.279302
C	-2.271874	3.168158	-0.479722
H	-3.294020	3.449374	-0.673073

N	-1.975260	-1.970673	2.386261
N	-0.634372	-1.794076	2.330287
C	-0.106780	-2.835285	2.951924
H	0.965028	-2.923689	3.040543
C	-1.107199	-3.702519	3.421566
H	-0.983273	-4.625433	3.962298
C	-2.287152	-3.117135	3.042502
H	-3.307180	-3.430241	3.193067
N	2.199154	-0.112292	1.237297
C	2.824947	-0.728197	0.234918
C	4.188216	-0.906663	0.183177
H	4.672913	-1.407981	-0.642622
C	4.949986	-0.408392	1.249150
C	4.298823	0.253822	2.305174
C	2.930866	0.374795	2.241215
N	1.949307	-1.173730	-0.774223
N	0.623500	-0.924808	-0.643395
C	0.060579	-1.408791	-1.735359
H	-1.007548	-1.332226	-1.868688
C	1.019569	-1.981215	-2.591718
H	0.857471	-2.451017	-3.546967
C	2.214735	-1.809645	-1.946550
H	3.216877	-2.085665	-2.231428
N	2.158107	1.031355	3.218658
N	0.816467	1.114856	3.039686
C	0.362785	1.814731	4.063589
H	-0.693200	2.020001	4.149071
C	1.409083	2.194142	4.924545
C	2.540539	1.680433	4.350822
H	3.571494	1.732418	4.659692
H	4.867546	0.656707	3.132179
H	-4.682131	1.873664	0.141373
S	-6.624800	-0.097839	1.171461
S	6.687618	-0.553719	1.339204
C	7.082671	-1.410973	-0.167296
C	7.718126	-2.761466	-2.517703
C	7.088213	-2.815145	-0.177546
C	7.399405	-0.692130	-1.303749
C	7.714058	-1.374099	-2.480129
C	7.402038	-3.496509	-1.340441
H	6.868095	-3.355519	0.733323
C	-7.085368	1.388576	0.311954
C	-7.827589	3.705777	-1.041874
C	-7.359891	2.538747	1.026177
C	-7.186708	1.368541	-1.088528
C	-7.553691	2.514721	-1.771527
C	-7.728468	3.698398	0.342417
H	-7.302596	2.544463	2.107484
H	-6.997380	0.447323	-1.622973
H	-7.957461	4.590955	0.908535
H	7.416287	0.390449	-1.286137
O	-8.177800	4.756810	-1.784882
O	-7.689185	2.613990	-3.100383
O	8.011038	-3.503662	-3.586614

C	-7.556319	1.441513	-3.880954
H	-6.540713	1.033630	-3.817151
H	-7.758258	1.743178	-4.905628
H	-8.280947	0.679277	-3.576523
C	-8.565184	5.955302	-1.131528
H	-7.736236	6.377025	-0.553665
H	-9.426747	5.785995	-0.478600
H	-8.841308	6.645528	-1.924377
C	8.437599	-2.852722	-4.773045
H	7.648950	-2.209805	-5.177631
H	8.655095	-3.647452	-5.481835
H	9.342986	-2.264854	-4.594370
H	7.976362	-0.805648	-3.361975
O	7.447092	-4.828800	-1.468352
C	7.264361	-5.623984	-0.312295
H	8.013455	-5.389618	0.451010
H	7.390229	-6.653428	-0.638050
H	6.258311	-5.496747	0.104386
H	1.343209	2.765987	5.834615

$[FeL_2]^{2+}$, pathway A; $\phi = 165°$ (fixed), $\theta' = 84.1°$ (minimized)

Fe	-0.155740	0.489357	-0.014377
N	0.271724	0.786545	2.082498
C	-0.397518	0.104097	3.009410
C	-0.268857	0.327954	4.361667
C	0.625701	1.324875	4.773437
C	1.349387	2.038728	3.801322
C	1.130779	1.724547	2.481591
N	-1.249084	-0.875649	2.465628
N	-1.281070	-1.034554	1.120037
C	-2.117970	-2.031876	0.898113
H	-2.317086	-2.352829	-0.112768
C	-2.639831	-2.535573	2.104625
H	-3.340407	-3.342823	2.235549
C	-2.053853	-1.781224	3.084613
H	-2.147508	-1.821138	4.157572
N	1.790382	2.353097	1.406412
N	1.495121	1.951508	0.147504
C	2.265724	2.672007	-0.648001
H	2.212225	2.534578	-1.717106
C	3.074772	3.553325	0.091138
H	3.798905	4.257931	-0.281209
C	2.746771	3.317867	1.399930
H	3.121727	3.757640	2.309386
N	-0.594059	0.756138	-2.113593
C	0.065784	0.056665	-3.034570
C	-0.088645	0.244165	-4.389660
C	-0.993998	1.226768	-4.811657
C	-1.703222	1.962909	-3.845489
C	-1.464220	1.679876	-2.522149
N	0.936735	-0.901065	-2.482416
N	0.980295	-1.040194	-1.134886
C	1.832633	-2.022293	-0.904925
H	2.042144	-2.327104	0.108848

C	2.355716	-2.533679	-2.107784
H	3.068306	-3.331396	-2.232147
C	1.757874	-1.797053	-3.093994
H	1.851679	-1.844567	-4.166615
N	-2.111788	2.329485	-1.452140
N	-1.800959	1.953220	-0.189226
C	-2.563637	2.687639	0.601258
H	-2.498072	2.570764	1.672185
C	-3.382811	3.553016	-0.145367
H	-4.104127	4.263135	0.221926
C	-3.065879	3.296466	-1.452969
H	-3.449048	3.720956	-2.366246
H	-0.826679	-0.237485	5.094776
H	2.052218	2.806553	4.095163
H	0.455934	-0.340604	-5.117492
H	-2.414282	2.720391	-4.146265
S	-1.304725	1.590048	-6.491585
S	0.901755	1.738528	6.447591
C	-0.176226	0.616004	7.306779
C	-1.845021	-1.139389	8.680710
C	0.307153	-0.649453	7.677661
C	-1.463876	1.004294	7.623477
C	-2.297761	0.119051	8.308443
C	-0.513877	-1.529501	8.360750
H	1.328670	-0.918022	7.444199
H	-1.825162	1.989744	7.357064
H	-3.299472	0.434730	8.566919
C	-0.231995	0.451050	-7.335273
C	1.453391	-1.324273	-8.665075
C	1.086311	0.836377	-7.626299
C	-0.706323	-0.790984	-7.711884
C	0.143372	-1.677892	-8.374932
C	1.932032	-0.038393	-8.285957
H	1.425697	1.826013	-7.350655
H	-1.730528	-1.076549	-7.506695
H	-0.239317	-2.642239	-8.679989
O	2.339682	-2.095008	-9.296145
O	3.203445	0.219280	-8.620312
O	-2.557862	-2.050798	9.343637
O	-0.163894	-2.754843	8.772377
C	1.178337	-3.168951	8.599309
H	1.443136	-3.228235	7.537289
H	1.241510	-4.160268	9.040895
H	1.869327	-2.495777	9.117132
C	-3.862171	-1.706314	9.782746
H	-4.223982	-2.570181	10.334168
H	-4.526364	-1.510170	8.934501
H	-3.836309	-0.835139	10.444600
C	1.917356	-3.356154	-9.791041
H	1.102927	-3.242471	-10.512823
H	1.604067	-4.016689	-8.975860
H	2.785194	-3.781611	-10.288273
C	3.710157	1.519653	-8.389857
H	3.121951	2.272963	-8.923709

H	4.726371	1.514842	-8.775968
H	3.730841	1.755633	-7.319450

$[FeL_2]^{2+}$, pathway A; $\phi = 160°$ (fixed), $\theta' = 80.7°$ (minimized)

Fe	-0.006770	0.345861	0.124323
N	-2.117688	0.689183	0.446903
C	-3.021761	-0.004762	-0.241037
C	-4.377660	0.219689	-0.161187
C	-4.818980	1.233733	0.699215
C	-3.871412	1.962168	1.440473
C	-2.545229	1.641209	1.275878
N	-2.450631	-1.001180	-1.055401
N	-1.108389	-1.186030	-1.012582
C	-0.860802	-2.198684	-1.823804
H	0.152752	-2.542460	-1.962249
C	-2.046952	-2.685614	-2.404571
H	-2.155736	-3.499269	-3.101519
C	-3.041571	-1.900286	-1.887501
H	-4.107750	-1.916216	-2.044641
N	-1.492731	2.274563	1.966308
N	-0.229081	1.844995	1.741483
C	0.543113	2.580056	2.521866
H	1.611499	2.426230	2.520059
C	-0.217054	3.498023	3.268421
H	0.134246	4.220853	3.985076
C	-1.513716	3.270716	2.889910
H	-2.431712	3.737089	3.207584
N	2.096720	0.752580	-0.162516
C	3.008457	0.097262	0.551970
C	4.354740	0.381466	0.513631
C	4.777594	1.410361	-0.338895
C	3.822372	2.094770	-1.111853
C	2.506201	1.720241	-0.981950
N	2.455258	-0.923222	1.348606
N	1.121940	-1.157021	1.271470
C	0.892735	-2.184555	2.069427
H	-0.110492	-2.566400	2.180841
C	2.081605	-2.632348	2.675276
H	2.203651	-3.447495	3.368279
C	3.058714	-1.806886	2.187968
H	4.121044	-1.786650	2.369032
N	1.446843	2.308440	-1.701490
N	0.196210	1.824839	-1.512681
C	-0.582391	2.521820	-2.320958
H	-1.642323	2.320570	-2.351556
C	0.159453	3.469522	-3.048539
H	-0.200610	4.173375	-3.779582
C	1.452140	3.302324	-2.627641
H	2.357832	3.808672	-2.918325
H	5.075395	-0.165442	1.105038
H	4.126300	2.882922	-1.787413
H	-4.188566	2.740261	2.121592
H	-5.091988	-0.362585	-0.726061
S	6.448686	1.892356	-0.505211

S	-6.503071	1.641555	0.920913
C	7.282705	0.765377	0.587611
C	8.599545	-1.005138	2.287583
C	7.540678	1.135389	1.893273
C	7.686482	-0.488757	0.101690
C	8.339448	-1.376738	0.938101
C	8.198401	0.243444	2.742085
H	7.248482	2.112050	2.258546
H	7.501006	-0.744560	-0.932712
H	8.411231	0.546826	3.758039
C	-7.325758	0.485623	-0.150169
C	-8.638247	-1.308831	-1.828126
C	-7.736807	-0.737550	0.344090
C	-7.571654	0.841345	-1.486053
C	-8.223982	-0.042138	-2.328096
C	-8.388984	-1.635287	-0.502300
H	-7.566786	-0.999605	1.380951
H	-7.268409	1.816594	-1.842390
H	-8.720150	-2.585103	-0.105229
O	9.238289	-1.923554	3.013887
O	8.773229	-2.593572	0.585946
C	9.623351	-1.599537	4.340581
H	10.289458	-0.731526	4.355612
H	10.154647	-2.471470	4.712965
H	8.748758	-1.409716	4.971428
C	8.656007	-2.985271	-0.768967
H	9.106402	-3.972609	-0.831999
H	9.194213	-2.296282	-1.427964
H	7.605639	-3.046863	-1.076332
O	-9.260337	-2.090513	-2.712067
O	-8.520559	0.190123	-3.613342
C	-8.256698	1.474104	-4.145761
H	-7.181839	1.689848	-4.151712
H	-8.786944	2.251551	-3.586011
H	-8.623739	1.451707	-5.168780
C	-9.791905	-3.330522	-2.272322
H	-8.998795	-3.998948	-1.921352
H	-10.274144	-3.768070	-3.142548
H	-10.532668	-3.182418	-1.480532

$[FeL_2]^{2+}$, pathway A; $\phi = 155°$ (fixed), $\theta' = 77.1°$ (minimized)

Fe	0.152937	-0.012577	0.049882
N	0.649046	-2.073685	0.487954
C	0.050227	-3.056500	-0.179841
C	0.188238	-4.388961	0.137815
C	1.014809	-4.715038	1.221777
C	1.662145	-3.682725	1.924167
C	1.443288	-2.390793	1.509331
N	-0.737074	-2.593245	-1.251917
N	-0.781673	-1.260290	-1.496279
C	-1.533146	-1.119184	-2.574073
H	-1.722189	-0.134316	-2.972844
C	-1.986366	-2.365959	-3.043454
H	-2.610870	-2.562996	-3.898272

C	-1.454697	-3.283684	-2.177794
H	-1.527443	-4.358829	-2.155981
N	2.033634	-1.261294	2.109725
N	1.744617	-0.041817	1.599388
C	2.441873	0.820968	2.316945
H	2.377086	1.876754	2.101363
C	3.197097	0.165222	3.305743
H	3.860033	0.602809	4.032752
C	2.912330	-1.164551	3.141419
H	3.269497	-2.031547	3.672342
N	-0.351439	2.031091	0.548356
C	0.299488	3.033503	-0.036122
C	0.183439	4.348432	0.354665
C	-0.681768	4.634447	1.419757
C	-1.384804	3.581929	2.033071
C	-1.179028	2.310111	1.554047
N	1.119802	2.610400	-1.099937
N	1.161789	1.288885	-1.401516
C	1.948884	1.188033	-2.458044
H	2.143923	0.219706	-2.892844
C	2.427306	2.449863	-2.856757
H	3.081157	2.678239	-3.681326
C	1.873776	3.333942	-1.969928
H	1.951621	4.406725	-1.900208
N	-1.818823	1.163733	2.065179
N	-1.524160	-0.034841	1.509708
C	-2.273352	-0.917605	2.145506
H	-2.214936	-1.962357	1.880516
C	-3.069776	-0.296409	3.124336
H	-3.778479	-0.755641	3.792427
C	-2.754017	1.033907	3.042145
H	-3.125575	1.880937	3.594982
H	0.726825	5.145416	-0.133222
H	-2.064048	3.779595	2.851299
H	-0.307424	-5.169805	-0.421730
H	2.312419	-3.911212	2.757744
S	-0.943818	6.246404	2.039335
S	1.306582	-6.353876	1.751381
C	0.091728	7.236916	0.987055
C	1.697641	8.806173	-0.659821
C	1.382113	7.538163	1.377301
C	-0.426158	7.721154	-0.225214
C	0.363510	8.501295	-1.051456
C	2.184163	8.322899	0.546920
H	1.769933	7.182767	2.323833
H	-1.449844	7.498106	-0.494343
H	3.188045	8.568835	0.865530
C	0.314188	-7.308282	0.627074
C	-1.244070	-8.825947	-1.112311
C	0.867933	-7.788371	-0.544175
C	-1.022177	-7.585063	0.958128
C	-1.805352	-8.338202	0.101149
C	0.081362	-8.545007	-1.414196
H	1.905366	-7.593121	-0.785515

H	-1.423181	-7.225596	1.896314
H	0.525610	-8.931171	-2.321579
O	2.379603	9.566755	-1.517176
O	-0.021952	9.023849	-2.222285
C	3.684616	9.993882	-1.160012
H	4.364699	9.142019	-1.055449
H	3.668176	10.574081	-0.232407
H	4.020657	10.626187	-1.977653
C	-1.367973	8.855336	-2.625946
H	-1.603737	7.797765	-2.792036
H	-1.465598	9.396191	-3.563825
H	-2.058532	9.277419	-1.888467
O	-2.072717	-9.544742	-1.870656
O	-3.085009	-8.672265	0.313173
C	-1.565936	-10.152584	-3.048527
H	-0.750411	-10.843337	-2.813961
H	-1.222520	-9.400104	-3.766104
H	-2.398522	-10.705851	-3.475052
C	-3.675409	-8.333214	1.553077
H	-3.127213	-8.781566	2.388150
H	-4.682870	-8.740526	1.522436
H	-3.729126	-7.246156	1.685060

$[FeL_2]^{2+}$, pathway A; ϕ = 150° (fixed), θ' = 74.3° (minimized)

Fe	0.076961	-0.011319	-0.102923
N	-0.299724	2.043189	0.471868
C	0.383910	3.022619	-0.113022
C	0.366209	4.328964	0.320826
C	-0.434465	4.631975	1.431094
C	-1.176885	3.605230	2.041566
C	-1.069924	2.339094	1.516504
N	1.131707	2.583973	-1.223546
N	1.088398	1.270519	-1.558735
C	1.827028	1.155007	-2.648874
H	1.951855	0.189156	-3.114037
C	2.357627	2.398939	-3.036132
H	2.989465	2.613477	-3.881287
C	1.888743	3.287947	-2.106128
H	2.027438	4.352890	-2.014104
N	-1.761483	1.218310	2.014972
N	-1.571725	0.025830	1.403386
C	-2.351390	-0.829410	2.040074
H	-2.375570	-1.864617	1.735364
C	-3.061891	-0.196060	3.075817
H	-3.771547	-0.633575	3.757328
C	-2.660851	1.112665	3.027807
H	-2.950114	1.960592	3.626580
N	0.508508	-2.064714	0.442783
C	-0.112428	-3.057104	-0.190120
C	-0.004732	-4.380469	0.172454
C	0.816578	-4.688478	1.265699
C	1.490163	-3.647856	1.929311
C	1.300763	-2.365943	1.470434
N	-0.891414	-2.615846	-1.278494

N	-0.894395	-1.294463	-1.582836
C	-1.653427	-1.176285	-2.658526
H	-1.815054	-0.204255	-3.098816
C	-2.151736	-2.426746	-3.067107
H	-2.791663	-2.640895	-3.906268
C	-1.641404	-3.322130	-2.165645
H	-1.750390	-4.392051	-2.094454
N	1.925797	-1.233238	2.025679
N	1.688086	-0.029588	1.455891
C	2.405085	0.839368	2.145680
H	2.381613	1.886265	1.882879
C	3.122609	0.203161	3.174894
H	3.790405	0.649895	3.891820
C	2.792641	-1.121558	3.066255
H	3.108959	-1.975426	3.642350
H	-0.517066	-5.168642	-0.361197
H	2.142405	-3.862822	2.764895
H	0.936243	5.107663	-0.166298
H	-1.811081	3.818020	2.891540
S	-0.569668	6.236114	2.109057
S	1.073657	-6.314985	1.848507
C	0.466319	7.199099	1.031884
C	2.071599	8.727062	-0.653614
C	-0.082951	7.740916	-0.141750
C	1.787807	7.423505	1.365524
C	2.589203	8.188099	0.515862
C	0.706190	8.500672	-0.987109
H	-1.128547	7.576762	-0.365006
H	2.201231	7.024379	2.283361
H	3.618365	8.374635	0.790656
C	0.076462	-7.284877	0.741429
C	-1.490927	-8.818305	-0.976390
C	-1.262569	-7.545714	1.074295
C	0.628332	-7.788854	-0.420712
C	-0.162856	-8.552536	-1.280292
C	-2.049542	-8.308484	0.229492
H	-1.663794	-7.167426	2.004771
H	1.667825	-7.606508	-0.663336
H	0.279940	-8.955353	-2.180875
O	2.749878	9.475156	-1.524596
O	0.293750	9.071937	-2.125303
O	-2.324824	-9.541443	-1.725016
O	-3.331412	-8.630319	0.445900
C	4.092005	9.824605	-1.224774
H	4.420479	10.457031	-2.045397
H	4.729873	8.936153	-1.172254
H	4.152554	10.383438	-0.286028
C	-1.078819	8.992483	-2.461460
H	-1.385942	7.954911	-2.636309
H	-1.191638	9.560400	-3.381421
H	-1.703619	9.436830	-1.679928
C	-3.918824	-8.271562	1.681946
H	-3.969419	-7.182731	1.798036
H	-3.369851	-8.708853	2.522268

H	-4.927484	-8.676248	1.659203
C	-1.823379	-10.166738	-2.895960
H	-1.012704	-10.860582	-2.653906
H	-1.475137	-9.425250	-3.622579
H	-2.660400	-10.718680	-3.315377

$[FeL_2]^{2+}$, pathway A; ϕ = 145° (fixed), θ' = 71.8° (minimized)

Fe	0.285974	-0.025330	0.139084
N	-0.343409	-2.070727	0.503292
C	0.256151	-3.058939	-0.155803
C	-0.170485	-4.366986	-0.125984
C	-1.294466	-4.664359	0.657133
C	-1.922242	-3.629653	1.372255
C	-1.398765	-2.362932	1.260587
N	1.380269	-2.631237	-0.891871
N	1.731268	-1.322173	-0.845001
C	2.829500	-1.218825	-1.574048
H	3.306615	-0.258326	-1.695255
C	3.206149	-2.466698	-2.101769
H	4.053561	-2.690932	-2.727179
C	2.260803	-3.344583	-1.642327
H	2.156125	-4.407685	-1.785744
N	-1.909506	-1.240579	1.938159
N	-1.285696	-0.052892	1.766006
C	-1.942400	0.806543	2.524114
H	-1.635499	1.841101	2.557015
C	-3.002627	0.179575	3.204087
H	-3.702915	0.621853	3.892197
C	-2.949640	-1.129473	2.805922
H	-3.562602	-1.973379	3.076388
N	-0.389719	2.007713	-0.205452
C	0.163855	2.997519	0.489207
C	-0.323857	4.284455	0.505881
C	-1.456710	4.557806	-0.273587
C	-2.034257	3.521747	-1.028612
C	-1.455781	2.276299	-0.955638
N	1.303704	2.593257	1.213766
N	1.691936	1.295492	1.147096
C	2.797672	1.214395	1.867634
H	3.302553	0.266353	1.973493
C	3.142242	2.465826	2.408760
H	3.987979	2.706507	3.030313
C	2.169440	3.322196	1.966294
H	2.037502	4.380737	2.120994
N	-1.914316	1.152183	-1.667442
N	-1.247309	-0.014900	-1.514919
C	-1.857645	-0.879203	-2.305354
H	-1.508737	-1.899399	-2.358896
C	-2.931087	-0.276330	-2.986469
H	-3.602892	-0.726975	-3.697174
C	-2.935169	1.023306	-2.555039
H	-3.575111	1.850218	-2.815505
H	0.139488	5.070273	1.085704
H	-2.899757	3.712162	-1.648690

H	0.335254	-5.152677	-0.669496
H	-2.780391	-3.837383	1.996962
S	-2.200066	6.135953	-0.367124
S	-1.958480	-6.272460	0.813145
C	-0.858107	-7.242333	-0.191666
C	0.853373	-8.771055	-1.770181
C	0.265169	-7.814042	0.374048
C	-1.155341	-7.431683	-1.550851
C	-0.313190	-8.191628	-2.343816
C	1.122321	-8.574746	-0.422522
H	0.479202	-7.685713	1.427798
H	-2.057429	-7.000647	-1.963706
H	1.992219	-9.030680	0.030347
C	-1.135309	7.124090	0.658164
C	0.532335	8.688835	2.247368
C	-1.447873	7.321797	1.989016
C	0.007586	7.711220	0.091542
C	0.843576	8.489809	0.872598
C	-0.607609	8.105552	2.782111
H	-2.342708	6.886966	2.416711
H	0.215046	7.567232	-0.960320
H	-0.866986	8.270890	3.818998
O	1.393201	9.456472	2.916691
O	1.953238	9.103664	0.442894
O	1.602389	-9.485429	-2.611319
O	-0.499421	-8.449093	-3.644919
C	1.106311	9.787534	4.266250
H	1.910780	10.445503	4.584297
H	1.094849	8.894034	4.899238
H	0.150667	10.313999	4.346892
C	2.259134	9.054077	-0.938201
H	3.156912	9.653757	-1.064174
H	1.448717	9.480746	-1.537980
H	2.461071	8.027358	-1.264403
C	-1.699534	-8.012706	-4.253877
H	-1.765940	-6.918378	-4.263855
H	-2.575301	-8.431995	-3.747896
H	-1.663095	-8.379092	-5.276708
C	2.730237	-10.179600	-2.101499
H	3.472761	-9.484373	-1.695924
H	3.156716	-10.709177	-2.949347
H	2.433530	-10.899600	-1.332908

$[FeL_2]^{2+}$, pathway A; ϕ = 140° (fixed), θ' = 69.7° (minimized)

Fe	0.124813	-0.015363	-0.538585
N	2.076180	0.037480	0.419488
C	2.918489	-0.968534	0.199754
C	4.090990	-1.151792	0.896348
C	4.414977	-0.205633	1.879055
C	3.541133	0.871772	2.103302
C	2.397363	0.940703	1.342363
N	2.489073	-1.837714	-0.825834
N	1.312031	-1.583753	-1.450201
C	1.192450	-2.513032	-2.383664

H	0.321433	-2.527143	-3.020898
C	2.297600	-3.381919	-2.376475
H	2.477685	-4.226408	-3.019872
C	3.106004	-2.917758	-1.373102
H	4.063190	-3.266068	-1.020641
N	1.449921	1.974477	1.445504
N	0.376406	1.940035	0.623670
C	-0.319398	3.026850	0.905309
H	-1.238790	3.232252	0.377881
C	0.301926	3.783651	1.915826
H	-0.028048	4.714830	2.344213
C	1.431780	3.080083	2.236086
H	2.210697	3.285573	2.951692
N	-1.971873	0.310964	-0.065903
C	-2.691605	1.082801	-0.875167
C	-3.946623	1.554711	-0.565066
C	-4.495233	1.172906	0.667696
C	-3.752829	0.332732	1.515118
C	-2.505598	-0.064894	1.093076
N	-2.032323	1.378800	-2.087290
N	-0.778818	0.898402	-2.283798
C	-0.435586	1.279155	-3.502877
H	0.535951	1.019051	-3.894547
C	-1.469062	2.009970	-4.114540
H	-1.478181	2.441468	-5.101094
C	-2.472936	2.049859	-3.183590
H	-3.456477	2.488908	-3.225248
N	-1.659953	-0.913763	1.829956
N	-0.456037	-1.229091	1.300190
C	0.106942	-2.052519	2.165789
H	1.091137	-2.450760	1.970071
C	-0.733465	-2.284874	3.270289
H	-0.546969	-2.912494	4.125154
C	-1.855552	-1.541255	3.019456
H	-2.763109	-1.421491	3.587882
H	-4.506159	2.191684	-1.235613
H	-4.160836	0.012229	2.464179
H	4.757259	-1.979855	0.699663
H	3.776193	1.622987	2.845007
S	5.873108	-0.279280	2.838830
S	-6.073393	1.685833	1.215410
C	6.663315	-1.745805	2.217909
C	7.893617	-4.051725	1.253720
C	7.524667	-1.661778	1.140857
C	6.411263	-2.977384	2.843157
C	7.021070	-4.128455	2.375373
C	8.132787	-2.821172	0.657013
H	7.737253	-0.706747	0.676695
H	5.757391	-3.014649	3.704326
H	8.816521	-2.745140	-0.177523
C	-6.642321	2.686315	-0.139639
C	-7.551867	4.246592	-2.258524
C	-6.432272	4.051623	-0.122440
C	-7.318844	2.073887	-1.207022

C	-7.773268	2.839926	-2.266288
C	-6.887860	4.829278	-1.188318
H	-5.931140	4.523046	0.713831
H	-7.497182	1.007364	-1.181314
H	-6.734675	5.899479	-1.160285
O	8.431205	-5.211731	0.874288
O	6.868076	-5.352090	2.898228
O	-8.031335	4.899238	-3.318206
O	-8.432142	2.371545	-3.333377
C	-8.793229	1.002988	-3.355192
H	-9.426818	0.748197	-2.499521
H	-7.906973	0.358039	-3.365262
H	-9.353013	0.858947	-4.275742
C	-7.943426	6.315076	-3.345860
H	-8.431618	6.621546	-4.267245
H	-6.899852	6.646798	-3.357883
H	-8.465775	6.757442	-2.492246
C	6.138472	-5.485317	4.103229
H	5.085479	-5.213654	3.964103
H	6.576662	-4.874710	4.899497
H	6.204512	-6.535741	4.375188
C	9.387221	-5.210173	-0.174348
H	8.941503	-4.871387	-1.115449
H	9.710145	-6.242661	-0.278634
H	10.246571	-4.581784	0.078651

References

(1) Halcrow, M. A. Iron(II) Complexes of 2,6-Di(pyrazol-1-yl)pyridines – a Versatile System for Spin-Crossover Research. *Coord. Chem. Rev.* **2009**, *253*, 2493–2514.

(2) (a) Šalitroš, I.; Herchel, R.; Fuhr, O.; González-Prieto, R.; Ruben, M. Polynuclear Iron(II) Complexes with 2,6-Bis(pyrazol-1-yl)pyridineanthracene Ligands Exhibiting Highly Distorted High-Spin Centers. *Inorg. Chem.* **2019**, *58*, 4310−4319.
(b) Kulmaczewski, R.; Armstrong, I. T.; Catchpole, P.; Ratcliffe, E. S. J.; Vasili, H. B.; Warriner, S. L.; Cespedes, O.; Halcrow, M. A. Di-ron(II) [2+2] Helicates of Bis-(Dipyrazolylpyridine) Ligands: the Influence of the Ligand Linker Group on Spin State Properties. *Chem. – Eur. J.* **2023**, *29*, e202202578.

(3) Sheldrick, G. M. *SHELXT* – Integrated Space-Group and Crystal Structure Determination. *Acta Cryst. Sect. A* **2015**, *71*, 3–8.

(4) Sheldrick, G. M. Crystal Structure Refinement with *SHELXL*. *Acta Cryst. Sect. C* **2015**, *71*, 3–8.

(5) Barbour, L. J. *X-Seed 4*: Updates to a Program for Small-Molecule Supramolecular Crystallography. *J. Appl. Cryst.* **2020**, *53*, 1141–1146.

(6) Dolomanov, O. V.; Bourhis, L. J.; Gildea, R. J.; Howard, J. A. K.; Puschmann, H. *OLEX2*: a Complete Structure Solution, Refinement and Analysis Program. *J. Appl. Cryst.* **2009**, *42*, 339–341.

(7) Guionneau, P.; Marchivie, M.; Bravic, G.; Létard, J.-F.; Chasseau, D. Structural Aspects of Spin Crossover. Example of the $[Fe^{II}L_n(NCS)_2]$ Complexes. *Top. Curr. Chem.* **2004**, *234*, 97–128.

(8) Capel Berdiell, I.; Kulmaczewski, R.; Halcrow, M. A. Iron(II) complexes of 2,4-Dipyrazolyl-1,3,5-triazine Derivatives – the Influence of Ligand Geometry on Metal Ion Spin State. *Inorg. Chem.* **2017**, *56*, 8817–8828.

(9) McCusker, J. K.; Rheingold, A. L.; Hendrickson, D. N. Variable-Temperature Studies of Laser-Initiated $^5T_2 \rightarrow {}^1A_1$ Intersystem Crossing in Spin-Crossover Complexes: Empirical Correlations between Activation Parameters and Ligand Structure in a Series of Polypyridyl Ferrous Complexes. *Inorg. Chem.* **1996**, *35*, 2100–2112.

(10) Halcrow, M. A. Structure:Function Relationships in Molecular Spin-Crossover Complexes. *Chem. Soc. Rev.* **2011**, *40*, 4119–4142.

(11) Holland, J. M.; McAllister, J. A.; Kilner, C. A.; Thornton-Pett, M.; Bridgeman, A. J.; Halcrow, M. A. Stereochemical Effects on the Spin-State Transition Shown by Salts of $[FeL_2]^{2+}$ [L = 2,6-Di(pyrazol-1-yl)pyridine]. *J. Chem. Soc., Dalton Trans.* **2002**, *2002*, 548–554.

(12) Kershaw Cook, L. J.; Thorp-Greenwood, F. L.; Comyn, T. P.; Cespedes, O.; Chastanet, G.; Halcrow, M. A. Unexpected Spin-Crossover and a Low Pressure Phase Change in an Iron(II)/Dipyrazolyl-pyridine Complex Exhibiting a High-Spin Jahn-Teller Distortion. *Inorg. Chem.* **2015**, *54*, 6319−6330.

(13) Capel Berdiell, I.; Kulmaczewski, R.; Shahid, N.; Cespedes, O.; Halcrow, M. A. The Number and Shape of Lattice Solvent Molecules Controls Spin-Crossover in an Isomorphous Series of Crystalline Solvate Salts. *Chem. Commun.* **2021**, *57*, 6566−6569.

(14) Suryadevara, N.; Mizuno, A.; Spieker, L.; Salamon, S.; Sleziona, S.; Maas, A.; Pollmann, E.; Heinrich, B.; Schleberger, M.; Wende, H.; Kuppusamy, S. K.; Ruben, M. Structural Insights into Hysteretic Spin-Crossover in a Set of Iron(II)-2,6-Bis(1*H*-pyrazol-1-yl)pyridine) Complexes. *Chem. − Eur. J.* **2022**, *28*, e202103853.

(15) Kulmaczewski, R.; Kershaw Cook, L. J.; Pask, C. M.; Cespedes, O.; Halcrow, M. A. Iron(II) Complexes of 4-(Alkyldisulfanyl)-2,6-di(pyrazolyl)pyridine Derivatives. Correlation of Spin-Crossover

Cooperativity with Molecular Structure Following Single-Crystal-to-Single-Crystal Desolvation. *Cryst. Growth Des* **2022**, *22*, 1960−1971.

(16) Kershaw Cook, L. J.; Mohammed, R.; Sherborne, G.; Roberts, T. D.; Alvarez, S.; Halcrow, M. A. Spin State Behaviour of Iron(II)/Dipyrazolylpyridine Complexes. New Insights from Crystallographic and Solution Measurements. *Coord. Chem. Rev.* **2015**, *289–290*, 2–12.2.

(17) Vela, S.; Novoa, J. J.; Ribas-Arino, J. Insights into the Crystal-Packing Effects on the Spin Crossover of $[Fe^{II}(1\text{-}bpp)_2]^{2+}$-Based Materials. *Phys. Chem. Chem. Phys.* **2014**, *16*, 27012–27024.

(18) Elhaïk, J.; Evans, D. J.; Kilner, C. A.; Halcrow, M. A. A Structural, Magnetic and Mössbauer Spectroscopic Study of an Unusual Angular Jahn-Teller Distortion in a Series of High-Spin Iron(II) Complexes. *Dalton Trans.* **2005**, 1693–1700.

(19) Kershaw Cook, L. J.; Kulmaczewski, R.; Mohammed, R.; Dudley, S.; Barrett, S. A.; Little, M. A.; Deeth, R. J.; Halcrow, M. A. A Unified Treatment of the Relationship Between Ligand Substituents and Spin State in a Family of Iron(II) Complexes. *Angew. Chem. Int. Ed.* **2016**, *55*, 4327–4331.

(20) Hansch, C.; Leo, A.; Taft, R. W. A Survey of Hammett Substituent Constants and Resonance and Field Parameters. *Chem. Rev.* **1991**, *91*, 165–195.

(21) Elhaïk, J.; Kilner, C. A.; Halcrow, M. A. Structural Diversity in Iron(II) Complexes of 2,6-Di(Pyrazol-1-yl)pyridine and 2,6-Di(3-methylpyrazol-1-yl)pyridine. *Dalton Trans.* **2006**, 823–830.

(22) Kulmaczewski, R.; Halcrow, M. A. CDS private communication.

(23) Oulmidi, A.; Radi, S.; Idir, A.; Zyad, A.; Kabach, I.; Nhiri, M.; Robeyns, K.; Rotaru, A.; Garcia, Y. Synthesis and Cytotoxicity Against Tumor Cells of Pincer N-Heterocyclic Ligands and their Transition Metal Complexes. *RSC Adv.* **2021**, *11*, 34742–34753.

(24) Galadzhun, I.; Kulmaczewski, R.; Shahid, N.; Cespedes, O.; Howard, M. J.; Halcrow, M. A. The Flexibility of Long Chain Substituents Influences Spin-Crossover in Isomorphous Lipid Bilayer Crystals. *Chem. Commun.* **2021**, *57*, 4039–4042.

(25) Kuramochi, S.; Shiga, T.; Cameron, J. M.; Newton, G. N.; Oshio, H. Synthesis, Crystal Structures and Magnetic Properties of Composites Incorporating an Fe(II) Spin Crossover Complex and Polyoxo-metalates. *Inorganics* **2017**, *5*, 48.

(26) Hasegawa, Y.; Sakamoto, R.; Takahashi, K.; Nishihara, H. Bis[(*E*)-2,6-bis(1*H*-pyrazol-1-yl)-4-styryl-pyridine]iron(II) Complex: Relationship between Thermal Spin Crossover and Crystal Solvent. *Inorg. Chem.* **2013**, *52*, 1658−1665.

(27) Takahashi, K.; Hasegawa, Y.; Sakamoto, R.; Nishikawa, M.; Kume, S.; Nishibori, E.; Nishihara, H. Solid-State Ligand-Driven Light-Induced Spin Change at Ambient Temperatures in Bis(dipyrazolyl-styrylpyridine)iron(II) Complexes. *Inorg. Chem.* **2012**, *51*, 5188−5198.

(28) González-Prieto, R.; Fleury, B.; Schramm, F.; Zoppellaro, G.; Chandrasekar, R.; Fuhr, O.; Lebedkin, S.; Kappes, M.; Ruben, M. Tuning the Spin-Transition Properties of Pyrene-Decorated 2,6-Bis-pyrazolylpyridine Based Fe(II) Complexes. *Dalton Trans.* **2011**, *40*, 7564–7570.

(29) Bridonneau, N.; Rigamonti, L.; Poneti, G.; Pinkowicz, D.; Forni A.; Cornia, A. Evidence of Crystal Packing Effects in Stabilizing High or Low Spin States of Iron(II) Complexes with Functionalized 2,6-Bis(pyrazol-1-yl)pyridine Ligands. *Dalton Trans.* **2017**, *46*, 4075–4085.

(30) Galadzhun, I.; Kulmaczewski, R.; Halcrow, M. A. Five 2,6-Di(pyrazol-1-yl)pyridine-4-carboxylate Esters, and the Spin States of their Iron(II) Complexes. *Magnetochemistry* **2019**, *5*, 9.

(31) Mohammed, R.; Halcrow, M. A. CDS private communication.

(32) Capel Berdiell, I.; Kulmaczewski, R.; Cespedes, O.; Halcrow, M. A. An Incomplete Spin-Transition Associated with a *Z*' = 1 → *Z*' = 24 Crystallographic Symmetry Breaking. *Chem. – Eur. J.* **2018**, *24*, 5055–5059.

(33) Capel Berdiell, I.; Kulmaczewski, R.; Warriner, S. L.; Cespedes, O.; Halcrow, M. A. Iron and Silver Complexes of 4-(Imidazol-1-yl)-2,6-di(pyrazol-1-yl)pyridine (*L*), Including a $[Fe_3(\mu\text{-}F)_2F_6L_8]^+$ Assembly. *Eur. J. Inorg. Chem.* **2020**, 4334–4340.

(34) Pukenas, L.; Benn, F.; Lovell, E.; Santoro, A.; Kershaw Cook, L. J.; Halcrow, M. A.; Evans, S. D. Bead-Like Structures and Self-Assembled Monolayers from 2,6-Dipyrazolylpyridines and their Iron(II) Complexes. *J. Mater. Chem. C* **2015**, *3*, 7890−7896.

(35) Kershaw Cook¸ L. J.; Halcrow, M. A. Synthesis of 4-Hydroxy-2,6-di(pyrazol-1-yl)pyridine, and the Spin State Behaviour of its Iron(II) Complex Salts. *Magnetochemistry* **2015**, *1*, 3–16.

(36) Kershaw Cook, L. J.; Kulmaczewski, R.; Cespedes, O.; Halcrow, M. A. Different Spin State Behaviors in Isostructural Solvates of a Molecular Iron(II) Complex. *Chem. – Eur. J.* **2016**, *22*, 1789–1799.

(37) Kulmaczewski, R.; Trzop, E.; Kershaw Cook, L. J.; Collet, E.; Chastanet, G.; Halcrow, M. A. Role of Symmetry Breaking in the Structural Trapping of Light-Induced Excited Spin States. *Chem. Commun.* **2017**, *53*, 13268–13271.

(38) Mohammed, R.; Chastanet, G.; Tuna, F.; Malkin, T. L.; Barrett, S. A.; Kilner, C. A.; Létard, J.-F.; Halcrow, M. A. The Synthesis of New 2,6-Di(pyrazol-1-yl)pyrazine Derivatives, and the Spin State Behavior of their Iron(II) Complexes. *Eur. J. Inorg. Chem.* **2013**, *2013*, 819–831.

(39) Nihei, M.; Tahira, H.; Takahashi, N.; Otake, Y.; Yamamura, Y.; Saito, K.; Oshio, H. Multiple Bistability and Tristability with Dual Spin-State Conversions in $[Fe(dpp)_2][Ni(mnt)_2]_2 \cdot MeNO_2$. *J. Am. Chem. Soc.* **2010**, *132*, 3553–3560.

(40) Kershaw Cook, L. J.; Shepherd, H. J.; Comyn, T. P.; Baldé, C.; Cespedes, O.; Chastanet, G.; Halcrow, M. A. Decoupled Spin-Crossover and Structural Phase Transition in a Molecular Iron(II) Complex. *Chem. – Eur. J.* **2015**, *21*, 4805–4816.

(41) Kumar, K. S.; Heinrich, B.; Vela, S.; Moreno-Pineda, E.; Bailly, C.; Ruben, M. Bi-Stable Spin-Crossover Characteristics of a Highly Distorted $[Fe(1\text{-}BPP\text{-}COOC_2H_5)_2](ClO_4)_2 \cdot CH_3CN$ Complex**.** *Dalton Trans.* **2019**, *48*, 3825–3830.

(42) Attwood, M.; Akutsu, H.; Martin, L.; Cruickshank, D.; Turner, S. S. Above Room Temperature Spin Crossover in Thioamide-Functionalized 2,6-Bis(pyrazol-1-yl)pyridine Iron(II) Complexes. *Dalton Trans.* **2019**, *48*, 90–98.

(43) Šalitroš, I.; Fuhr, O.; Eichhöfer, A.; Kruk, R.; Pavlik, J.; Dlháň, L.; Boča, R.; Ruben, M. The Interplay of Iron(II) Spin Transition and Polymorphism. *Dalton Trans.* **2012**, *41*, 5163–5171.

(44) Kershaw Cook, L. J.; Kulmaczewski, R.; Barrett, S. A.; Halcrow, M. A. Iron(II) Complexes of 4-Sulfanyl-, 4-Sulfinyl- and 4-Sulfonyl-2,6-Dipyrazolylpyridine Ligands. A Subtle Interplay Between Spin-Crossover and Crystallographic Phase Changes. *Inorg. Chem. Front.* **2015**, *2*, 662–670.

(45) Kulmaczewski, R.; Bamiduro, F.; Cespedes, O.; Halcrow, M. A. Structural Transformations and Spin-Crossover in $[FeL_2]^{2+}$ Salts (*L* = 4-{*Tert*butylsulfanyl}-2,6-di{pyrazol-1-yl}pyridine) − the Influence of Bulky Ligand Substituents. *Chem. – Eur. J.* **2021**, *27*, 2082–2092.

(46) Michaels, E.; Pask, C. M.; Capel Berdiell, I.; Vasili, H. B.; Howard, M. J.; Cespedes, O.; Halcrow, M. A. Spin-Crossover in a New Iron(II)/Di(pyrazolyl)pyridine Complex with a Terpyridine Embrace Lattice. Thermally Induced Excited Spin State Trapping and Clarification of a Structure: Function Correlation. *Cryst. Growth Des.* **2022**, *22*, 6809–6817.
